# A taxonomic review of the freshwater shrimp genus *Caridina* H. Milne Edwards, 1837 (Decapoda, Atyidae) from southern Thailand, with descriptions of five new species

**DOI:** 10.3897/zookeys.1292.193168

**Published:** 2026-09-15

**Authors:** Kongkit Macharoenboon, Thomas von Rintelen, Werner Klotz, Chirasak Sutcharit, Warut Siriwut, Ekgachai Jeratthitikul

**Affiliations:** 1 Animal Systematics and Molecular Ecology Laboratory, Department of Biology, Faculty of Science, Mahidol University, Bangkok 10400, Thailand Animal Systematics and Molecular Ecology Laboratory, Department of Biology, Faculty of Science, Mahidol University Bangkok Thailand; 2 Museum für Naturkunde, Leibniz Institute for Evolution and Biodiversity Science, Invalidenstr. 43, 10115 Berlin, Germany Museum für Naturkunde, Leibniz Institute for Evolution and Biodiversity Science Berlin Germany; 3 Wiesenweg 1, 6063 Rum, Austria Unaffiliated Rum Austria; 4 Animal Systematics Research Unit, Department of Biology, Faculty of Science, Chulalongkorn University, Bangkok 10330, Thailand Animal Systematics Research Unit, Department of Biology, Faculty of Science, Chulalongkorn University Bangkok Thailand

**Keywords:** Amphidromous, crustacean, freshwater fauna, landlocked, Malay Peninsula, molecular phylogeny, Thailand

## Abstract

A comprehensive taxonomic review of the genus *Caridina* in southern Thailand is presented, integrating extensive field surveys with comprehensive morphological and molecular analyses to document the species diversity. Phylogenetic tree reconstructed from partial sequences of two mitochondrial genes (16S rRNA and COI) indicated 18 distinct lineages, recognized as species in our study, belonging to six species groups, from southern Thailand. Five of these are new to science and are described as *C. kuehnei***sp. nov**., *C. nangroma***sp. nov**., *C. srivijaya***sp. nov**., *C. takkola***sp. nov**., and *C. tambralinga***sp. nov**. Including historical records, there are 21 species of *Caridina* found in southern Thailand of which almost half are endemic to the region. Updated distribution maps and a key to all known species in the region are also provided. Our findings highlight that while morphological identification is challenging due to significant intraspecific variation and interspecific similarity, molecular data is essential for helping taxonomists to delineate species boundaries.

## Introduction

The freshwater shrimp genus *Caridina* H. Milne-Edwards, 1837 is one of the most species-rich genera in the family Atyidae De Haan, 1849. It currently comprises 356 species and has an extensive distribution throughout the Indo-Pacific area (de Mazancourt et al. 2021; De Grave et al. 2023; DecaNet 2025). In general, the members of the genus *Caridina* are small and often exhibit striking coloration. They occupy various types of freshwater habitats, including headwater streams, lakes, lowland rivers, estuaries, and even subterranean freshwater systems (von Rintelen et al. 2012; de Mazancourt et al. 2021; Klotz et al. 2021; Macharoenboon et al. 2024). Although they are not as important to rural and industrial aquacultures as palaemonid freshwater shrimps (Mather and de Bruyn 2003; Wowor and Ng 2007; Na-Nakorn and Jintasataporn 2012), several *Caridina* species are well-known in the aquarium trade because of their eye-catching colors (de Grave et al. 2008; Klotz and von Rintelen 2014). In terms of ecology, *Caridina* holds several important roles as a food source for predators (Hart 1981; Cornelissen et al. 2018), planktivores (Lehman et al. 1996), and basibionts for microorganisms (Fernandez-Leborans et al. 2006; Fernandez-Leborans and von Rintelen 2010). Most significantly, they serve as detritivores (Hart et al. 2003; Yam and Dudgeon 2005; Yam 2016), consuming and processing organic matter, which is vital for nutrient cycling and the overall health of the freshwater ecosystem (Wallace and Webster 1996; Covich et al. 1999).

Traditionally, species delineation of *Caridina* has mainly relied on external morphological characters such as the shape of the rostrum, telson characters, the number of setae on each appendage, and the proportion between segments on each appendage (Cai et al. 2007; von Rintelen and Cai 2009; Richard and Clark 2010, 2014; de Mazancourt et al. 2020). However, this approach is often hindered by intraspecific variation and morphological resemblance between closely related species (De Man 1908a, 1908b; Kemp 1918b; Bouvier 1925; Cai et al. 2007; Richard and Clark 2005, 2014; de Mazancourt et al. 2018a, 2020). To overcome these challenges, molecular phylogenetic analyses are now being used as crucial tools in integrative taxonomy. The approach provides greater precision in defining species boundaries, facilitates the uncovering of novel species, and elucidates evolutionary relationships among *Caridina* species (Klotz and von Rintelen 2014; Bernardes et al. 2017; de Mazancourt et al. 2018a, 2018b, 2019a, 2019b; Klotz et al. 2021, 2023, 2024). However, the availability of molecular data, including DNA sequences of barcoding markers, remain limited for *Caridina* across a number of key geographical areas.

**Figure 1.**
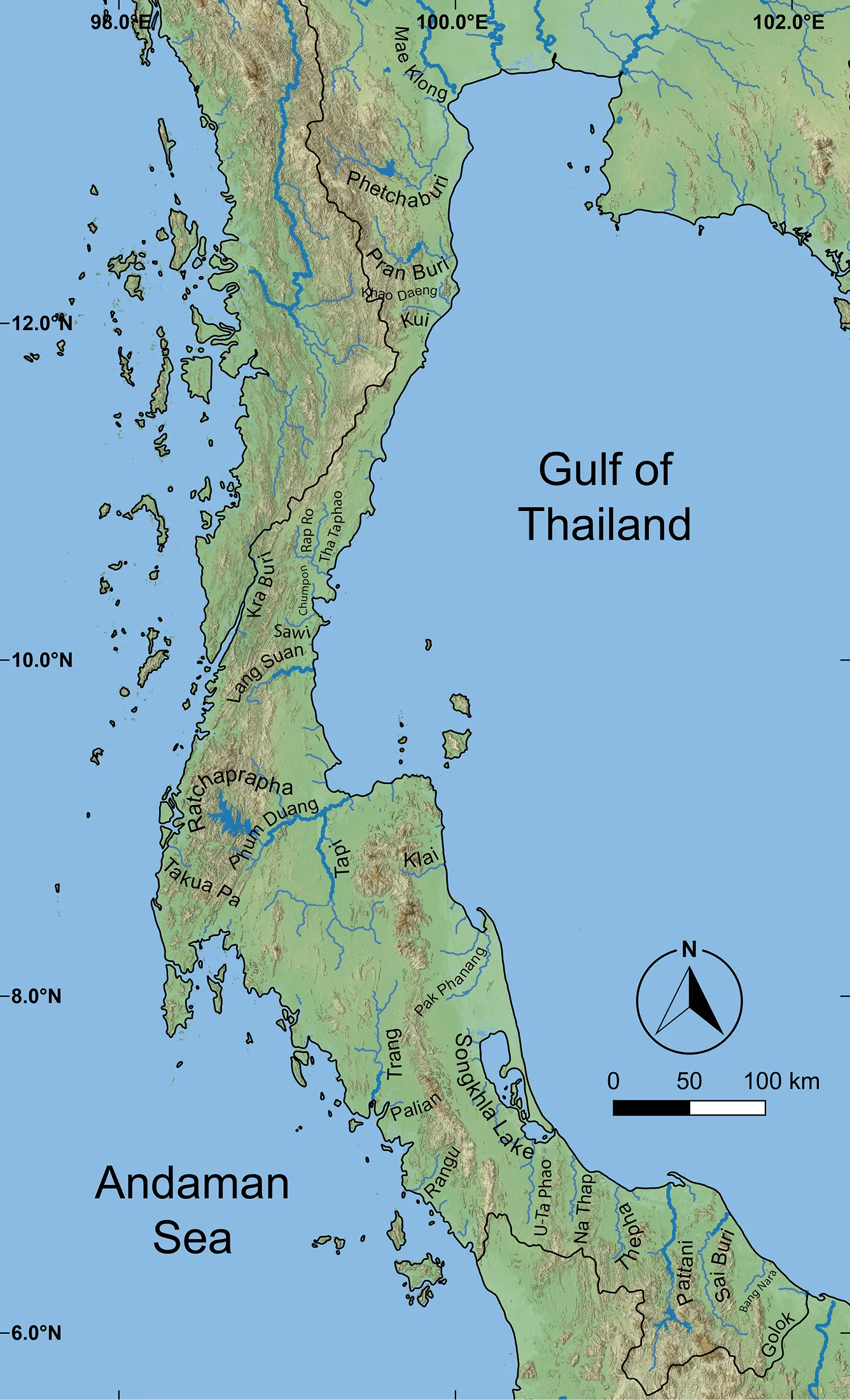
The river systems of southern Thailand. Map was generated in QGIS v. 3.24.3 by compiling topographic base maps of rivers and lakes from the HydroSHEDS database (available at https://www.hydrosheds.org) and map raster data from NASA EARTHDATA (available at https://www.earthdata.nasa.gov/).

Characterized as a part of the Malay Peninsula, the southern region of Thailand is bordered on the west by the Andaman Sea and on the east by the Gulf of Thailand, extending from the Phetchaburi Basin, at approximately 12.9°N, southward to the Malaysia border (Fig. 1). This north-south oriented landmass covers the transition zone between the Indo-Burma and Sundaland biodiversity hotspots (Myers et al. 2000; Woodruff 2003, 2010; de Bruyn et al. 2014). Its geographical configuration, together with an extensive network of river basins, gives rise to a wide range of aquatic habitats and diverse ecosystems, including coastal basins, lowland rivers, lakes, and headwater streams. Collectively, these features contribute to the region’s exceptional freshwater biodiversity (Pookpakdi 2000; Abell et al. 2008; Kottelat 2013; Pfeiffer et al. 2021; Konopleva et al. 2023; Thepprasit et al. 2025).

To date, 13 species of *Caridina* have been reported from southern Thailand, viz. *C. typus* H. Milne Edwards, 1837, *C. cf. laevis* Heller, 1862, *C. gracilirostris* De Man, 1892, *C. sumatrensis* De Man, 1892, *C. gracillima* Lanchester, 1902, *C. aff. multidentata* Stimpson, 1860, *C. macrophora* Kemp, 1918, *C. peninsularis* Kemp, 1918, *C. lanceifrons* Yu, 1936, *C. temasek* Choy & Ng, 1991, *C. johnsoni* Cai et al., 2007, *C. thai* Cai & Naiyanetr, 2024, and *C. kottelati* Cai & Naiyanetr, 2024 (Lanchester 1902; Kemp 1918b; Tiwari and Pillai 1971; Cai et al. 2007; Naiyanetr 2007; Do et al. 2021b; Cai and Naiyanetr 2024). Four of these species (*C. gracillima*, *C. kottelati*, *C. macrophora*, and *C. peninsularis*) were originally described from this region (Lanchester 1902; Kemp 1918b; Cai and Naiyanetr 2024).

Although several studies have investigated the genus *Caridina* in southern Thailand (Lanchester 1902; Kemp 1918b; Kamita 1966; Cai et al. 2007; Cai and Ng 2007; Do et al. 2021b; Cai and Naiyanetr 2024), the regional species inventory remains incomplete, and integrative molecular-based taxonomic approaches have not yet been applied comprehensively. Consequently, the existence of undescribed or previously unrecorded species, particularly those with restricted distributions in highly localized habitats or representing cryptic species, cannot be excluded. Therefore, this study aims to clarify the taxonomy of *Caridina* in southern Thailand by integrating comparative morphological data with molecular-based analyses, thereby providing a more robust and comprehensive taxonomic framework for the genus in the region, and, more broadly, in Southeast Asia.

## Materials and methods

### Specimen sampling

Freshwater shrimps were collected by dip net near the banks of rivers and streams throughout southern Thailand, including Phetchaburi and Prachuab Khiri Khan provinces. The live color of the specimens was recorded. Selected shrimps were temporarily housed in a small glass aquarium to take photos using a Nikon D7200 with an AF-S VR Nikkor 105 mm f/2.8G IF-ED Macro Lens. Euthanasia was conducted following AVMA Guidelines (2020) under the approval of animal use protocol by the Faculty of Science, Mahidol University Animal Care and Use Committee (MUSC64-039-588 and MUSC68-009-779). Specimens were preserved in 75% and 95% (v/v) ethanol for morphological study and molecular work, respectively. Voucher specimens were deposited at Mahidol University Museum of Natural History, Salaya, Thailand (**MUMNH**), and Museum für Naturkunde, Berlin, Germany (**ZMB**).

### Morphological comparison

General external morphology and terminology of *Caridina* in this study followed the format of von Rintelen and Cai (2009), Richard and Clark (2014), and Klotz et al. (2021). All measurements were acquired from ovigerous females except first and second pleopods, which were taken from male shrimps. Specimens were examined using an Olympus CH30 microscope and a ZEISS Stemi 305 stereo microscope fitted with a digital eyepiece (Dino-Lite AM423X). Morphological characters were measured via DinoCapture software v. 2.0 and reported in millimeters. The rostral formula was written as (postorbital teeth) + entire teeth on dorsal margin + subapical teeth (if any)/ ventral teeth. For instance, (2–4) 13–18 + 1–2/ 5–9 refers to a rostrum with 13–18 dorsal teeth in total, of which 2–4 are postorbital teeth, one or two subapical teeth, and 5–9 teeth on the ventral margin.

In this study, we use the terms ‘embryo’, ‘embryo size’, and ‘the number of embryos’ to refer to the fertilized, developing brood carried on the female (Bauer 2023). These correspond to the terms ‘egg’, ‘egg size’, and ‘clutch size’ used in earlier taxonomic literature for the genus (e.g., Holthuis 1965; Cai et al. 2007; von Rintelen and Cai 2009; Richard and Clark 2014; de Mazancourt et al. 2020; Klotz et al. 2021, 2024). The number of embryos was determined by observing 5–10 ovigerous females per species. To reduce the variation in size due to embryogenesis (Hancock et al. 1998; Anger et al. 2002), embryo measurements were derived from a sample of 50 embryos with eyes per species by recording both length (**L**) and width (**W**). Embryos were treated as an ellipsoid (prolate spheroid) and the embryo volume was calculated using the formula:

$V=\frac{\pi }{6}\times L\times W^{2}$

where *L* represents the length of the long axis and *W* represents the length of the short axis (Hancock et al. 1998; Nazari et al. 2003). The differences in embryo volume and the number of embryos with respect to species were examined using analysis of variance (ANOVA) and followed by a post-hoc Tukey-HSD test.

Abbreviations of morphological characters are as follows: **cl**, carapace length (mm); **P1**, first pereopod; **P2**, second pereopod; **P3**, third pereopod; **P5**, fifth pereopod; **Pl1**, first male pleopod; and **Pl2**, second male pleopod.

### DNA extraction and PCR methodology

Genomic DNA was extracted from abdominal muscle tissue using a NucleoSpin tissue kit (MACHEREY-NAGEL, Germany). Fragments of two mitochondrial genes, ~590 bp of the 16S ribosomal ribonucleic acid (16s rRNA) and 861 bp of the cytochrome c oxidase subunit I (COI) genes, were amplified using the primer sets 16Sar-Lmod (5’–AAA AAC TAT TTG TCC GTC TTC AT–3’) / 16Sbmod (5’–GGT CTG AAC TCA AAT CAT GTA AA–3’) (de Mazancourt et al. 2019a) for 16S rRNA and COI–F–Car (5’–GCT GCT AAT TTT ATA TCT ACA G–3’) / COI–R–Car (5’–TGT GTA GGC ATC TGG GTA ATC–3’) for COI (von Rintelen et al. 2007). DNA amplification was performed with the following conditions: 5 min at 94 °C; 30 cycles of denaturation for 60 s at 94 °C, annealing for 45 s at 46–50 °C, and elongation for 90 s at 72 °C, and post-elongation for 4 min at 72 °C. The final total PCR volume was 30 μl, consisting of 15 μl of EmeraldAmp PCR Master Mix (TAKARA BIO INC.), 1.5 μl of each 10 µM forward and reverse primers, 9 μl of distilled water, and 3 μl of at least 10-ng template DNA. Amplified PCR products were purified before being sequenced in both directions by an automated sequencer (ABI prism 3730XL). Contigs of forward and reverse strands were assembled using the MUSCLE algorithm as implemented in MEGA11 v. 11.0.13 (Tamura et al. 2021) before being checked and corrected manually. The newly obtained nucleotide sequences in this study were deposited in the GenBank database under the accession numbers provided in Suppl. material 1: table SS1 as well as specimen vouchers.

### Phylogenetic analyses

In total, the dataset for phylogenetic analysis comprised 208 sequences from 46 *Caridina* species and seven outgroup sequences. This dataset included 131 newly sequenced individuals in the present study and 77 sequences retrieved from GenBank. The outgroup included seven sequences of the atyid genera *Atya* Leach, 1816, *Atyopsis* Chace, 1983, and *Atyoida* Randall, 1840, following Klotz et al. (2024). We also included two sequences of *C. thai* collected from the type locality in central Thailand into the dataset. Sequences were aligned using MAFFT v. 7.49 (Katoh and Standley 2013) with the L-INS-i setting for both 16S rRNA and COI genes.

Maximum Likelihood (ML) phylogenetic trees were reconstructed using IQ-Tree v. 2.1.2 (Nguyen et al. 2015). Models of sequence evolution were estimated using ModelFinder (Kalyaanamoorthy et al. 2017) as implemented in IQ-Tree. Codon partitioning and selected models are summarized in Table 1. Support for clades was assessed with 10,000 ultrafast bootstrap replications (Hoang et al. 2018). Bayesian inference (BI) analyses were conducted with MrBayes v. 3.2.6 (Ronquist et al. 2012). The best-fit models of nucleotide substitution for each gene were estimated using jModeltest v. 2.1.10 (Darriba et al. 2012) using the Bayesian Information Criterion. Metropolis-coupled Markov chain Monte Carlo (MCMC) was run for 10,000,000 generations with trees sampled every 2,000 generations. The first 25% were discarded as burn-in. Tracer v. 1.6 was used to check that all parameters had reached stationary conditions (defined as standard deviation > 0.01, effective sampling size (ESS) ≥ 200 for all parameters, and the potential scale reduction factor (PSRF) reaching 1.0) after 10% burn-in (Rambaut et al. 2018). Both ML and BI analyses were run on the online CIPRES Science Gateway server (Miller et al. 2010). Only clades with ML bootstrap values of ≥ 80 and Bayesian posterior probability of ≥ 0.95 were considered as significantly supported in this study (San Mauro and Agorreta 2010; Hoang et al. 2018). To prevent the formation of polyphyletic morphological groups, the species groups proposed in this study are strictly defined by supported monophyletic clades, accompanied by congruent morphological characters.

**Table 1.** Best substitution models for each partition in molecular analyses.

**Molecular marker**	**Codon position**	**Substitution models**	
		**IQ–TREE**	**MrBayes**
16S	–	TPM3+F+G4	HKY + I + G
COI	1	TNe+FQ+G4	SYM + G
COI	2	TPM3+F+I	HKY + I
COI	3	TIM2+F+G4	GTR + G

## Results

**Figure 2.**
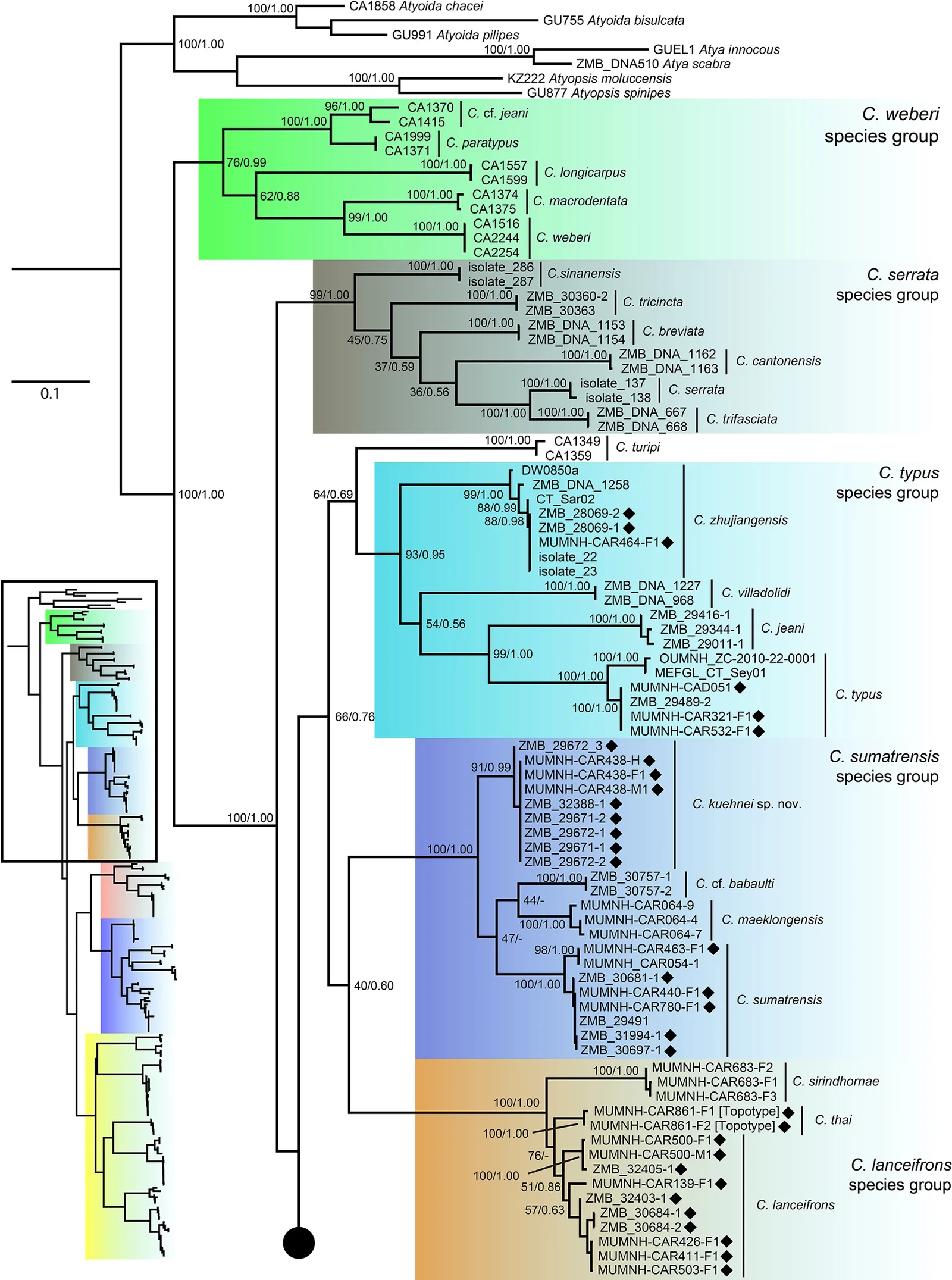
Part of Maximum likelihood tree reconstructed from 16s rRNA and COI genes, showing phylogenetic relationship in *Caridina weberi*, *C. serrata*, *C. typus*, *C. sumatrensis*, and *C. lanceifrons* species groups. Sequences newly obtained from the current study are labeled with black diamonds. Nodal supports are shown as ML bootstrap value/Bayesian posterior probability.

**Figure 3.**
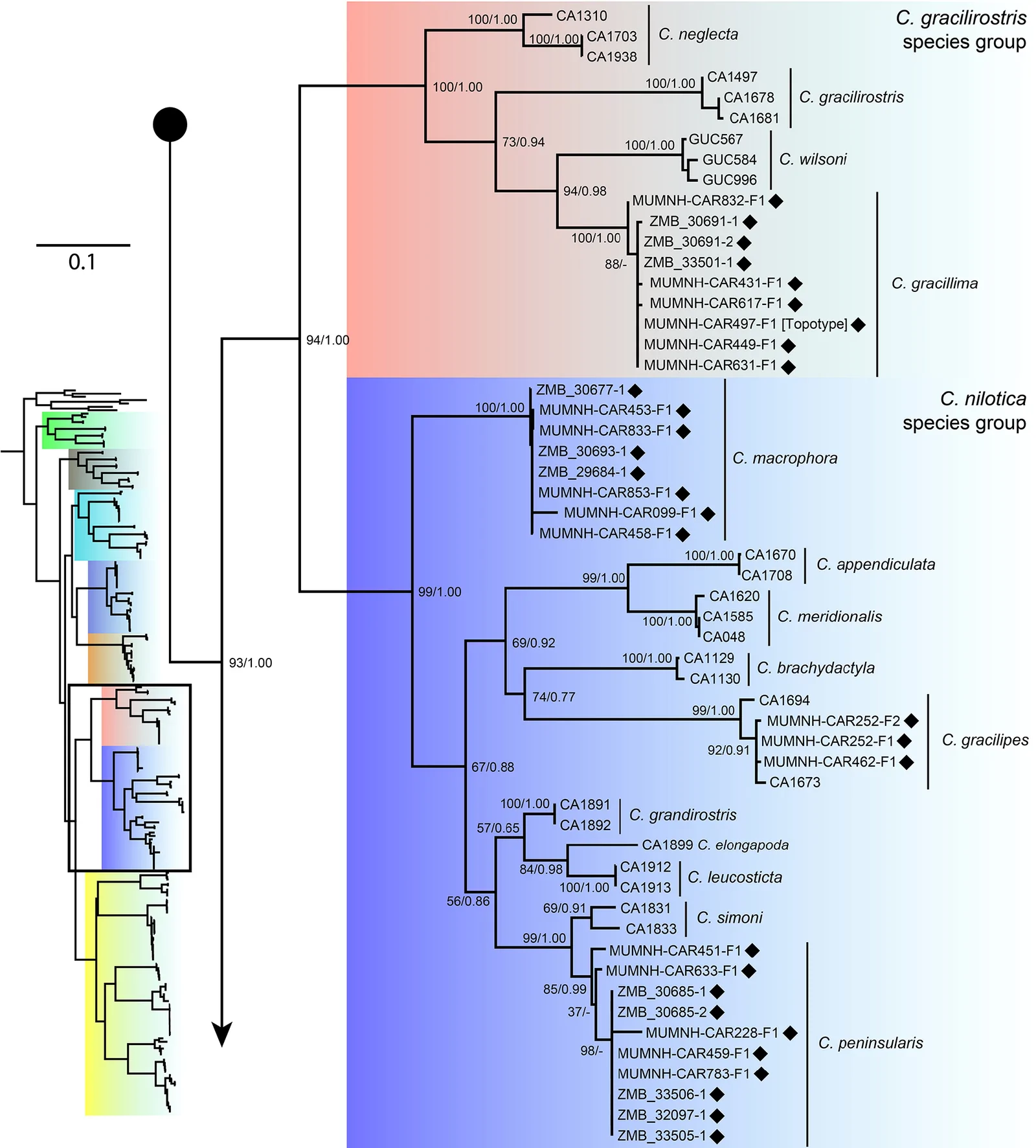
Part of the Maximum likelihood tree reconstructed from 16s rRNA and COI genes, showing phylogenetic relationship in *Caridina gracilirostris* and *C. nilotica* species groups. Sequences received from the current study are labeled with black diamonds. Nodal supports are shown as ML bootstrap value/Bayesian posterior probability.

**Figure 4.**
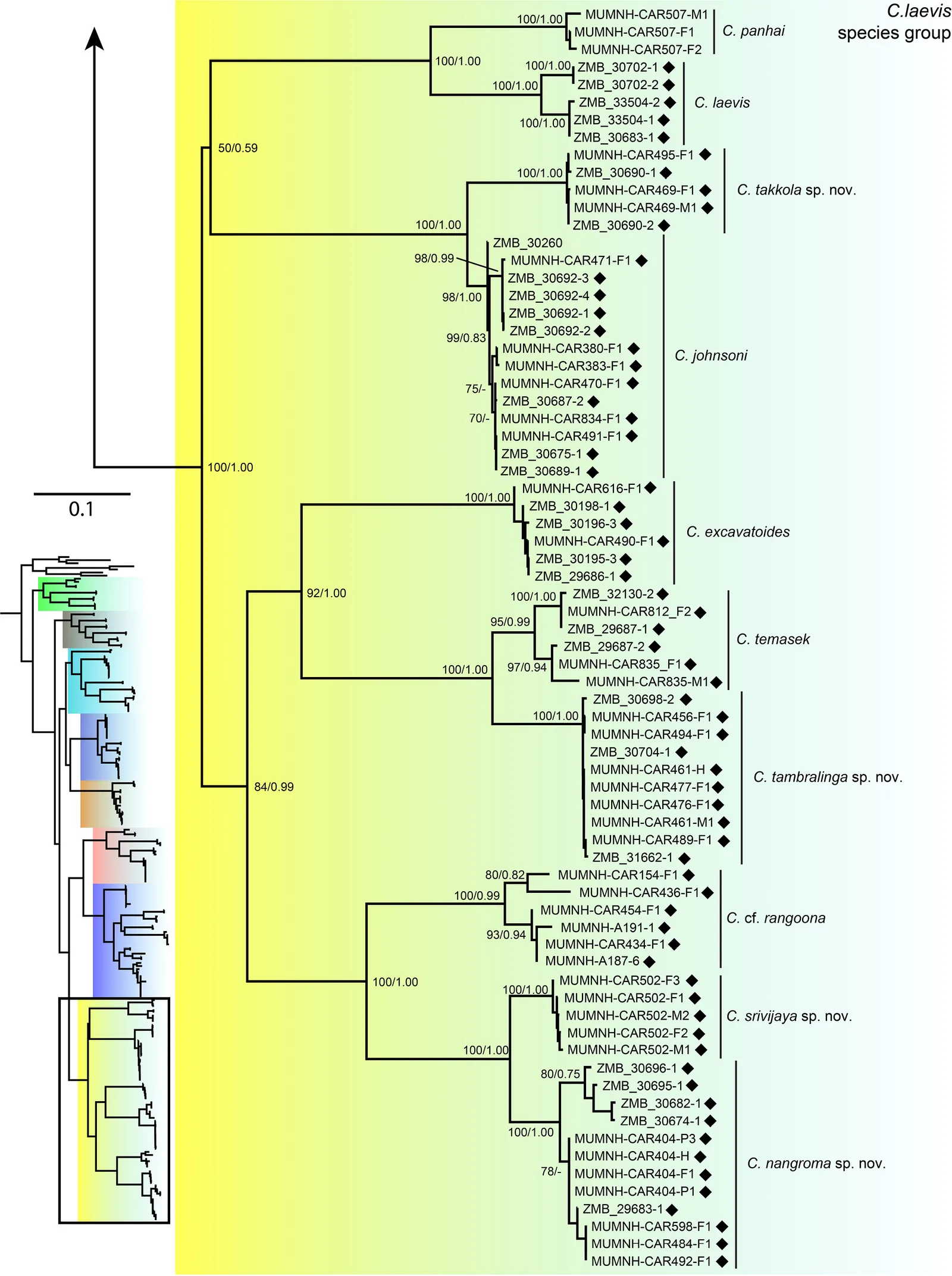
Part of the Maximum likelihood tree reconstructed from 16s rRNA and COI genes, showing phylogenetic relationship in *Caridina laevis* species group. Sequences newly obtained from the current study are labeled with black diamonds. Nodal supports are shown as ML bootstrap value/Bayesian posterior probability.

Phylogenetic trees from ML and BI analyses revealed a total of 18 distinct species-level clades of southern Thailand *Caridina* with high nodal support (BS = 85–100%, BPP = 0.99–1.00; Figs 2, 3, 4), except for *C. lanceifrons* with only moderate support (BS = 51%, BPP = 0.86; Fig. 2). These clades correspond to 13 described and valid species and five unknown new species (Figs 2, 3, 4). Each of the undescribed species displays morphological distinctiveness from its congeners; consequently, they are described herein as new species, *C. kuehnei* sp. nov., *C. nangroma* sp. nov., *C. srivijaya* sp. nov., *C. takkola* sp. nov., and *C. tambralinga* sp. nov. *Caridina thai* collected from the type locality was also recovered with fully nodal supports (BS = 100%, BPP = 1.00; Fig. 2).

**Figure 5.**
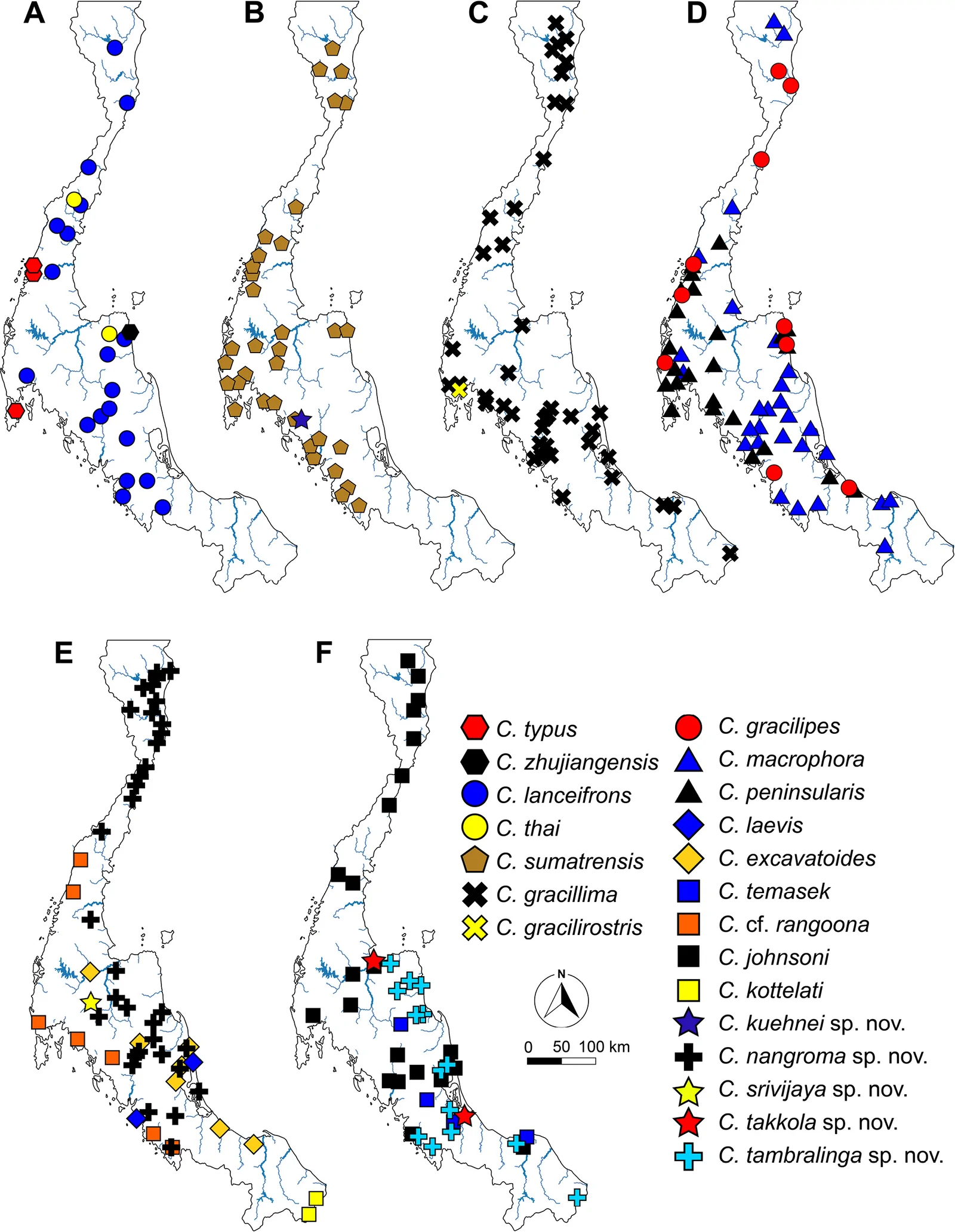
Distribution map of *Caridina* in southern Thailand. **A**. *C. typus*, *C. zhujiangensis*, *C. lanceifrons*, and *C. thai*; **B**. *C. sumatrensis* and *C. kuehnei* sp. nov.; **C**. *C. gracillima* and *C. gracilirostris*; **D**. *C. gracilipes*, *C. macrophora*, and *C. peninsularis*; **E**. *C. laevis*, *C. excavatoides*, *C. cf. rangoona*, *C. kottelati*, *C. nangroma* sp. nov., and *C. srivijaya* sp. nov.; **F**. *C. temasek*, *C. johnsoni*, *C. takkola* sp. nov., and *C. tambralinga* sp. nov. Distribution ranges for all species were obtained from the current study, except for those of *C. gracilirostris*, *C. thai*, and *C. kottelati* whose distributions were obtained from Cai and Naiyanetr (2024).

All proposed species groups were also largely recovered as monophyletic groups with high nodal supports (BS = 76–100%, BPP = 0.95–1.00; Figs 2, 3, 4). When combined with previously recorded species that were not found in this study, *Caridina* diversity in southern Thailand totals 21 species. A distribution map of all species in southern Thailand is shown in Fig. 5. The taxonomic information for each taxon is provided in the taxonomic section below.

**Figure 6.**
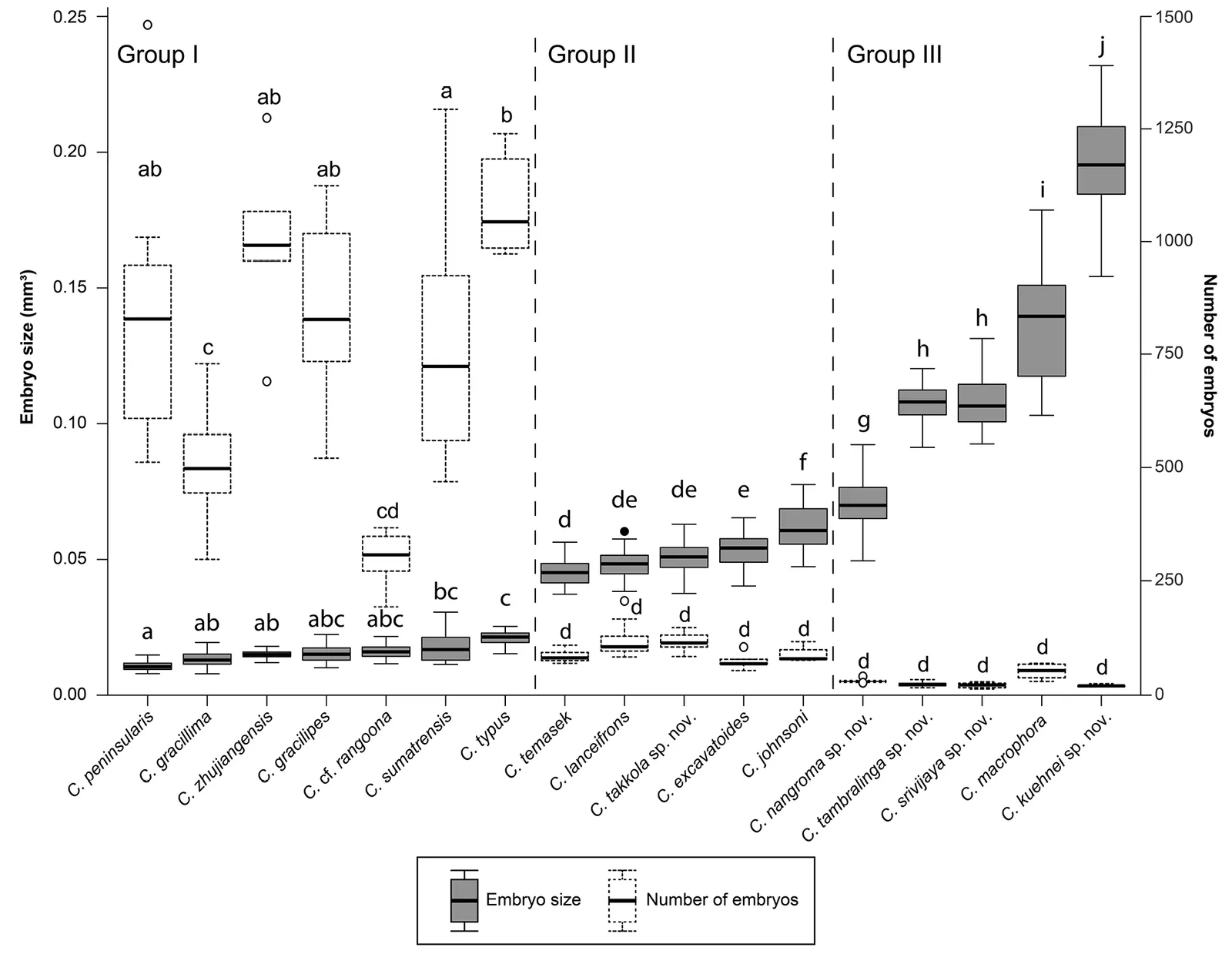
Bivariate box plots illustrating embryo size (left Y-axis) and the number of embryos (right Y-axis) for *Caridina* species in southern Thailand. The letters positioned above the data points indicate the results of post-hoc statistical testing (ANOVA followed by Tukey’s HSD test, p < 0.05), with groups sharing the same letter not being significantly different.

Embryo size of *Caridina* from southern Thailand showed substantial interspecific variability, statistically grouping the species into three distinct major categories (ANOVA, F (16, 833) = 1865, p < 0.05, and post-hoc pairwise analysis, p < 0.05; Fig. 6, Suppl. material 1: table S2). Group I included *C. gracilipes* De Man, 1892, *C. gracillima*, *C. peninsularis*, *C. zhujiangensis* Chen, Chen & Guo, 2018, *C. cf. rangoona* Cai & Ng, 2000, *C. sumatrensis*, and *C. typus*. This group is characterized by the production of numerous small-sized embryos (0.35–0.52 × 0.20–0.34 mm). Group II included *C. excavatoides* Johnson, 1961, *C. johnsoni*, *C. lanceifrons* Yu, 1936, *C. takkola* sp. nov., and *C. temasek* Choy & Ng, 1991. Species in this group spawn an intermediate number of medium-sized embryos (0.57–0.73 × 0.34–0.45 mm). Finally, Group III is comprised of *C. kuehnei* sp. nov. *C. macrophora*, *C. nangroma* sp. nov., *C. srivijaya* sp. nov., and *C. tambralinga* sp. nov., with a small number of large-sized embryos (0.67–1.07 × 0.35–0.67 mm). When comparing the number of embryos, there was a significant difference among the examined *Caridina* species (ANOVA, F (16, 101) = 50.97, p < 0.05, Suppl. material 1: table S3). However, the post-hoc analysis only indicated a significantly higher number of embryos in Group I (195–1483 embryos per individual; mean 758 embryos; median 748 embryos) compared to Group II and Group III. A trend towards a moderate number of embryos was observed in Group II (54–208 embryos per individual; mean 101 embryos; median 97 embryos), while Group III tended to have the lowest number of embryos (13–70 embryos per individual; mean 33 embryos; median 29 embryos), although these differences were not significant. Overall, the data demonstrated a clear inverse trend between embryo size and the number of embryos across the examined species, where increasing individual embryo size is associated with a reduction in the total number of embryos.

### Taxonomic account

#### Family ATYIDAE De Haan, 1849

##### Genus Caridina

Taxon classificationAnimaliaDecapodaAtyidae

H. Milne Edwards, 1837

0CC5256A-6565-5868-AB70-513F5A5673F2

###### Type species.

*Caridina typus* H. Milne Edwards, 1837

#### *Caridina typus* species group

**Diagnosis**. Rostrum quite straight, unarmed dorsally, without subapical teeth. Antennular peduncle blunt, ~ 0.4–0.7× as long as carapace length. Walking legs stout. P3 and P5 dactylus stout. Epipods well-developed on P1–P4. Sixth abdominal somite ~ 0.6× as long as cl. Plumose setae on distal end of telson subequal in length. Uropodal diaeresis with > 15 moveable spiniform setae. Median plumose setae on distal end of telson slightly longer than lateral plumose setae or subequal in length. Preanal carina high, subtriangular, without a spine. Endopod of male first pleopod long, subtriangular, with an appendix interna, emerging from distal 1/3.

**Remarks**. The *Caridina typus* species group was first proposed by Bouvier (1905), with the shared characteristic of a dorsally unarmed rostrum. Subsequently, Bouvier (1925) suggested more diagnostic characters of the *C. typus* species group, including (1) uropodal diaeresis with > 20 moveable spiniform setae, (2) antennular peduncle < 1/2 of cl, and (3) sixth abdominal somite < 1/2 of cl.

De Mazancourt et al. (2020) included *C. turipi* de Mazancourt, Boseto, Marquet & Keith, 2020 in the *C. typus* species group because this species shared several characters with *C. typus*. Our phylogenetic tree also showed a sister relationship between *C. turipi* and the other species in *C. typus* species group, but with considerable genetic distance and low nodal support (BS = 64%, BPP = 0.69; Fig. 2). Morphologically, *C. turipi* possesses dorsal teeth on its rostrum, whereas all other species within the *C. typus* group are characterized by a dorsally unarmed rostrum. Therefore, we propose to exclude *C. turipi* from the *C. typus* species group in this study and retain the unarmed rostrum as a shared character within this species group. Further investigation is required to definitively resolve the taxonomic position of *C. turipi*.

The present phylogenetic tree recovered four species in the *C. typus* species group as well-supported monophyletic lineage (BS = 99–100%, BPP = 1.00; Fig. 2). *Caridina zhujiangensis* was recovered as basal clade of the species group and as sister to a clade of *C. villadolidi* Blanco, 1939. The latter species was synonymized with *C. siamensis* Giebel, 1863 by Cai and Naiyanetr (2024), although this synonymy awaits confirmation because it was based entirely on the text description of *C. siamensis*, while the original type specimen is currently lost (H. Müller, pers. comm. to T. von Rintelen, July 2026). Furthermore, based on current records, the distribution of *C. villadolidi* is geographically restricted exclusively to Indonesia and the Philippines. The remaining species of this group are *C. jeani* Cai, 2010, and *C. typus*. In southern Thailand, only two species have been recorded to date: *Caridina typus* and *C. zhujiangensis* (Cai and Naiyanetr 2024; this study).

##### Caridina typus

Taxon classificationAnimaliaDecapodaAtyidae

H. Milne Edwards, 1837

4BF1F3AB-703F-52D2-84CA-80FB5BF2AC7C

Figs 5A, 7, 8A

Caridina typus H. Milne Edwards, 1837: 363, pl. 25, figs 4, 5. Type locality not specified. It was suspected to Mauritius Island, the Indian Ocean by later authors (see Bouvier 1925 and de Mazancourt et al. 2020).
Caridina typus — Miers 1879: 492; Richters 1880: 162, pl. 17, fig. 23; Pfeffer 1889: 35; De Man 1892: 367, pl. 21, fig. 22; Sharp 1893: 111; Ortmann 1894a: 8; Ortmann 1894b: 403; Weber 1897: 167 (in part); Hilgendorf 1898: 34; Nobili 1899: 232; Doflein 1900: 127; Roux 1904: 552; Lenz 1905: 385; Lenz 1910: 570; Bouvier 1904: 134; Borradaile 1907: 67; Edmondson 1935: 10, fig. 3g–l; Kubo 1938: 83, fig. 13; Miyake 1938: 111, fig. 1; Kamita 1959: 21; Johnson 1960: 180; Johnson 1963: 27; Kamita 1963: 5, fig. 5; Holthuis 1965: 10, fig. 3; Johnson 1966: 418; Johnson 1969: 110; Holthuis 1969: 93; Ooishi 1970: 87; Tiwari and Pillai 1971: 86; Suzuki 1972: 10, figs 8–10; Fujino 1972: 7, fig. 8; Shokita 1975: 119; Shokita and Nishijima 1977: 187; Holthuis 1978: 30; Shokita 1979: 204; Shokita 1981: 15; Ng 1985: 161; Holthuis 1986: 590; Maciolek and Ford 1987: 628; Coetzee 1988: 62; Bright 1989: 34; Hayashi 1989: 310, fig. 168; Choy 1991: 356; Hung et al. 1993: 486, figs 1c, 4a–f; Suzuki et al. 1993: 56; Suzuki and Sato 1994: 60; Ng 1995: 189, fig. 7; Hamano and Honke 1997: 162; Chace 1997: 21; Choy and Marshall 1997: 26; Yeo et al. 1999: 225; Ebenezer and Richard 1999: 427, figs 1–3; Keith et al. 1999: 46; Ideguchi et al. 2000: 5; Short 2000: 61; Buden et al. 2001: 261; Leberer and Nelson 2001: 393; Day et al. 2001: 96, fig. 6.3; Choy and Marquet 2002: 219; Marquet et al. 2002: 222; Shokita et al. 2002: 76; Marquet et al. 2003: 68; Keith 2002: 100; Leberer and Cai 2003: 354; Shokita et al. 2003: 117; Cai and Anker 2004: 236, fig. 9g; Cai et al. 2006: 412, figs 13–15; Cai and Shokita 2006a: 2134; Keith et al. 2006: 62; Cai et al. 2007: 279; Cai and Ng 2009: 1094; Soomro et al. 2010: 721; Keith et al. 2010: 46; Soomro et al. 2011: 41; Saito et al. 2012: 27; Cook et al. 2012: 289, fig. 3; Soomro et al. 2013: 49; Bouchard et al. 2013: 9; Emmerson 2016: 162, 173; Fujita et al. 2016: 105; Soomro et al. 2016: 569; Soomro et al. 2020: 1333; Bernardes et al. 2017: 1 [in part; ARC clade]; de Mazancourt et al. 2017b: 226, fig. 4; de Mazancourt et al. 2019a: 167; Choy et al. 2019: 115; Rahayu and Annawaty 2019: 64; Klotz et al. 2019: 164, fig. 18; de Mazancourt et al. 2020: 34–37, figs 2d, 10; Imai et al. 2020: 63, fig. 3a; Vijayamma et al. 2021: 409; Cai and Naiyanetr 2024: 770; de Mazancourt et al. 2024: 17, fig. 3e; de Mazancourt et al. 2025: 66, figs 2g, 5f.
Caridina *typa* [sic] — Bouvier 1905: 88; Bouvier 1913: 465; Bouvier 1914: 699; Bouvier 1925: 249, figs 271–297; Roux 1926a: 246; Roux 1926b: 201; Riek 1953: 117; Choy and Horwitz 1995: 52.
Caridina exilirostris Stimpson, 1860: 29. Type locality “insulam Loo-choo” [= Okinawa, Ryukyu Island, Japan].
Caridina typus f. typica Bouvier, 1925: 250, figs 272–295. Type locality “Ile Maurice” [= Mauritius], La Réunion, Madagascar, Saleyer, “Celebes” [= Sulawesi], “Japonais” [= Japan], “iles Loo-choo” [= Okinawa, Ryukyu Island, Japan].
Caridina typus f. caledonica Bouvier, 1925: 253, figs 296–297. Type locality “Nouvelle Caledonie” [= New Caledonia].

###### Material examined.

Five lots (see Suppl. material 1: Data S1).

###### Description.

**Figure 7.**
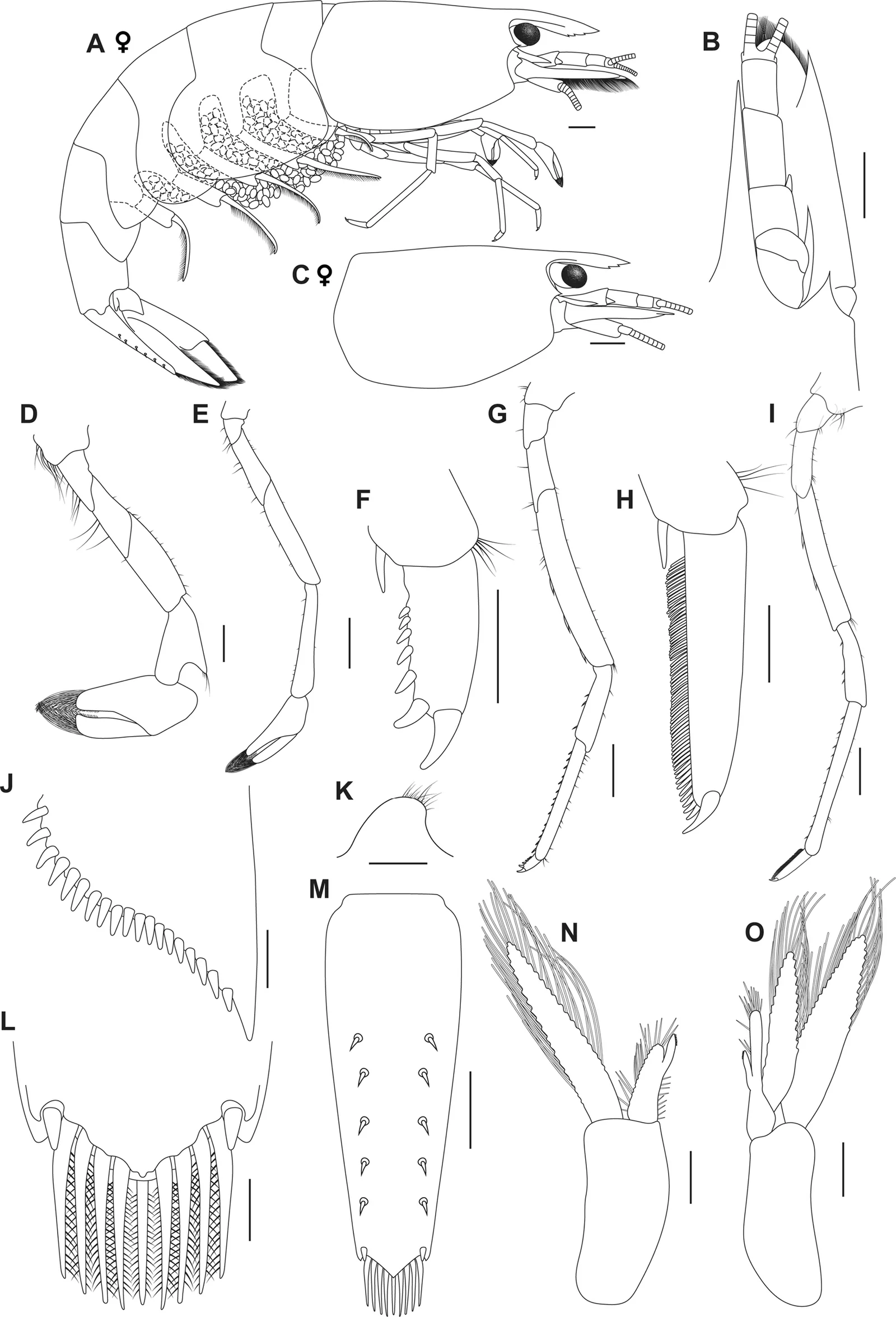
*Caridina typus*. **A**. Ovigerous female; **B**. Top view of carapace; **C**. Cephalothorax and cephalic appendages of female; **D**. First pereopod; **E**. Second pereopod; **F**. Dactylus of third pereopod; **G**. Third pereopod; **H**. Dactylus of fifth pereopod; **I**. Fifth pereopod; **J**. Uropodal diaeresis; **K**. Preanal carina; **L**. Distal end of telson; **M**. Telson; **N**. First pleopod; **O**. Second pleopod. Drawings were made of MUMNH-CAR532-D1 (**A**, **B**, **D**–**M**); MUMNH-CAR093-D1 (**C**); MUMNH-CAR532-M1 (**N**, **O**). Scale bars: 1 mm (**A, B, C, G, I, M**); 0.5 mm (**D, E, N, O**); 0.25 mm (**F, H, J–L**).

***Cephalothorax and cephalic appendage*** (*n* = 5). cl 5.31–6.79 (median 5.84; Fig. 7A–C) mm, width 4.06–4.80 (median 4.61) mm. Rostrum unarmed dorsally (Fig. 7A–C), straight or slightly curved down, reaching near the end of third segment of antennular peduncle (Fig. 7B), 0.38–0.53 (median 0.47) as long as cl. Rostral formula: (0) 0 / 0–4. Antennal spine indistinctively fused with orbital margin. Pterygostomian margin round. Eye well developed, anterior end reaching to 0.42–0.63 (median 0.49) of first segment of antennular peduncle. Antennular peduncle 0.42–0.60 (median 0.56; Fig. 7A–C) as long as cl, first segment 1.25–1.73 (median 1.59) × as long as second segment, second segment 1.07–1.63 (median 1.37) × as long as third segment. Tooth on distolateral margin of first segment of antennular peduncle well-developed. Stylocerite reaching to 0.81–0.95 (median 0.86) of first segment of antennular peduncle. Scaphocerite 2.57–2.97 (median 2.62) × as long as wide, distal margin with short plumose setae.

***Branchial and mouthparts***. Podobranch on second maxilliped well developed. Third maxilliped possessed one small and one large arthrobranch. Pleurobranchs present on all pereopods. Third maxilliped reaching to end of second segment of antennular peduncle; ultimate segment of endopod ending with one large claw and three to six spiniform setae on distal 1/3 of posterior margin, 0.74–0.88 (median 0.78, *n* = 5) as long as penultimate segment.

***Pereopods*** (*n* = 5). Epipod well developed on first four pereopods. Chelae of P1 and P2 well-developed.

P1 short (Fig. 7D); chela 2.02–2.11 (median 2.07) × as long as wide, 1.33–1.46 (median 1.39) × as long as carpus, tips of fingers rounded, with tuft of setae near tip; dactylus 0.86–1.42 (median 1.17) as long as palm; carpus very stout, excavated distally, 1.33–1.70 (median 1.53) × as long as wide, 0.89–1.28 (median 1.00) × as long as merus; merus 1.92–2.70 (median 2.54) × as long as wide, 1.17–1.75 (median 1.41) × as long as ischium.

P2 slenderer, longer than P1 (Fig. 7E); chela 2.80–3.14 (median 2.92) × as long as wide, 0.72–0.76 (median 0.75) as long as carpus, tips of fingers rounded, with tuft of setae near tip; dactylus 1.32–1.89 (median 1.55) × as long as palm; carpus slender, 4.69–6.85 (median 6.10) × as long as wide, 0.98–1.28 (median 1.10) × as long as merus; merus 4.74–6.07 (median 5.51) × as long as wide, 1.27–1.65 (median 1.53) × as long as ischium.

P3 not sexually dimorphic (Fig. 7G); dactylus with 5–7 (mode 6) spiniform setae on flexor margin (Fig. 7F), 2.46–2.82 (median 2.61) × as long as wide (including terminal claw), terminating with one large claw; propodus bearing numerous spiniform setae on posterior margin, 8.86–10.37 (median 9.81) × as long as wide, 4.40–5.35 (median 5.01) × as long as dactylus; carpus possessed four setae on posterior margin of outer surface, the distal seta largest, the other setae minute, 4.29–5.44 (median 4.86) × as long as wide, 0.63–0.74 (median 0.68) as long as propodus; merus possessed four large setae on posterior margin of outer surface, 6.51–7.24 (median 6.71) × as long as wide, 1.90–2.30 (median 2.23) × as long as carpus; ischium with or without one spiniform seta.

P5 slender (Fig. 7I); Dactylus with 65–72 spiniform setae on flexor margin (Fig. 7H), 3.58–4.69 (median 4.47) × as long as wide (including terminal claw), terminating with one large claw; propodus bearing numerous spiniform setae on posterior margin, 11.39–13.55 (median 12.81) × as long as wide, 3.28–3.62 (median 3.34) × as long as dactylus; carpus possessed three setae on posterior margin of outer surface, the distal seta largest, the other setae minute, 4.47–4.91 (median 4.49) × as long as wide, 0.51–0.53 (median 0.52) as long as propodus; merus possessed two or three large setae on posterior margin of outer surface, 6.29–6.95 (median 6.56) × as long as wide, 1.52–1.71 (median 1.59) × as long as carpus; ischium without spiniform seta.

***Male pleopods*** (*n* = 7). Pl1 endopod subtriangular (Fig. 7N), wider proximally, 2.45–3.34 (median 3.09) × as long as wide, 0.34–0.41 (median 0.38) of exopod length, with appendix interna, emerging from distal 1/3 of endopod. Pl2 appendix masculina rod-shaped (Fig. 7O), slender with many setae, 0.63–0.77 (median 0.65) as long as endopod; appendix interna reaching 0.61–0.71 (median 0.67) of appendix masculina length.

***Abdomen*** (*n* = 5). Sixth abdominal somite ~ 0.46–0.56 (median 0.53) of cl, 1.24–1.46 (median 1.37) × as long as fifth somite, 0.82–0.89 (median 0.88) as long as telson (Fig. 7A). Telson 2.51–2.87 (median 2.76; Fig. 7M) × as long as wide, with five or six pairs (mode 5) of dorsal spiniform setae and one pair of dorsolateral spiniform setae; distal margin subtriangular with posteromedian projection, 6–9 moveable plumose setae, median plumose setae slightly longer than lateral setae (Fig. 7L). Preanal carina high, subtriangular, slightly skewed backward, with a few setae, without a spine (Fig. 7K). Uropodal diaeresis with 19–21 moveable spiniform setae (Fig. 7J).

***Embryos***. Ovigerous females with 976–1,242 embryos (mean 1,088, *n* = 5). Size of embryos with eyes 0.42–0.52 × 0.26–0.31 mm (*n* = 50).

###### Coloration.

**Figure 8.**
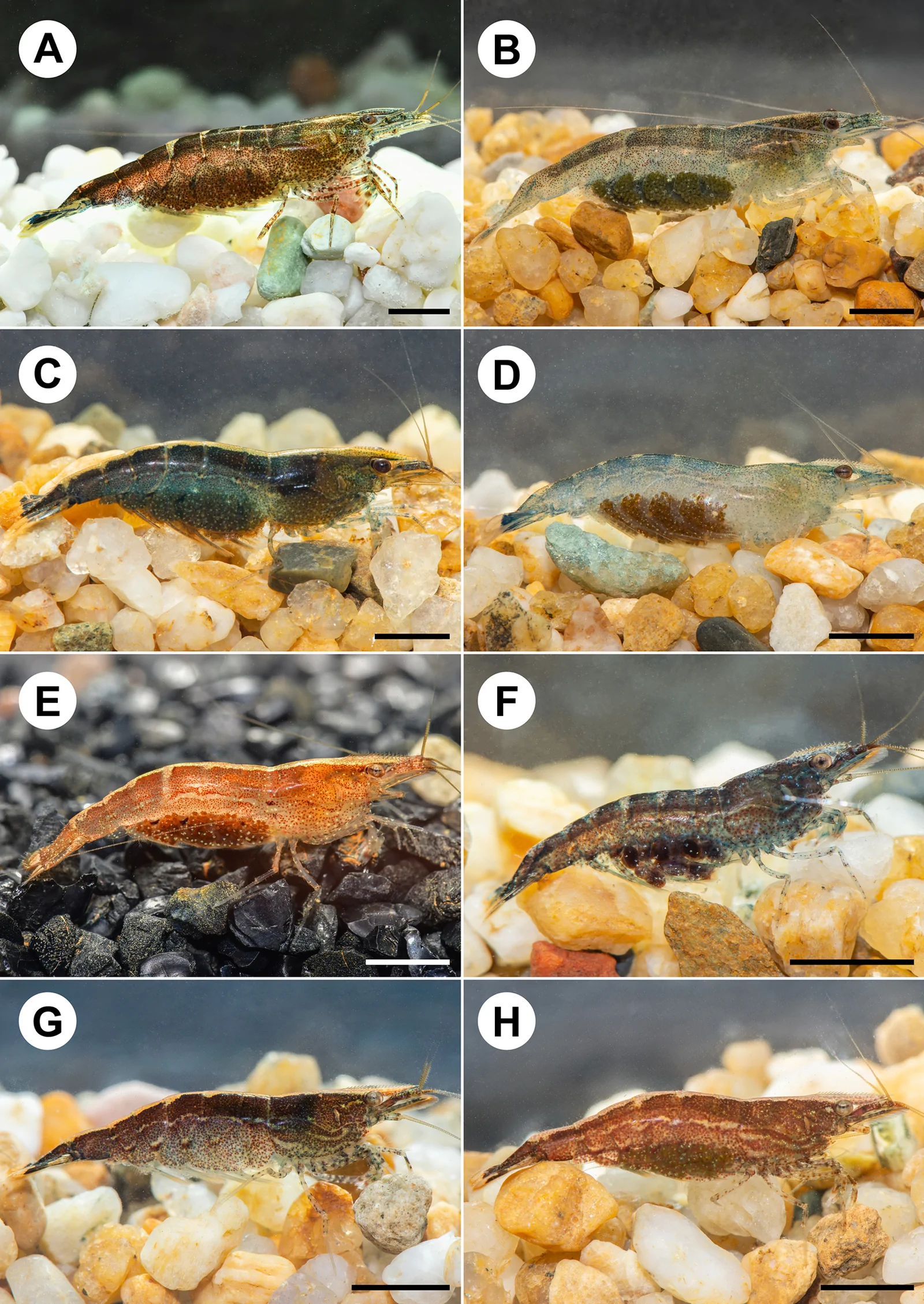
Living specimens of *Caridina* species. **A**. *C. typus*, specimen MUMNH-CAR532-P1 from Phuket Province, Thalang District, Bang Prae Stream; **B**. *C. zhujiangensis*, specimen MUMNH-CAR464-P1 from Nakhon Si Thammarat Province, Khanom District, Khao Phlai Dam; **C–E**. *C. sumatrensis*, specimens; **C**. MUMNH-CAR430-P1 from Phuket Province, Thalang District, Bang Prae Stream; **D**. MUMNH-CAR452-P1 from Satun Province, La-ngu District, La-ngu Stream; **E**. MUMNH-CAR219-P1 from Ranong Province, Mueang District, Ngao Noi Stream; **F**. *C. kuehnei* sp. nov., paratype MUMNH-CAR438-P1 from its type locality; **G, H**. *C. lanceifrons*, specimens; **G**. MUMNH-CAR411-P1 from Prachuap Khiri Khan Province, Bang Saphan District, Sai Khu Waterfall; **H**. MUMNH-CAR426-F1 from Chumphon Province, Mueang District, Hin Wang Stream. Scale bar: 4 mm.

Body orangish yellow to brown, furnished with numerous small dark spots. Rostrum pale brown. Cephalic region and dorsal abdominal somite darker than the other parts. Lower abdominal somite is thistle green. Each abdominal somite dorsally decorated with conspicuous light transverse bands. Embryos brown to dark brown (Fig. 8A).

###### Distribution and habitat.

*Caridina typus* is widely distributed from the eastern coast of Africa (mainland South Africa, Madagascar, and Seychelles) to Melanesia (Papua New Guinea, Solomon Islands, New Caledonia, Vanuatu, and Fiji), including Japan and the Philippines (Bernardes et al. 2017; de Mazancourt et al. 2020, 2024). In southern Thailand, *C. typus* can be found at the western coast of the peninsula (Andaman Sea; Fig. 5A). This species inhabits estuaries and lowland rivers near the coastal area, where the bottom substrate is gravel and sand. It is found on the water bottom, aquatic vegetation along the banks, or under rocks or leaf litter.

###### Phylogenetic placement.

The molecular analyses indicate that *C. typus* from Thailand is monophyletic (BS = 100%, BPP = 1.00; Fig. 2) and nested with individuals from Pacific and Western Indian Ocean regions. It was placed as a sister to *C. jeani* (BS = 99%, BPP = 1.00).

###### Remarks.

Bernardes et al. (2017) investigated the intraspecific phylogeny of *C. typus* by molecular phylogenetic analyses. Their findings indicated that the morphospecies ‘*C. typus*’ was paraphyletic, being clustered in four species-level clades. One clade corresponded to *C. villadolidi sensu*Bernardes et al. (2017) and three others were represented as the *C. typus* s.l. complex. Later, Cai (2025) and de Mazancourt et al. (2025) demonstrated that these three lineages correspond to *C. jeani*, *C. typus* s.s., and *C. zhujiangensis*.

In the present study, our specimens grouped with individuals from the *C. typus* s.s. clade, which comprises individuals from a broad geographic distribution extending from the Indian Ocean (Seychelles and Mascarene islands) to the Pacific Ocean (Langkawi, Philippines, Australia, and Melanesia) (Bernardes et al. 2017). This is congruent with the geographic distribution of *C. typus* in southern Thailand, which is found only at the western coast of the peninsula (Andaman Sea coast; Fig. 5A).

Our *C. typus* specimens from southern Thailand show a similar morphology to previous reports, including the lack of dorsal teeth, but with 0–4 teeth on ventral margin (De Man 1892; Holthuis 1965; Tiwari and Pillai 1971; Ebenezer and Richard 1999; Cai and Anker 2004; Cai et al. 2006; de Mazancourt et al. 2020), rostrum length (0.38–0.53 as long as cl vs 0.3–0.6; de Mazancourt et al. 2020), P1 chela (2.02–2.11× as long as wide vs 1.9–2.7; Tiwari and Pillai 1971; Cai et al. 2006; de Mazancourt et al. 2020), P1 dactylus (0.86–1.42× as long as palm vs 0.6–1.3; Tiwari and Pillai 1971; de Mazancourt et al. 2020), P1 carpus (1.33–1.70× as long as wide vs 1.2–2.2; Tiwari and Pillai 1971; Ebenezer and Richard 1999; Cai et al. 2006; de Mazancourt et al. 2020), P2 chela (2.80–3.14× as long as wide vs 2.4–3.3; Tiwari and Pillai 1971; Cai et al. 2006; de Mazancourt et al. 2020), P2 dactylus (1.32–1.89× as long as palm vs 1.3–1.8; Tiwari and Pillai 1971; Cai et al. 2006; de Mazancourt et al. 2020), P2 carpus (4.69–6.85× as long as wide vs 5.0–6.5; Cai et al. 2006; de Mazancourt et al. 2020), spiniform setae on P3 dactylus (5–7 vs 4–7; Holthuis 1965; Tiwari and Pillai 1971; Ebenezer and Richard 1999; de Mazancourt et al. 2020; Cai et al. 2006), P3 dactylus (2.46–2.82× as long as wide vs 2.4–3.4; Cai et al. 2006; de Mazancourt et al. 2020), spiniform setae on P5 dactylus (65–72 vs 33–81; Cai et al. 2006; de Mazancourt et al. 2020), P5 dactylus (3.58–4.69× as long as wide vs 3.2–5.1; Ebenezer and Richard 1999; Cai et al. 2006; de Mazancourt et al. 2020), the sixth abdominal somite (0.43–0.52 as long as cl vs 0.43–0.48; de Mazancourt et al. 2020), dorsal spiniform setae on telson (5–6 pairs vs 4–6 pairs; Holthuis 1965; Tiwari and Pillai 1971; Ebenezer and Richard 1999; Cai et al. 2006; de Mazancourt et al. 2020), plumose setae on distal end of telson (6–9 vs 4–10; Holthuis 1965; Tiwari and Pillai 1971; Ebenezer and Richard 1999; Cai et al. 2006; de Mazancourt et al. 2020), spiniform setae on uropodal diaeresis (19–21 vs 15–24; Ebenezer and Richard 1999; Cai et al. 2006; de Mazancourt et al. 2020), and small embryo size (0.42–0.52 × 0.26–0.31 mm vs 0.37–0.54 × 0.23–0.32 mm; Tiwari and Pillai 1971; Ebenezer and Richard 1999; Cai et al. 2006; de Mazancourt et al. 2020).

##### Caridina zhujiangensis

Taxon classificationAnimaliaDecapodaAtyidae

Chen, Chen & Guo, 2018

77BE255A-74DA-5E2F-97CF-59995C783CFD

Figs 5A, 8B, 9

Caridina zhujiangensis Chen et al., 2018: 319, figs 4–6. Type locality “near the Resort Hotel, Dong’ao Island, Guangdong Province (22°01'06"N, 113°42'03"E)", China.
Caridina zhujiangensis — Xu et al. 2020: 21, figs 6, 7; Chen et al. 2020: 18, figs 6, 7; Feng et al. 2021: 34, fig. 2; de Mazancourt et al. 2023b: 291; Cai 2025: 7.
Caridina typus [non H. Milne Edwards, 1837] — Bernardes et al. 2017: 1 (in part; TAL clade).

###### Material examined.

Two lots (see Suppl. material 1: Data S1).

###### Description.

**Figure 9.**
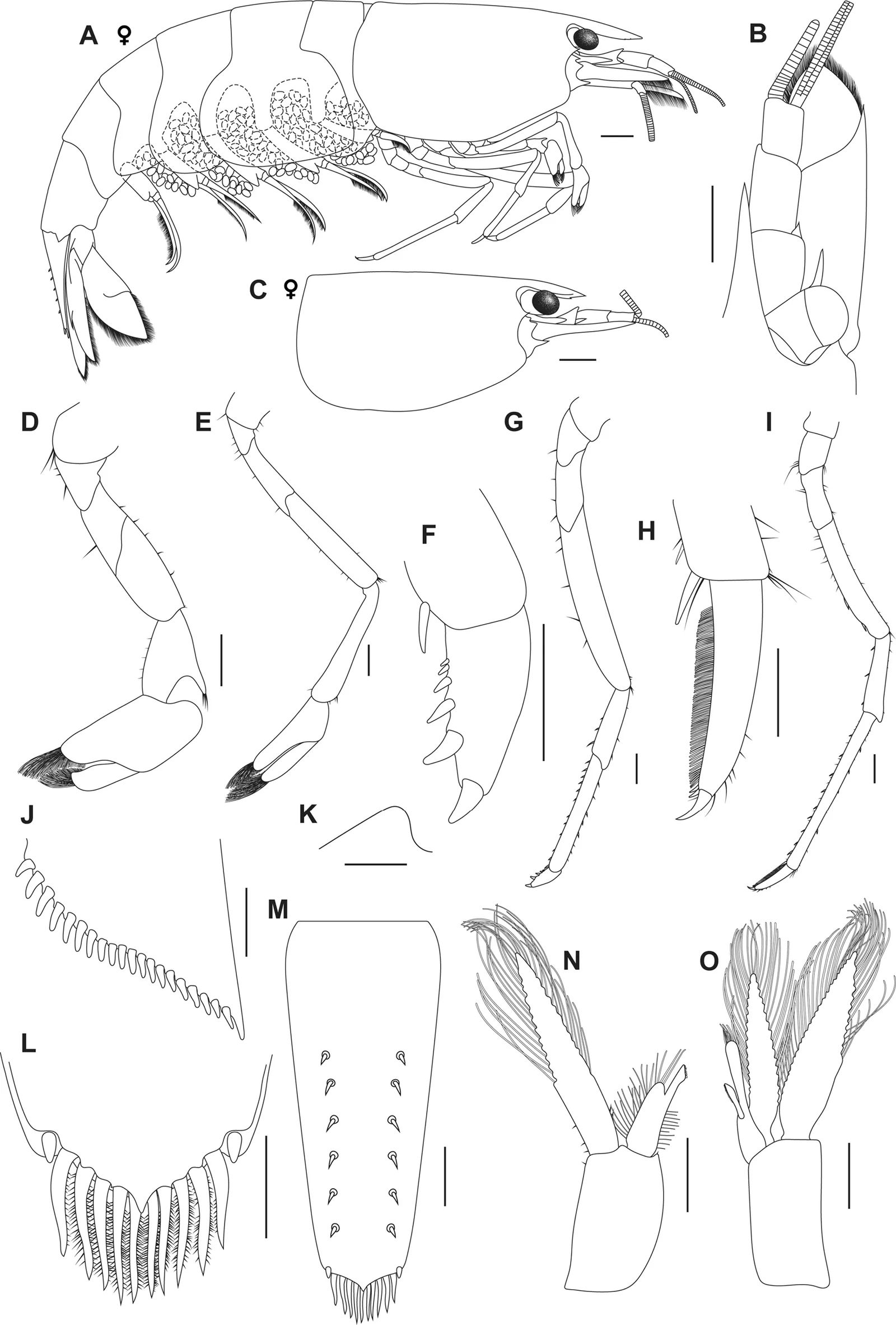
*Caridina zhujiangensis*. **A**. Ovigerous female; **B**. Top view of carapace; **C**. Cephalothorax and cephalic appendages of female; **D**. First pereopod; **E**. Second pereopod; **F**. Dactylus of third pereopod; **G**. Third pereopod; **H**. Dactylus of fifth pereopod; **I**. Fifth pereopod; **J**. Uropodal diaeresis; **K**. Preanal carina; **L**. Distal end of telson; **M**. Telson; **N**. First pleopod; **O**. Second pleopod. Drawings were made of MUMNH-CAR464-D1 (**A, B, D–M**); MUMNH-CAR464-D2 (**C**); MUMNH-CAR464-M1 (**N, O**). Scale bars: 1 mm (**A–C**); 0.5 mm (**D, E, G, I, M–O**); 0.25 mm (**F, H, J–L**).

***Cephalothorax and cephalic appendage*** (*n* = 10). cl 5.39–6.37 (median 5.94; Fig. 9A–C) mm, width 3.68–4.41 (median 4.26) mm. Rostrum straight or slightly curved downward (Fig. 9A, C), reaching beyond the end of first segment of antennular peduncle but not longer than the proximal 1/2 of third segment of antennular peduncle (Fig. 9B), 0.30–0.39 (median 0.35) as long as cl. Rostral formula: (0) 0/ 0–4, ventral teeth minute, placed near the distal end. Antennal spine indistinctively fused with orbital margin. Pterygostomian margin broadly round. Eye well developed, anterior end reaching to 0.55–0.69 (median 0.62) of first segment of antennular peduncle. Antennular peduncle 0.44–0.58 (median 0.53; Fig. 9A–C) as long as cl, first segment 1.61–2.32 (median 1.81) × as long as second segment, second segment 1.19–2.03 (median 1.51) × as long as third segment. Tooth on distolateral margin of first segment of antennular peduncle well-developed. Stylocerite reaching to 0.69–0.86 (median 0.72) of first segment of antennular peduncle. Scaphocerite 2.31–2.75 (median 2.46) × as long as wide, distal margin with short plumose setae.

***Branchial and mouthparts*** (*n* = 3). Podobranch on second maxilliped well-developed. Third maxilliped possessed one small and one large arthrobranch. Pleurobranchs present on all pereopods. Third maxilliped reaching to end of second segment of antennular peduncle; ultimate segment of endopod ending with one large claw and four or five spiniform setae on distal 1/3 of posterior margin, 0.75–0.91 (median 0.84, *n* = 10) as long as penultimate segment.

***Pereopods*** (*n* = 10). Epipod well-developed on first four pereopods. Chelae of P1 and P2 well developed.

P1 short (Fig. 9D); chela 1.88–2.31 (median 2.04) × as long as wide, 1.26–1.75 (median 1.50) × as long as carpus, tips of fingers rounded, with tuft of setae near tip; dactylus 0.56–0.80 (median 0.72) as long as palm; carpus stout, excavated distally, 1.48–2.00 (median 1.72) × as long as wide, 0.75–1.19 (median 0.92) × as long as merus; merus 1.82–2.98 (median 2.33) × as long as wide, 1.18–1.95 (median 1.56) × as long as ischium.

P2 slenderer, longer than P1 (Fig. 9E); chela 2.92–3.78 (median 3.18) × as long as wide, 0.64–0.79 (median 0.70) as long as carpus, tips of fingers rounded, with tuft of setae near tip; dactylus 1.21–1.75 (median 1.48) × as long as palm; carpus slender, excavated distally, 5.12–5.97 (median 5.60) × as long as wide, 0.95–1.09 (median 1.06) × as long as merus; merus 5.51–7.69 (median 7.01) × as long as wide, 1.31–1.70 (median 1.49) × as long as ischium.

P3 not sexually dimorphic (Fig. 9G); dactylus with 3–6 (mode 4) spiniform setae on flexor margin (Fig. 9F), 2.80–4.50 (median 3.88) × as long as wide (including terminal claw), terminating with one large claw; propodus bearing numerous spiniform setae on posterior margin, 8.73–12.25 (median 9.91) × as long as wide, 5.19–6.84 (median 6.14) × as long as dactylus; carpus possessed three or four (mode 4) small setae on posterior margin of outer surface, the distal seta largest, the other setae minute, 4.55–6.07 (median 5.87) × as long as wide, 0.73–0.88 (median 0.77) as long as propodus; merus possessed 2–5 (mode 4) large setae on posterior margin of outer surface, 6.02–7.89 (median 7.29) × as long as wide, 1.74–2.14 (median 2.05) × as long as carpus; ischium without spiniform seta.

P5 slender (Fig. 9I); dactylus with 64–81 spiniform setae on flexor margin (Fig. 9H), 3.78–5.13 (median 4.61) × as long as wide (including terminal claw), terminating with one large claw; propodus bearing numerous spiniform setae on posterior margin, 12.22–15.20 (median 14.10) × as long as wide, 3.39–4.47 (median 3.54) × as long as dactylus; carpus possessed 1–3 (mode 3) small setae on posterior margin of outer surface, the distal seta largest, the other setae minute, 4.21–5.89 (median 5.17) × as long as wide, 0.43–0.56 (median 0.51) as long as propodus; merus possessed two large setae on posterior margin of outer surface, 6.06–7.17 (median 6.88) × as long as wide, 1.41–1.65 (median 1.50) × as long as carpus; ischium without spiniform seta.

***Male pleopods*** (*n* = 10). Pl1 endopod subtriangular (Fig. 9N), wider proximally, 3.38–3.78 (median 3.59) × as long as wide, 0.34–0.53 (median 0.43) of exopod length; appendix interna long with curly bristles at the tip, emerging from distal 1/3 of endopod. Pl2 appendix masculina rod-shaped (Fig. 9O) with many setae, 0.54–0.72 (median 0.66) as long as endopod; appendix interna reaching 0.45–0.63 (median 0.57) of appendix masculina length.

***Abdomen*** (*n* = 10). Sixth abdominal somite ~ 0.44–0.54 (median 0.47) of cl, 1.24–1.67 (median 1.36) × as long as fifth somite, 0.75–1.02 (median 0.91) × as long as telson (Fig. 9A). Telson 2.39–2.84 (median 2.75; Fig. 9M) × as long as wide, with 4–6 pairs (mode 5, 6) of dorsal spiniform setae and one pair of dorsolateral spiniform setae; distal margin subtriangular with posteromedian projection, 8–10 moveable plumose setae, similar in length (Fig. 9L). Preanal carina large, subtriangular, with a few setae, without a spine (Fig. 9K). Uropodal diaeresis with 20–22 moveable spiniform setae (Fig. 9J).

***Embryos***. Ovigerous females with 694–1,277 embryos (mean 999, *n* = 5). Size of embryos with eyes 0.40–0.45 × 0.24–0.27 mm (*n* = 50).

###### Coloration.

Body gray to mist blue, decorated with numerous small dark spots. Rostrum orange to pale brown. Body darker dorsally than ventrally. Embryos bronze (Fig. 8B).

###### Distribution and habitat.

China (Guangdong), Thailand, the Philippines, and Indonesia (Bernardes et al. 2017; Chen et al. 2018; de Mazancourt et al. 2023b; this study). In southern Thailand, *C. zhujiangensis* occurs in one location on the eastern coast of the peninsula (Gulf of Thailand; Fig. 5A), inhabiting streams near the coast. Individuals are typically found beneath accumulations of leaf litter in shallow water over sandy and gravelly substrates.

###### Phylogenetic placement.

The examined *C. zhujiangensis* specimens formed a monophyletic clade in our molecular analyses (BS = 99%, BPP = 1.00; Fig. 2). It was placed in the *C. typus* species group as the basal-most clade.

###### Remarks.

Our specimens possess some different traits to individuals from Dong’ao Island, Guangdong Province, China used in the original description by Chen et al. (2018), by having a longer rostrum (0.30–0.39 as long as cl and always reaching beyond the end of first segment of first antennular peduncle vs 0.15–0.19 as long as cl and reaching near the end of the first segment of antennular peduncle), slenderer P2 chela (2.92–3.74× as long as wide vs 1.9–2.5), slenderer P2 carpus (5.12–5.97× as long as wide vs 4.1–4.7), slenderer P3 propodus (5.19–6.84× as long as dactylus vs 3.4–4.2), a greater number of spiniform setae on P5 dactylus (64–81 vs 45–48), slenderer P5 dactylus (3.78–5.13× as long as wide vs 2.3–3.9), and a slenderer endopod of male first pleopod (3.38–3.78× as long as wide vs 2.5). Such variations are confirmed as intraspecific ranges by molecular analysis in this study. Sequences of our samples and those individuals collected from the same island of the type locality (Xu et al. 2020) were clustered together and formed a well-supported clade.

Although *C. zhujiangensis* and *C. typus* are genetically distinct (Bernardes et al. 2017; this study), their morphology is remarkably similar. The original description by Chen et al. (2018) proposed five characters to differentiate them. *Caridina zhujiangensis* possesses (1) a shorter rostrum (reaching near the end of first segment of antennular peduncle vs reaching near the end of second segment of antennular peduncle in *C. typus*), (2) a broader P2 carpus (4.09–4.64× as long as wide vs 5.4 as in *C. typus*), (3) fewer spiniform setae on the P5 dactylus (45–48 vs 100 as in *C. typus*), (4) an appendix interna on endopod of male first pleopod that is longer than the endopod width and emerges from distal 1/3 of inner margin (vs shorter than proximal width and emerging from distal end of endopod as in *C. typus*), and (5) a shorter appendix interna of male second pleopod, reaching nearly 1/2 length of appendix interna (vs reaching ~ 0.9 as in *C. typus*).

Based on our present observations, these two species in southern Thailand exhibit a considerable degree of overlap in some traits. The number of spiniform setae on the P5 dactylus is similar in both species (64–81 in *C. zhujiangensis* vs 65–72 in *C. typus*). The appendix interna on the endopod of the male first pleopod in both species originates from the distal 1/3 of the inner margin, which is consistent with previous reports (Ebenezer and Richard 1999; Cai et al. 2006; de Mazancourt et al. 2020). Furthermore, the P2 carpus of *C. zhujiangensis* is as broad as that of *C. typus* (5.12–5.97× as long as wide vs 4.69–6.85). Despite these similarities, we observed key morphological differences that are consistent with Chen et al. (2018). The rostrum of *C. zhujiangensis* is likely shorter than that of *C. typus* (0.30–0.39 as long as cl vs 0.38–0.53). The appendix interna on endopod of male first pleopod of *C. zhujiangensis* is much longer than that of *C. typus* (slightly longer than endopod width vs shorter than endopod width in *C. typus*). Additionally, we confirm that the P1 dactylus of *C. zhujiangensis* is clearly shorter than that of *C. typus* (0.56–0.80 as long as palm vs 0.86–1.42).

#### *Caridina sumatrensis* species group

**Diagnosis**. Rostrum short, straight, or slightly pointed down, usually not reaching beyond the end of the third segment of antennular peduncle, and with a basal ridge. Dorsal teeth placed throughout the dorsal margin. Subapical teeth always absent. Antennular peduncle blunt, ~ 0.4–0.7 as long as cl. P3 and P5 dactylus stout. Epipods well developed on P1–P4. Sixth abdominal somite ~ 0.4–0.6 as long as cl. Lateral plumose setae of distal end of telson slightly longer than median plumose setae. Preanal carina high, subtriangular, without a spine. Uropodal diaeresis with > 15 moveable spiniform setae. Endopod of male first pleopod subtriangular with an appendix interna emerging near the tip.

**Remarks**. At least four species are included in this species group, consisting of *C. cf. babaulti* Bouvier, 1918, *C. kuehnei* sp. nov., *C. maeklongensis* Macharoenboon, Manonai & Jeratthitikul, 2024, and *C. sumatrensis*. All of these species were recovered as monophyletic with high nodal support (BS = 91–100%, BPP = 0.99–1.00; Fig. 2). Phylogenetically, *C. kuehnei* sp. nov. is the basal clade of the species group and sister to the clade of *C. cf. babaulti*, *C. maeklongensis*, and *C. sumatrensis*.

Our phylogenetic tree indicated that the *C. sumatrensis* species group is the sister group of the *C. lanceifrons* species group, albeit with low nodal support (BS = 40%, BPP = 0.6; Fig. 2). These two species groups share some diagnostic characters including the overall shape of rostrum, a basal ridge on the rostrum, blunt antennular peduncle, high unarmed preanal carina, many spiniform setae on the uropodal diaeresis, and the presence of appendix interna on the endopod of male Pl1. Nevertheless, telson characters are useful for discriminating between these two species groups. The median plumose setae on distal end of telson of *C. sumatrensis* species group are slightly shorter than the lateral plumose setae, whereas *C. lanceifrons* species group possesses median plumose setae on the distal end of telson that are slightly longer than the lateral plumose setae.

##### Caridina sumatrensis

Taxon classificationAnimaliaDecapodaAtyidae

De Man, 1892

9C54F115-ADA7-5973-A14B-E68C1D21FB79

Figs 5B, 8C–E, 10

Caridina weberi var. sumatrensis De Man, 1892: 375, pl. 22, fig. 23g. Type locality “den Flüssen des unteren Battaklandes bei Deli, an der Ostküste von Sumatra” [= River near Deli, east coast of Sumatra, Indonesia].
Caridina weberi var. sumatrensis — Kemp 1918a: 99; Pillai 1964: 43.
Caridina weberi sumatrensis — Bouvier 1904: 132; Bouvier 1905: 75, 83; Kemp 1918b: 292; Bouvier 1925: 247, fig. 567; Johnson 1961b: 46.
Caridina sumatrensis — Wowor et al. 2004: 343, fig. 6p, q; Cai and Shokita 2006b: 246, fig. 6c–f; Cai et al. 2007: 285, fig. 7; Naiyanetr 2007: 34; de Mazancourt et al. 2020: 40; Macharoenboon et al. 2024: 461, figs 4e–f, 6; Cai and Naiyanetr 2024: 774, figs 2, 3.
Caridina weberi [non De Man, 1892] — Naiyanetr 2007: 34.

###### Material examined.

40 lots (see Suppl. material 1: Data S1).

###### Description.

**Figure 10.**
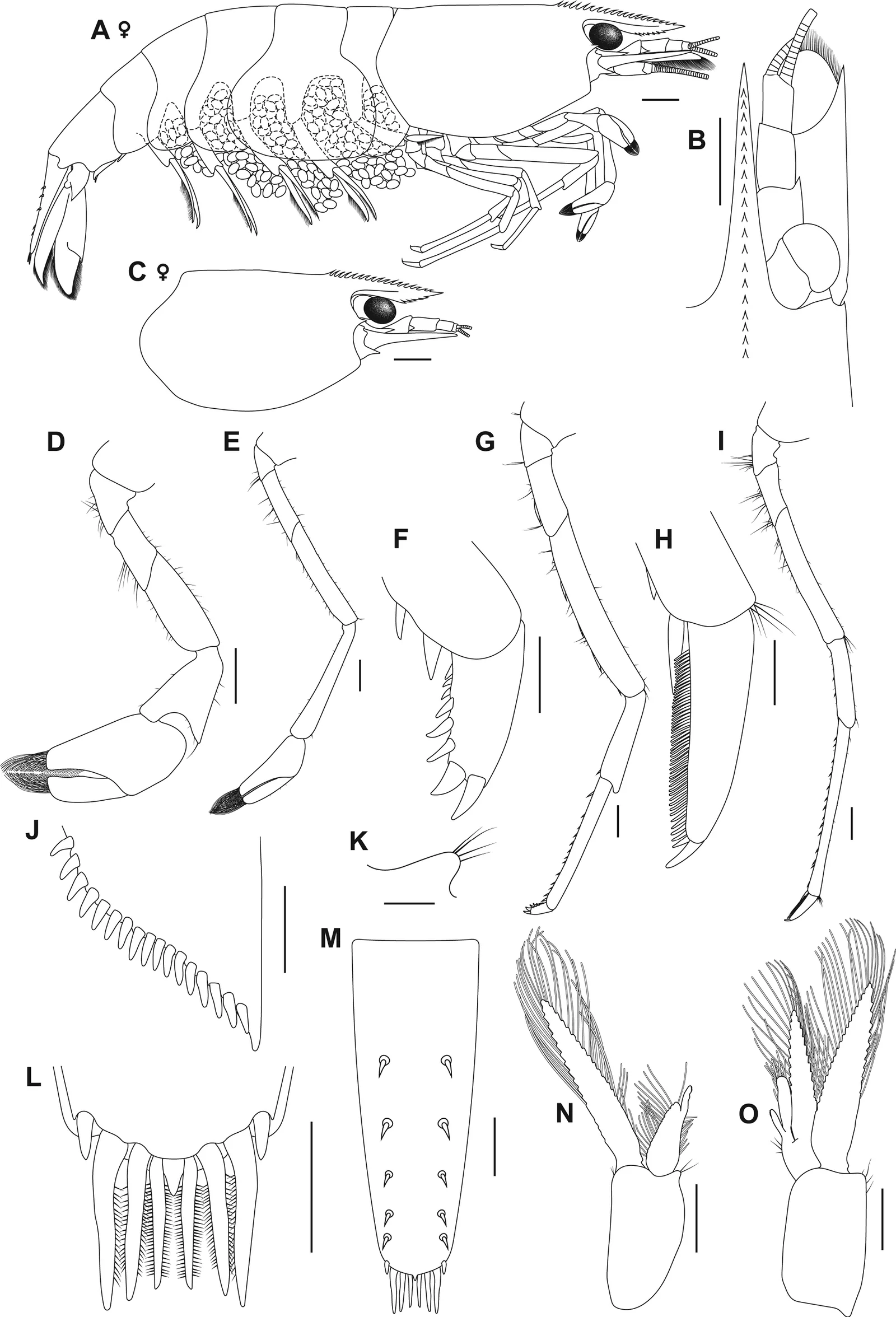
*Caridina sumatrensis*. **A**. Ovigerous female; **B**. Top view of carapace; **C**. Cephalothorax and cephalic appendages of female; **D**. First pereopod; **E**. Second pereopod; **F**. Dactylus of third pereopod; **G**. Third pereopod; **H**. Dactylus of fifth pereopod; **I**. Fifth pereopod; **J**. Uropodal diaeresis; **K**. Preanal carina; **L**. Distal end of telson; **M**. Telson; **N**. First pleopod; **O**. Second pleopod. Drawings were made of MUMNH-CAR440-D1 (**A, B, D–M**); MUMNH-CAR178-D1 (**C**); MUMNH-CAR440-M1 (**N, O**). Scale bars: 1 mm (**A–C**); 0.5 mm (**D, E, G, I, M–O**); 0.25 mm (**J–L**); 0.1 mm (**F, H**).

***Cephalothorax and cephalic appendage*** (*n* = 10). cl 4.05–5.82 (median 4.95; Fig. 10A, C) mm, width 2.97–4.44 (median 3.58) mm. Rostrum slightly pointed downward (Fig. 10A–C), with conspicuous basal ridge, reaching near the end of third segment of antennular peduncle (Fig. 10B), or at least reaching beyond the end of second segment of antennular peduncle, 0.46–0.64 (median 0.51) as long as cl. Rostral formula: (3–6) 14–20/ 5–8, dorsal teeth usually placed throughout dorsal margin. Antennal spine placed slightly below inferior orbital angle. Pterygostomian margin subrectangular. Eye well developed, anterior end reaching to 0.53–0.72 (median 0.62) of first segment of antennular peduncle. Antennular peduncle 0.52–0.67 (median 0.56; Fig. 10A–C) as long as cl, first segment 1.84–2.84 (median 2.14) × as long as second segment, second segment 1.00–1.77 (median 1.47) × as long as third segment. Tooth on distolateral margin of first segment of antennular peduncle well developed. Stylocerite reaching to 0.61–0.78 (median 0.71) of first segment of antennular peduncle. Scaphocerite 2.48–3.05 (median 2.73) × as long as wide, distal margin with short plumose setae.

***Branchial and mouthparts*** (*n* = 3). Podobranch on second maxilliped well developed. Third maxilliped possessed one small and one large arthrobranchs. Pleurobranchs present on all pereopods. Third maxilliped reaching to end of second segment of antennular peduncle; ultimate segment of endopod ending with one large claw and six to eight spiniform setae on distal 1/3 of posterior margin, 0.77–0.90 (median 0.84, *n* = 10) as long as penultimate segment.

***Pereopods*** (*n* = 10). Epipod well developed on first four pereopods. Chelae of P1 and P2 well developed.

P1 short (Fig. 10D); chela 1.67–2.31 (median 1.95) × as long as wide, 1.22–1.62 (median 1.42) × as long as carpus, tips of fingers rounded, with tuft of setae near tip; dactylus 0.99–1.42 (median 1.13) × as long as palm; carpus stout, excavated distally, 1.77–2.48 (median 2.18) × as long as wide, 0.79–0.97 (median 0.89) as long as merus; merus 2.40–3.14 (median 2.88) × as long as wide, 1.40–1.87 (median 1.69) × as long as ischium.

P2 slenderer and longer than P1 (Fig. 10E); chela 2.67–3.61 (median 2.92) × as long as wide, 0.63–0.73 (median 0.69) as long as carpus, tips of fingers rounded, with tuft of setae near tip; dactylus 1.13–2.20 (median 1.62) × as long as palm; carpus slender, 4.82–6.25 (median 5.65) × as long as wide, 1.02–1.18 (median 1.09) × as long as merus; merus 5.67–6.81 (median 6.30) × as long as wide, 1.43–1.90 (median 1.62) × as long as ischium.

P3 not sexually dimorphic (Fig. 10G); dactylus with 5–8 (mode 5–6) spiniform setae on flexor margin (Fig. 10F), 2.46–4.40 (median 3.21) × as long as wide (including terminal claw), terminating with one large claw; propodus bearing numerous spiniform setae on posterior margin, 8.97–11.99 (median 10.50) × as long as wide, 4.65–6.33 (median 5.39) × as long as dactylus; carpus possessed 4–6 (mode 4) setae on posterior margin of outer surface, the distal seta largest, the other setae minute, 5.93–7.49 (median 6.55) × as long as wide, 0.51–0.61 (median 0.56) as long as propodus; merus possessed 3–5 (mode 4) large setae on posterior margin of outer surface, 6.15–7.02 (median 6.59) × as long as wide, 1.73–2.31 (median 1.98) × as long as carpus; ischium with one spiniform seta.

P5 slender (Fig. 10I); dactylus with 34–47 spiniform setae on flexor margin (Fig. 10H), 3.48–4.64 (median 3.96) × as long as wide (including terminal claw), terminating with one large claw; propodus bearing numerous spiniform setae on posterior margin, 15.35–18.56 (median 17.32) × as long as wide, 4.66–5.77 (median 5.23) × as long as dactylus; carpus possessed 1–4 spiniform setae on posterior margin of outer surface, the distal seta largest, the other setae minute, 4.69–6.34 (median 5.64) × as long as wide, 0.49–0.57 (median 0.52) as long as propodus; merus possessed one or two (mode 2) large setae on posterior margin of outer surface, 7.00–9.03 (median 7.55) × as long as wide, 1.46–1.74 (median 1.58) × as long as carpus; ischium without spiniform seta.

***Male pleopods*** (*n* = 10). Pl1 endopod subtriangular (Fig. 10N), wider proximally, 2.02–3.05 (median 2.31) × as long as wide, 0.32–0.41 (median 0.34) of exopod length, with appendix interna emerged near the tip. Pl2 appendix masculina rod-shaped (Fig. 10O), with many setae, 0.59–0.78 (median 0.69) as long as endopod; appendix interna reaching 0.62–0.72 (median 0.65) of appendix masculina length.

***Abdomen*** (*n* = 10). Sixth abdominal somite ~ 0.38–0.55 (median 0.45) of cl, 1.14–1.64 (median 1.47) × as long as fifth somite, 0.75–1.03 (median 0.86) × as long as telson (Fig. 10A). Telson 2.37–3.11 (median 2.77; Fig. 10M) × as long as wide, with four or five pairs (mode 5) of dorsal spiniform setae and one pair of dorsolateral spiniform setae; distal margin round with posteromedian projection and 6–9 moveable plumose setae, similar in length or lateral pair slightly longer than intermediate pair (Fig. 10L). Preanal carina subtriangular, with a few setae, without a spine (Fig. 10K). Uropodal diaeresis with 17–22 moveable spiniform setae (Fig. 10J).

***Embryos***. Ovigerous females with 472–1,296 embryos (mean 776, *n* = 10). Size of eye-developed embryos 0.38–0.52 × 0.23–0.34 mm (*n* = 50).

###### Coloration.

Body color variable, being either reddish orange, bluish gray, or dark teal blue. Body usually decorated with numerous small dark spots. Rostrum yellow to gold. Each abdominal somite dorsally decorated with conspicuous light transverse bands. Embryos orange to dark brown (Fig. 8C–E).

###### Distribution and habitat.

Indonesia (Sumatra), the Philippines, Singapore, Malaysia, Thailand, Myanmar, and India (Kemp 1918a, 1918b; Cai et al. 2007; Cai and Naiyanetr 2024). In southern Thailand, *C. sumatrensis* is mainly distributed on the western coast of the peninsula (Andaman Sea; Fig. 5B), with some populations being found in the basins draining to the Gulf of Thailand. This species inhabits a wide range of freshwater habitats, from coastal lowland rivers to headwater streams, where the bottom substrate usually consists of gravel or sand. Individuals are commonly found among aquatic vegetation along stream banks, within submerged root systems of riparian plants, beneath leaf litter, or on the undersides of rocks.

###### Phylogenetic placement.

Our molecular analyses show *C. sumatrensis* to be monophyletic (BS = 100%, BPP = 1.00; Fig. 2) as part of the *C. sumatrensis* species group together with *C. cf. babaulti*, *C. kuehnei* sp. nov., and *C. maeklongensis*, but the relationship of these species is not resolved with support.

###### Remarks.

*Caridina sumatrensis* was originally proposed as a subspecies of *C. weberi* by De Man (1892). It was latter treated as a valid species by Wowor et al. (2004), where the name “*C. sumatrensis*” was first applied in the keys. This taxonomic treatment has been widely accepted in subsequent works (Cai and Shokita 2006b; Cai et al. 2007; de Mazancourt et al. 2020; Cai and Naiyanetr 2024; Macharoenboon et al. 2024). *Caridina sumatrensis* differs from the typical form of *C. weberi* in being smaller but having a longer rostrum (extending near the end of third segment of antennular peduncle vs extending to base or near middle of second segment of antennular peduncle), the higher number of rostral teeth, particularly on the dorsal teeth (16–20 vs 10–19, usually 13–14) and the postorbital teeth (3–6 vs 0–2) (De Man 1892; de Mazancourt et al. 2020). They also differ in several characters beyond the rostrum structure, as follows. *Caridina sumatrensis* possesses slightly slender P2 carpus (4.82–6.25× as long as wide vs 4.8–5.5), smaller number of spiniform setae on P5 dactylus (34–47 vs 47–66), slightly shorter appendix interna of male second pleopod (0.58–0.67× as long as wide vs 0.75), smaller number of dorsal spiniform setae on telson (4–5 pairs vs 5–7 pairs), and smaller number of plumose setae at distal end of telson (6–8 vs 6–11) (de Mazancourt et al. 2020).

The rostral formula of our *C. sumatrensis* specimens matches well with the original description: (3–6) 14–20/ 5–8 vs (3–6) 16–20/ 5–6 (De Man 1892). Our specimens are also consistent with previous works in term of the rostral formula: (3–6) 14–20/ 5–8 vs (0–9) 12–24/ 3–6 (Kemp 1918b; Pillai 1964; Macharoenboon et al. 2024); the rostral length (0.46–0.64× as long as cl vs 0.50–0.60; Macharoenboon et al. 2024), the P1 chela (1.95–2.33× as long as wide vs 1.90–2.66; Cai and Naiyanetr 2024; Macharoenboon et al. 2024), the P1 carpus (1.67–2.31× as long as wide vs 1.60–2.66; Kemp 1918b; Pillai 1964; Macharoenboon et al. 2024), the P2 chela (2.67–3.61× as long as wide vs 2.71–3.40; Macharoenboon et al. 2024), the P2 carpus (4.82–6.25× as long as wide vs 4.63–6.69; Cai and Naiyanetr 2024; Macharoenboon et al. 2024), spiniform setae on P3 dactylus (5–8 vs 5–7; Kemp 1918b; Pillai 1964; Macharoenboon et al. 2024), spiniform setae of P5 dactylus (34–47 vs 27–49; Kemp 1918b; Pillai 1964; Cai et al. 2007; Cai and Naiyanetr 2024; Macharoenboon et al. 2024), spiniform setae on uropodal diaeresis (17–21 vs 15–22; Cai and Naiyanetr 2024; Macharoenboon et al. 2024), and the embryo size (0.38–0.52 × 0.23–0.34 mm vs 0.40–0.46 × 0.22–0.29 mm; Cai and Naiyanetr 2024; Macharoenboon et al. 2024).

##### Caridina kuehnei sp. nov.

Taxon classificationAnimaliaDecapodaAtyidae

4AED56D6-A27B-58AE-B0E8-60EF5CA147E6

https://zoobank.org/16FF23A1-D0C7-45FD-B4B3-B6720AFFFFFC

Figs 5B, 8F, 11, 12

###### Material examined.

***Holotype*. Thailand** • ovigerous ♀, cl 3.41 mm; Krabi Province, Khlong Thom District, Thom Stream (connected to Emerald Pool); 7.9230°N, 99.2598°E; MUMNH-CAR438-H, coll. K. Macharoenboon, E. Jeratthitikul & N. Wutthituntisil, 14 June 2022. ***Paratypes*. Thailand** • 12 ♀, cl 2.38–3.40 mm; same data as for holotype; MUMNH-CAR438-F1 to F6, F8, CAR438-P1, coll. K. Macharoenboon, E. Jeratthitikul & N. Wutthituntisil, 14 June 2022, ZMB29671-1, ZMB29671-2, and ZMB32388-2, ZMB29672-3, coll. J. Kühne, 2012 • 9 ♂, cl 2.19–2.70 mm; MUMNH-CAR438-M1 to M6 coll. K. Macharoenboon, E. Jeratthitikul & N. Wutthituntisil, 14 June 2022, ZMB29672-1, ZMB29672-2, ZMB32388-3, coll. J. Kühne, 2012.

###### Description.

**Figure 11.**
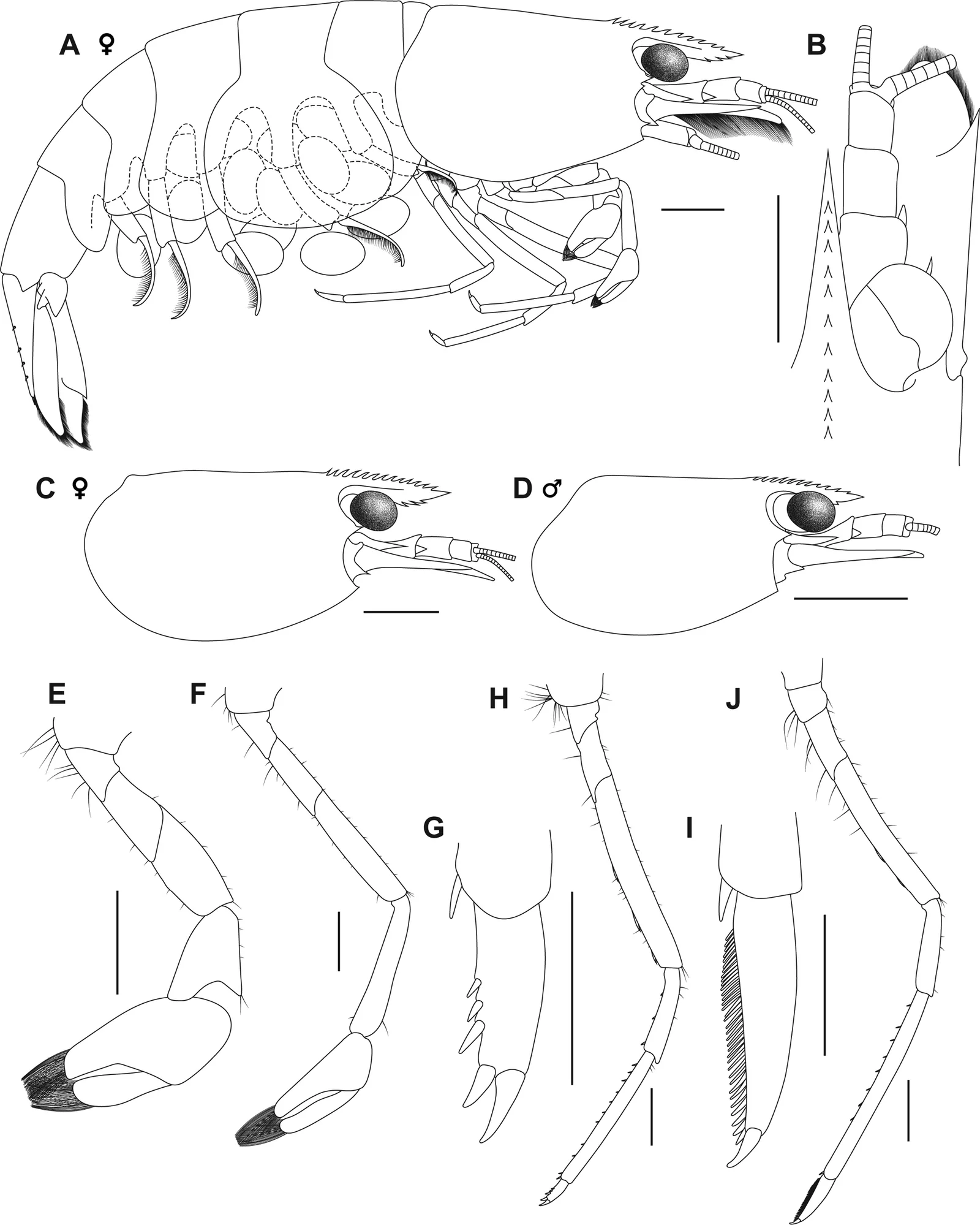
*Caridina kuehnei* sp. nov. **A**. Ovigerous female; **B**. Top view of carapace; **C**. Cephalothorax and cephalic appendages of female; **D**. Cephalothorax and cephalic appendages of male; **E**. First pereopod; **F**. Second pereopod; **G**. Dactylus of third pereopod; **H**. Third pereopod; **I**. Dactylus of fifth pereopod; **J**. Fifth pereopod. Drawings were made of paratype MUMNH-CAR438-F8 (**A, B, E–J**); paratype MUMNH-CAR438-F1 (**C**); paratype MUMNH-CAR438-M1 (**D**). Scale bars: 1 mm (**A–C**); 0.5 mm (**D–F, H, J**); 0.25 mm (**G, I**).

***Cephalothorax and cephalic appendage*** (*n* = 7). cl 2.43–3.65 (median 3.02; Fig. 11A, C) mm, width 2.54–2.81 (median 2.73) mm. Rostrum slightly pointed downward (Fig. 11A–C), reaching near the end of second segment of antennular peduncle (Fig. 11B), 0.39–0.54 (median 0.51) as long as cl. Rostral formula: (3–4) 10–16/ 1–4, dorsal teeth usually placed throughout dorsal margin. Antennal spine placed slightly below inferior orbital angle or fused with orbital angle. Pterygostomian margin acute or subrectangular, rarely with a minute spine. Eye well developed, anterior end reaching to 0.57–0.76 (median 0.67) of first segment of antennular peduncle. Antennular peduncle 0.51–0.71 (median 0.65; Fig. 11A–C) as long as cl, first segment 1.98–2.67 (median 2.26) × as long as second segment, second segment 1.08–1.50 (median 1.21) × as long as third segment. Tooth on distolateral margin of first segment of antennular peduncle well developed. Stylocerite reaching to 0.71–0.83 (median 0.81) of first segment of antennular peduncle. Scaphocerite 2.47–3.10 (median 2.68) × as long as wide, distal margin with short plumose setae.

***Branchial formula*** (*n* = 7). Podobranch on second maxilliped well developed. Third maxilliped possessed one small and one large arthrobranchs. Pleurobranchs present on all pereopods.

**Figure 12.**
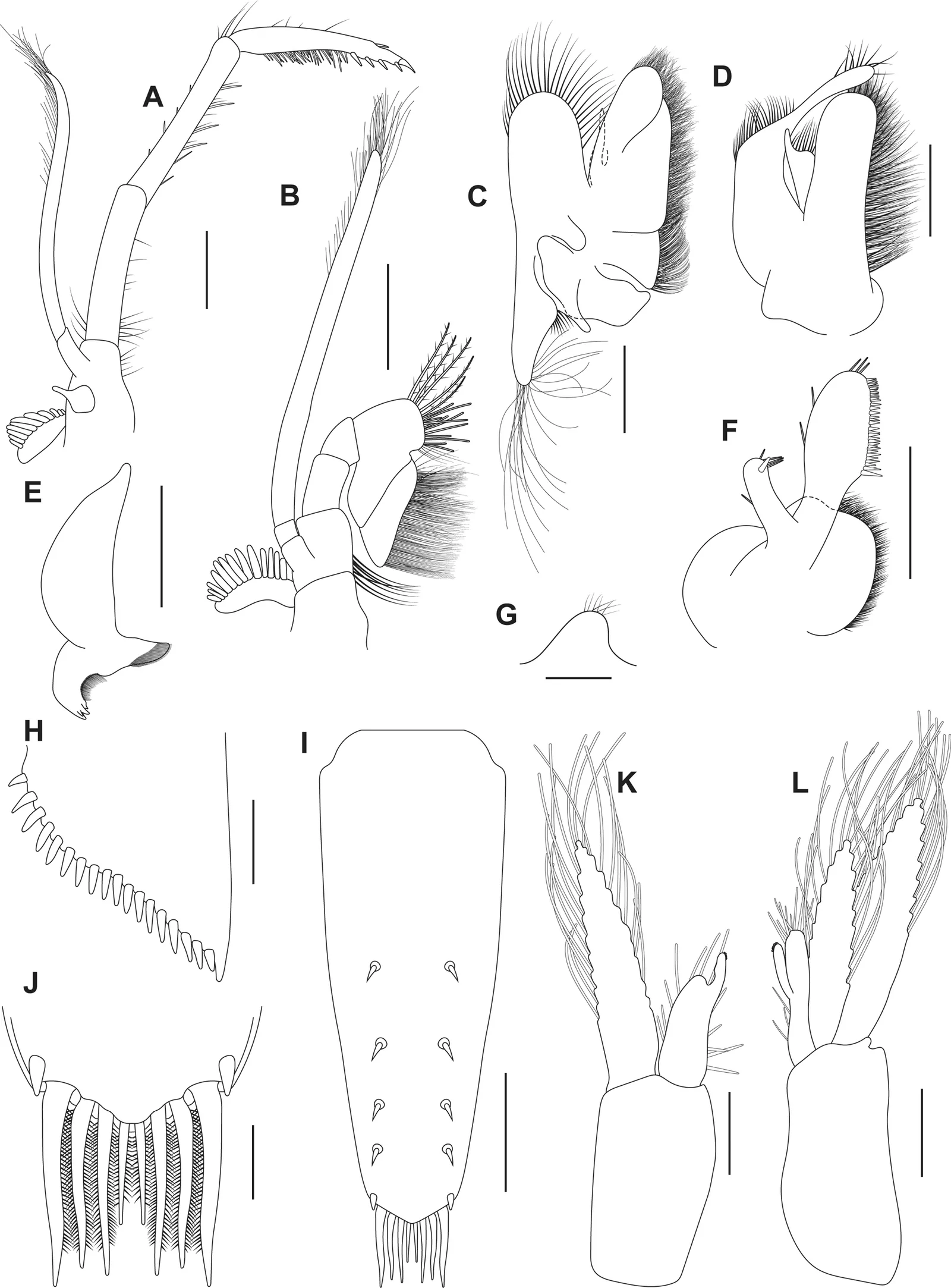
*Caridina kuehnei* sp. nov. **A**. Third maxilliped; **B**. Second maxilliped; **C**. Maxilla; **D**. First maxilliped; **E**. Mandible; **F**. Maxillula; **G**. Preanal carina; **H**. Uropodal diaeresis; **I**. Telson; **J**. Distal end of telson; **K**. First pleopod; **L**. Second pleopod. Drawings were made of paratype MUMNH-CAR438-F8 **(A–I)**; paratype MUMNH-CAR438-M1 (**K**, **L**). Scale bars: 0.5 mm (**A–F, I, K, L**); 0.25 mm (**J**); 0.1 mm (**G, H**).

***Mouthparts*** (*n* = 3). Mandible without palp (Fig. 12E); incisor process possessed with 4–6 irregular teeth, and two rows of setae placed along inner margin; molar process truncated. Lower lacinia of maxillula subrectangular (Fig. 12F), with many rows of plumose setae; upper lacinia elongate, inner margin straight, with numerous conical setae and distal setae; palp elongate, with one conical seta and few distal setae. Scaphognathite of maxilla wide anteriorly and tapering posteriorly (Fig. 12C), ending in a tuft of setae and short marginal setae placed along proximal triangular process; anterior margin with long plumose setae; palp slender; upper and middle endites subdivided with many rows of marginal setae; lower endite partly fused with middle endite, with relatively longer marginal setae.

Palp of first maxilliped triangular (Fig. 12D), ending in slender triangular projection and with few plumose setae; caridean lobe broad, with marginal plumose setae; exopod well developed with distally marginal plumose setae; ultimate and penultimate segments of endopod indistinctly divided, with marginal setae. Ultimate and penultimate segments of endopod of second maxilliped incompletely divided (Fig. 12B), inner margin of ultimate and penultimate segments with simple and pappose setae; exopod long and slender, with a tuft of long setae at the tip. Third maxilliped with epipod (Fig. 12A), reaching to end of second segment of antennular peduncle; ultimate segment of endopod ending with one large claw and four to six spiniform setae on distal 1/3 of posterior margin, 0.90–1.11 (median 1.02, *n* = *4*) × as long as penultimate segment.

***Pereopods*** (*n* = 6). Epipod well developed on first four pereopods. Chelae of first and second pereopods well developed.

P1 short (Fig. 11E); chela 1.83–1.98 (median 1.85) × as long as wide, 1.39–1.82 (median 1.42) × as long as carpus, tips of fingers rounded, with tuft of setae near tip; dactylus 0.92–1.15 (median 0.95) × as long as palm; carpus stout, excavated distally, 1.39–1.70 (median 1.56) × as long as wide, 0.81–1.00 (median 0.96) × as long as merus; merus 2.27–2.60 (median 2.43) × as long as wide, 1.25–1.77 (median 1.50) × as long as ischium.

P2 slenderer, longer than P1 (Fig. 11F); chela 2.33–2.65 (median 2.56) × as long as wide, 0.78–0.88 (median 0.83) as long as carpus, tips of fingers rounded, with tuft of setae near tip. Dactylus 1.12–1.89 (median 1.38) × as long as palm; carpus slender, 4.44–5.54 (median 5.21) × as long as wide, 1.07–1.25 (median 1.09) × as long as merus; merus 4.63–5.15 (median 4.93) × as long as wide, 1.31–1.85 (median 1.51) × as long as ischium.

P3 not sexually dimorphic (Fig. 11H); dactylus with four spiniform setae on flexor margin (Fig. 11G), 2.63–3.50 (median 3.11) × as long as wide (including terminal claw), terminating with one large claw; propodus bearing numerous spiniform setae on posterior margin, 9.02–11.25 (median 9.62) × as long as wide, 3.29–5.07 (median 4.33) × as long as dactylus; carpus possessed three or four setae on posterior margin of outer surface, the distal seta largest, the other setae minute, 4.29–5.67 (median 4.78) × as long as wide, 0.57–0.76 (median 0.63) as long as propodus; merus possessed three or four large setae on posterior margin of outer surface, 5.99–7.97 (median 7.23) × as long as wide, 2.00–2.60 (median 2.12) × as long as carpus; ischium with one spiniform seta.

P5 slender (Fig. 11J); dactylus with 22–34 spiniform setae on flexor margin (Fig. 11I), 3.15–4.50 (median 3.79) × as long as wide (including terminal claw), terminating with one large claw; propodus bearing numerous spiniform setae on posterior margin, 11.07–14.20 (median 12.26) × as long as wide, 3.33–4.43 (median 3.97) × as long as dactylus; carpus possessed 1–4 (mode 3) large setae on posterior margin of outer surface, 4.70–5.68 (median 5.01) × as long as wide, 0.45–0.67 (median 0.50) as long as propodus; merus possessed two large setae on posterior margin of outer surface, 6.71–7.76 (median 7.06) × as long as wide, 1.57–1.97 (median 1.79) × as long as carpus; ischium without spiniform seta.

***Male pleopods*** (*n* = 6). Pl1 endopod subtriangular (Fig. 12K), wider proximally, 2.14–2.71 (median 2.21) × as long as width, 0.31–0.51 (median 0.39) of exopod length, with appendix interna emerged near the tip. Pl2 appendix masculina rod-shaped (Fig. 12L), with many long setae, 0.58–0.64 (median 0.59) as long as endopod; appendix interna reaching 0.75–0.89 (median 0.83) of appendix masculina length.

***Abdomen*** (*n* = 6). Sixth abdominal somite ~ 0.45–0.54 (median 0.52) of cl, 1.26–1.72 (median 1.43) × as long as fifth somite, 0.74–1.00 (median 0.84) × as long as telson (Fig. 11A). Telson 2.39–2.88 (median 2.68; Fig. 12I) × as long as wide, with three to five pairs of dorsal spiniform setae and one pair of dorsolateral spiniform setae; distal margin subtriangular with a tiny posteromedian projection and with eight plumose setae, lateral pairs strongly longer than the intermediate pair (Fig. 12J). Preanal carina rounded to subtriangular, with a few setae, without a spine (Fig. 12G). Uropodal diaeresis with 15–19 moveable spiniform setae (Fig. 12H), outermost spiniform seta shorter than lateral angle.

***Embryos***. Ovigerous females with 18–26 embryos (mean 20, *n* = 5). Size of embryos with eyes 0.92–1.07 × 0.54–0.67 mm (*n* = 50).

###### Coloration.

Body dark grayish blue, furnished with numerous small red and orange spots. Rostrum pastel gray. Cephalic region and upper abdominal somite darker than the other parts. Each abdominal somite dorsally decorated with conspicuous light transverse bands. Embryos greenish black (Fig. 8F).

###### Distribution and habitat.

Thailand. This new species is currently known only from the type locality (Fig. 5B), where specimens were collected from submerged root systems of riparian plants in a stream connected to the Emerald Pool, a famous isolated freshwater spring in Krabi Province.

###### Phylogenetic placement.

The molecular analyses show *C. kuehnei* sp. nov. to be monophyletic (BS = 91%, BPP = 0.99; Fig. 2) as part of the *C. sumatrensis* species group.

###### Etymology.

The specific name, *C. kuehnei* sp. nov., is given in honor of Mr. Jens Kühne, a resident of Thailand who first collected this new species and other material, thus making a substantial contribution to the study of freshwater shrimps in Thailand.

###### Remarks.

This species is very similar to *C. maeklongensis* in having a short rostrum, stout P1 and P2 chelae, the same number of spiniform setae on P5 dactylus and uropodal diaeresis, the reduced posteromedian projection, and producing a few very large-sized embryos. However, *C. kuehnei* sp. nov. possesses fewer spiniform setae on P3 dactylus (4 vs 5–7 in *C. maeklongensis*). Furthermore, their geographic distributions are allopatric. The occurrence of the new species is currently restricted to a single stream in southern Thailand, whereas *C. maeklongensis* is found exclusively within the Mae Klong Basin, western part of Thailand (Macharoenboon et al. 2024).

*Caridina kuehnei* sp. nov. also resembles *C. sumatrensis*. However, they are easily distinguishable by the following characters. The rostrum of *C. kuehnei* usually reaches near the end of second segment of antennular peduncle, whereas that of *C. sumatrensis* reaches near the end of the third segment of antennular peduncle. P1 carpus of *C. kuehnei* is stouter (1.39–1.70× as long as wide vs 1.67–2.31), and P2 chela is stouter (2.33–2.65× as long as wide vs 2.67–3.61). P3 dactylus of *C. kuehnei* possesses fewer spiniform setae (4 vs 5–8), as well as P5 dactylus (31–34 vs 34–47). Finally, *C. kuehnei* possesses much larger embryos than of *C. sumatrensis* (0.92–1.07 × 0.52–0.67 mm vs 0.38–0.52 × 0.23–0.34 mm).

#### *Caridina lanceifrons* species group

**Diagnosis**. Rostrum straight, or slightly pointed down, usually reaching beyond the end of the third segment of antennular peduncle. Dorsal teeth placed throughout the dorsal margin. Subapical teeth always absent. Antennular peduncle blunt, ~ 0.4–0.7× as long as carapace length. P3 and P5 dactylus stout. Sixth abdominal somite ~ 0.5–0.6× as long as cl. Epipods well developed on P1–P4. Telson with a posteromedian projection. Median plumose setae on distal end of telson slightly longer than lateral plumose setae or subequal in length. Preanal carina high, subtriangular, without a spine. Uropodal diaeresis with > 15 moveable spiniform setae. Endopod of male first pleopod long subtriangular with an appendix interna, emerging from distal 1/3.

**Remarks**. Our phylogenetic tree recovered three species found in Thailand as monophyletic in this species group, viz. *C. lanceifrons*, *C. sirindhornae* Macharoenboon, Sutcharit, Siriwut & Jeratthitikul, 2025, and *C. thai*, with high nodal support (BS = 100%, BPP = 1.00; Fig. 2), except for *C. lanceifrons* (BS = 51%, BPP = 0.86; Fig. 2). However, the relationship of these species was not significantly supported. All species are found in lotic habitats such as streams and waterfalls (Cai and Naiyanetr 2024; Macharoenboon et al. 2025).

##### Caridina lanceifrons

Taxon classificationAnimaliaDecapodaAtyidae

Yu, 1936

EA03B90E-B399-505D-AE41-D66580980F7E

Figs 5A, 8G, 8H, 13

Caridina lanceifrons Yu, 1936: 89, figs 5–7. Type locality Near the light house at Hai-kiu-sche, in the salt water, Hainan Province, China.
Caridina lanceifrons — Yu 1938: 281, fig. 3; Dudgeon 1985: 142; Liang and Zheng 1988: 15; Zheng 1989: 6; Liang and Zhou 1993: 231; Liang et al. 1993: 41; Li and Liang 2002: 709; Cai 2014: 217, figs 6–7; Do et al. 2021b: 15, figs 5–7.
Caridina flavilineata Dang, 1975: 70–71, fig. 5. Type locality “Nam hà (Bắc Việt nam)” [= Ha Nam and Nam Dinh provinces, northern Vietnam].
Caridina flavilineata — Dang and Do 2008: 8–10, fig. 5; Dang and Ho 2012: 154–156, fig. 53.
Caridina vietriensis Dang & Do, 2007: 9–10, figs 7–8. Type locality “ngã ba sông Việt Trì, Phú Thọ” [= River in Phu Tho Province, Vietnam].
Caridina pseudoflavilineata Do & Dang, 2010: 31–34, figs 3–4. Type locality “suối trên đèo Hải Vân” [= Stream on Hai Van Pass, Thua Thien Hue Province, Vietnam].

###### Material examined.

21 lots (see Suppl. material 1: Data S1).

###### Description.

**Figure 13.**
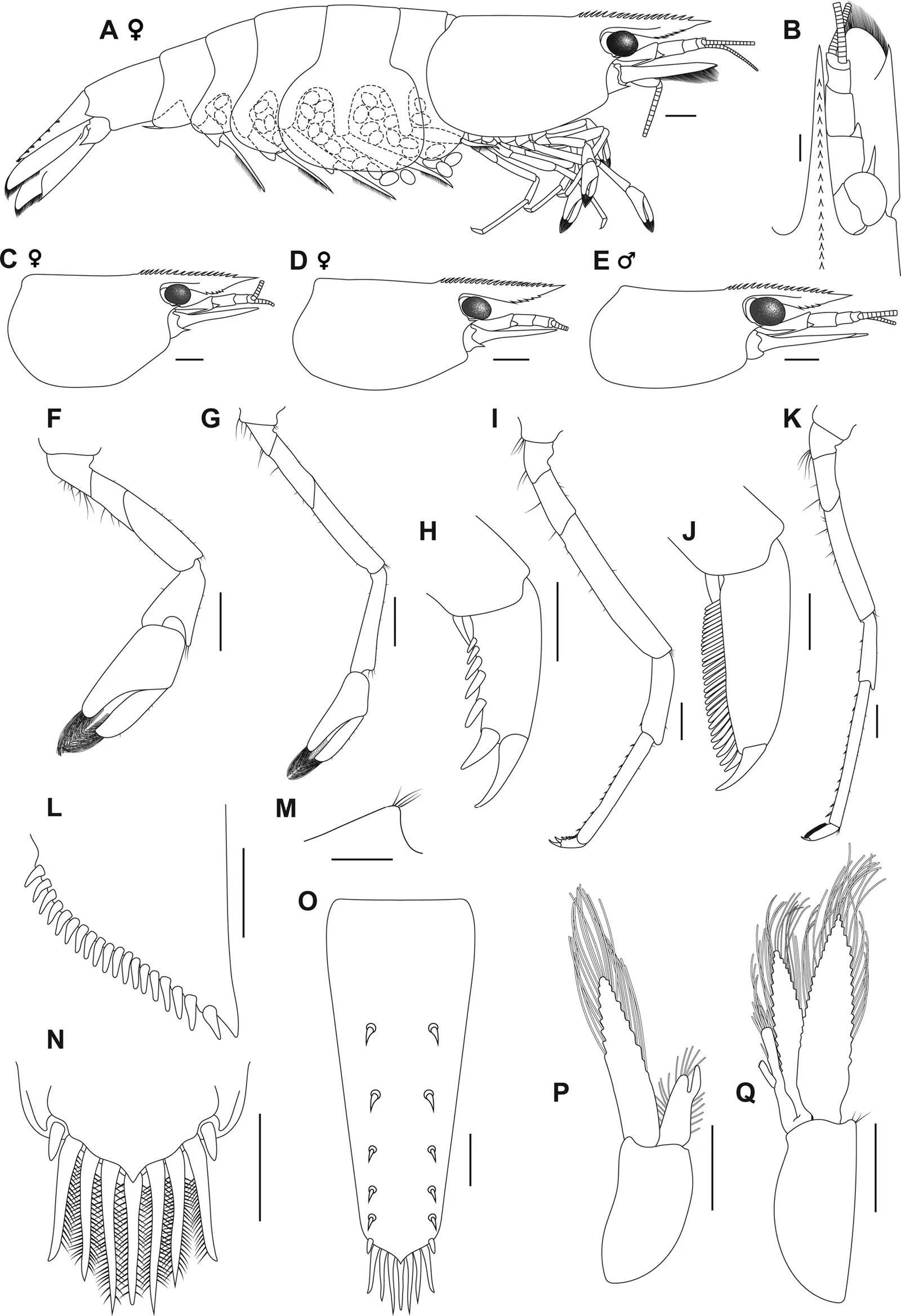
*Caridina lanceifrons*. **A**. Ovigerous female; **B**. Top view of carapace; C, **D**. Cephalothorax and cephalic appendages of female; **E**. Cephalothorax and cephalic appendages of male; **F**. First pereopod; **G**. Second pereopod; **H**. Dactylus of third pereopod; **I**. Third pereopod; **J**. Dactylus of fifth pereopod; **K**. Fifth pereopod; **L**. Uropodal diaeresis; **M**. Preanal carina; **N**. Distal end of telson; **O**. Telson; **P**. First pleopod; **Q**. Second pleopod. Drawings were made of MUMNH-CAR411-D1 (**A, B, F–O**); MUMNH-CAR411-D2 (**C**); MUMNH-CAR503-F1 (**D**); MUMNH-CAR411-M1 (**E, P, Q**). Scale bars: 1 mm (**A–E**); 0.5 mm (**F, G, I–K, P, Q**); 0.25 mm (**L–N**); 0.1 mm (**H, J**).

***Cephalothorax and cephalic appendage*** (*n* = 10). cl 3.63–4.78 (median 4.08; Fig. 13A, C, D) mm, width 2.78–3.90 (median 3.25) mm. Rostrum pointed straight (Fig. 13A–D), slightly to moderately wider at the 1/2 length and abruptly tapering toward the tip, usually reaching slightly beyond the end of third segment of antennular peduncle (Fig. 13B), 0.62–0.80 (median 0.72) as long as cl. Rostral formula: (3–5) 12–21/ 3–6, dorsal teeth usually placed throughout dorsal margin. Antennal spine placed slightly below inferior orbital angle. Pterygostomian margin round. Eye well developed, anterior end reaching to 0.45–0.67 (median 0.55) of first segment of antennular peduncle. Antennular peduncle 0.55–0.76 (median 0.67; Fig. 13A–D) as long as cl, first segment 1.98–2.70 (median 2.30) × as long as second segment, second segment 1.39–2.01 (median 1.59) × as long as third segment. Tooth on distolateral margin of first segment of antennular peduncle well developed. Stylocerite reaching to 0.62–0.96 (median 0.76) of first segment of antennular peduncle. Scaphocerite 2.93–3.21 (median 3.05) × as long as wide, distal margin with short plumose setae.

***Branchial and mouthparts*** (*n* = 3). Podobranch on second maxilliped well developed. Third maxilliped possessed one small and one large arthrobranchs. Pleurobranchs present on all pereopods. Third maxilliped reaching to end of second segment of antennular peduncle; ultimate segment of endopod ending with one large claw and four or five spiniform setae on distal 1/3 of posterior margin, 0.71–0.89 (median 0.82, *n* = 10) as long as penultimate segment.

***Pereopods*** (*n* = 10). Epipod well developed on first four pereopods. Chelae of P1 and P2 well developed.

P1 short (Fig. 13F); chela 2.14–2.52 (median 2.23) × as long as wide, 0.95–1.69 (median 1.39) × as long as carpus, tips of fingers rounded, with tuft of setae near tip; dactylus 1.03–1.79 (median 1.29) × as long as palm; carpus very stout, excavated distally, 1.57–2.55 (median 2.05) × as long as wide, 0.75–1.11 (median 0.91) × as long as merus; merus 2.36–4.78 (median 2.94) × as long as wide, 1.60–2.14 (median 1.79) × as long as ischium.

P2 slenderer, longer than P1 (Fig. 13G); chela 2.23–3.04 (median 2.75) × as long as wide, 0.78–1.04 (median 0.86) × as long as carpus, tips of fingers rounded, with tuft of setae near tip; dactylus 0.84–1.97 (median 1.21) × as long as palm; carpus slender, 4.75–6.30 (median 5.78) × as long as wide, 0.95–1.96 (median 1.24) × as long as merus; merus 3.27–5.66 (median 4.88) × as long as wide, 1.20–1.80 (median 1.52) × as long as ischium.

P3 not sexually dimorphic (Fig. 13I); dactylus with 5–7 (mode 6) spiniform setae on flexor margin (Fig. 13H), 2.37–3.71 (median 2.90) × as long as wide (including terminal claw), terminating with one large claw; propodus bearing numerous spiniform setae on posterior margin, 8.12–10.80 (median 8.77) × as long as wide, 3.75–5.95 (median 4.95) × as long as dactylus; carpus possessed 3–5 (mode 4) setae on posterior margin of outer surface, the distal seta largest, the other setae minute, 3.98–6.16 (median 4.43) × as long as wide, 0.47–0.68 (median 0.64) as long as propodus; merus possessed 2–5 (mode 4) large setae on posterior margin of outer surface, 5.32–7.32 (median 6.40) × as long as wide, 1.86–2.40 (median 2.03) × as long as carpus; ischium with or without one spiniform seta.

P5 slender (Fig. 13K); dactylus with 26–34 spiniform setae on flexor margin (Fig. 13J), 2.70–3.44 (median 2.79) × as long as wide (including terminal claw), terminating with one large claw; propodus bearing numerous spiniform setae on posterior margin, 9.84–14.17 (median 12.12) × as long as wide, 4.47–5.35 (median 4.74) × as long as dactylus; carpus possessed 3–5 (mode 3) spiniform setae on posterior margin of outer surface, the distal seta largest, the other setae minute, 4.39–5.34 (median 4.81) × as long as wide, 0.47–0.56 (median 0.51) as long as propodus; merus possessed one or two (mode 2) large setae on posterior margin of outer surface, 5.07–6.58 (median 5.65) × as long as wide, 1.46–2.05 (median 1.52) × as long as carpus; ischium without spiniform seta.

***Male pleopods*** (*n* = 10). Pl1 endopod subtriangular (Fig. 13P), wider proximally, 1.98–2.95 (median 2.23) × as long as wide, 0.30–0.42 (median 0.38) of exopod length, without (very rare in population) or with appendix interna, emerging from distal 1/3 end of endopod. Pl2 appendix masculina rod-shaped (Fig. 13Q), slender, with many setae, 0.56–0.64 (median 0.61) × as long as endopod; appendix interna reaching 0.71–0.84 (median 0.80) of appendix masculina length.

***Abdomen*** (*n* = 10). Sixth abdominal somite ~ 0.46–0.58 (median 0.51) of cl, 1.34–1.66 (median 1.51) × as long as fifth somite, 0.79–0.92 (median 0.85) × as long as telson (Fig. 13A). Telson 2.64–3.53 (median 3.13; Fig. 13O) × as long as wide, with 4–6 pairs (mode 5) of dorsal spiniform setae and one pair of dorsolateral spiniform setae; distal margin subtriangular without or with a tiny posteromedian projection, 7–10 moveable plumose setae, lateral pair longest, intermediate pair shortest (Fig. 13N). Preanal carina subtriangular, with a few setae, without a spine (Fig. 13M). Uropodal diaeresis with 15–19 moveable spiniform setae (Fig. 13L).

***Embryos***. Ovigerous females with 84–208 embryos (mean 121, *n* = 10). Size of embryos with eyes 0.59–0.69 × 0.35–0.44 mm (*n* = 50).

###### Coloration.

Body color variable, either reddish orange or black. Body usually decorated with numerous small dark spots. Rostrum orange to light brown. Cephalic region and abdominal somite furnished with sandy brown stripes. Body darker dorsally than ventrally. Each abdominal somite dorsally decorated with conspicuous light transverse bands. Embryos brown (Fig. 8G, H).

###### Distribution and habitat.

China, Vietnam, and Thailand (Yu 1938; Cai 2014; Do et al. 2021b; this study). This species was first recorded in Thailand by Do et al. (2021b). In the present study, we collected this species throughout southern Thailand (Fig. 5A). Although Yu (1936) reported collecting *C. lanceifrons* from salt water in Hainan Province, we barely found *C. lanceifrons* in coastal areas or brackish water. Instead, this species was commonly encountered in lotic habitats such as streams and waterfalls, where individuals occurred along the banks over gravelly or sandy substrates. Our observation agrees with those of Do et al. (2021b), who also reported that this species does not occur in coastal habitats.

###### Phylogenetic placement.

Our molecular analyses show this species to be monophyletic, although with low support (BS = 51%, BPP = 0.86; Fig. 2). This suggests the need for further phylogenetic investigation by incorporating more genes and an extended range of populations. It forms a clade with *C. thai* and *C. sirindhornae* (BS = 100%, BPP = 1.00).

###### Remarks.

*Caridina lanceifrons* was described by Yu (1936) based on specimens from Hainan Province, China. Later, Cai (2014) designated neotypes because the original type specimen no longer existed. The morphology of *C. lanceifrons* in our collection is well consistent with previous works, in terms of the rostral formula: (3–5) 13–18/ 3–6 vs (4–6) 10–19/ 1–6 (Yu 1936, 1938; Cai 2014; Do et al. 2021b), rostrum length (0.54–0.69× as long as cl vs 0.45–0.96; Do et al. 2021b), P1 chela (2.14–2.52× as long as wide vs 1.89–2.4; Cai 2014; Do et al. 2021b), P1 dactylus (1.03–1.79× as long as palm vs 1.00–1.33; Do et al. 2021b), P1 carpus (1.57–2.55× as long as wide vs 1.22–2.00; Do et al. 2021b), P3 merus (2.36–4.78× as long as wide vs 2.57–3.25; Do et al. 2021b), P2 chela (2.23–3.04× as long as wide vs 2.46–3.29; Yu 1938; Cai 2014; Do et al. 2021b), P2 dactylus (0.84–1.97× as long as wide vs 1.09–1.89; Do et al. 2021b), P2 carpus (4.75–6.30× as long as wide vs 4.0–6.0; Yu 1938; Do et al. 2021b), P2 merus (3.27–5.66× as long as wide vs 4.2–5.25; Do et al. 2021b), P3 dactylus (2.37–3.71× as long as wide vs 2.75–4.00; Yu 1938; Cai 2014; Do et al. 2021b), P3 propodus (8.12–10.80× as long as wide vs 8.0–11.0; Yu 1938; Cai 2014; Do et al. 2021b), P3 carpus (3.98–6.16× as long as wide vs 3.75–5.0; Do et al. 2021b), spiniform setae on P3 dactylus (5–7; Yu 1938; Cai 2014; Do et al. 2021b), P3 merus (5.32–7.32× as long as wide vs 5.83–7.33; Do et al. 2021b), setae on P3 merus (2–5 vs 3–5; Do et al. 2021b), P5 dactylus (2.70–4.23× as long as wide vs 2.33–3.6; Cai 2014; Do et al. 2021b), P5 propodus (9.84–14.17× as long as wide vs 12.0–14.0; Yu 1938; Cai 2014; Do et al. 2021b), P5 carpus (4.39–5.34× as long as wide vs 4.33–6.25; Do et al. 2021b), spiniform setae on P5 dactylus (26–34 vs 22–38; Yu 1938; Cai 2014; Do et al. 2021b), P5 merus (5.07–6.58× as long as wide vs 5.33–6.73; Do et al. 2021b), telson (2.64–3.53× as long as wide vs 2.33–3.85; Do et al. 2021b), dorsal spiniform setae on telson (4–5 pairs vs 5–6 pairs; Yu 1936, 1938; Cai 2014; Do et al. 2021b), plumose setae on distal margin of telson (3–4 pairs vs 4–5 pairs; Do et al. 2021b), spiniform setae on uropodal diaeresis (15–19 vs 14–22; Cai 2014; Do et al. 2021b). The embryo size of *C. lanceifrons* in this study was significantly smaller than previously reported (0.60–0.71 × 0.33–0.44 mm vs 0.74–1.05 × 0.50–0.70 mm; Yu 1938; Cai 2014; Do et al. 2021b).

Some *C. lanceifrons* specimens appear very similar to *C. sumatrensis* in terms of the overall body shape and size, and color pattern. However, they can be distinguished by using rostrum characters. The rostrum of *C. lanceifrons* is quite straight, wider near the 1/2-length, then abruptly tapering at the tip. In contrast, the rostrum of *C. sumatrensis* is slightly directed downward, with a similar width throughout the rostrum. *Caridina lanceifrons* also differs from *C. sumatrensis* by possessing fewer spiniform setae on P5 dactylus (26–34 vs 34–47), and stouter P5 dactylus (2.70–3.44× as long as wide vs 3.48–4.64). Moreover, the embryo size of *C. lanceifrons* is much larger than that of *C. sumatrensis* (0.59–0.69 × 0.35–0.44 mm vs 0.38–0.52 × 0.23–0.34 mm).

##### Caridina thai

Taxon classificationAnimaliaDecapodaAtyidae

Cai & Naiyanetr, 2024

B4AE7AD7-52E3-5DDB-9D60-88F73F2CE792

Figs 5A, 14A

Caridina thai Cai & Naiyanetr, 2024: 786, figs 6, 7. Type locality Muak Lek Waterfall, Muak Lek District, Saraburi Province, Thailand.

###### Material examined.

One lot (topotype; see Suppl. material 1: Data S1).

**Figure 14.**
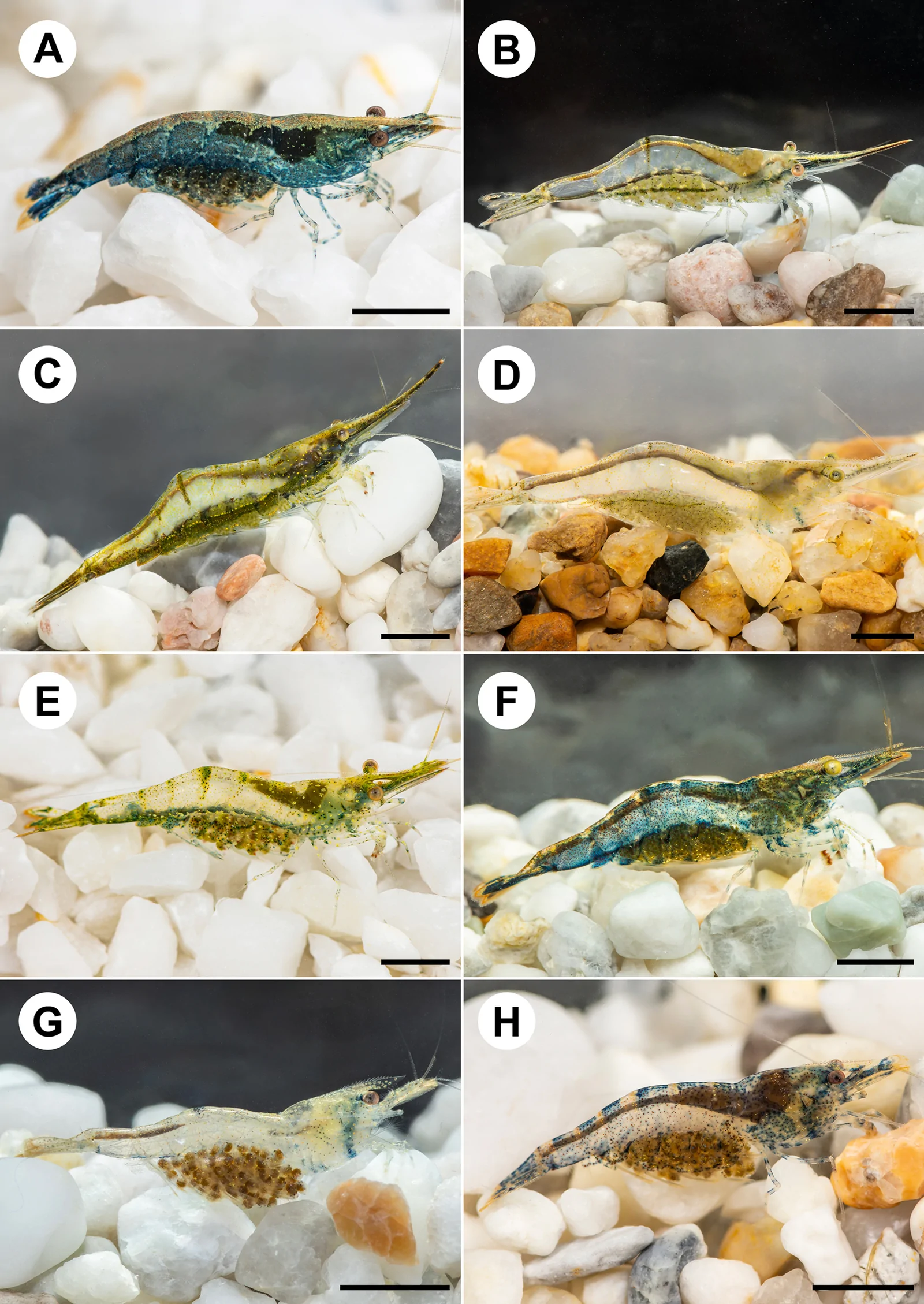
Living specimens of *Caridina* species. **A**. *C. thai*, topotype MUMNH-CAR861-F1 from Nakhon Ratchasima Province, Pak Chong District, Muak Lek Waterfall; **B, C**. *C. gracillima*, specimens; **B**. MUMNH-CAR626-P1 from Trang Province, Mueang District, Trang River; **C**. MUMNH-CAR631-P1 from Krabi Province, Mueang District, Tha Hindarn Stream; **D**. *C. gracilipes*, specimen MUMNH-CAR462-P1 from Nakhon Si Thammarat Province, Khanom District, Khao Phlai Dam; **E**. *C. macrophora*, specimen MUMNH-CAR800 from Nakhon Si Thammarat Province, Bang Khan District, Lam Dang Stream; **F**. *C. peninsularis*, specimen MUMNH-CAR633-P1 from Phang Nga Province, Khum Mut Stream; **G**. *C. excavatoides*, specimen MUMNH-CAR616-F2 from Nakhon Sri Thammarat Province, Thung Song District, Tha-Luang Stream; **H**. *C. temasek*, specimen MUMNH-CAR835-P1 from Pattani Province, Mueang District, Pattani River. Scale bar: 4 mm.

###### Distribution and habitat.

Thailand. Cai and Naiyanetr (2024) described *C. thai* from specimens collected from Muak Lek Waterfall, Saraburi Province in Central Thailand. Their examined materials also included two lots from southern Thailand (Chumphon and Nakhon Sri Thammarat provinces; Fig. 5A). We have visited these localities, but we were unable to obtain *C. thai* in our surveys. Based on Cai and Naiyanetr (2024), this species is a stream specialist. They only live in lotic habitats, such as fast-flowing streams and waterfalls.

###### Remarks.

The present phylogenetic tree includes two topotype sequences of *C. thai* presently obtained by us in this study. Our molecular phylogeny indicates that *C. thai* belongs to the *C. lanceifrons* species group. It is placed as the sister species to *C. lanceifrons*, although with poorly nodal support. While these two species are very similar, *C. thai* differs from *C. lanceifrons* by possessing a greater number of spiniform setae on the P5 dactylus (43 vs 26–34).

#### *Caridina gracilirostris* species group

**Diagnosis**. Rostrum very long, at least 1.5× as long as cl, strongly curved upward, with few dorsal teeth (< 10) and numerous ventral teeth (usually > 20). Subapical teeth usually present. Antennular peduncle long, > 0.7× of cl. Walking leg elongated. Dorsal hump at third abdominal somite. Sixth abdominal somite longer than 1/2 of cl. Epipods well developed on P1–P4. Telson very slender, > 0.8× as long as cl, with short plumose setae. Uropodal diaeresis usually with < 10 moveable spiniform setae. Endopod of male first pleopod with or without appendix interna.

**Remarks**. The *Caridina gracilirostris* species group was first proposed by Cai and Ng (2007), and its diagnostic characters were further refined by de Mazancourt et al. (2020), whose diagnosis is consistent with the present study. These diagnostic characters include a very long and upcurved rostrum with few dorsal teeth, a long antennular peduncle, slender segments of walking legs, a typical dorsal hump over the third abdominal somite, a long sixth abdominal somite, few spiniform setae on uropodal diaeresis, endopod of male first pleopod with or without appendix interna, and few setae on the distal end of telson. The phylogenetic tree in this study recovered four species as monophyletic with maximum nodal supports (BS = 100%, BPP= 1.00; Fig. 3). *Caridina neglecta* Cai & Ng, 2007 was retrieved as the basal clade of the species group, followed by *C. gracilirostris*, and then a clade of *C. wilsoni* Klotz, von Rintelen & von Rintelen, 2024 and *C. gracillima*. The latter three species are highly similar morphologically (see remarks in *C. gracillima*). *Caridina gracilirostris* and *C. gracillima* were previously reported from southern Thailand by Cai and Naiyanetr (2024). However, in our intensive survey throughout the region we obtained only *C. gracillima*.

Recently, Cai (2026) established the new genus *Gracidina* Cai, 2026, to accommodate members of the *Caridina gracilirostris* species group. Although this group represents a well-supported monophyletic clade, and the genus *Caridina* s.l. is widely recognized as polyphyletic and in need of a comprehensive systematic revision (e.g., von Rintelen et al. 2012; de Mazancourt et al. 2019a), recognizing *Gracidina* alone leaves the remaining allied *Caridina* taxa paraphyletic, while the genus as a whole remains polyphyletic (de Mazancourt et al. 2019a, 2020, 2023b; this study). Consequently, this taxonomic change does not resolve the broader systematic issues within *Caridina*. We therefore prefer to retain these taxa within *Caridina* pending a comprehensive generic revision based on robust comparative morphological and phylogenomic evidence.

##### Caridina gracilirostris

Taxon classificationAnimaliaDecapodaAtyidae

De Man, 1892

62FA0673-CB97-51FB-8AB1-BE78A066AA09

Fig. 5C

Caridina gracilirostris De Man, 1892: 399–404 (in part), pl. 25, fig. 31a–c. Type locality “Celebes, aus dem Flusse bei Maros” [= river near Maros, Sulawesi, Indonesia]. De Man (1892) collected specimens from various locations in Sulawesi, Indonesia. Cai and Ng (2007) subsequently designated a lectotype and paralectotypes using materials collected from the river near Maros, Sulawesi, Indonesia.
Caridina gracilirostris — Kemp 1918b: 282; Blanco 1935: 32, figs 11–17; Riek 1953: 121; Holthuis 1965: 23 (in part); Holthuis 1978: 35 (in part); Johnson 1961a: 124, figs 1, 2; Richard and Chandran 1994: 242; Chace 1997: 10 (in part); Jalihal and Shenoy 1998: 128; Cai and Ng 2001: 674, fig. 7; Naiyanetr 2007: 34; de Mazancourt et al. 2019a: 166, 169–170; de Mazancourt et al. 2020: 12, figs 2e, 4, 25a; Cai and Naiyanetr 2024: 773.
Caridina pseudogracilirostris Thomas, Pillai & Pillai, 1973: 871, fig. 1. Type locality “Cochin Backwater” [= Kochi Backwaters, Kerala, India].

###### Material examined.

None.

###### Distribution and habitat.

Indonesia, Malaysia, Thailand, Cambodia, Singapore, Taiwan, Japan, Philippines, Fiji, Palau, India, and Madagascar (Cai and Ng 2007; de Mazancourt et al. 2020; Cai and Naiyanetr 2024).

###### Remarks.

*Caridina gracilirostris* was reported from several regions in Thailand, including the Chao Phraya and Mae Klong basins, as well as several drainages emptying into the Andaman Sea on the western side of southern Thailand (Cai and Naiyanetr 2024). Despite a report from southern Thailand (Cai and Naiyanetr 2024), no specimens were collected during the extensive surveys in the present study.

##### Caridina gracillima

Taxon classificationAnimaliaDecapodaAtyidae

Lanchester, 1902

0EAEE974-27A6-5D2D-A10D-582FA66696DE

**Figure 15.**
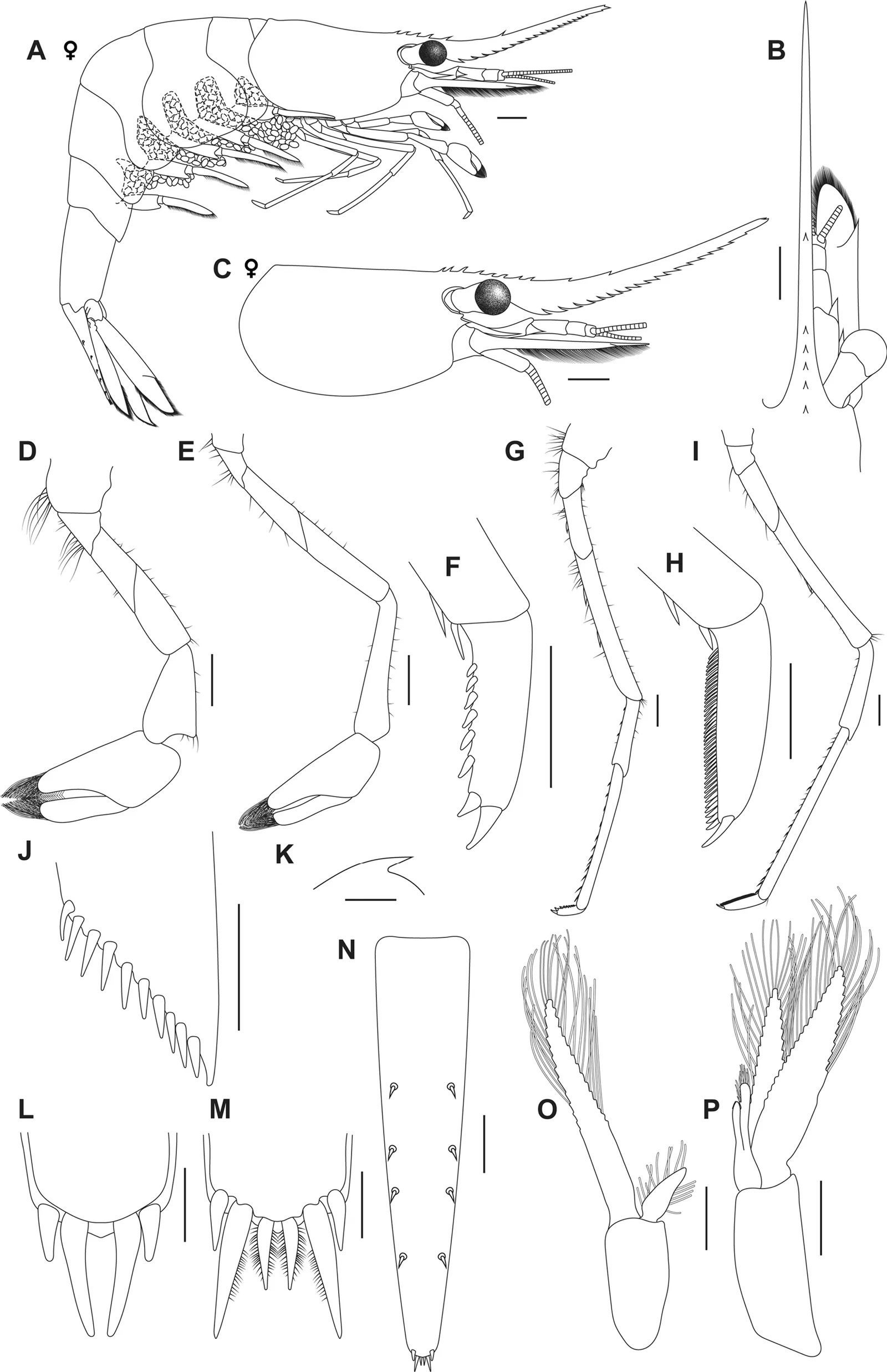
*Caridina gracillima*. **A**. Ovigerous female; **B**. Top view of carapace; **C**. Cephalothorax and cephalic appendages of female; **D**. First pereopod; **E**. Second pereopod; **F**. Dactylus of third pereopod; **G**. Third pereopod; **H**. Dactylus of fifth pereopod; **I**. Fifth pereopod; **J**. Uropodal diaeresis; **K**. Preanal carina; **L–M**. Distal end of telson; **N**. Telson; **O**. First pleopod; **P**. Second pleopod. Drawings were made of MUMNH-CAR497-F1 (**A, B, D–K, M, N**); MUMNH-CAR431-D1 (**C, L**); MUMNH-CAR497-M1 (**O, P**). Scale bars: 1 mm (**A–C**); 0.5 mm (**D, E, G, I, N–P**); 0.25 mm (**F, H, J–M**).

Figs 5C, 14B, 14C, 15

Caridina gracillima Lanchester, 1902: 560, pl. 34, fig. 1. Type locality “Inland Sea near Singora” [= Songkhla Lake system, southern Thailand].
Caridina gracillima — Bouvier 1905: 72, pl. 39; Kemp 1918b: 285.
Caridina *gracilima* [sic] — Cai and Ng 2007: 1592, fig. 3; Cai and Naiyanetr 2024: 777.
Caridina gracilirostris [non De Man, 1892] — Johnson 1961a: 124 (in part).

###### Material examined.

49 lots (see Suppl. material 1: Data S1).

###### Description.

***Cephalothorax and cephalic appendage*** (*n* = 10). cl 3.37–4.57 (median 4.02; Fig. 15A, C) mm, width 2.50–3.16 (median 2.81) mm. Rostrum very long and strongly curved upward (Fig. 15A, C), ending in bifid, reaching beyond end of the third segment of antennular peduncle (Fig. 15B), 1.56–2.13 (median 1.76) × as long as cl. Rostral formula: (0–2) 6–11 + 1–4/ 19–34, subapical teeth always present. Antennal spine placed below inferior orbital angle. Pterygostomian margin subrectangular. Eye well developed, anterior end reaching to 0.47–0.82 (median 0.67) of first segment of antennular peduncle. Antennular peduncle 0.72–0.89 (median 0.78; Fig. 15A–C) as long as cl, first segment 1.73–2.46 (median 2.09) × as long as second segment, second segment 1.20–1.57 (median 1.43) × as long as third segment. Tooth on distolateral margin of first segment of antennular peduncle acute and well developed. Stylocerite reaching to 0.74–0.90 (median 0.79) of first segment of antennular peduncle. Scaphocerite 3.20–4.14 (median 3.74) × as long as wide, distal margin with short plumose setae.

***Branchial and mouthparts*** (*n* = 3). Podobranch on second maxilliped well developed. Third maxilliped possessed one small and one large arthrobranch. Pleurobranchs present on all pereopods. Third maxilliped reaching to end of second segment of antennular peduncle; ultimate segment of endopod ending with one large claw and seven to ten spiniform setae on distal 1/3 of posterior margin, 0.63–0.83 (median 0.73, *n* = *10*) as long as penultimate segment.

***Pereopods*** (*n* = 10). Epipod well developed on first four pereopods. Chelae of P1 and P2 well developed.

P1 short (Fig. 15D); chela of first pereopod 1.88–2.16 (median 2.00) × as long as wide, 1.37–1.80 (median 1.51) × as long as carpus, tips of fingers rounded, with tuft of setae near tip; dactylus 1.06–1.58 (median 1.20) × as long as palm; carpus stout, excavated distally, 1.49–2.00 (median 1.57) × as long as wide, 0.98–1.16 (median 1.07) × as long as merus; merus 2.14–2.57 (median 2.30) × as long as wide, 1.00–1.38 (median 1.14) × as long as ischium.

P2 slenderer and longer than P1 (Fig. 15E); chela of second pereopod 1.91–2.54 (median 2.16) × as long as wide, 0.79–0.98 (median 0.88) as long as carpus, tips of fingers rounded, with tufts of setae near tip; dactylus 1.13–1.58 (median 1.23) × as long as palm; carpus slender, 3.53–4.70 (median 4.10) × as long as wide, 1.01–1.33 (median 1.25) × as long as merus; merus 3.75–4.69 (median 4.14) × as long as wide, 1.05–1.52 (median 1.27) × as long as ischium.

P3 not sexually dimorphic (Fig. 15G); dactylus with 6–8 (mode 7) spiniform setae on flexor margin (Fig. 15F), 2.75–3.82 (median 3.53) × as long as wide (including terminal claw), terminating with one large claw; propodus bearing numerous spiniform setae on posterior margin, 12.36–15.20 (median 13.86) × as long as wide, 4.49–5.77 (median 4.90) × as long as dactylus; carpus possessed 2–4 (mode 3) setae on posterior margin of outer surface, the distal seta largest, the other setae minute, 5.49–6.89 (median 6.06) × as long as wide, 0.50–0.63 (median 0.54) as long as propodus; merus possessed three or four (mode 3) large setae on posterior margin of outer surface, 8.61–10.72 (median 9.26) × as long as wide, 1.87–2.50 (median 2.19) × as long as carpus; ischium with one spiniform seta.

P5 slender (Fig. 15I); dactylus with 35–48 spiniform setae on flexor margin (Fig. 15H), 3.15–4.08 (median 3.61) × as long as wide (including terminal claw), terminating with one large claw; propodus bearing numerous spiniform setae on posterior margin, 13.64–18.69 (median 16.23) × as long as wide, 3.83–5.08 (median 4.43) × as long as dactylus; carpus possessed 1–4 setae on posterior margin of outer surface, the distal seta largest, the other setae minute, 4.45–6.82 (median 5.74) × as long as wide, 0.41–0.53 (median 0.46) as long as propodus; merus possessed two large setae on posterior margin of outer surface, 8.31–9.59 (median 9.04) × as long as wide, 1.52–1.94 (median 1.83)× as long as carpus; ischium without spiniform seta.

***Male pleopods*** (*n* = 10). Pl1 endopod slender (Fig. 15O), wider proximally, 2.31–3.31 (median 2.46) × as long as wide, 0.25–0.30 (median 0.27) of exopod length, without appendix interna. Pl2 appendix masculina rod-shaped with many setae (Fig. 15P), 0.49–0.57 (median 0.54) as long as endopod; appendix interna very slender, reaching 0.77–0.92 (median 0.81) of appendix masculina length.

***Abdomen*** (*n* = 10). Sixth abdominal somite ~ 0.80–0.96 (median 0.90) of cl, 1.80–2.23 (median 1.95) × as long as fifth somite, 0.80–1.09 (median 0.95) × as long as telson (Fig. 15A). Telson very slender, 4.43–5.02 (median 4.69; Fig. 15N) × as long as wide, with 3–5 (mode 4) pairs of dorsal spiniform setae and one pair of dorsolateral spiniform setae; distal margin round without posteromedian projection and 2–6 (mode 4) moveable plumose setae, the lateral pair longer than the median pair (Fig. 15L, M). Preanal carina large, with a spine (Fig. 15K) or without a spine (rarely found). Uropodal diaeresis slender, with 6–10 moveable spiniform setae (Fig. 15J).

***Embryos***. Ovigerous females with 300–799 embryos (mean 504, *n* = 10). Size of embryos with eyes 0.35–0.43 × 0.21–0.28 mm (*n* = 50).

###### Coloration.

Body rather translucent, usually with a lateral thick purple brown or brown line running from cephalic region to the end of abdomen. Rostrum gold to medium brown. Cephalic region furnished with a few small yellow spots. Embryos yellowish orange to gold (Fig. 14B, C).

###### Distribution and habitat.

southern Thailand and Malaysia (Sabah) (Klotz et al. 2024; this study). *Caridina gracillima* is widespread throughout southern Thailand (Fig. 5C). This species inhabits various habitats such as lowland rivers, reservoirs, lakes, and estuaries. The shrimps are found living on aquatic vegetation along the banks.

###### Phylogenetic placement.

The molecular analyses recovered *C. gracillima* as monophyletic (BS = 100%, BPP = 1.00; Fig. 3) and placed it as a sister to *C. wilsoni* (BS = 94%, BPP = 0.98).

###### Remarks.

Lanchester (1902) mentioned the type locality of *C. gracillima* as either “Tale Sap” or “Thale Nawi”. That author further specified that “Tale Sap” was an “inland sea located just north of the town of Singora” [= area recognized as the present day Mueang District, Songkhla Province, Thailand] and that of “Thale Nawi” was a lake at its head. Based on this historical demonstration, “Tale Sap” is definitively identified as Songkhla Lake, while “Thale Nawi” represents an alternative romanization of Thale Noi, a protected freshwater wetland complex situated immediately north of the Songkhla Lake. Since the label can only narrow the collecting area to these two water bodies, the precise type locality cannot be further resolved. Consequently, the definitive type locality for this specimen should be designated as the general area, the entire Songkhla Lake system (including Thale Sap and Thale Noi areas; Fig. 1).

*Caridina gracillima* is distinct from other Thai *Caridina* species in having a very long and strongly upcurved rostrum. The morphology of *C. gracillima* observed in this study is congruent with previous investigations in terms of the shape of rostrum, dorsal teeth (6–11 vs 5–10; Lanchester 1902; Kemp 1918b; Cai and Ng 2007), scaphocerite (3.20–4.14× as long as wide vs 3.5–4; Kemp 1918b; Cai and Ng 2007), P1 chela (1.88–2.16× as long as wide vs 2.2; Cai and Ng 2007), P1 carpus (1.49–2.00× as long as wide vs 1.6; Cai and Ng 2007), P2 carpus (3.53–4.70× as long as wide vs 4.2; Cai and Ng 2007), spiniform setae on P3 dactylus (6–8 vs 6–9; Lanchester 1902; Cai and Ng 2007), P3 dactylus (2.75–3.82× as long as wide vs 3.5; Cai and Ng 2007), spiniform setae on P5 dactylus (35–48 vs 30–47; Lanchester 1902; Cai and Ng 2007), P5 dactylus (3.15–4.08× as long as wide vs 4; Cai and Ng 2007), sixth abdominal somite (0.80–0.96× as long as cl vs 0.8; Cai and Ng 2007), sixth abdominal somite (1.80–2.23× as long as the fifth abdominal somite vs 1.9; Cai and Ng 2007), spiniform setae on uropodal diaeresis (6–10 vs 6–10; Kemp 1918b; Cai and Ng 2007).

However, our detailed examination of topotypes and other material from southern Thailand revealed some different morphological traits that contradict with the previous studies. Most notably, the number of ventral rostral teeth in our *C. gracillima* specimens is significantly higher (19–34 vs 12–24; Lanchester 1902; Kemp 1918b; Cai and Ng 2007). Most of *C. gracillima* specimens in the present study possesses a distinctive spine on its preanal carina, with very rare cases lacking a spine (vs always lacking a spine in previous reports; Cai and Ng 2007; Klotz et al. 2024). Moreover, the embryo size of our specimens is much smaller compared to previous reports (0.35–0.43 × 0.21–0.28 mm vs 0.55–0.70 × 0.35–0.45 mm; Kemp 1918b; Cai and Ng 2007), particularly when compared to the lectotype (vs 0.58 × 0.42 mm; Cai and Ng 2007).

*Caridina gracillima* is very similar to *C. gracilirostris*. Unfortunately, we failed to obtain specimens of *C. gracilirostris* to compare their morphology. Previous documents distinguish *C. gracillima* from *C. gracilirostris* based on several key characters, including a shorter rostrum, fewer ventral teeth, fewer spiniform setae on uropodal diaeresis, absence of spine on preanal carina, and slightly larger embryos (Lanchester 1902; Kemp 1918b; Bouvier 1925; Cai and Ng 2007). However, our result suggests that these characters overlap considerably between *C. gracillima* and *C. gracilirostris*, indicating that the two species cannot be reliably separated using the currently established morphological criteria. Furthermore, the similarity between *C. gracillima* and *C. gracilirostris* is also found in other characters, including the rostral formula: (0–2) + 6–10 + 1–4 / 19–34 vs (0–1) + 5–9 + 1–2/ 21–35 (de Mazancourt et al. 2020), rostrum length (1.56–2.13× as long as cl vs 1.3–2.6; Cai and Ng 2007; de Mazancourt et al. 2020), P1 chela (1.88–2.16× as long as wide vs 1.9–2.9; de Mazancourt et al. 2020), P1 dactylus (1.06–1.58× as long as wide vs 0.7–1.3; de Mazancourt et al. 2020), P1 carpus (1.49–2.00× as long as wide vs 1.5–1.9; de Mazancourt et al. 2020), P2 chela (1.91–2.54× as long as wide vs 2.0–2.5; de Mazancourt et al. 2020), P2 dactylus (1.13–1.58× as long as wide vs 1.1–1.8; de Mazancourt et al. 2020), P2 carpus (3.53–4.70× as long as wide vs 3.2–5.1; de Mazancourt et al. 2020), spiniform setae on P3 dactylus (6–8 vs 7–10; de Mazancourt et al. 2020), P3 dactylus (2.75–3.82× as long as wide vs 3.3–3.8; de Mazancourt et al. 2020), P5 dactylus (3.15–4.08× as long as wide vs 4.0–4.4; de Mazancourt et al. 2020), spiniform setae on P5 dactylus (35–48 vs 26–47; Cai and Ng 2007; de Mazancourt et al. 2020), spiniform setae on uropodal diaeresis (6–10 vs 6–11; Cai and Ng 2007; de Mazancourt et al. 2020), and embryo size (0.35–0.43 × 0.20–0.28 mm vs 0.34–0.43 × 0.19–0.26 mm; de Mazancourt et al. 2020).

The molecular analysis in the present study confirmed that *C. gracillima* and *C. gracilirostris* (Fig. 3) are distinct species, a conclusion reinforced by their allopatric distribution. *Caridina gracillima* is restricted to mainland Southeast Asia, whereas *C. gracilirostris* is widely distributed across the Indo–Pacific region (Cai and Ng 2007; de Mazancourt et al. 2020). An additional comparative morphology study is still essential to fully resolve and define their species boundaries.

*Caridina gracillima* is almost identical to *C. wilsoni* in terms of the rostrum shape, rostral formula: (0–2) + 6–11 + 1–4/ 19–34 vs (0–1) + 6–9 + 1/ 19–35, and the proportion in appendage segments (Klotz et al. 2024). This morphological similarity was reflected in the phylogenetic results, which placed them together as a sister clade with high support (BS = 100%, BPP= 1.00; Fig. 3). However, *C. gracillima* differs from *C. wilsoni* in having more spiniform setae on the P5 dactylus (35–48 vs 21–35), and a smaller embryo size (0.35–0.43 × 0.21–0.28 mm vs 0.59–0.66 × 0.34–0.43; Klotz et al. 2024).

#### *Caridina nilotica* species group

**Diagnosis**. Rostrum long, straight or slightly to moderately curved upward, frequently extended beyond the end of third segment of antennular peduncle. Antennular peduncle long, > 0.7× as long as cl. Dorsal humped at third abdominal somite. Epipods well developed on P1–P4. Sixth abdominal somite ~ 0.6–0.8× as long as cl. Uropodal diaeresis usually with < 15 spiniform setae. Endopod of male Pl1 subtriangular with or without appendix interna.

**Remarks**. Bouvier (1905) was the first to establish the *C. nilotica* species group. Subsequently, both the defining characters and the species composition of the *C. nilotica* species group have been refined extensively by numerous authors (Bouvier 1925; Johnson 1963; Jalihal et al. 1984; Richard and Clark 2005, 2014; de Mazancourt et al. 2018a, 2019a, 2020). In agreement with previous studies, we confirm that the diagnostic characters of *C. nilotica* species group, specifically the rostrum length, sixth abdominal somite length, number of moveable spiniform setae on uropodal diaeresis, endopod of male first pleopod, and the dorsal hump on the third abdominal somite, are all shared characters for the group (Bouvier 1905, 1925; Jalihal et al. 1984; de Mazancourt et al. 2020). Comparative morphology of this group has been well investigated by several authors (i.e., Richard and Clark 2005, 2010, 2014; Cai 2014; de Mazancourt et al. 2018a).

The *C. nilotica* species group was recovered as a monophyletic group in this study (BS = 99%, BPP = 1.00; Fig. 3) and other recent phylogenetic analyses (de Mazancourt et al. 2019a, 2020). *Caridina macrophora* was recovered as a basal clade here. The remaining taxa were roughly divided into two subclades, although with moderate nodal supports: (1) a clade of *C. appendiculata* Jalihal & Shenoy, 1998, *C. meridionalis* Roux, 1926b, *C. brachydactyla* De Man, 1908b, and *C. gracilipes* (BS = 69%, BPP = 0.92) and (2) a clade of *C. grandirostris* Stimpson, 1860, *C. elongapoda* Liang & Yan, 1977, *C. leucosticta* Stimpson, 1860, *C. simoni* Bouvier, 1904, and *C. peninsularis* (BS = 56%, BPP = 0.86). No clear shared characters were detected for each subclade.

Our phylogenetic tree further showed that the *C. nilotica* and *C. gracilirostris* species groups were sister groups with high nodal supports (BS = 94%, BPP = 1.00; Fig. 3). They consistently share some morphological characters, comprising an elongated curved-up rostrum, long antennular peduncle, epipods present on the first four pereopods, and a small number of spiniform setae on uropodal diaeresis.

Three species of the *C. nilotica* species group, consisting of *C. gracilipes*, *C. macrophora*, and *C. peninsularis*, were collected from southern Thailand in this study.

##### Caridina gracilipes

Taxon classificationAnimaliaDecapodaAtyidae

De Man, 1892

A1F06A39-1B5F-59A9-9F72-7E4A4DDF7FAD

Figs 5D, 14D, 16

Caridina *Wyckii* var. gracilipes De Man, 1892: 387, pl. 24 figs 29–29e. Type locality “Celebes, aus dem Flusse von Maros” [= river near Maros, Sulawesi]; “Celebes, Makassar, im Wasser eines Reisfeldes” [= Makassar, Sulawesi]; “Celebes, aus einem kleinen, mit dem Meere nicht in Verbindung stehenden Bache zu Balangnipa” [= small stream near Balangnipa, which is not connected with the ocean, Sulawesi]; “Celebes, Palima, aus dem Tjenrana, in Brackwasser” [= Cenrana River, Palima, Sulawesi]; “Celebes, Pampanua, aus dem Tjenrana” [= Cenrana River, Pampanua, Sulawesi]; “Saleyer, aus dem Flusse Bonéa" [= Bonea River, Selayar].
Caridina wycki var. gracilipes — Bouvier 1904: 130; Schenkel 1902: 498.
Caridina nilotica gracilipes — De Man 1908b: 270, fig. 7a, b; Kemp 1918b: 275; Yu 1936: 88; Yu 1938: 277, figs 1, 2; Ueno 1935: 272, fig. 2; Yu 1974: 52, figs 2, 3; Liang and Zheng 1988: 15; Liang and Zhou 1993: 231; Liang 2004: 198 (in part).
Caridina gracilipes — Wowor et al. 2004: 341, fig. 6c, d; Cai and Shokita 2006b: 250; Mariappan and Richard 2006: 9, figs 5–7; Cai et al. 2007: 284; Cai 2014: 208, figs 1–3; Richard and Clark 2014: 310, figs 4, 5; de Mazancourt et al. 2018a: 1438, fig. 6; Cai 2020: 1407, figs 1–3; Annawaty et al. 2022: 346, fig. 5a–h.
Caridina nilotica var. bengalensis De Man, 1908a: 226. Type locality brackish ponds at Port Canning and at Dhappa, near Calcutta.
Caridina nilotica var. bengalensis — De Man 1908b: 265, pl. XX, fig. 6a, b; Kemp 1915: 307; Kemp 1918b: 275; Bouvier 1925: 246.
Caridina bengalensis — Mariappan and Richard 2006: 17, figs 8–11.
Caridina acuticaudata Dang, 1975: 70, fig. 4. Type locality “Hòa bình (Bắc Việt nam)” [= Hoa Binh, North Vietnam].

###### Material examined.

12 lots (see Suppl. material 1: Data S1).

###### Description.

**Figure 16.**
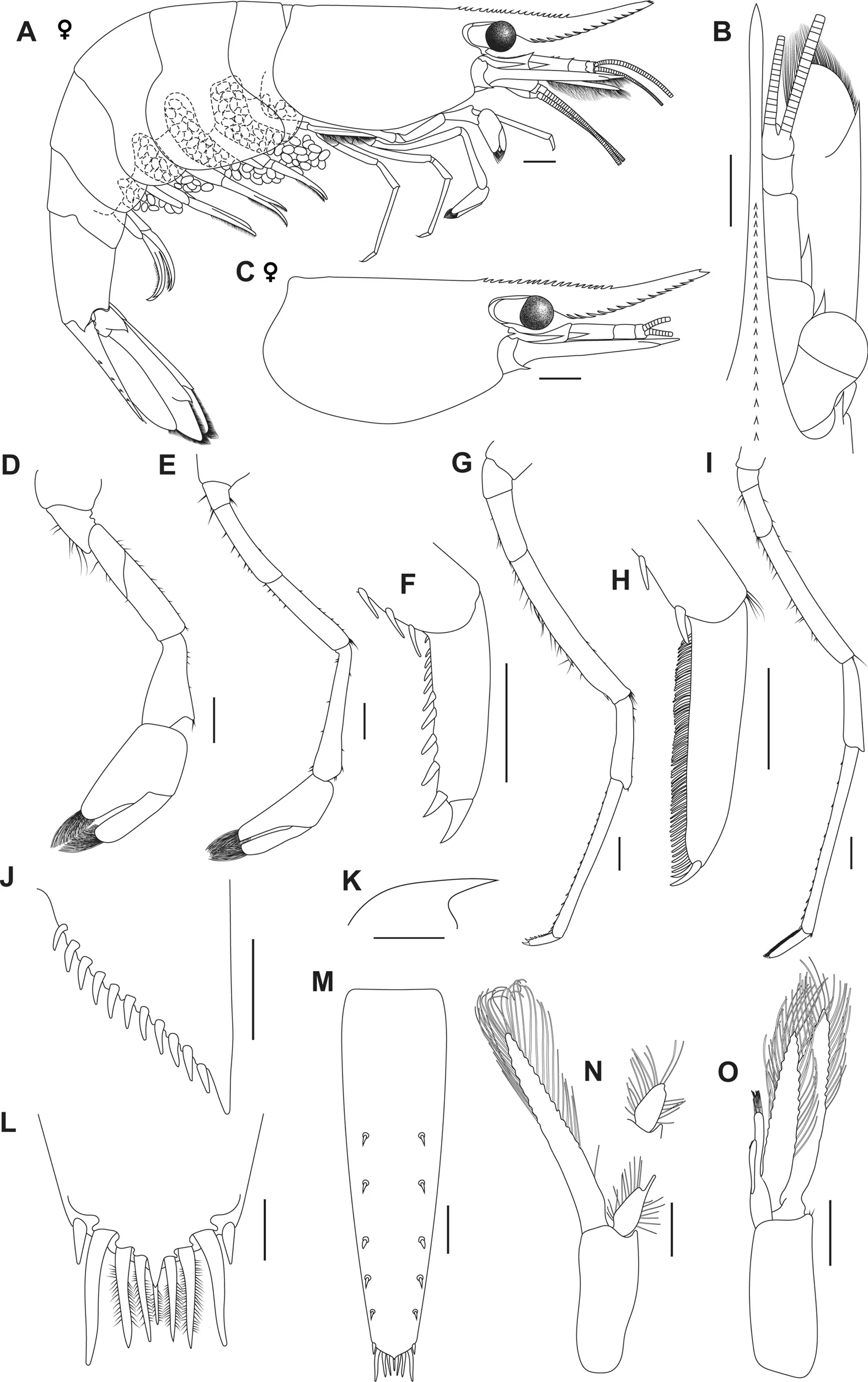
*Caridina gracilipes*. **A**. Ovigerous female; **B**. Top view of carapace; **C**. Cephalothorax and cephalic appendages of female; **D**. First pereopod; **E**. Second pereopod; **F**. Dactylus of third pereopod; **G**. Third pereopod; **H**. Dactylus of fifth pereopod; **I**. Fifth pereopod; **J**. Uropodal diaeresis; **K**. Preanal carina; **L**. Distal end of telson; **M**. Telson; **N**. First pleopod; **O**. Second pleopod. Drawings were made of MUMNH-CAR462-D1 (**A, B, D–M**); MUMNH-CAR218-D1 (**C**); MUMNH-CAR462-M1 (**N**; with appendix interna, **O**); MUMNH-CAR462-D2 (**N**; without appendix interna). Scale bars: 1 mm (**A–C**); 0.5 mm (**D, E, G, I, M–O**); 0.25 mm (**F, H, J–L**).

***Cephalothorax and cephalic appendage*** (*n* = 10). cl 4.12–5.39 (median 4.77; Fig. 16A, C) mm, width 3.06–4.50 (median 4.14) mm. Rostrum (Fig. 16A, C) straight or slightly curved downward in its proximal 1/2, then upcurved anteriorly, ending in bifid, reaching beyond the end of third segment of antennular peduncle (Fig. 16B), not longer than 2× of cl, 1.05–1.38 (median 1.16) × as long as cl. Rostral formula: (1–2) 14–21 + 0–1/ 13–17, dorsal teeth placed from the base to the middle of rostrum, subapical teeth sometimes present. Antennal spine placed below inferior orbital angle. Pterygostomian margin round. Eye well developed, anterior end reaching to 0.60–0.82 (median 0.72) of first segment of antennular peduncle. Antennular peduncle 0.72–0.97 (median 0.82; Fig. 16A–C) as long as cl, first segment 1.43–2.52 (median 1.74) × as long as second segment, second segment 1.42–2.39 (median 1.69) × as long as third segment. Tooth on distolateral margin of first segment of antennular peduncle acute and well developed. Stylocerite reaching to 0.68–0.89 (median 0.77) of first segment of antennular peduncle. Scaphocerite 3.38–4.09 (median 3.59) × as long as wide, distal margin with short plumose setae.

***Branchial and mouthparts*** (*n* = 3). Podobranch on second maxilliped well developed. Third maxilliped possessed one small and one large arthrobranch. Pleurobranchs present on all pereopods. Third maxilliped reaching to the end of second segment of antennular peduncle; ultimate segment of endopod ending with one large claw, eight to eleven spiniform setae on distal 1/3 of posterior margin, 0.76–0.96 (median 0.89, *n* = 10) as long as penultimate segment.

***Pereopods*** (*n* = 10). Epipod well developed on first four pereopods. Chelae of P1 and P2 well developed.

P1 short (Fig. 16D); chela 1.67–2.31 (median 2.19) × as long as wide, 1.23–1.87 (median 1.47) × as long as carpus, tips of fingers rounded with tuft of setae near tip; dactylus 0.84–1.47 (median 1.22) × as long as palm; carpus stout, excavated distally, 1.98–3.10 (median 2.31) × as long as wide, 0.70–1.09 (median 0.90) × as long as merus; merus 2.70–4.11 (median 3.16) × as long as wide, 1.15–1.61 (median 1.35) × as long as ischium.

P2 slenderer (Fig. 16E), longer than P1; chela 1.95–2.82 (median 2.56) × as long as wide, 0.70–0.88 (median 0.79) as long as carpus, tips of fingers rounded, with tuft of setae near tip; dactylus 1.02–1.76 (median 1.25) × as long as palm; carpus slender, 5.24–6.66 (median 6.05) × as long as wide, 1.03–1.52 (median 1.13) × as long as merus; merus 5.03–6.67 (median 6.20) × as long as wide, 0.92–1.50 (median 1.15) × as long as ischium.

P3 not sexually dimorphic (Fig. 16G); dactylus with 6–10 (mode 7) spiniform setae on flexor margin (Fig. 16F), 3.82–5.30 (median 4.35) × as long as wide (including terminal claw), terminating with one large claw; propodus with numerous setae on lateral and posterior margin, 14.16–16.95 (median 15.17) × as long as wide, 4.33–5.74 (median 5.22) × as long as dactylus; carpus with 3–5 (mode 4) setae on posterior margin of outer surface, the distal spine largest, other setae minute, 5.28–7.45 (median 6.90) × as long as wide, 0.51–0.62 (median 0.58) as long as propodus; merus possessed 2–5 (mode 4) large setae on posterior margin of outer surface, 7.50–10.13 (median 9.67) × as long as wide, 1.80–2.53 (median 1.97) × as long as carpus; ischium with one spiniform seta.

P5 slender (Fig. 16I); dactylus with 47–64 spiniform setae on flexor margin (Fig. 16H), 4.33–6.41 (median 5.33) × as long as wide (including terminal claw), terminating with one large claw; propodus with numerous setae on posterior margin, 17.34–19.73 (median 18.04) × as long as wide, 3.92–4.89 (median 3.54) × as long as dactylus; carpus with 1–3 (mode 2, 3) setae on posterior margin of outer surface, the distal spine largest, other setae minute, 5.51–7.37 (median 5.17) × as long as wide, 0.42–0.56 (median 0.50) as long as propodus; merus possessed two large setae on posterior margin of outer surface, 8.23–9.89 (median 9.44) × as long as wide, 1.51–1.94 (median 1.63) × as long as carpus; ischium without spiniform seta.

***Male pleopods*** (*n* = 10). Pl1 endopod subtriangular (Fig. 16N), wider proximally, 2.22–2.97 (median 2.52) × as long as wide, 0.22–0.27 (median 0.23) of exopod length, with or without appendix interna. Pl2 appendix masculina rod-shaped (Fig. 16O), slender, with many setae, 0.43–0.63 (median 0.58) as long as endopod; appendix interna reaching 0.72–0.95 (median 0.77) of appendix masculina.

***Abdomen*** (*n* = 10). Sixth abdominal somite, 0.62–0.88 (median 0.71) of cl, 1.44–1.99 (median 1.79) × as long as fifth somite, 0.84–0.98 (median 0.90) as long as telson (Fig. 16A). Telson 3.20–4.40 (median 3.89; Fig. 16M) × as long as wide, with four or five pairs (mode 5) of dorsal spiniform setae and one pair of dorsolateral spiniform setae; distal margin subtriangular, with posteromedian projection, 7–9 moveable plumose setae, lateral setae longest, middle seta shortest (Fig. 16L). Preanal carina large, high, with a large spine (Fig. 16K). Uropodal diaeresis with 10–15 moveable spiniform setae (Fig. 16J).

***Embryos***. Ovigerous females with 524–1,127 embryos (mean 853, *n* = 10). Size of embryos with eyes 0.38–0.44 × 0.22–0.29 mm (*n* = 50).

###### Coloration.

The body and rostrum quite translucent. Cephalic region darker than the other parts. Embryos brown (Fig. 14D).

###### Distribution and habitat.

India, Thailand, China, Vietnam, Taiwan, the Philippines, Malaysia, Singapore, Indonesia (Sulawesi, Selayar, Lombok, and Australia (Kemp 1918b; Cai and Shokita 2006b; Richard and Clark 2014; Cai 2014; de Mazancourt et al. 2018a; this study). This species is rarely encountered in southern Thailand. Its geographical distribution includes the coastal areas along both the western and eastern sides of the peninsula (Fig. 5D). *Caridina gracilipes* is commonly found in brackish water, streams, and ponds that undergo tidal influence. Individuals are commonly found among aquatic vegetation or beneath leaf litter along the banks.

###### Phylogenetic placement.

The molecular analyses supported the monophyly of *C. gracilipes* (BS = 99%, BPP = 1.00; Fig. 3) and placed it in the *C. nilotica* species group as sister to *C. brachydactyla*, although with moderate support (BS = 74%, BPP = 0.77).

###### Remarks.

The morphology of *C. gracilipes* from southern of Thailand is largely consistent with previous studies. The comparison of some significant characters between our specimens and those from previous reports is as follows: the rostral teeth formula: (1–2) 14–21 + 0 –1/ 13–17 vs (1–3) 10–27 + 1 –3/ 7–18 (De Man 1908b; Kemp 1918b; Richard and Clark 2014; de Mazancourt et al. 2018a; Cai 2020); rostrum length (1.05–1.38× as long as cl vs 0.80–1.50; Richard and Clark 2014; de Mazancourt et al. 2018a), P1 chela (1.67–2.31× as long as wide vs 1.8–2.7; Kemp 1918b; Richard and Clark 2014; de Mazancourt et al. 2018a; Cai 2020); P1 carpus (1.98–3.10× as long as wide vs 1.8–2.6; De Man 1908b; Richard and Clark 2014; de Mazancourt et al. 2018a; Cai 2020), P2 chela (1.95–2.82× as long as wide vs 2.3–3.2; Richard and Clark 2014; de Mazancourt et al. 2018a; Cai 2020), P2 carpus (5.24–6.66× as long as wide vs 4.4–6.3; Richard and Clark 2014; de Mazancourt et al. 2018a); P3 dactylus (3.82–5.30× as long as wide vs 2.5–5.1; De Man 1908b; Kemp 1918b; Richard and Clark 2014; de Mazancourt et al. 2018a), spiniform setae on P3 dactylus (6–9 vs 7–11; De Man 1908b; Kemp 1918b; Richard and Clark 2014; de Mazancourt et al. 2018a; Cai 2020); P5 dactylus (4.33–6.41× as long as wide vs 4.4–6.6; de Mazancourt et al. 2018a), spiniform setae on P5 dactylus (47–64 vs 34–70; De Man 1908b; Kemp 1918b; Richard and Clark 2014; de Mazancourt et al. 2018a; Cai 2020), moveable setae on uropodal diaeresis (10–15 vs 8–13; Richard and Clark 2014; de Mazancourt et al. 2018a), and embryo size (0.38–0.44 × 0.22–0.29 mm vs 0.32–0.46 × 0.18–0.27 mm; Richard and Clark 2014; de Mazancourt et al. 2018a).

Nevertheless, our specimens bear a relatively longer P3 propodus (4.33–5.74× as long as dactylus vs 2.9–4.5; Kemp 1918b; de Mazancourt et al. 2018a; Cai 2020); longer P5 propodus (3.92–4.89× as long as dactylus vs 2.7–3.8; Kemp 1918b; Richard and Clark 2014; Cai 2020), and slightly stouter Pl1 endopod (2.22–2.97× as long as wide vs 3.0–3.5; Richard and Clark 2014).

The presence of an appendix interna on the Pl1 endopod has been used as a diagnostic character to separate *C. gracilipes* from *C. bengalensis* De Man, 1908. The specimens possessing an appendix interna have been identified as *C. gracilipes*, whereas those lacking it have been assigned to *C. bengalensis* (Cai and Shokita 2006b; Mariappan and Richard 2006). However, Richard and Clark (2014) reexamined the type series of *C. gracilipes* deposited in Naturalis Biodiversity Center, Leiden. Interestingly, males of both morphotypes (with and without an appendix interna on Pl1) were found together in the type lot. This implied that the presence or absence of an appendix interna on the Pl1 endopod cannot be used as a reliable diagnostic character to separate *C. gracilipes* and *C. bengalensis* from each other. In the absence of any stable diagnostic character, Richard and Clark (2014) thus considered *C. bengalensis* to be a junior synonym of *C. gracilipes*. Subsequent studies have likewise treated both character states—presence and absence of an appendix interna on the endopod of Pl1—as intraspecific variation observed within *C. gracilipes* (Cai et al. 2007; Cai 2014; Richard and Clark 2014; de Mazancourt et al. 2018a). Our observations are consistent with this interpretation, as both character states were found intermixed among the examined populations in this study.

Among the three species of the *C. nilotica* species group occurring in southern Thailand, *C. gracilipes* is most similar to *C. macrophora*. Based on our specimens, *C. gracilipes* differs from *C. macrophora* by having a larger body size (cl of ovigerous female 4.12–5.39 mm vs 2.87–3.61 mm), much smaller embryos (0.38–0.44 × 0.22–0.29 mm vs 0.79–0.98 × 0.49–0.60 mm), higher preanal carina and with a conspicuous spine (vs low preanal carina and without a spine), and telson with posteromedian projection at the distal margin (vs rounded distal margin and without a posteromedian projection).

*Caridina gracilipes* can also be distinguished from *C. peninsularis*, another similar species encountered in southern Thailand, by the absence of dorsal teeth beyond the proximal 1/2 of the rostrum (vs dorsal teeth present throughout the dorsal margin or at least beyond the proximal 1/2 of the rostrum), having a spine on preanal carina (vs spine absence), and higher number of spiniform setae on P5 dactylus (47–64 vs 30–42).

##### Caridina macrophora

Taxon classificationAnimaliaDecapodaAtyidae

Kemp, 1918

0E20F72C-4469-5F11-A0C4-2FC64E9CFC18

Figs 5D, 14E, 17

Caridina nilotica subsp. macrophora Kemp, 1918b: 277, fig. 9a–f. Type locality “Tale sap in Peninsular Siam, only in the inner part of the lake in water that in all probability is permanently fresh. At the northern end of the Tale Sap in and near the mouth of the Patalung River” [= at the mouth of Lampam Stream, Phatthalung Province, Thailand (approximately 7.6232°N, 100.1508°E)].
Caridina nilotica macrophora — Liang and Yan 1983: 211; Li and Liang 2002: 708.
Caridina macrophora — Naiyanetr 2007: 34; Cai 2014: 209, figs 4, 5; Cai 2020: 1411, fig. 4; Cai and Naiyanetr 2024: 779.
Caridina subnilotica Dang, 1975: 69, fig. 3. Type locality “Hà nội (Bắc Việt nam)” [= Hanoi, North Vietnam].

###### Material examined.

37 lots (see Suppl. material 1: Data S1).

###### Description.

**Figure 17.**
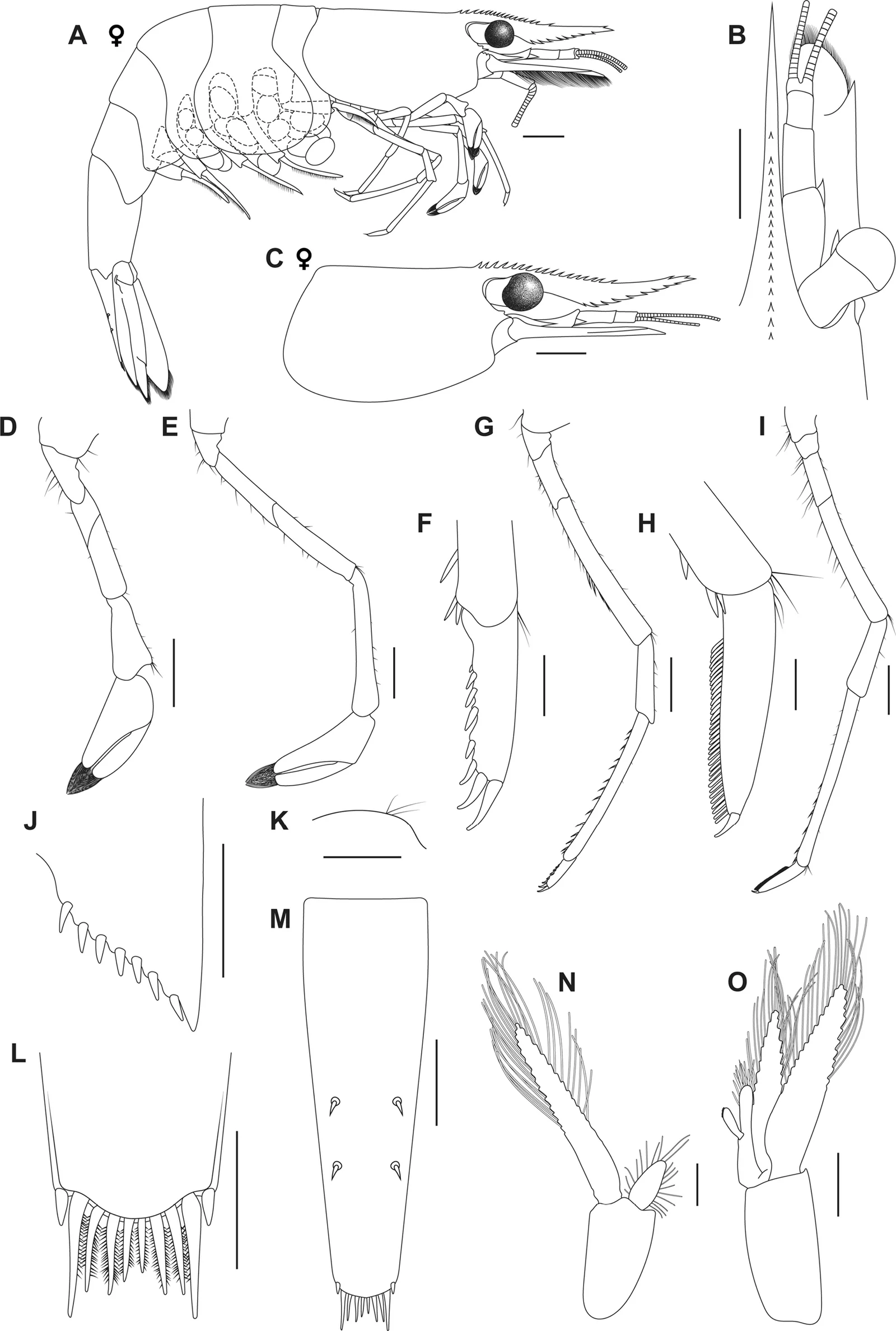
*Caridina macrophora*. **A**. Ovigerous female; **B**. Top view of carapace; **C**. Cephalothorax and cephalic appendages of female; **D**. First pereopod; **E**. Second pereopod; **F**. Dactylus of third pereopod; **G**. Third pereopod; **H**. Dactylus of fifth pereopod; **I**. Fifth pereopod; **J**. Uropodal diaeresis; **K**. Preanal carina; **L**. Distal end of telson; **M**. Telson; **N**. First pleopod; **O**. Second pleopod. Drawings were made of MUMNH-CAR458-D1 (**A, B, D–M**); MUMNH-CAR498-D1 (**C**); MUMNH-CAR498-M1 (**N, O**). Scale bars: 1 mm (**A–C**); 0.5 mm (**D, E, G, I, M**); 0.25 mm (**J–L, N, O**); 0.1 mm (**F, H**).

***Cephalothorax and cephalic appendage*** (*n* = 10). cl 2.87–3.61 (median 3.29; Fig. 17A, C) mm, width 2.43–2.72 (median 2.56) mm. Rostrum slightly to moderately curved downward in its proximal 1/2 (Fig. 17A, C), then upcurved anteriorly, ending in bifid, reaching beyond the end of third segment of antennular peduncle (Fig. 17B), but not longer than 2× of cl, 1.02–1.43 (median 1.23) × as long as cl. Rostral formula: (1–3) 13–19 + 0–2/ 7–14, distal 1/3 end of dorsal margin without teeth or sometimes with 1–2 subapical teeth. Antennal spine placed below inferior orbital angle. Pterygostomian margin broadly round. Eye well developed, anterior end reaching to 0.41–0.72 (median 0.53) of first segment of antennular peduncle. Antennular peduncle 0.71–0.89 (median 0.79; Fig. 17A–C) × as long as cl, first segment 2.15–2.81 (median 2.37) × as long as second segment, second segment 1.37–2.13 (median 1.79) × as long as third segment. Tooth on distolateral margin of first segment of antennular peduncle acute and well developed. Stylocerite reaching to 0.64–0.79 (median 0.69) of first segment of antennular peduncle. Scaphocerite 3.15–4.36 (median 3.60) × as long as wide, distal margin with short plumose setae.

***Branchial and mouthparts*** (*n* = 3). Podobranch on second maxilliped well developed. Third maxilliped possessed one small and one large arthrobranch. Pleurobranchs present on all pereopods. Third maxilliped reaching to end of second segment of antennular peduncle; ultimate segment of endopod ending with one large claw and six to nine spiniform setae on distal 1/3 of posterior margin, 0.72–0.93 (median 0.81, *n* = 10) as long as penultimate segment.

***Pereopods*** (*n* = 10). Epipod well developed on first four pereopods. Chelae of P1 and P2 well developed.

P1 short (Fig. 17D); chela 1.70–2.17 (median 1.93) × as long as wide, 1.32–1.67 (median 1.48) × as long as carpus, tips of fingers rounded, with tuft of setae near tip; dactylus 1.02–1.63 (median 1.19) × as long as palm; carpus stout, excavated distally, 1.86–2.72 (median 2.19) × as long as wide, 0.81–1.24 (median 1.11) × as long as merus; merus 2.70–4.62 (median 3.81) × as long as wide, 0.96–2.04 (median 1.55) × as long as ischium.

P2 slenderer (Fig. 17E), longer than P1; chela 2.31–3.43 (median 2.77) × as long as wide, 0.73–0.98 (median 0.81) as long as carpus, tips of fingers rounded, with tuft of setae near tip; dactylus 1.02–2.03 (median 1.38) × as long as palm; carpus slender, slightly excavated distally, 4.67–6.33 (median 5.80) × as long as wide, 1.20–1.43 (median 1.31) × as long as merus; merus 4.87–6.20 (median 5.76) × as long as wide, 1.00–1.37 (median 1.10) × as long as ischium.

P3 not sexually dimorphic (Fig. 17G); dactylus with 6–10 (mode 8) spiniform setae on flexor margin (Fig. 17F), 4.13–5.43 (median 4.81) × as long as wide (including terminal claw), terminating with one large claw; propodus bearing numerous spiniform setae on posterior margin, 10.15–12.21 (median 11.32) × as long as wide, 3.26–5.00 (median 4.12) × as long as dactylus; carpus possessed 1–4 (mode 3) small setae on posterior margin of outer surface, the distal seta largest, the other setae minute, 5.27–6.67 (median 6.14) × as long as wide, 0.46–0.61 (median 0.55) as long as propodus; merus possessed three or four (mode 3) large setae on posterior margin of outer surface, 8.38–11.63 (median 10.02) × as long as wide, 1.77–2.33 (median 2.07) × as long as carpus; ischium without spiniform seta.

P5 slender (Fig. 17I); dactylus with 31–40 spiniform setae on flexor margin (Fig. 17H), 3.36–5.00 (median 4.47) × as long as wide (including terminal claw), terminating with one large claw; propodus bearing numerous spiniform setae on posterior margin, 13.00–14.50 (median 13.33) × as long as wide, 2.97–4.58 (median 3.73) × as long as dactylus; carpus possessed one or two (mode 1) small setae on posterior margin of outer surface, the distal seta largest, the other setae minute, 4.13–6.19 (median 5.58) × as long as wide, 0.34–0.61 (median 0.44) as long as propodus; merus possessed 0–2 (mode 1) large setae on posterior margin of outer surface, 6.19–9.07 (median 8.17) × as long as wide, 1.24–2.31 (median 1.69) × as long as carpus; ischium without spiniform seta.

***Male pleopods*** (*n* = 10). Pl1 endopod subtriangular (Fig. 17N), wider proximally, 2.09–3.00 (median 2.46) × as long as wide, 0.16–0.29 (median 0.23) of exopod length, without appendix interna. Pl2 appendix masculina rod-shaped (Fig. 17O), with many setae, 0.39–0.62 (median 0.50) as long as endopod; appendix interna reaching 0.62–0.81 (median 0.71) of appendix masculina length.

***Abdomen*** (*n* = 10). Sixth abdominal somite ~ 0.66–0.96 (median 0.81) of cl, 1.75–2.52 (median 2.14) × as long as fifth somite, 0.88–1.24 (median 1.08) × as long as telson (Fig. 17A). Telson 3.30–4.64 (median 3.87; Fig. 17M) × as long as wide, with two or three pairs (mode 2) of dorsal spiniform setae and one pair of dorsolateral spiniform setae; distal margin round without posteromedian projection and 6–8 moveable plumose setae, lateral setae longest, intermediate pair shortest (Fig. 17L). Preanal carina rounded, blunt, without a spine (Fig. 17K). Uropodal diaeresis with 6–10 moveable spiniform setae (mode 7; Fig. 17J).

***Embryos***. Ovigerous females with 30–70 embryos (mean 53, *n* = 10). Size of eye-developed embryos 0.79–0.98 × 0.49–0.60 mm (*n* = 50).

###### Coloration.

Body rather translucent, pale brown, decorated with scattered dark brown spots. Rostrum brownish gold. Cephalic region, first to second dorsal somites, and telson are darker than other parts. Each abdominal somite decorated dorsally with transverse dark brown bands. Embryos brown (Fig. 14E).

###### Distribution and habitat.

Thailand, Cambodia, Vietnam, and South China (Guangxi and Hainan) (Kemp 1918b; Cai 2014, 2020; this study). In southern Thailand, *C. macrophora* occurs throughout the peninsula (Fig. 5D). This species is abundant in lentic freshwater habitats such as lakes, ponds, and reservoirs. It is sometimes found in lotic habitats such as small streams and lowland rivers. They are found living on floating plants or aquatic vegetation along the banks.

###### Phylogenetic placement.

The molecular analyses showed *C. macrophora* to be monophyletic (BS = 100%, BPP = 1.00; Fig. 3) and placed it as the basal clade within the *C. nilotica* species group.

###### Remarks.

*Caridina macrophora* was described as ‘*Caridina nilotica* subsp. *macrophora’* by Kemp (1918b), based on specimens collected by Dr. Annandale. The type locality was cited as “the northern end of the Tale Sap in and near the mouth of the Patalung River” where the shrimp was specifically noted to occur only in the inner portion of the lake, where water is likely permanently fresh. However, the name “Patalung River”, as it appeared on a map illustrated by Annandale (1916), is not associated with any major waterway today. Based on his mapping, the water body designated as the “Patalung River” in Annandale’s context is now probably known as the Lampam Stream, the major waterway that flows into the northern region of the Songkhla Lake (approximately 7.6232°N, 100.1508°E). This historical correlation provides a precise geographical correction for the detailed type locality. Later, it was considered as a full species by subsequent authors (Naiyanetr 2007; Cai 2014, 2020; Cai and Naiyanetr 2024).

In this study, we failed to obtain *C. macrophora* from its type locality. However, the morphology of *C. macrophora* in our collection is well compatible with the original description by Kemp (1918b), including the rostral formula: (1–3) 13–19/ 7–14 vs (1–3) 13–20/ 6–12, spiniform setae on P3 dactylus (6–14 vs 6–10), spiniform setae on P5 dactylus (31–40 vs 35–45), P1 dactylus (1.02–1.63× as long as palm vs 1.5), P1 carpus (1.86–2.72× as long as wide vs 2.5), P2 dactylus (1.02–2.03× as long as palm vs 1.5), P2 carpus (4.67–6.33× as long as wide vs 5.5–7), P3 dactylus (4.13–5.43× as long as wide vs 4.3–5) P3 propodus (3.26–5.00× as long as dactylus vs 3.25–3.3), P5 dactylus (3.36–5.50× as long as wide vs 4.25–4.75), P5 propodus (2.97–4.58× as long as dactylus vs 3.25–3.3), spiniform setae on uropodal diaeresis (6–10 vs 8–9), and embryo size (0.79–0.98 × 0.49–0.60 mm vs 0.90–0.96 × 0.52–0.58 mm). However, P5 ischium of our specimens lacks a large spine, while Kemp (1918b) clearly reported its presence. This discrepancy may indicate intraspecific variation or suggest that this character is less stable than previously assumed.

Cai (2014, 2020) reexamined syntypes deposited in Muséum national d’Histoire naturelle, Paris. The morphology of our specimens is largely consistent with his reports, except for a longer P2 carpus (4.67–6.33× as long as wide vs 4.3) and lower number of dorsal spiniform setae on telson (2–3 pairs vs 3–5 pairs).

*Caridina macrophora* can be distinguished from the other species in the *C. nilotica* species group by its small and unarmed preanal carina, rounded distal margin of telson without a posteromedian projection, fewer moveable spiniform setae on uropodal diaeresis, an absence of appendix interna of endopod of male first pleopod, and its much larger embryos (Kemp 1918b; Richard and Clark 2010, 2014; Cai 2014, 2020; de Mazancourt et al. 2018a).

##### Caridina peninsularis

Taxon classificationAnimaliaDecapodaAtyidae

Kemp, 1918

F861CF18-0452-506E-A823-BFFF3F5D9299

Figs 5D, 14F, 18

Caridina brachydactyla subsp. peninsularis Kemp, 1918b: 279, fig. 10a–g. Type locality “Patani, in the Siamese Malay States and on Penang” [= Pattani Province, Thailand and Penang, Malaysia].
Caridina brachydactyla var. peninsularis — Bouvier 1925: 155, figs 321, 322.
Caridina brachydactyla peninsularis — Johnson 1961b: 45; Dutt and Ravindranath 1975: 269–270.
Caridina simoni peninsularis — Johnson 1966: 422; Johnson 1969: 110; Ng and Choy 1990: 16; de Silva 1983: 209.
Caridina peninsularis — Wowor et al. 2004: 343, fig. 6j; Cai and Shokita 2006b: 248; Cai et al. 2007: 280, figs 2, 3; Richard and Clark 2014: 315, figs 6–8; Cai and Naiyanetr 2024: 778.
Caridina mccullochi Roux, 1926b: 249. Type locality New South Wales.
Caridina mccullochi — Riek 1953: 118.
Caridina nilotica var. brachydactyla [non De Man, 1908b] — Johnson 1961a: 123.
Caridina meridionalis [non Roux, 1926a] — Roux 1926a: 207 (in part).
Caridina *simony* [sic; non Bouvier, 1904] — Johnson 1963: 21 (in part).

###### Material examined.

31 lots (see Suppl. material 1: Data S1).

###### Description.

**Figure 18.**
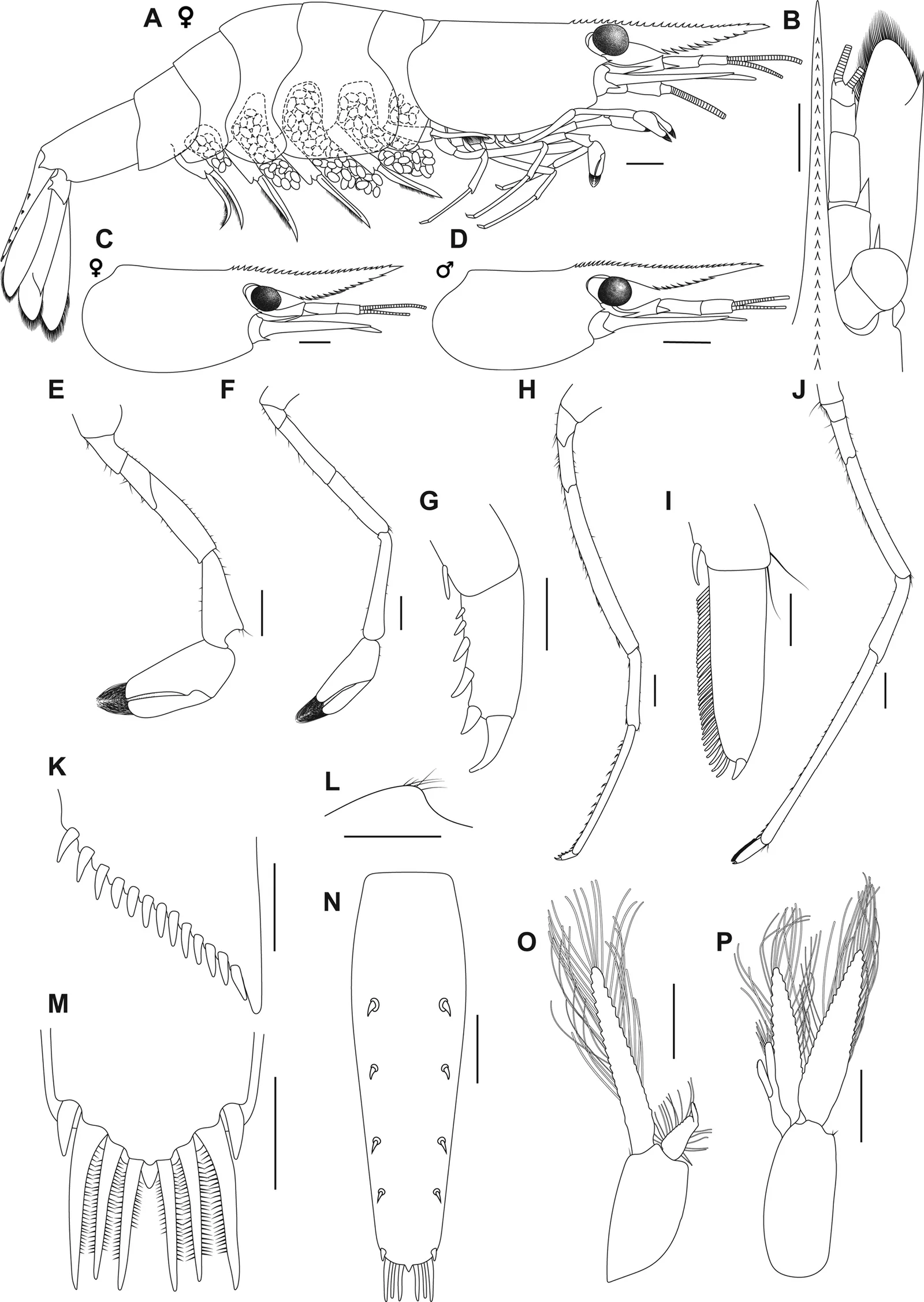
*Caridina peninsularis*. **A**. Ovigerous female; **B**. Top view of carapace; **C**. Cephalothorax and cephalic appendages of female; **D**. Cephalothorax and cephalic appendages of male; **E**. First pereopod; **F**. Second pereopod; **G**. Dactylus of third pereopod; **H**. Third pereopod; **I**. Dactylus of fifth pereopod; **J**. Fifth pereopod; **K**. Uropodal diaeresis; **L**. Preanal carina; **M**. Distal end of telson; **N**. Telson; **O**. First pleopod; **P**. Second pleopod. Drawings were made of MUMNH-CAR451-D1 (**A, B, E–N**); MUMNH-CAR134-D1 (**C**); MUMNH-CAR451-M1 (**D, O, P**). Scale bars: 1 mm (**A–D**); 0.5 mm (**E, F, H, J, N–P**); 0.25 mm (**K–M**); 0.1 mm (**G, I**).

***Cephalothorax and cephalic appendage*** (*n* = 10). cl 4.02–5.63 (median 4.73; Fig. 18A, C) mm, width 2.85–3.84 (median 3.43) mm. Rostrum straight or slightly upcurved anteriorly (Fig. 18A, C), frequently ending in bifid, reaching beyond the end of third segment of antennular peduncle (Fig. 18B), but not longer than 2× of cl, 0.96–1.15 (median 1.05) × as long as cl. Rostral formula: (2–5) 21–32/ 7–14, dorsal teeth usually placed throughout dorsal margin. Antennal spine placed slightly below inferior orbital angle. Pterygostomian margin round. Eye well developed, anterior end reaching to 0.45–0.75 (median 0.61) of first segment of antennular peduncle. Antennular peduncle 0.75–0.88 (median 0.82; Fig. 18A–C) as long as cl, first segment 1.55–1.94 (median 1.80) × as long as second segment, second segment 1.58–2.03 (median 1.77) × as long as third segment. Tooth on distolateral margin of first segment of antennular peduncle acute and well developed. Stylocerite reaching to 0.61–0.85 (median 0.74) of first segment of antennular peduncle. Scaphocerite 3.26–4.00 (median 3.48) × as long as wide, distal margin with short plumose setae.

***Branchial and mouthparts*** (*n* = 3). Podobranch on second maxilliped well developed. Third maxilliped possessed one small and one large arthrobranch. Pleurobranchs present on all pereopods. Third maxilliped reaching to end of second segment of antennular peduncle; ultimate segment of endopod ending with one large claw and six to eleven spiniform setae on distal 1/3 of posterior margin, 0.66–0.87 (median 0.78, *n* = 10) as long as penultimate segment.

***Pereopods*** (*n* = 10). Epipod well developed on first four pereopods. Chelae of P1 and P2 well developed.

P1 short (Fig. 18E); chela 1.78–2.28 (median 2.13) × as long as wide, 1.00–1.30 (median 1.21) × as long as carpus, tips of fingers rounded, with tuft of setae near tip; dactylus 1.13–2.03 (median 1.50) × as long as palm; carpus stout, excavated distally, 2.44–3.73 (median 3.31) × as long as wide, 0.87–1.38 (median 1.09) × as long as merus; merus 3.08–3.86 (median 3.48) × as long as wide, 1.32–2.06 (median 1.64) × as long as ischium.

P2 slenderer and longer than P1 (Fig. 18F); chela 2.38–2.91 (median 2.63) × as long as wide, 0.64–0.86 (median 0.78) as long as carpus, tips of fingers rounded, with tuft of setae near tip; dactylus 1.12–2.10 (median 1.53) × as long as palm; carpus slender, 4.98–6.70 (median 5.60) × as long as wide, 1.06–1.45 (median 1.30) × as long as merus; merus 4.78–6.10 (median 5.59) × as long as wide, 0.93–1.56 (median 1.24) × as long as ischium.

P3 not sexually dimorphic (Fig. 18H); dactylus with four or five (mode 5) spiniform setae on flexor margin (Fig. 18G), 2.54–3.33 (median 2.68) × as long as wide (including terminal claw), terminating with one large claw; propodus bearing numerous spiniform setae on posterior margin, 14.14–18.55 (median 15.45) × as long as wide, 6.21–8.93 (median 7.74) × as long as dactylus; carpus possessed 2–4 (mode 3) setae on posterior margin of outer surface, the distal seta largest, the other setae minute, 5.93–7.49 (median 6.44) × as long as wide, 0.51–0.61 (median 0.56) as long as propodus; merus possessed 3–5 (mode 4) large setae on posterior margin of outer surface, 8.29–11.00 (median 9.09) × as long as wide, 1.66–2.13 (median 2.06) × as long as carpus; ischium without spiniform seta.

P5 slender (Fig. 18J); dactylus with 30–42 spiniform setae on flexor margin (Fig. 18I), 2.87–4.53 (median 3.41) × as long as wide (including terminal claw), terminating with one large claw; propodus bearing numerous spiniform setae on posterior margin, 16.13–19.53 (median 17.57) × as long as wide, 4.72–7.47 (median 5.51) × as long as dactylus; carpus possessed 1–4 (mode 3) setae on posterior margin of outer surface, the distal seta largest, the other setae minute, 4.47–7.06 (median 6.04) × as long as wide, 0.40–0.52 (median 0.45) as long as propodus; merus possessed one or two (mode 2) large setae on posterior margin of outer surface, 7.47–9.04 (median 8.29) × as long as wide, 1.40–1.87 (median 1.68) × as long as carpus; ischium without spiniform seta.

***Male pleopods*** (*n* = 10). Pl1 endopod subtriangular (Fig. 18O), wider proximally, 1.62–2.73 (median 1.96) × as long as wide, 0.20–0.27 (median 0.24) of exopod length, with elongated appendix interna emerged near the tip. Pl2 appendix masculina rod-shaped (Fig. 18P), slender with many setae, 0.56–0.79 (median 0.68) as long as endopod; appendix interna reaching 0.55–0.70 (median 0.63) of appendix masculina length.

***Abdomen*** (*n* = 10). Sixth abdominal somite ~ 0.59–0.70 (median 0.66) of cl, 1.75–2.13 (median 1.96) × as long as fifth somite, 0.85–1.03 (median 0.95) × as long as telson (Fig. 18A). Telson 3.24–4.16 (median 3.48; Fig. 18N) × as long as wide, with three or four pairs (mode 4) of dorsal spiniform setae and one pair of dorsolateral spiniform setae; distal margin round with posteromedian projection and 6–8 moveable plumose setae, similar in length (Fig. 18M). Preanal carina subtriangular, with few setae, without a spine (Fig. 18L). Uropodal diaeresis with 11–15 moveable spiniform setae (Fig. 18K).

***Embryos***. Ovigerous females with 515–1,483 embryos (mean 847, *n* = 10). Size of eye-developed embryos 0.38–0.44 × 0.22–0.29 mm (*n* = 50).

###### Coloration.

Body either deep-sea blue or brownish grey and furnished with many sandy brown, graphite, and bistre patches, and many small dark spots. Rostrum translucent or either olive green or smokey grey. Ventral region of rostrum darker than the upper region. Cephalic region, the last two somites, and telson are darker than other parts. Embryos clay brown to greenish brown (Fig. 14F).

###### Distribution and habitat.

Thailand, Malaysia, Singapore, the Philippines, Indonesia (Borneo), India, Sri Lanka, and Australia (Cai et al. 2007; Richard and Clark 2014; this study). In southern Thailand, *C. peninsularis* is widely distributed throughout the southern part of the peninsula (Fig. 5D). This species is common to lowland rivers and streams. They are found living on aquatic vegetations, under rocks along the banks, where the bottom substrate can be gravel, sand, or mud.

###### Phylogenetic placement.

Our molecular analyses support the monophyly of *C. peninsularis* (BS = 85%, BPP = 0.99) and place it in the *C. nilotica* group (Fig. 3). It is sister to *C. simoni* with high nodal support (BS = 99%, BPP = 1.00).

###### Remarks.

The morphology of *C. peninsularis* in our material agrees well with the original description by Kemp (1918b), particularly with respect to the following characters: shape of rostrum, the rostral formula: (2–5) 21–32/ 7–14 vs (3–4) 21–37/ 6–17, P1 carpus (2.44–3.73× as long as wide vs 2.2–2.6), P3 dactylus (2.54–3.33× as long as wide vs 2.0–2.6), setae on posterior margin of P3 merus (3–5 vs 2–4), spiniform setae on P3 dactylus (4–6 vs 5–7), spiniform setae on P5 dactylus (30–42 vs 29–43), dorsal spiniform setae on telson (3–4 pairs vs 3–5 pairs), spiniform setae on uropodal diaeresis (11–14 vs 12–14), and embryo size (0.37–0.42 × 0.20–0.27 mm vs 0.35–0.42 × 0.25–0.30 mm).

Additionally, the morphological of our specimens also match with that reported in subsequent studies: rostral formula: (2–5) 21–32/ 7–14 vs (2–3) 15–37/ 5–12 (Cai et al. 2007) rostral length 0.86–1.04× as long as cl (vs 0.85–1.0; Richard and Clark 2014), P1 dactylus (1.13–2.03× as long as palm vs 1.2–1.6; Cai et al. 2007; Richard and Clark 2014), P1 carpus (2.44–3.73× as long as wide vs 2.5–3.1; Cai et al. 2007; Richard and Clark 2014), P2 chela (2.38–2.91× as long as wide vs 2.7–3.2; Richard and Clark 2014), P2 dactylus (1.12–2.10× as long as palm vs 1.2–1.7; Cai et al. 2007; Richard and Clark 2014), P3 dactylus (2.54–3.33× as long as wide vs 2.5–4.0; Cai et al. 2007; Richard and Clark 2014), spiniform setae on P3 dactylus (4–6 vs 4–8; Cai et al. 2007; Richard and Clark 2014), spiniform setae on P5 dactylus (30–42 vs 38–50; Cai et al. 2007; Richard and Clark 2014), sixth abdominal somite (0.59–0.70× as long as cl vs 0.60–0.75; Richard and Clark 2014), and embryo size (0.37–0.42 × 0.20–0.27 mm vs 0.40–0.42 × 0.25–0.30 mm; Cai et al. 2007).

However, our specimens tend to have a stouter P1 chela (1.78–2.28× as long as wide vs 2.1–2.5; Cai et al. 2007; Richard and Clark 2014), slightly slenderer P2 carpus (4.98–6.70× as long as wide vs 4.9–5.8; Kemp 1918b; Cai et al. 2007), slenderer P3 propodus (6.21–8.93× as long as dactylus vs 4.5–6.6; Kemp 1918b; Cai et al. 2007; Richard and Clark 2014). Moreover, our ovigerous specimens carry smaller embryos (0.37–0.42 × 0.20–0.27 mm vs 0.49–0.58 × 0.23–0.35 mm; Richard and Clark 2014).

*Caridina peninsularis* can be distinguished from *C. brachydactyla* and *C. simoni* by its rostral features. The dorsal teeth of *C. peninsularis* appear throughout the dorsal margin (vs appear only in the proximally 1/2 in the latter two species), and subapical teeth on the dorsal margin are absent (vs present in the latter two species) (Cai et al. 2007; Richard and Clark 2010, 2014).

*Caridina peninsularis* can also be separated from other species in the *C. nilotica* group occurring in southern Thailand by the following characters. Its dorsal teeth on the rostrum appear on the entire dorsal margin (vs not present beyond the proximal 2/3 in *C. gracilipes* and *C. macrophora*). The preanal carina of *C. peninsularis* is subtriangular and lacks a spine (vs subtriangular with a spine in *C. gracilipes*, and vs rounded without a spine in *C. macrophora*). Furthermore, the embryo size of *C. peninsularis* is considerably smaller than that of *C. macrophora* (0.37–0.42 × 0.20–0.27 mm vs 0.79–0.98 × 0.49–0.60 mm), but comparable to that of *C. gracilipes* (0.38–0.44 × 0.22–0.29 mm).

#### *Caridina laevis* species group

**Diagnosis**. Rostrum varies in both shape and length, frequently sigmoidal, typically reaching near or slightly beyond the distal end of antennular peduncle. Antennular peduncle long, at least 0.6 of cl. The number of epipod variable: an epipod on P1 always present, epipods on P2 and P3 sometimes reduced or absent, epipods on the last two pereopods always absent. Lateral plumose setae on distal end of telson usually slightly longer than median plumose setae. Uropodal diaeresis usually with < 20 moveable spiniform setae. Endopod of male first pleopod with or without appendix interna.

**Remarks**. In the present study, ten southern Thai *Caridina* species are included in this species group: *C. excavatoides*, *C. johnsoni*, *C. kottelati*, *C. laevis*, *C. nangroma* sp. nov., *C. cf. rangoona*, *C. srivijaya* sp. nov., *C. takkola* sp. nov., *C. tambralinga* sp. nov., and *C. temasek*, as well as *C. panhai* Macharoenboon, Sutcharit, Siriwut & Jeratthitikul, 2023, an endemic species from Mekong Basin. All of these species were recovered as monophyletic with strong support (BS = 95–100%, BPP = 0.99–1.00; Fig. 4). Our phylogenetic tree showed that these species can be divided into two groups, consisting of (1) a group with low nodal support comprising *C. johnsoni*, *C. laevis*, *C. panhai*, and *C. takkola* sp. nov. (BS = 50%, BPP = 0.59; Fig. 4), and (2) a well-supported clade consisting of *C. excavatoides*, *C. nangroma* sp. nov., *C. cf. rangoona*, *C. srivijaya* sp. nov. *C. tambralinga* sp. nov., and *C. temasek*. (BS = 84%, BPP = 0.99; Fig. 4). These two clades are not supported by any clear diagnostic characters.

Based on the studies by Bouvier (1905, 1925), the diagnostic characters of the *C. laevis* species group include a straight or bent-downward rostrum, rostrum length not reaching beyond antennular peduncle, moderate antennular peduncle ~ 0.75 as long as cl, uropodal diaeresis with 9–12 moveable spiniform setae. Subsequently, this species group received little attention, and later authors merely expanded its diagnosis by adding additional characters and documenting their variation (e.g., Johnson 1961a; Lakshmi 1975; Jalihal et al. 1984). Our morphological analysis of eleven species within this group largely confirms the original diagnostic characters. However, we observed an exception regarding the number of spiniform setae on the uropodal diaeresis. The count in Thai species is variable but consistently ranges from 10 to 20. Furthermore, while the rostral shape in the *C. laevis* species group generally exhibits a slight downward slope, the specific shape and length are notably variable among species. For example, *C. panhai* is characterized by an elongated sigmoidal rostrum, while *C. takkola* sp. nov. exhibits a very short convex rostrum. The rostrum of *C. nangroma* sp. nov. is sloped downward, wider at the middle, and tapers toward the tip, contrasting with that of *C. johnsoni* which is relatively narrow and maintains subequal width throughout its length. Overall, these detailed comparisons reveal that the diagnostic characters of the *C. laevis* species group are not rigidly defined and exhibit significant morphological variation among species assigned to this group.

The current phylogenetic tree indicated that the *C. laevis* species group can be distinctly separated from other *Caridina* species groups with maximum nodal support (BS = 100%, BPP = 1.00). Morphologically, this species group possesses a unique character of epipods, which are always present on P1, reduced or absent on P2 and P3 and completely absent on the last two pereopods. Other species groups, such as *C. gracilirostris*, *C. lanceifrons*, *C. nilotica*, *C. sumatrensis*, and *C. weberi* species groups, exhibit well developed epipods on the first four pereopods (de Mazancourt et al. 2020; this study).

##### Caridina laevis

Taxon classificationAnimaliaDecapodaAtyidae

Heller, 1862

8888D759-1F70-5649-A327-AFDB058961DB

Figs 5E, 19

Caridina laevis Heller, 1862: 411. Type locality Java.
Caridina laevis — Blanco 1935: 34, pl. 3, figs 26–32; Ortmann 1894b: 402, 404; Bouvier 1904: 132; Bouvier 1905: 74; Kemp 1918b: 289; Bouvier 1925: 183; Pillai 1964: 42; Chace 1997: 10.
Caridina cf. laevis — Cai and Naiyanetr 2024: 771.

###### Material examined.

Two lots (see Suppl. material 1: Data S1).

###### Description.

**Figure 19.**
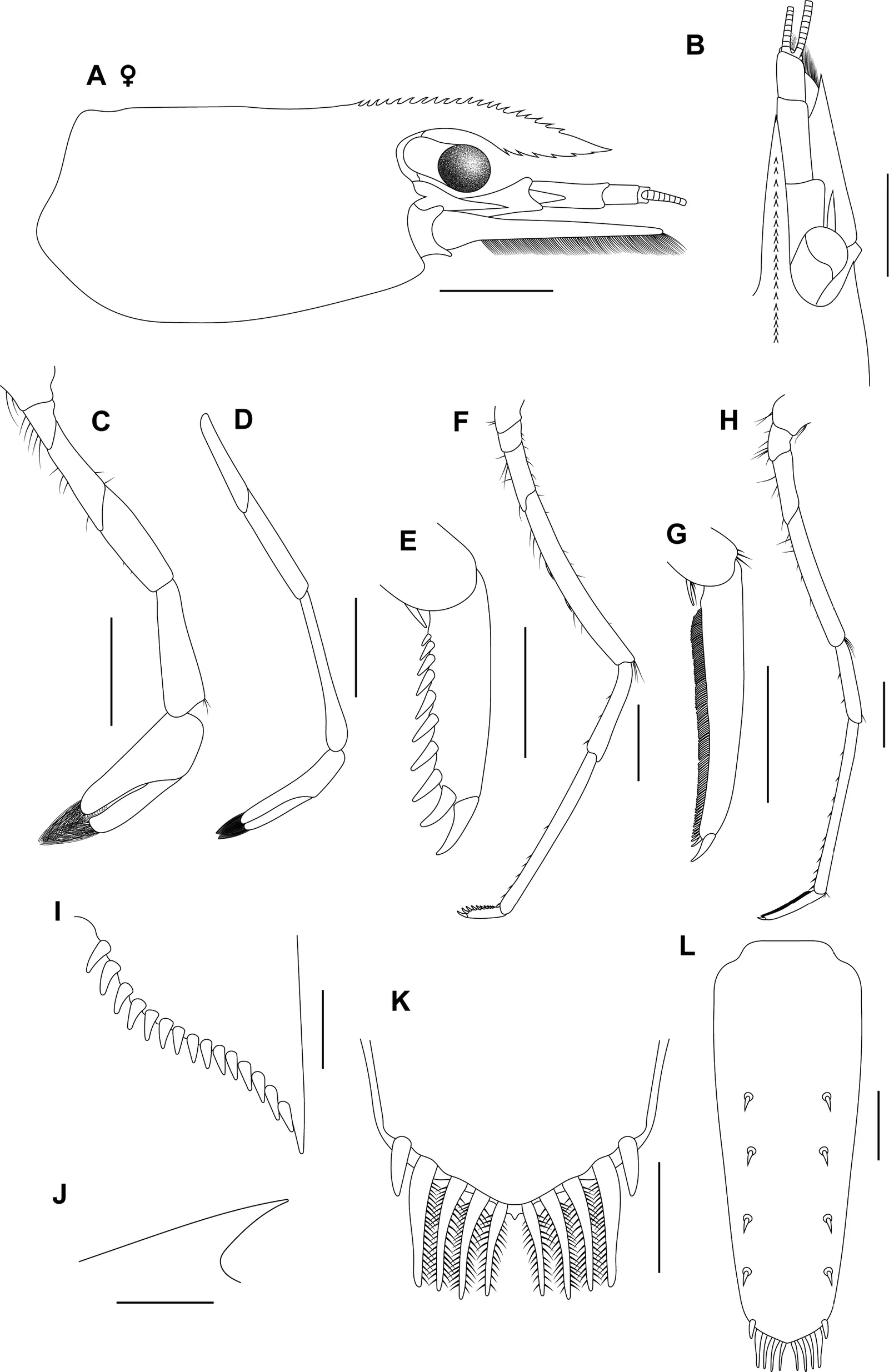
*Caridina laevis*. **A**. Cephalothorax and cephalic appendages of female; **B**. Top view of carapace; **C**. First pereopod; **D**. Second pereopod; **E**. Dactylus of third pereopod; **F**. Third pereopod; **G**. Dactylus of fifth pereopod; **H**. Fifth pereopod; **I**. Uropodal diaeresis; **J**. Preanal carina; **K**. Distal end of telson; **L**. Telson. Drawings were made of ZMB30683-1 (**A–F, I–L**); ZMB 30702-3 (**G, H**). Scale bar: 2 mm (**A, B**); 1 mm (**C, D, F–H**); 0.5 mm (**G, I, L**); 0.25 mm (**E, J, K**).

***Cephalothorax and cephalic appendage*** (*n* = 2). cl 5.02–5.54 mm, width 3.73–3.76 mm (Fig. 19A). Rostrum relatively large (Fig. 19A), slightly sigmoidal, strongly sloped downward, reaching near the end of second segment of antennular peduncle (Fig. 19B), 0.54–0.68 as long as cl. Rostral formula: (3–5) + 13–20/ 3–6. Antennal spine placed below inferior orbital angle margin. Pterygostomian margin subrectangular. Eye well developed, anterior end reaching to 0.60 of first segment of antennular peduncle. Antennular peduncle 0.74–0.76 as long as cl (Fig. 19A, B), first segment 1.56–2.02× as long as second segment, second segment 2.15–2.70× as long as third segment. Tooth on distolateral margin of first segment of antennular peduncle long, acute. Stylocerite reaching to 0.76–0.86 of first segment of antennular peduncle.

***Pereopods*** (*n* = 2). Epipod only present on first two pereopods, absent in the last three pereopod. Chelae of P1 and P2 well developed.

P1 short (Fig. 19C); chela 2.63–3.17× as long as wide, 1.10–1.27× as long as carpus, tips of fingers rounded, with tuft of setae near tip; dactylus 1.48–1.96× as long as palm; carpus slightly excavated distally, 2.81–3.40× as long as wide, 1.10–1.19× as long as merus; merus 3.12–4.32× as long as wide, 0.90–1.05× as long as ischium.

P2 slenderer (Fig. 19D), longer than P1; chela 4.31–4.87× as long as wide, 0.73–0.80 as long as carpus, tips of fingers rounded, with tuft of setae near tip; dactylus 1.55–1.62× as long as palm; carpus slender, 6.99–8.38× as long as wide, 1.42–1.45× as long as merus; merus 6.02–6.54× as long as wide, 0.94–1.10× as long as ischium.

P3 slender (Fig. 19F); dactylus with 6–9 spiniform setae on flexor margin (Fig. 19E), 4.00–4.26× as long as wide (including terminal claw), terminating with one large claw; propodus bearing numerous spiniform setae on posterior margin, 13.00–14.00× as long as wide, 3.54–3.92× as long as dactylus; carpus 5.56–6.59× as long as wide, 0.52–0.66 as long as propodus; merus possessed three large setae on posterior margin of outer surface, 9.18–9.60× as long as wide, 1.80–2.11× as long as carpus.

P5 slender (Fig. 19H); dactylus with 92–105 spiniform setae on flexor margin (Fig. 19G), 5.71–7.17× as long as wide (including terminal claw), terminating with one large claw; propodus bearing numerous spiniform setae on posterior margin, 13.38–16.67× as long as wide, 2.38–2.78× as long as dactylus; carpus possessed one seta on posterior margin of outer surface, 5.57–6.11× as long as wide, 0.46–0.51 as long as propodus; merus possessed three large setae on posterior margin of outer surface, 8.00–9.00× as long as wide, 1.48–1.70× as long as carpus.

***Abdomen*** (*n* = 2). Telson 2.70× as long as wide (Fig. 19L), with three or four pairs of dorsal spiniform setae and one pair of dorsolateral spiniform setae; distal margin broadly triangular to rounded with a small posteromedian projection and 6–8 moveable plumose setae, similar in length (Fig. 19K). Preanal carina high, with a prominent spine (Fig. 19J). Uropodal diaeresis with 15–18 moveable spiniform setae (Fig. 19I).

###### Coloration.

Transparent to brownish with cream transverse stripes on abdomen, some populations translucent bright orange.

###### Distribution and habitat.

Indonesia (Java), India (Travancore, corresponding to the southern part of modern India), and Thailand (Heller 1862; Pillai 1964; Kemp 1918b; Kamita 1966). *Caridina laevis* was reported from Thailand by Kamita (1966) without any locality details. Based on the current study, *C. laevis* is rarely found in southern Thailand. The species has been documented from only two localities (Fig. 5E). This species inhabits brackish water.

###### Phylogenetic placement.

Our molecular analyses support the monophyly of *C. laevis* (BS = 100%, BPP = 1.00; Fig. 4) and place it as a sister clade to *C. panhai* with high support (BS = 100%, BPP = 1.00).

###### Remarks.

Our specimens agreed well with previous observations, including slightly sigmoidal rostrum (Heller 1862; Bouvier 1925; Pillai 1964), slender P2 carpus (6.99–8.38× as long as wide vs 6.5–7.4; Pillai 1964), and numerous spiniform setae on P5 dactylus (92–105 vs 58–95; Bouvier 1925; Pillai 1964).

The current phylogenetic tree indicated a sister relationship between *C. laevis* and *C. panhai* with high nodal support (BS = 100%, BPP = 1.00; Fig. 4). Morphologically, these species share several characters such as a long sigmoidal rostrum, slender P2 chela, and slender P5 dactylus. However, *C. laevis* differs from *C. panhai* in having a larger number of dorsal teeth (13–20 vs 9–15), stouter P2 carpus (6.99–8.38× as long as wide vs 8.11–10.04), and a larger number of spiniform setae on P5 dactylus (92–105 vs 57–68; Macharoenboon et al. 2023). Notably, their geographic distributions are allopatric. The distribution of *C. panhai* is limited to the Middle Mekong Basin (Macharoenboon et al. 2023); while that of *C. laevis* is broader from Java, Southern India, to southern Thailand (Heller 1862; Pillai 1964; Kemp 1918b; Kamita 1966; this study).

##### Caridina excavatoides

Taxon classificationAnimaliaDecapodaAtyidae

Johnson, 1961

91470817-1801-5BCB-90F2-441F1075F6C9

Figs 5E, 14G, 20

Caridina excavatoides Johnson, 1961a: 127, figs 3–11. Type locality a small, slow, stream running between rubber plantations and rice-fields, ~ 9 miles from Alor Star, Kedah, on the Pokok Sena Road, Malaysia.
Caridina excavatoides — Wowor et al. 2004: 343, fig. 7t, u; Cai et al. 2007: 294, figs 11, 12.

###### Material examined.

Seven lots (see Suppl. material 1: Data S1).

###### Description.

**Figure 20.**
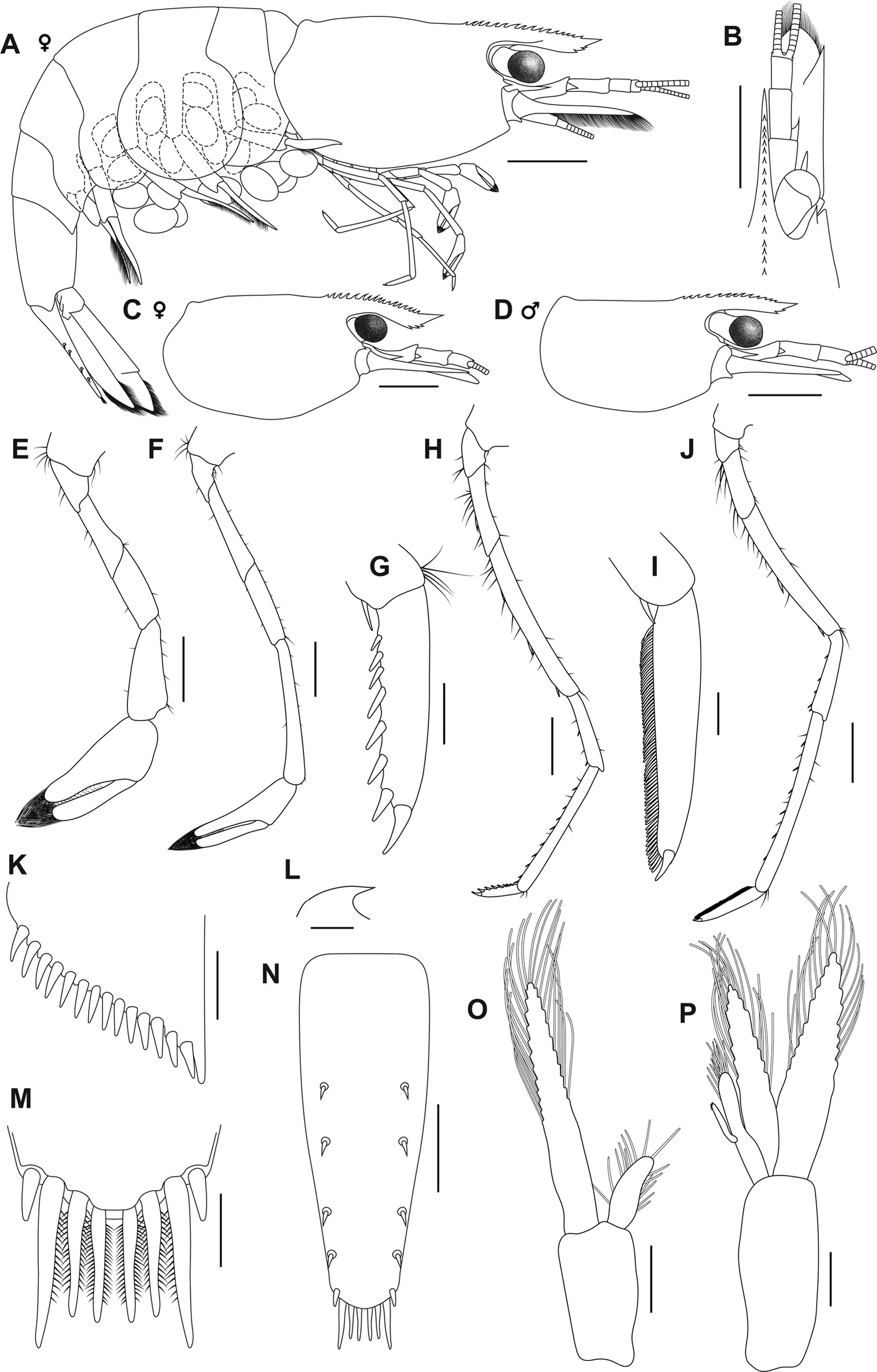
*Caridina excavatoides*. **A**. Ovigerous female; **B**. Top view of carapace; **C**. Cephalothorax and cephalic appendages of female; **D**. Cephalothorax and cephalic appendages of male; **E**. First pereopod; **F**. Second pereopod; **G**. Dactylus of third pereopod; **H**. Third pereopod; **I**. Dactylus of fifth pereopod; **J**. Fifth pereopod; **K**. Uropodal diaeresis; **L**. Preanal carina; **M**. Distal end of telson; **N**. Telson; **O**. First pleopod; **P**. Second pleopod. Drawings were made of MUMNH-CAR490-F1 (**A, B, K–N**); MUMNH-CAR616-F1 (**C, E–J**), MUMNH-CAR616-M1 (**D, O–P**). Scale bars: 1 mm (**A–D**); 0.5 mm (**E, F, H, J, N, O**); 0.25 mm (**I, P**); 0.1 mm (**G, K–M**).

***Cephalothorax and cephalic appendage*** (*n* = 7). cl 2.36–3.59 (median 2.76; Fig. 20A, B) mm, width 1.88–2.92 (median 2.21) mm. Rostrum curved down and slightly recurved near the tip (Fig. 20A, C), reaching near the end of second segment of antennular peduncle (Fig. 20B), 0.55–0.68 (median 0.63) as long as cl. Rostral formula: (3–5) 13–20/ 2–5. Antennal spine placed below inferior orbital angle (Fig. 20A, B), fused with, or slightly separated from orbital margin. Pterygostomian margin broadly round. Eye well developed, anterior end reaching to 0.37–0.72 (median 0.62) of first segment of antennular peduncle. Antennular peduncle 0.72–0.89 (median 0.81) as long as cl, first segment 1.13–1.61 (median 1.31) × as long as second segment, second segment 1.46–2.08 (median 1.69) × as long as third segment. Tooth on distolateral margin of first segment of antennular peduncle acute and well developed. Stylocerite reaching to 0.74–0.82 (median 0.79) of first segment of antennular peduncle. Scaphocerite 3.13–3.77 (median 3.36) × as long as wide, distal margin with short plumose setae.

***Branchial and mouthparts*** (*n* = 3). Podobranch on second maxilliped well developed. Third maxilliped possessed two small arthrobranchs. Pleurobranchs present on all pereopods. Third maxilliped reaching to end of second segment of antennular peduncle; ultimate segment of endopod ending with one large claw and 8–12 spiniform setae on distal 1/3 of posterior margin, 0.90–1.04 (median 0.94, *n* = 7) × as long as penultimate segment.

***Pereopods*** (*n* = 7). Epipod well developed on first two pereopods, or sometimes reduced on second pereopod, absent in last three pereopods. Chelae of P1 and P2 well developed.

P1 short (Fig. 20E) chela 2.24–2.72 (median 2.47) × as long as wide, 1.11–1.45 (median 1.18) × as long as carpus, tips of fingers rounded, with tuft of setae near tip; dactylus 1.39–1.97 (median 1.57) × as long as palm; carpus excavated distally, 2.39–3.30 (median 2.89) × as long as wide, 1.39–1.60 (median 1.46) × as long as merus; merus 2.16–3.04 (median 2.36) × as long as wide, 0.90–1.09 (median 0.97) × as long as ischium.

P2 slenderer and longer than P1 (Fig. 20F); chela 3.68–4.53 (median 3.87) × as long as wide, 0.71–0.79 (median 0.76) as long as carpus, tips of fingers rounded, with tuft of setae near tip; dactylus 1.42–1.66 (median 1.56) × as long as palm; carpus slender, 6.82–8.51 (median 7.24) × as long as wide, 1.59–1.95 (median 1.77) × as long as merus; merus 3.91–5.85 (median 4.58) × as long as wide, 0.79–1.05 (median 0.93) × as long as ischium.

P3 not sexually dimorphic (Fig. 20H); dactylus with 6–8 spiniform setae on flexor margin (Fig. 20G), 4.11–5.18 (median 4.32) × as long as wide (including terminal claw), terminating with one large claw; propodus bearing numerous spiniform setae on posterior margin, 11.19–14.68 (median 11.97) × as long as wide, 2.91–3.70 (median 3.17) × as long as dactylus; carpus possessed 3–5 setae on posterior margin of outer surface, the distal seta largest, the other setae minute, 5.13–7.45 (median 5.44) × as long as wide, 0.49–0.59 (median 0.56) as long as propodus; merus possessed three large setae on posterior margin of outer surface, 7.16–9.55 (median 9.01) × as long as wide, 1.80–2.44 (median 2.05) × as long as carpus; ischium with one spiniform seta.

P5 slender (Fig. 20J); dactylus with 58–66 spiniform setae on flexor margin (Fig. 20I), 4.70–6.87 (median 5.17) × as long as wide (including terminal claw), terminating with one large claw; propodus bearing numerous spiniform setae on posterior margin, 12.66–17.35 (median 14.47) × as long as wide, 2.39–3.28 (median 2.55) × as long as dactylus; carpus possessed 3–4 (mode 3) setae on posterior margin of outer surface, the distal seta largest, the other setae minute, margin of outer surface, 4.67–6.39 (median 5.44) × as long as wide, 0.46–0.48 (median 0.47) × as long as propodus; merus possessed three large setae on posterior margin of outer surface, 6.88–8.33 (median 7.60) × as long as wide, 1.61–2.08 (median 1.70) × as long as carpus; ischium without spiniform seta.

***Male pleopods*** (*n* = 6). Pl1 endopod subtriangular (Fig. 20O), wider proximally, 2.01–2.78 (median 2.38) × as long as width, 0.28–0.31 (median 0.30) of exopod length, without appendix interna. Pl2 appendix masculina rod-shaped (Fig. 20P), with many setae, 0.64–0.73 (median 0.69) as long as endopod; appendix interna very slender, reaching 0.68–0.75 (median 0.71) of appendix masculina length.

***Abdomen*** (*n* = 7). Sixth abdominal somite ~ 0.53–0.83 (median 0.71) of cl, 1.59–1.91 (median 1.69) × as long as fifth somite, 0.75–0.96 (median 0.83) as long as telson (Fig. 20A). Telson 2.81–4.03 (median 3.34; Fig. 20N) × as long as wide, with three or four pairs of dorsal spiniform setae and one pair of dorsolateral spiniform setae; distal margin rounded without posteromedian projection and 6–8 moveable plumose setae, the lateral pair longest (Fig. 20M). Preanal carina with a spine (Fig. 20L). Uropodal diaeresis with 10–15 moveable spiniform setae (Fig. 20K).

***Embryos***. Ovigerous females with 54–106 embryos (mean 75, *n* = 5). Size of embryos with eyes 0.59–0.70 × 0.36–0.43 mm (*n* = 50).

###### Coloration.

Body translucent, decorated with scattered blue stripes and dark spots on cephalothorax and abdomen. Rostrum translucent. Embryos brown (Fig. 14G).

###### Distribution and habitat.

Malaysia, Indonesia (Sumatra), and Thailand (Johnson 1961a; Cai et al. 2007; this study). *Caridina excavatoides* is uncommon in southern Thailand, where it can be found in the southern region of the peninsula (Fig. 5E). This species inhabits shallow water of streams or wetlands, where it lives on submerged root systems of riparian plants, typically over sandy or muddy bottom substrate.

###### Phylogenetic placement.

The molecular analyses indicated that *C. excavatoides* is monophyletic (BS = 100%, BPP = 1.00; Fig. 4) and placed it in the *C. laevis* group. It is sister to a clade of *C. temasek* and *C. tambralinga* sp. nov. (BS = 92%, BPP = 1.00).

###### Remarks.

The morphology of *C. excavatoides* in our collection fits well with its original description and the previous work in terms of the shape of the rostrum, the rostral formula: (3–5) 13–20/ 2–5 vs (3–6) 10–17/ 2–4 (Johnson 1961a; Cai et al. 2007), P2 chela (3.87–4.53× as long as wide vs 4.0 or > 4.00; Johnson 1961a; Cai et al. 2007), P2 carpus (6.82–8.51× as long as wide vs 7.00–7.80; Johnson 1961a; Cai et al. 2007), spiniform setae on P5 dactylus (58–66 vs 50–60; Johnson 1961a; Cai et al. 2007), three or four pairs of dorsal spiniform setae on telson (vs 3 pairs; Cai et al. 2007), same number of plumose setae on the distal telson (3 or 4 pairs; Johnson 1961a; Cai et al. 2007), spiniform setae on uropodal diaeresis (12–15 vs 12–13; Johnson 1961a; Cai et al. 2007), and the embryo size (0.59–0.70 × 0.36–0.43 mm vs 0.60–0.78 × 0.40–0.48 mm; Cai et al. 2007).

However, *C. excavatoides* in our collection possesses a slenderer P5 dactylus (4.70–6.87 vs 4.2; Cai et al. 2007), slenderer P5 propodus (12.66–17.35× as long as wide vs 11.00; Cai et al. 2007). The preanal carina of our specimens has a conspicuous spine (vs preanal carina low, without spine; Cai et al. 2007).

Some individuals of *C. excavatoides* are similar to *C. johnsoni*. However, they can be distinguished from each other by their rostral shape and the second pereopod. The rostrum of *C. excavatoides* is slightly to moderately curved downward, then again curved upward near the tip, whereas that of *C. johnsoni* is notably narrow, not strongly curved downward, and not recurved upward near the tip. *Caridina excavatoides* also possesses a slenderer P2 chela (3.68–4.53× as long as wide vs 2.72–3.28) and P2 carpus (6.82–8.51× as long as wide vs 5.42–6.79) than those of *C. johnsoni*.

This species also resembles *C. temasek*, but they differ by several characters. *Caridina excavatoides* possesses slenderer P3 dactylus (4.11–5.18× as long as wide vs 3.89–4.17), and longer telson (2.81–4.03× as long as wide vs 2.46–2.99). Finally, the endopod of the male Pl1 of *C. excavatoides* lacks an appendix interna, but it is present in *C. temasek*.

##### Caridina temasek

Taxon classificationAnimaliaDecapodaAtyidae

Choy & Ng, 1991

F1CD91A0-CE17-505A-9370-9A1BD06B38E9

Figs 5F, 14H, 21

Caridina temasek Choy & Ng, 1991: 265, figs 1–5. Type locality freshwater stream in Sime Road, MacRitchie catchment area, Singapore, 01°20'14"N, 103°48'47"E.
Caridina temasek — Wowor et al. 2004: 343, fig. 7i; Cai et al. 2007: 297, figs 13, 14; Cai and Naiyanetr 2024: 785.
Caridina cf. babaulti [non Bouvier, 1918] — Johnson 1961a: 136, figs 21–24.
Caridina sp. — Ng 1990: 200; Ng, 1991: 120.

###### Material examined.

Five lots (see Suppl. material 1: Data S1).

###### Description.

**Figure 21.**
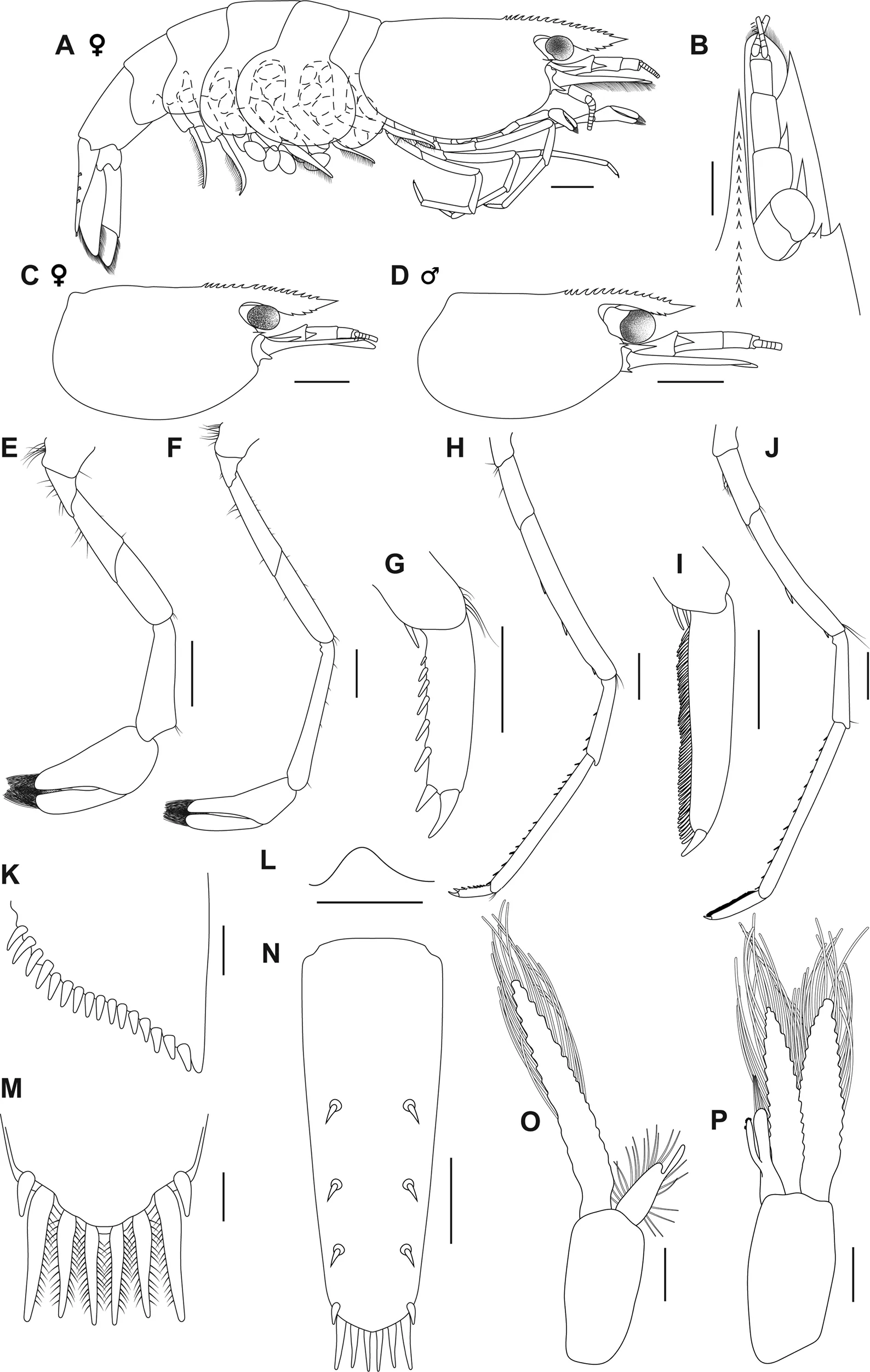
*Caridina temasek*. **A**. Ovigerous female; **B**. Top view of carapace; **C**. Cephalothorax and cephalic appendages of female; **D**. Cephalothorax and cephalic appendages of male; **E**. First pereopod; **F**. Second pereopod; **G**. Dactylus of third pereopod; **H**. Third pereopod; **I**. Dactylus of fifth pereopod; **J**. Fifth pereopod; **K**. Uropodal diaeresis; **L**. Preanal carina; **M**. Distal end of telson; **N**. Telson; **O**. First pleopod; **P**. Second pleopod. Drawings were made of MUMNH-CAR835-F1 (**A, B, E–N**); MUMNH-CAR835-F2 (**C**), MUMNH-CAR835-M1 (**D, O, P**). Scale bars: 1 mm (**A–D**); 0.5 mm (**E, F, H, J, N**); 0.25 mm (**G, I, O, P**); 0.1 mm (**K–M**).

***Cephalothorax and cephalic appendage*** (*n* = 6). cl 3.03–3.61 (median 3.44; Fig. 21A, C) mm, width 2.30–3.04 (median 2.73) mm. Rostrum slightly curved down, wider near middle length and tapering toward the tip (Fig. 21A, C), frequently reaching slightly beyond the end of second segment of antennular peduncle (Fig. 21B), rarely reaching to the end of third segment of antennular peduncle, 0.57–0.64 (median 0.61) as long as cl. Rostral formula: (4–7) 14–18/ 0–5. Antennal spine placed below inferior orbital angle, fused with, or slightly separated from orbital margin. Pterygostomian margin broadly round. Eye well developed, anterior end reaching to 0.42–0.64 (median 0.48) of first segment of antennular peduncle. Antennular peduncle 0.67–0.79 (median 0.74; Fig. 21A–C) as long as cl, first segment 1.77–2.07 (median 2.03) × as long as second segment, second segment 1.22–1.55 (median 1.42) × as long as third segment. Tooth on distolateral margin of first segment of antennular peduncle acute and well developed. Stylocerite reaching to 0.71–0.90 (median 0.79) of first segment of antennular peduncle. Scaphocerite 2.99–3.25 (median 3.19) × as long as wide, distal margin with short plumose setae.

***Branchial and mouthparts*** (*n* = 3). Podobranch on second maxilliped well developed. Third maxilliped possessed two small arthrobranchs. Pleurobranchs present on all pereopods. Third maxilliped reaching to end of second segment of antennular peduncle; ultimate segment of endopod ending with one large claw and seven to nine spiniform setae on distal 1/3 of posterior margin, 0.90–1.00 (median 0.96, *n* = *5*) × as long as penultimate segment.

***Pereopods*** (*n* = 6). Epipod present on first two pereopods, reduced or absent on third pereopod, and absent in last two pereopods. Chelae of P1 and P2 well developed.

P1 short (Fig. 21E); chela 2.15–2.63 (median 2.22) × as long as wide, 0.99–1.25 (median 1.12) × as long as carpus, tips of fingers rounded, with tuft of setae near tip; dactylus 1.26–1.81× as long as palm; carpus hardly excavated distally, 2.65–3.07 (median 2.97) × as long as wide, 1.19–1.48 (median 1.36) × as long as merus; merus 2.38–3.06 (median 2.72) × as long as wide, 0.89–1.04 (median 0.92) × as long as ischium.

P2 slenderer, longer than P1 (Fig. 21F); chela 2.89–4.00 (median 3.13) × as long as wide, 0.60–0.78 (median 0.70) as long as carpus, tips of fingers rounded, with tuft of setae near tip; dactylus 1.06–2.09 (median 1.90) × as long as palm; carpus slender, 6.04–6.91 (median 6.31) × as long as wide, 1.51–1.77 (median 1.63) × as long as merus; merus 4.08–5.48 (median 4.86) × as long as wide, 0.86–1.02 (median 0.95) × as long as ischium.

P3 not sexually dimorphic (Fig. 21H); dactylus with 5–8 spiniform setae on flexor margin (Fig. 21G), 3.89–4.17 (median 3.93) × as long as wide (including terminal claw), terminating with one large claw; propodus bearing numerous spiniform setae on posterior margin, 10.35–14.54 (median 11.32) × as long as wide, 3.18–4.37 (median 3.60) × as long as dactylus; carpus possessed three setae on posterior margin of outer surface, the distal seta largest, the other setae minute, 5.26–6.76 (median 5.74) × as long as wide, 0.50–0.61 (median 0.57) as long as propodus; merus possessed three large setae on posterior margin of outer surface, 7.51–8.24× as long as wide, 1.78–2.23 (median 2.04) × as long as carpus.

P5 slender (Fig. 21J); dactylus with 59–80 spiniform setae on flexor margin (Fig. 21I), 3.95–5.96 (median 4.61) × as long as wide (including terminal claw), terminating with one large claw; propodus bearing numerous spiniform setae on posterior margin, 12.05–15.44 (median 13.58) × as long as wide, 2.80–3.44 (median 2.99) × as long as dactylus; carpus possessed three or four (mode 3) setae on posterior margin of outer surface, the distal seta largest, the other setae minute, 4.84–5.36× as long as wide, 0.44–0.52 (median 0.48) as long as propodus; merus 7.03–7.84 (median 7.14) × as long as wide, 1.57–1.76 (median 1.61) × as long as carpus.

***Male pleopods*** (*n* = 5). Pl1 endopod subtriangular (Fig. 21O), wider proximally, 1.84–2.65 (median 2.01) × as long as wide, 0.26–0.32 (median 0.29) of exopod length, with a long appendix interna arising near distal end. Pl2 appendix masculina rod-shaped (Fig. 21P) with numerous long setae, 0.46–0.57 (median 0.51) as long as endopod; appendix interna very slender, reaching 0.84–0.96 (median 0.91) of appendix masculina length.

***Abdomen*** (*n* = 6). Sixth abdominal somite ~ 0.51–0.58 (median 0.53) of cl, 1.28–1.61 (median 1.55) × as long as fifth somite, 0.82–0.95 (median 0.86) as long as telson (Fig. 21A). Telson 2.46–2.99 (median 2.85) × as long as wide (Fig. 21N), with three or four pairs of dorsal spiniform setae and one pair of dorsolateral spiniform setae; distal margin convex to broadly rounded, without posteromedian projection, with 6–9 moveable plumose setae, the lateral pair longest (Fig. 21M). Preanal carina low, with or without a spine (Fig. 21L). Uropodal diaeresis with 12–16 moveable spiniform setae (Fig. 21K).

***Embryos***. Ovigerous females with 70–110 embryos (mean = 86, *n* = 5). Size of embryos with eyes 0.59–0.67 × 0.34–0.41 mm (*n* = 50).

###### Coloration.

Body slightly translucent, with blue and cream stripes on dorsal and dark spots throughout the body. Rostrum translucent. Embryos brown (Fig. 14H).

###### Distribution and habitat.

Singapore, Malaysia, Indonesia (Borneo), and Thailand (Cai et al. 2007; Cai and Naiyanetr 2024; this study). In southern Thailand, *C. temasek* can be found in the southern region of the peninsula (Fig. 5F). This species inhabits streams and lowland rivers and can be found living on aquatic vegetation along the banks.

###### Phylogenetic placement.

The molecular analyses indicated that *C. temasek* is monophyletic (BS = 95%, BPP = 0.99; Fig. 4). It was placed as a sister clade to *C. tambralinga* sp. nov. with high nodal support (BS = 100%, BPP = 1.00).

###### Remarks.

This species has been reported previously from southern Thailand in Songkhla and Narathiwat provinces (Cai et al. 2007). The morphology of our specimens aligns with the original description and those reported in the previous works, including the shape of rostrum (slightly sigmoid and reaching slightly beyond the end of second segment of antennular peduncle; Cai et al. 2007), the rostral formula: (4–6) 14–18/ 3–5 vs (4–6) 14–18/ 2–6 (Choy and Ng 1991), P1 chela (2.15–2.63× as long as wide vs 2.4–2.5; Choy and Ng 1991), P2 chela (2.89–4.00× as long as wide vs 3.0–3.8; Choy and Ng 1991; Cai et al. 2007), spiniform setae on P3 dactylus (5–8 vs 6–10; Choy and Ng 1991; Cai et al. 2007), spiniform setae on P5 dactylus (59–80 vs 55–72; Choy and Ng 1991; Cai et al. 2007), the presence of appendix interna on endopod of male first pleopod (Choy and Ng 1991; Cai et al. (2007), the same number of spiniform setae on uropodal diaeresis (12–16; Choy and Ng 1991; Cai et al. 2007). However, the observed embryo size is smaller than that of previous reports (0.59–0.67 × 0.34–0.41 mm vs 0.70–0.85 × 0.44–0.54 mm; Choy and Ng 1991; Cai et al. 2007).

*Caridina temasek* can be distinguished clearly from other species in the *C. laevis* species group by the presence of an appendix interna on the endopod of male first pleopod.

##### Caridina cf. rangoona

Taxon classificationAnimaliaDecapodaAtyidae

Cai & Ng, 2000

FD8AE1BD-5260-5D8F-B941-1B6DDA0AEF21

Figs 5E, 22, 23A

Caridina rangoona Cai & Ng, 2000: 939, fig. 6. Type locality 17°09.97'N, 96°99.20'E, Win Paw Hta River, near border between Pegu (Bago) and Yangon, Yangon State, Myanmar.
Caridina rangoona — Cai and Naiyanetr 2024: 784.

###### Material examined.

Eleven lots (see Suppl. material 1: Data S1).

###### Description.

**Figure 22.**
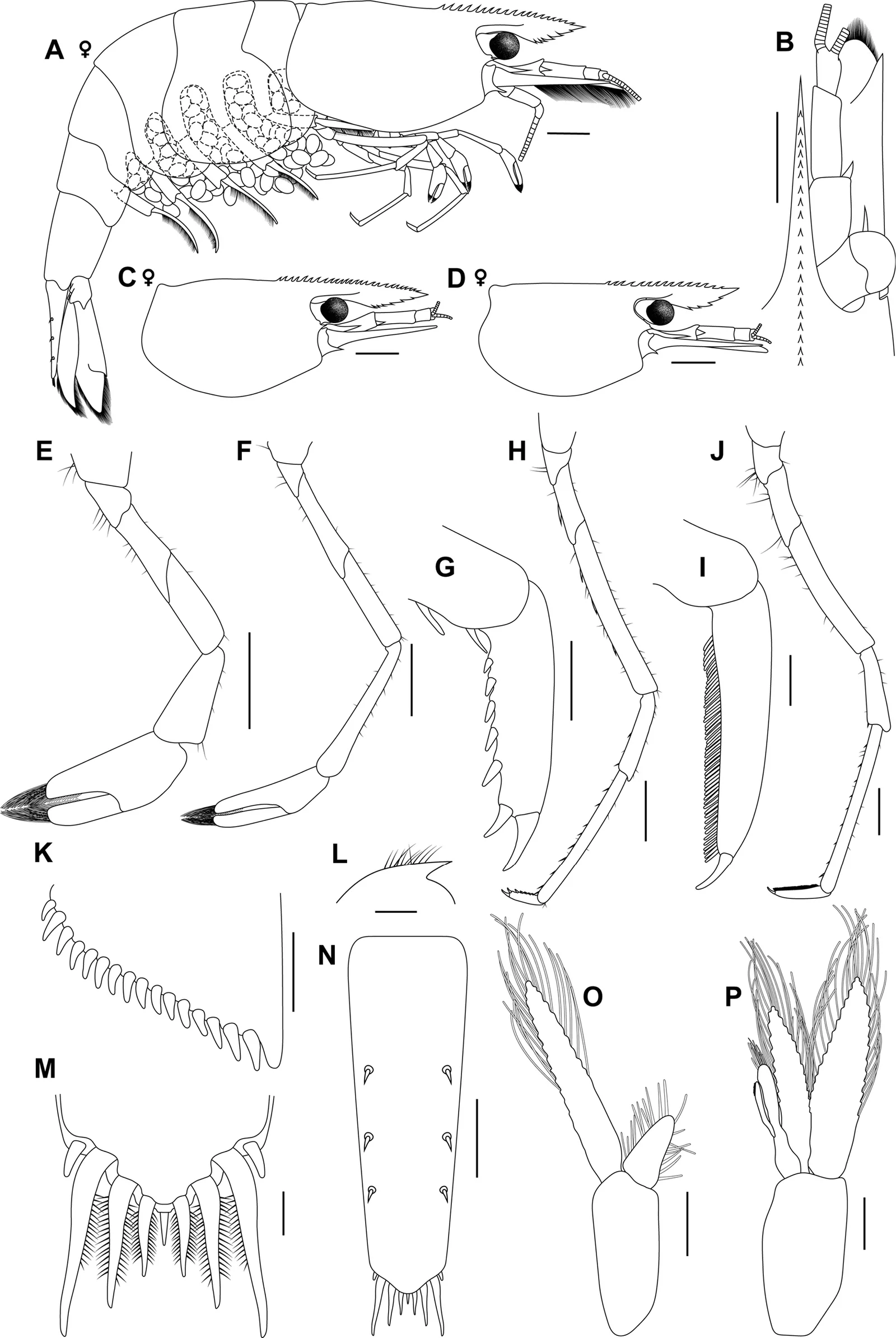
*Caridina cf. rangoona*. **A**. Ovigerous female; **B**. Top view of carapace; **C**. Cephalothorax and cephalic appendages of female; **D**. Cephalothorax and cephalic appendages of male; **E**. First pereopod; **F**. Second pereopod; **G**. Dactylus of third pereopod; **H**. Third pereopod; **I**. Dactylus of fifth pereopod; **J**. Fifth pereopod; **K**. Uropodal diaeresis; **L**. Preanal carina; **M**. Distal end of telson; **N**. Telson; **O**. First pleopod; **P**. Second pleopod. Drawings were made of MUMNH-CAR154-D1 (**A, B, E–N**); MUMNH-CAR154-F1 (**C**); MUMNH-CAR434-F1 (**D**); MUMNH-CAR154-M1 (**O, P**). Scale bars: 1 mm (**A–D**); 0.5 mm (**E, F, H, J, N**); 0.25 mm (**O, P**); 0.1 mm (**G, I, K–M**).

***Cephalothorax and cephalic appendage*** (*n* = 5). cl 2.70–4.00 (median 3.25; Fig. 22A, C, D) mm, width 2.59–3.00 (median 2.66) mm. Rostrum straight or slightly curved down anteriorly (Fig. 22A–D), reaching between the third segment of antennular peduncle or slightly beyond (Fig. 22B), 0.57–0.75 (median 0.72) as long as cl. Rostral formula: (4–6) 17–22/ 4–7, dorsal teeth usually placed throughout dorsal margin. Antennal spine placed below inferior orbital angle, spine fused with or slightly separated from orbital margin. Pterygostomian margin round. Eye well developed, anterior end reaching to 0.46–0.66 (median 0.59) of first segment of antennular peduncle. Antennular peduncle 0.63–0.84 (median 0.76; Fig. 22A–D) as long as cl, first segment 1.38–1.65 (median 1.57) × as long as second segment, second segment 1.49–2.33 (median 1.74) × as long as third segment. Tooth on distolateral margin of first segment of antennular peduncle acute and well developed. Stylocerite reaching to 0.71–0.82 (median 0.80) of first segment of antennular peduncle. Scaphocerite 3.24–3.57 (median 3.49) × as long as wide, distal margin with short plumose setae.

***Branchial and mouthparts*** (*n* = 3). Podobranch on second maxilliped well developed. Third maxilliped possessed two small arthrobranchs. Pleurobranchs present on all pereopods. Third maxilliped reaching to end of second segment of antennular peduncle; ultimate segment of endopod ending with one large claw and seven or eight spiniform setae on distal 1/3 of posterior margin, 0.87–0.93 (median 0.89, *n* = *5*) as long as penultimate segment.

***Pereopods*** (*n* = 5). Epipods present on first two pereopods, reduced on third pereopod, absent in last two pereopods. Chelae of P1 and P2 well developed.

P1 short (Fig. 22E); chela 2.27–2.82 (median 2.50) × as long as wide, 1.25–1.36 (median 1.33) × as long as carpus, tips of fingers rounded, with tufts of setae near tip; dactylus 1.22–1.63 (median 1.55) × as long as palm; carpus stout, excavated distally, 2.11–2.92 (median 2.56) × as long as wide, 1.03–1.23 (median 1.15) × as long as merus; merus 2.64–3.46 (median 2.83) × as long as wide, 0.97–1.15 (median 1.04) × as long as ischium.

P2 slenderer, longer than P1 (Fig. 22F); chela 3.05–3.83 (median 3.51) × as long as wide, 0.76–0.83 (median 0.78) as long as carpus, tips of fingers rounded, with tufts of setae near tip; dactylus 1.40–1.73 (median 1.55) × as long as palm; carpus slender, 5.93–7.39 (median 6.77) × as long as wide, 1.32–1.52 (median 1.40) × as long as merus; merus 4.79–5.54 (median 5.16) × as long as wide, 0.88–1.12 (median 0.94) × as long as ischium.

P3 not sexually dimorphic (Fig. 22H); dactylus with 5–8 spiniform setae on flexor margin (Fig. 22G), 3.72–4.60 (median 4.15) × as long as wide (including terminal claw), terminating with one large claw; propodus bearing numerous spiniform setae on posterior margin, 9.23–12.63 (median 11.79) × as long as wide, 3.59–4.35 (median 4.17) × as long as dactylus; carpus possessed three setae on posterior margin of outer surface, the distal seta largest, the other setae minute, 5.43–6.04 (median 5.67) × as long as wide, 0.56–0.61 (median 0.57) as long as propodus; merus possessed three large setae on posterior margin of outer surface, 7.26–9.47 (median 8.38) × as long as wide, 1.84–2.02 (median 1.97) × as long as carpus; ischium with one spiniform seta.

P5 slender (Fig. 22J); dactylus with 51–58 spiniform setae on flexor margin (Fig. 22I), 4.73–5.95 (median 5.32) × as long as wide (including terminal claw), terminating with one large claw; propodus bearing numerous spiniform setae on posterior margin, 12.19–15.24 (median 14.54) × as long as wide, 2.69–3.33 (median 2.95) × as long as dactylus; carpus possessed three setae on posterior margin of outer surface, the distal seta largest, the other setae minute, 4.63–6.19 (median 5.59) × as long as wide, 0.48–0.54 (median 0.51) as long as propodus; merus possessed three large setae on posterior margin of outer surface, 6.29–8.32 (median 7.44) × as long as wide, 1.51–1.75 (median 1.62) × as long as carpus; ischium without a spiniform seta.

***Male pleopods*** (*n* = 8). Pl1 endopod subtriangular (Fig. 22O), wider proximally, 2.08–2.89 (median 2.55) × as long as wide, 0.30–0.39 (median 0.35) of exopod length, without appendix interna. Pl2 appendix masculina rod-shaped (Fig. 22P), slender, with many setae, 0.50–0.73 (median 0.62) × as long as endopod; appendix interna reaching 0.76–0.90 (median 0.85) of appendix masculina length.

***Abdomen*** (*n* = 5). Sixth abdominal somite ~ 0.52–0.68 (median 0.59) of cl, 1.52–1.81 (median 1.69) × as long as fifth somite, 0.83–0.92 (median 0.89) as long as telson (Fig. 22A). Telson 3.36–3.97 (median 3.77; Fig. 22N) × as long as wide, with three pairs of dorsal spiniform setae and one pair of dorsolateral spiniform setae; distal margin rounded without posteromedian projection, with one short median moveable seta and six moveable plumose setae, the lateral pair longest, the intermediate pair shortest (Fig. 22M). Preanal carina round with a few setae and a spine (Fig. 22L). Uropodal diaeresis with 14–20 moveable spiniform setae (Fig. 22K).

***Embryos***. Ovigerous females with 195–370 embryos (mean = 300, *n* = 5). Size of embryos with eyes 0.37–0.46 × 0.24–0.31 mm (*n* = 50).

###### Coloration.

**Figure 23.**
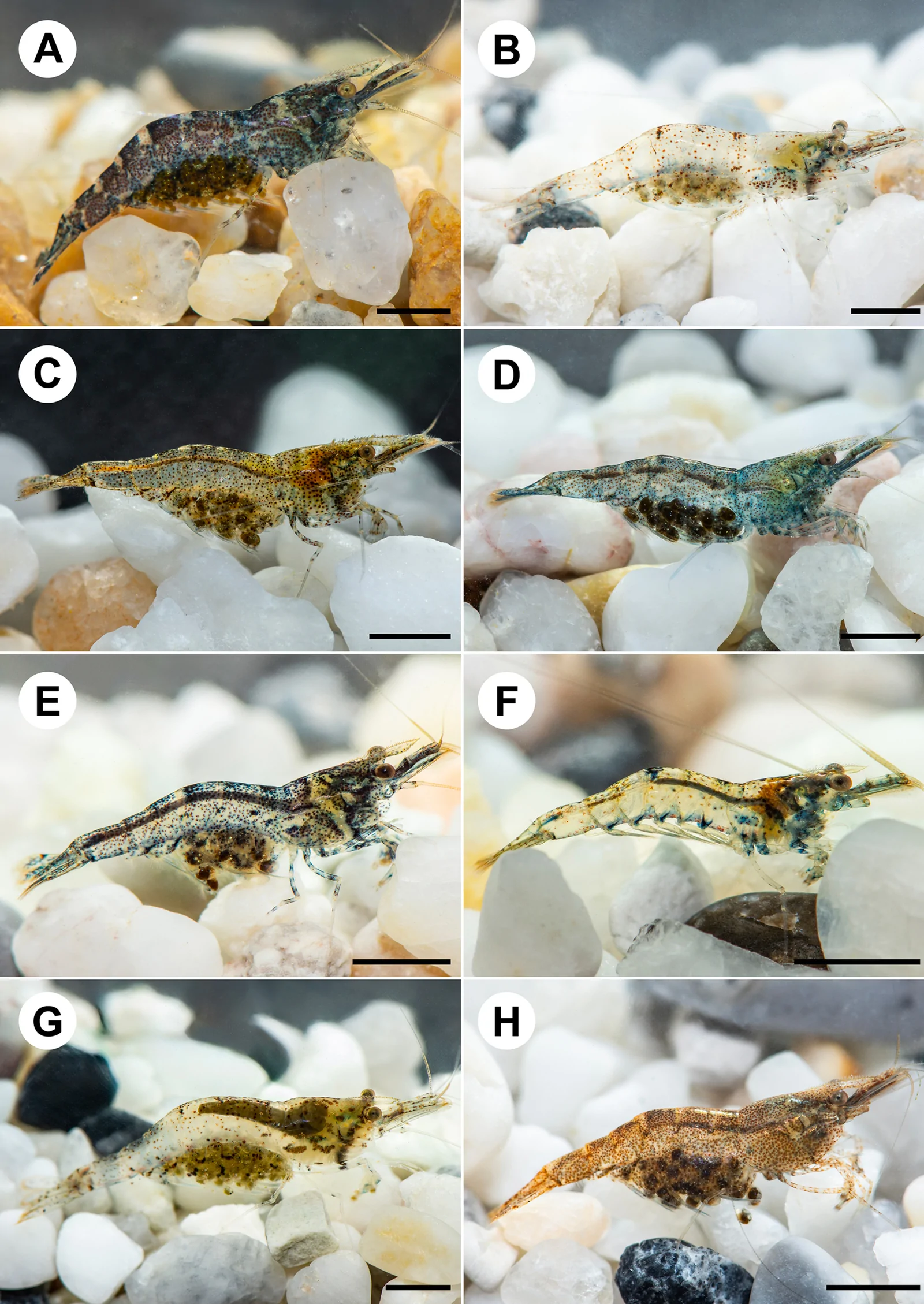
Living specimens of *Caridina* species. **A**. *C. cf. rangoona*, specimen MUMNH-CAR434-P1 from Phang Nga Province, Takuatung District, Khum Mut Stream; **B**. *C. johnsoni*, specimen MUMNH-CAR471-P1 from Surat Thani Province, Mueang District, Phunphin Stream; **C, D**. *C. nangroma* sp. nov.; **C**. paratype MUMNH-CAR404-P1 and **D**. specimen MUMNHCAR474-P1 from Chumphon Province, Lamae District, Lamae Stream; **E, F**. *C. srivijaya* sp. nov., paratypes; **E**. MUMNH-CAR502-P1; **F**. MUMNH-CAR502-M1; **G**. *C. takkola* sp. nov., paratype MUMNH-CAR469-P1; **H**. *C. tambralinga* sp. nov., specimen MUMNH-CAR489-P1 from Songkhla Province, Ranot District, Thale Noi Non-Hunting Area. Scale bar: 3 mm.

The body dark cyan, greyish brown, or dark grey, with grayish green or grey stripes and numerous dark spots. Rostrum quite translucent or thistle green. Cephalic region decorated with light stripes. Each abdominal somite dorsally decorated with light transverse bands. Embryos brown (Fig. 23A).

###### Distribution and habitat.

*Caridina cf. rangoona* is distributed along the western side of southern Thailand (Andaman Sea; Fig. 5E). This species inhabits various habitats, such as streams, ponds, lakes, lowland rivers, and brackish water, and can be found living on aquatic vegetation along the banks.

###### Phylogenetic placement.

The molecular analyses indicated *C. cf. rangoona* as monophyletic (BS = 100%, BPP = 0.99; Fig. 4), and placed it in the *C. laevis* group, being sister to a clade of *C. srivijaya* sp. nov. and *C. nangroma* sp. nov. (BS = 100%, BPP = 1.00; Fig. 4).

###### Remarks.

Our specimens are morphologically closest to *C. rangoona* described by Cai and Ng (2000), including P1 dactylus (1.14–1.63× as long as palm vs 1.5), P1 carpus (2.11–2.92 as long as wide vs 2.3), P3 dactylus (3.72–4.60× as long as wide vs 4.3), and P5 dactylus (4.73–5.95× as long as wide vs 5.0). However, the morphology of *C. cf. rangoona* in our collection shows some differences from its original description by Cai and Ng (2000). The rostrum of our specimens is lanciform and often equal or reaching slightly beyond the end of third segment of antennular peduncle (vs reaching near the end of second segment of antennular peduncle). The preanal carina of *C. cf. rangoona* in our collection possesses a prominent spine (vs absent). P5 dactylus possesses fewer spiniform setae (51–57 vs 65–67). Embryo size is relatively smaller than the original description (0.33–0.46 × 0.23–0.29 mm vs 0.6–0.7 × 0.31–0.40 mm). While this morphological difference could be attributed to either intraspecific variation or distinct lineages, the present study cannot confirm a definite taxonomic status because the molecular data for the *C. rangoona* type series is currently unavailable. Consequently, we assign these specimens as *Caridina cf. rangoona* pending further taxonomic revision and molecular evidence.

##### Caridina johnsoni

Taxon classificationAnimaliaDecapodaAtyidae

Cai, Ng & Choy, 2007

4516C949-DABA-5247-A930-263BE1A33402

Figs 5F, 23B, 24

Caridina johnsoni Cai et al., 2007: 301, figs 15, 16. Type locality Lower Peirce Reservoir, north arm, Singapore.
Caridina johnsoni — Cai and Naiyanetr 2024: 785.
Caridina propinqua [non De Man, 1908] — Ng 1990: 200 (in part).
Caridina tonkinensis [non Bouvier, 1919] — Johnson 1961a: 133, figs 16–20; Johnson 1965: 8; Ng 1990: 200; Ng and Choy 1990: 16.
Caridina aff. tonkinensis [non Bouvier, 1919] — Wowor et al. 2004: 343, fig. 7m–o.

###### Material examined.

28 lots (see Suppl. material 1: Data S1).

###### Description.

**Figure 24.**
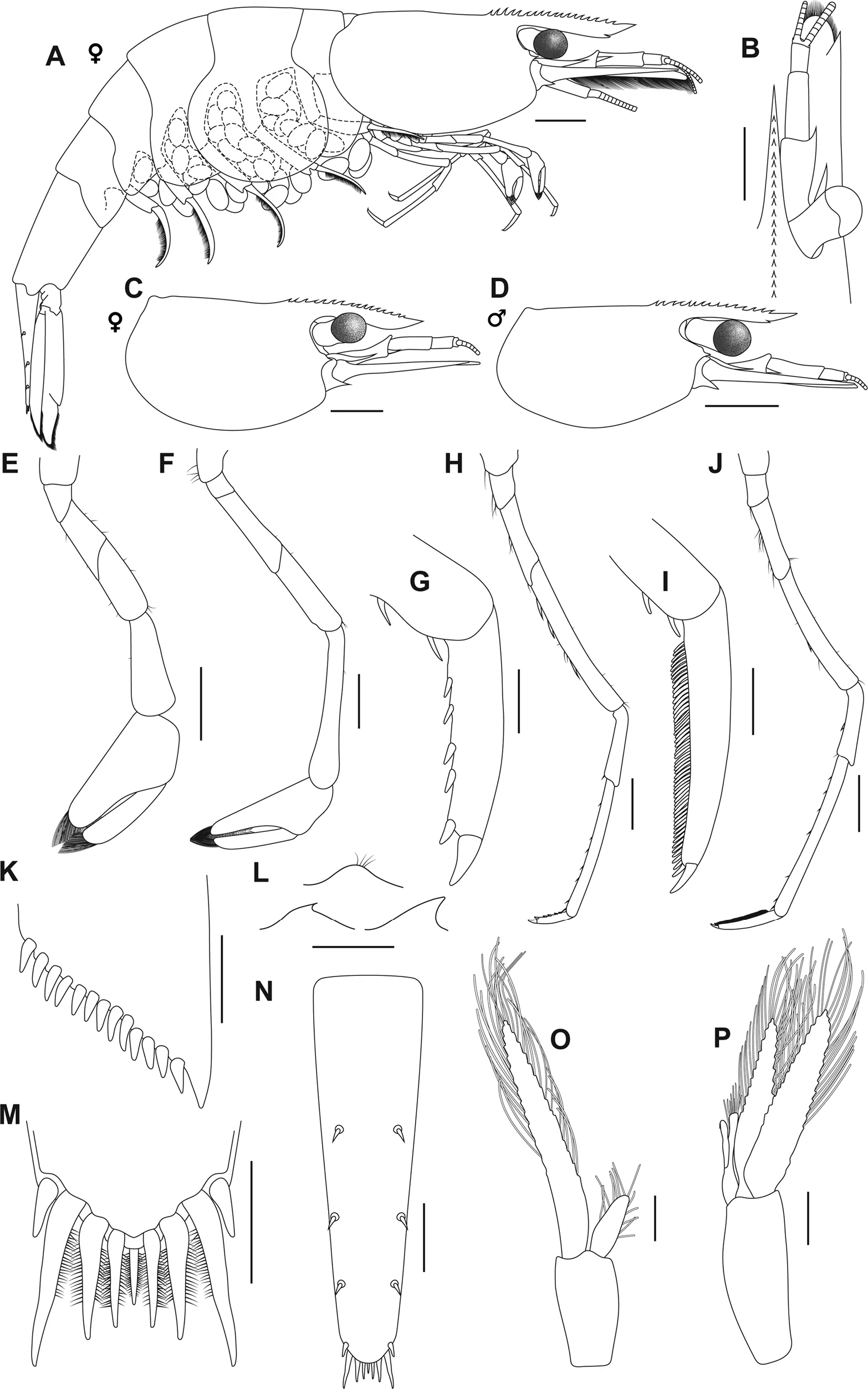
*Caridina johnsoni*. **A**. Ovigerous female; **B**. Top view of carapace; **C**. Cephalothorax and cephalic appendages of female; **D**. Cephalothorax and cephalic appendages of male; **E**. First pereopod; **F**. Second pereopod; **G**. Dactylus of third pereopod; **H**. Third pereopod; **I**. Dactylus of fifth pereopod; **J**. Fifth pereopod; **K**. Uropodal diaeresis; **L**. Preanal carina; **M**. Distal end of telson; **N**. Telson; **O**. First pleopod; **P**. Second pleopod. Drawings were made of MUMNH-CAR470-D1 (**A, B, E–N**); MUMNH-CAR383-D1 (**L**; without spine); MUMNH-CAR470-D2 (**C**); MUMNH-CAR470-M1 (**D, O, P**). Scale bars: 1 mm (**A–D**); 0.5 mm (**E, F, H, J, N**); 0.25 mm (**L, O, P**); 0.1 mm (**G, I, K**).

***Cephalothorax and cephalic appendage*** (*n* = 10). cl 2.83–3.34 (median 3.10; Fig. 24A, C) mm, width 2.02–2.86 (median 2.41) mm. Rostrum relatively narrow (Fig. 24A, C), slightly curved downward, often reaching near the 1/2-length of second segment of antennular peduncle (Fig. 24B), 0.62–0.75 (median 0.68) as long as cl. Rostral formula: (2–5) 13–20/ 0–3. Antennal spine placed below inferior orbital angle, fused with, or slightly separated from orbital margin. Pterygostomian margin round. Eye well developed, anterior end reaching to 0.49–0.68 (median 0.61) of first segment of antennular peduncle. Antennular peduncle 0.73–0.91 (median 0.83; Fig. 24A–C) as long as cl, first segment 1.40–2.12 (median 1.76) × as long as second segment, second segment 1.47–2.10 (median 1.69) × as long as third segment. Tooth on distolateral margin of first segment of antennular peduncle acute and well developed. Stylocerite reaching to 0.73–0.88 (median 0.77) of first segment of antennular peduncle. Scaphocerite 3.03–3.70 (median 3.46) × as long as wide, distal margin with short plumose setae.

***Branchial and mouthparts*** (*n* = 3). Podobranch on second maxilliped well developed. Third maxilliped possessed two small arthrobranchs. Pleurobranchs present on all pereopods. Third maxilliped reaching to end of second segment of antennular peduncle; ultimate segment of endopod ending with one large claw and 7–12 spiniform setae on distal 1/3 of posterior margin, 0.73–0.88 (median 0.80, *n* = 10) as long as penultimate segment.

***Pereopods*** (*n* = 10). Epipods well developed on first pereopod, slightly reduced in second pereopod, strongly reduced in third pereopod, and absent in last two pereopods. Chelae of P1 and P2 well developed.

P1 short (Fig. 24E); chela 1.94–2.54 (median 2.23) × as long as wide, 1.16–1.47 (median 1.30) × as long as carpus, tips of fingers rounded, with tuft of setae near tip; dactylus 1.38–1.92 (median 1.60) × as long as palm; carpus excavated distally, 2.20–3.45 (median 2.70) × as long as wide, 1.06–1.39 (median 1.25) × as long as merus; merus 2.33–2.85 (median 2.57) × as long as wide, 0.95–1.12 (median 1.01) × as long as ischium.

P2 slenderer, longer than P1 (Fig. 24F); chela 2.72–3.28 (median 3.00) × as long as wide, 0.72–0.82 (median 0.79) × as long as carpus, tips of fingers rounded, with tuft of setae near tip; dactylus 1.26–1.65 (median 1.56) × as long as palm; carpus slender, 5.42–6.79 (median 6.00) × as long as wide, 1.21–1.91 (median 1.54) × as long as merus; merus 3.44–5.35 (median 4.57) × as long as wide, 0.92–1.10 (median 1.02) × as long as ischium.

P3 not sexually dimorphic (Fig. 24H); dactylus with 4–6 (mode 6) spiniform setae on flexor margin (Fig. 24G), 3.33–4.88 (median 4.56, *n* = 9) × as long as wide (including terminal claw), terminating with one large claw; propodus bearing numerous spiniform setae on posterior margin, 9.66–14.09 (median 11.87, *n* = 9) × as long as wide, 3.10–3.95 (median 3.38, *n* = 9) × as long as dactylus; carpus possessed four setae on posterior margin of outer surface, the distal seta largest, the other setae minute, 4.94–6.26 (median 5.64, *n* = 9) × as long as wide, 0.52–0.61 (median 0.55, *n* = 9) as long as propodus; merus possessed four large setae on posterior margin of outer surface, 7.93–9.27 (median 8.70, *n* = 9) × as long as wide, 2.02–2.29 (median 2.10, *n* = 9) × as long as carpus; ischium with one spiniform seta.

P5 slender (Fig. 24J); dactylus with 50–62 spiniform setae on flexor margin (Fig. 24I), 4.35–6.34 (median 5.34) × as long as wide (including terminal claw), terminating with one large claw; propodus bearing numerous spiniform setae on posterior margin, 12.39–16.94 (median 14.90) × as long as wide, 2.52–2.83 (median 2.73) × as long as dactylus; carpus possessed four setae on posterior margin of outer surface, the distal seta largest, the other setae minute, 4.13–5.85 (median 5.03) × as long as wide, 0.42–0.49 (median 0.47) as long as propodus; merus possessed two large setae on posterior margin of outer surface, 7.17–8.62 (median 7.71) × as long as wide, 1.61–1.86 (median 1.73) × as long as carpus; ischium without a spiniform seta.

***Male pleopods*** (*n* = 10). Pl1 endopod subtriangular (Fig. 24O), wider proximally, 2.27–2.92 (median 2.61) × as long as wide, 0.23–0.27 (median 0.26) of exopod length, without appendix interna. Pl2 appendix masculina rod-shaped (Fig. 24P), with many setae, 0.48–0.61 (median 0.50) as long as endopod; appendix interna very slender, reaching 0.79–0.99 (median 0.87) of appendix masculina length.

***Abdomen*** (*n* = 10). Sixth abdominal somite ~ 0.62–0.81 (median 0.68) of cl, 1.66–2.03 (median 1.85) × as long as fifth somite, 0.80–0.96 (median 0.87) as long as telson (Fig. 24A). Telson 3.16–4.15 (median 3.60; Fig. 24L) × as long as wide, with three pairs of dorsal spiniform setae and one pair of dorsolateral spiniform setae; distal margin rounded without posteromedian projection, with one median plumose seta and six or seven moveable plumose setae, the lateral pair longest (Fig. 24M). Preanal carina various in shape (Fig. 24L): (1) blunt with a few setae, (2) stout with a spine, and (3) subtriangular. Uropodal diaeresis with 11–15 moveable spiniform setae (Fig. 24K).

***Embryos***. Ovigerous females with 77–118 embryos (mean = 91, *n* = 5). Size of embryos with eyes 0.62–0.73 × 0.38–0.45 mm (*n* = 50).

###### Coloration.

Body translucent, with brown stripes and dark spots. Rostrum translucent. Embryos brown (Fig. 23B).

###### Distribution and habitat.

Vietnam, Thailand, Singapore, and Indonesia (Cai et al. 2007; Cai and Naiyanetr 2024; this study). *Caridina johnsoni* is widespread throughout southern Thailand (Fig. 5F). This species inhabits various habitats such as lowland rivers, brackish water, streams, reservoirs, ponds, and lakes. It can be found living on aquatic vegetation along the banks.

###### Phylogenetic placement.

The molecular analyses showed *C. johnsoni* as monophyletic (BS = 98%, BPP = 1.00; Fig. 4), being sister to *C. takkola* sp. nov. (BS = 100%, BPP = 1.00; Fig. 4).

###### Remarks.

While the original description of *C. johnsoni* states that its preanal carina lacks a spine (Cai et al. 2007), our observations revealed morphological variation in this feature. Some individuals from southern Thailand possessed a spine on the preanal carina, whereas others from the same geographic area exhibited an unarmed preanal carina. Molecular analyses confirmed that these two forms represent the same species.

*Caridina johnsoni* in our collection also shows some variation with respect to the original description by Cai et al. (2007) by having slightly stouter P1 chela (1.94–2.54× as long as wide vs 2.6), stouter P2 chela (2.72–3.28× as long as wide vs 4), fewer plumose setae on telson (3–4 pairs vs 4–8 pairs), and slightly larger embryos (0.62–0.73 × 0.38–0.45 mm vs 0.6 × 0.4 mm). Other than those characters, our specimens were morphologically consistent with the original description, which are the feature of rostrum (narrow, reaching between the second segment of antennular peduncle), rostral formula: (2–5) + 13–20 / 0–3 vs (3–4) + 10–17/ 0–4), P1 dactylus (1.38–1.92× as long as palm vs 1.5), P1 carpus (2.20–3.45× as long as wide vs 2.9), P2 dactylus (1.26–1.65× as long as palm vs 1.6), P2 carpus (3.44–5.35× as long as wide vs 4), spiniform setae on P3 dactylus (4–6 vs 6–7), P3 dactylus (3.33–4.88× as long as wide vs 4.6), spiniform setae on P5 dactylus (50–62 vs 55–69), P5 dactylus (4.35–6.34× as long as wide vs 5.2), sixth abdominal somite (0.62–0.81 as long as cl vs 0.7), telson (3.16–4.15× as long as wide vs 3.4), spiniform setae on uropodal diaeresis (11–15 vs 11–13).

##### Caridina nangroma sp. nov.

Taxon classificationAnimaliaDecapodaAtyidae

E055ABDE-D908-54CF-BBBB-5D39E41F8372

https://zoobank.org/392C9085-CF89-491D-A24C-0209A6B3C2FF

**Figure 25.**
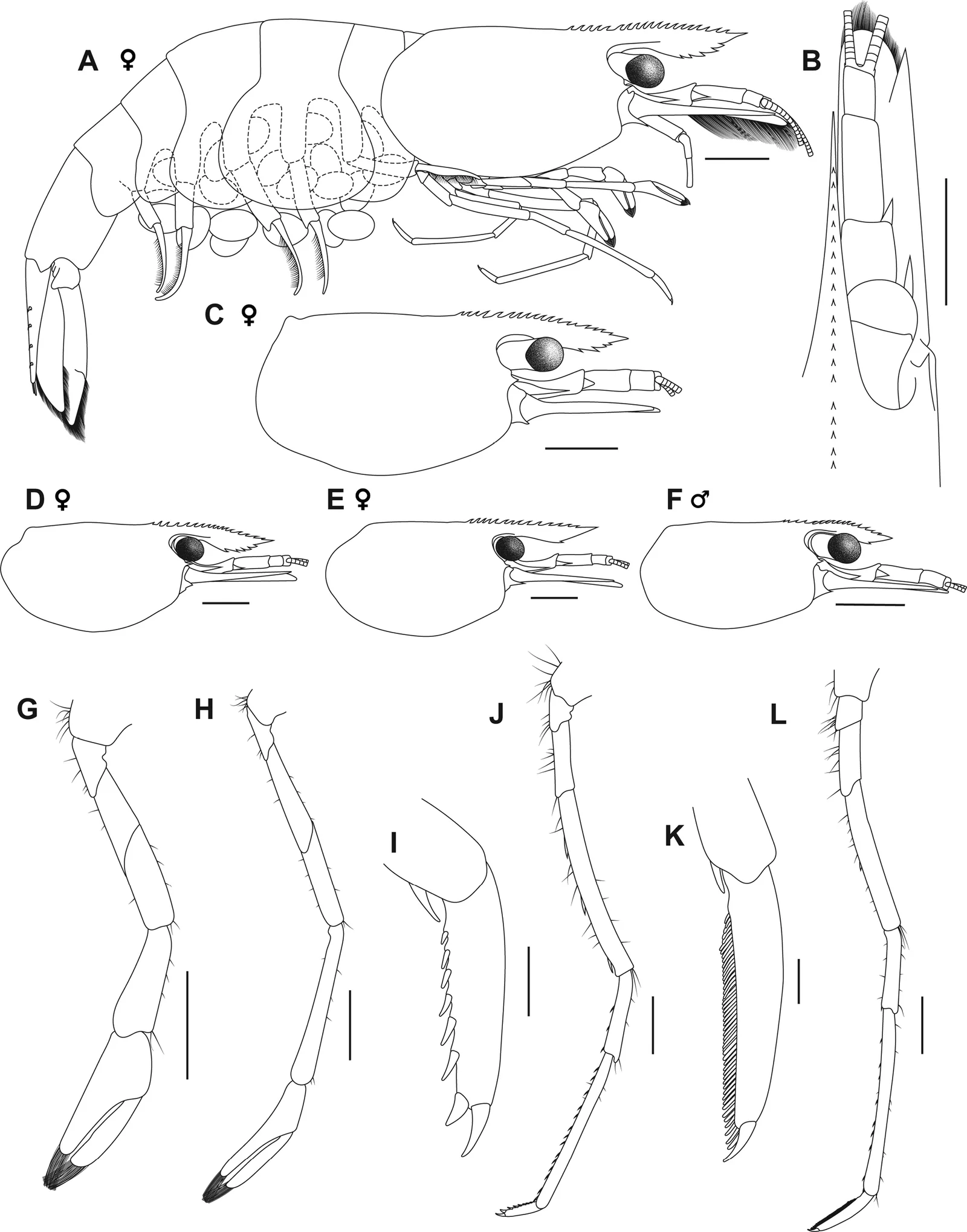
*Caridina nangroma* sp. nov. **A**. Ovigerous female; **B**. Top view of carapace; **C–E**. Cephalothorax and cephalic appendages of female; **F**. Cephalothorax and cephalic appendages of male; **G**. First pereopod; **H**. Second pereopod; **I**. Dactylus of third pereopod; **J**. Third pereopod; **K**. Dactylus of fifth pereopod; **L**. Fifth pereopod. Drawings were made of paratype MUMNH-CAR404-F7 (**A, B, G–L**); MUMNH-CAR404-H (**C**); MUMNH-CAR404-F1 (**D**); MUMNH-CAR402-F1 (**E**); paratype MUMNH-CAR404-M1 (**F**). Scale bars: 1 mm (**A–F**); 0.5 mm (**G, H, J, L**); 0.1 mm (**I, K**).

**Figure 26.**
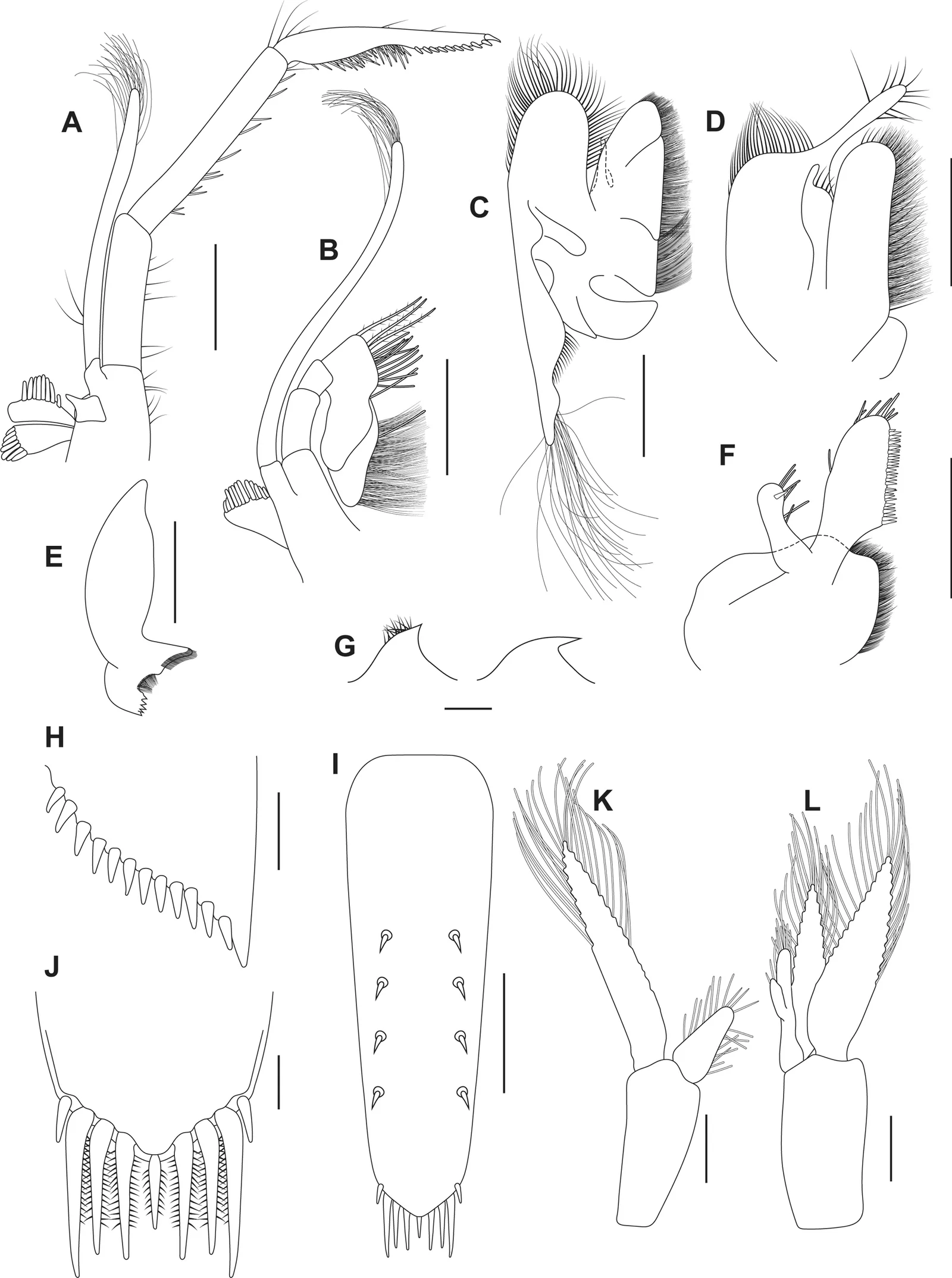
*Caridina nangroma* sp. nov. **A**. Third maxilliped; **B**. Second maxilliped; **C**. Maxilla; **D**. First maxilliped; **E**. Mandible; **F**. Maxillula; **G**. Preanal carina; **H**. Uropodal diaeresis; **I**. Telson; **J**. Distal end of telson; **K**. First pleopod; **L**. Second pleopod. Drawings were made of paratype MUMNH-CAR404-F7 (**A–I**); paratype MUMNH-CAR404-M1 (**K, L**). Scale bars: 0.5 mm (**A–F, I**); 0.25 mm (**K, L**); 0.1 mm (**G, H, J**).

Figs 5E, 23C–D, 25, 26

###### Material examined.

***Holotype*. Thailand** • ovigerous ♀, cl 2.88 mm; Prachuap Khiri Khan Province, Hua Hin District, Pranburi River; 12.4780°N, 99.7982°E; MUMNH-CAR404-H, coll. E. Jeratthitikul, W. Siriwut, K. Macharoenboon & V. Bundit, 05 April 2022. ***Paratypes***. Thailand • 9 ♀, cl 2.49–3.28 mm; same data as for holotype; MUMNH-CAR404-F1 to F7 and CAR404-P1, P3 • 8 ♂, cl 1.88–2.26 mm; same data as for holotype; MUMNH-CAR404-M1 to M8, coll. E. Jeratthitikul, W. Siriwut, K. Macharoenboon & V. Bundit, 05 April 2022.

###### Other material.

39 lots (see Suppl. material 1: Data S1).

###### Description.

***Cephalothorax and cephalic appendage*** (*n* = 7). cl 2.49–3.28 (median 2.70; Fig. 25A, C–E) mm, width 2.15–2.62 (median 2.23) mm. Rostrum straight to strongly curved downward (Fig. 25A, C–E), wider at the 1/2-length then abruptly tapering toward the tip, reaching at least beyond the end of second segment of antennular peduncle (Fig. 25B), 0.61–0.84 (median 0.75) as long as cl. Rostral formula: (2–5) 15–19/ 1–5. Antennal spine placed below inferior orbital angle. Pterygostomian margin subrectangular. Eye well developed, anterior end reaching to 0.50–0.74 (median 0.60) of first segment of antennular peduncle. Antennular peduncle 0.76–0.91 (median 0.83; Fig. 25A–E) as long as cl, first segment 1.51–1.93 (median 1.45) × as long as second segment, second segment 1.35–2.01 (median 1.88) × as long as third segment. Tooth on distolateral margin of first segment of antennular peduncle acute and well developed. Stylocerite reaching to 0.71–0.89 (median 0.79) of first segment of antennular peduncle. Scaphocerite 3.10–3.80 (median 3.44) × as long as wide, distal margin with short plumose setae.

***Branchial formula*** (*n* = 7) Podobranch on second maxilliped well developed. Third maxilliped possessed two small arthrobranchs. Pleurobranchs present on all pereopods.

***Mouthparts*** (*n* = 3). Mandible without palp (Fig. 26E); incisor process possessed five or six irregular teeth, and two rows of setae placed along inner margin; molar process truncated. Lower lacinia of maxillula sub–rectangular (Fig. 26F), with many rows of plumose setae; upper lacinia elongate, inner margin straight, with numerous conical setae and distal setae; palp elongate, with one conical seta and few distal setae. Scaphognathite of maxilla wide anteriorly and tapering posteriorly (Fig. 26C), ending in a tuft of setae and short marginal setae placed along proximal triangular process; anterior margin with long plumose setae; palp slender; upper and middle endites subdivided with many rows of marginal setae; lower endite partly fused with middle endite, with relatively longer marginal setae.

Palp of first maxilliped broadly triangular (Fig. 26D), ending in finger-like projection and with few plumose setae; caridean lobe broad, with marginal plumose setae; exopod well developed with distally marginal plumose setae, ultimate and penultimate segments of endopod indistinctly divided, with marginal setae. Ultimate and penultimate segments of endopod of second maxilliped incompletely divided (Fig. 26B); inner margin of ultimate and penultimate segments with simple and pappose setae; exopod long and slender, with a tuft of long setae at the tip. Third maxilliped with epipod (Fig. 26A), reaching to end of second segment of antennular peduncle; ultimate segment of endopod, with a row of strong setae at proximal 2/3 of posterior margin, ending in one large claw and 8–10 spiniform setae on distal 1/3 of posterior margin, 0.78–1.03 (median 0.95, *n* = 7) × as long as penultimate segment; exopod long and slender, with a tuft of long setae at the tip.

***Pereopods*** (*n* = 7). Epipod present on first two pereopods, reduced in third pereopod, and absent in last two pereopods. Chelae of P1 and P2 well developed.

P1 short (Fig. 25G); chela 2.12–3.24 (median 2.38) × as long as wide, 1.20–1.45 (median 1.34) × as long as carpus, tips of fingers rounded, with tufts of setae near tip; dactylus 1.32–1.69 (median 1.45) × as long as palm; carpus excavated distally, 2.33–2.88 (median 2.63) × as long as wide, 1.15–1.41 (median 1.21) × as long as merus; merus 1.87–2.74 (median 2.44) × as long as wide, 0.91–1.08 (median 0.98) × as long as ischium.

P2 slenderer, longer than P1 (Fig. 25H); chela 3.03–4.39 (median 3.72) × as long as wide, 0.76–0.88 (median 0.80) as long as carpus, tips of fingers rounded, with tufts of setae near tip; dactylus 1.28–1.80 (median 1.42) × as long as palm; carpus slender, 5.44–7.01 (median 6.23) × as long as wide, 1.32–1.59 (median 1.57) × as long as merus; merus 4.09–5.87 (median 4.71) × as long as wide, 0.88–1.05 (median 0.98) × as long as ischium.

P3 not sexually dimorphic (Fig. 25J); dactylus with 5–8 (mode 6) spiniform setae on flexor margin (Fig. 25I), 4.06–5.46 (median 5.21) × as long as wide (including terminal claw), terminating with one large claw; propodus bearing numerous spiniform setae on posterior margin, 9.62–14.31 (median 12.14) × as long as wide, 3.03–3.89 (median 3.24) × as long as dactylus; carpus possessed 1–3 (mode 3) setae on posterior margin of outer surface, the distal seta largest, the other setae minute, 4.61–6.10 (median 5.12) × as long as wide, 0.51–0.64 (median 0.57) as long as propodus; merus possessed three or four large setae on posterior margin of outer surface, 7.18–9.84 (median 8.61) × as long as wide, 1.78–2.18 (median 2.12) × as long as carpus; ischium with one spiniform seta.

P5 slender (Fig. 25L); dactylus with 47–55 spiniform setae on flexor margin (Fig. 25K), 4.90–6.61 (median 5.80) × as long as wide (including terminal claw), terminating with one large claw; propodus bearing numerous spiniform setae on posterior margin, 10.68–15.66 (median 12.06) × as long as wide, 2.39–2.77 (median 2.59) × as long as dactylus; carpus possessed 1–3 (mode 3) setae on posterior margin of outer surface, the distal seta largest, the other setae minute, 4.60–5.67 (median 5.15) × as long as wide, 0.44–0.51 (median 0.49) as long as propodus; merus possessed two or three (mode 2) large setae on posterior margin of outer surface, 6.40–8.16 (median 7.65) × as long as wide, 1.52–1.87 (median 1.75) × as long as carpus; ischium without spiniform seta.

***Male pleopods*** (*n* = 8). Pl1 endopod subtriangular (Fig. 26K), wider proximally, 1.70–2.88 (median 2.27) × as long as wide, 0.28–0.36 (median 0.32) of exopod length, without appendix interna. Pl2 appendix masculina rod-shaped (Fig. 26L), with many setae, 0.46–0.70 (median 0.60) as long as endopod; appendix interna very slender, reaching 0.69–0.90 (median 0.79) of appendix masculina length.

***Abdomen*** (*n* = 7). Sixth abdominal somite ~ 0.51–0.74 (median 0.64) of cl, 1.58–1.99 (median 1.70) × as long as fifth somite, 0.79–1.06 (median 0.93) × as long as telson (Fig. 25A). Telson 3.05–3.87 (median 3.28; Fig. 26I) × as long as wide, with three or four pairs of dorsal spiniform setae and one pair of dorsolateral spiniform setae; distal margin subtriangular without posteromedian projection, with six or seven moveable plumose setae, the lateral pair longest (Fig. 26J). Preanal carina subtriangular, with a prominent or small spine (Fig. 26G). Uropodal diaeresis with 11–13 moveable spiniform setae (Fig. 26H).

***Embryos***. Ovigerous females with 27–41 embryos (mean 32, n = 5). Size of embryos with eyes 0.67–0.77 × 0.37–0.49 mm (*n* = 50).

###### Coloration.

Color of body variable. Body and rostrum greyish blue, translucent gray, or camo green, usually decorated with numerous dark spots. Cephalothorax darker than the other parts with dark brown and black stripes. Embryos brown to dark brown or black (Fig. 23C–D).

###### Distribution and habitat.

Thailand. *Caridina nangroma* sp. nov. is widely distributed throughout southern Thailand (Fig. 5E). This species inhabits various habitats, such as small streams, lowland rivers, lakes, and reservoirs. Individuals are found living on aquatic vegetation along the banks.

###### Phylogenetic placement.

The molecular analyses indicated that *C. nangroma* sp. nov. is monophyletic (BS = 100%, BPP = 1.00; Fig. 4) and sister to *C. srivijaya* sp. nov. (BS = 100%, BPP = 1.00).

###### Etymology.

The specific name *nangroma* refers to the city named “Bang Nang Rom”, which was the name of ancient city located in Prachuap Khiri Khan Province, where this new species was discovered.

###### Remarks.

*Caridina nangroma* sp. nov. resembles *C. propinqua* in the morphology of the rostrum. Both species exhibit a sloped-down rostrum that reaches beyond the end of second segment of antennular peduncle (De Man 1908a; Cai et al. 2007). However, this species can be distinguished by the following characteristics, which are compared against De Man’s original description of *C. propinqua* (De Man 1908a). *Caridina nangroma* sp. nov. possesses a slightly slenderer P1 chela (2.12–3.24× as long as wide vs 2.00 in *C. propinqua*), and slenderer P2 carpus (5.44–7.01× as long as wide vs 4.5–5.5 in *C. propinqua*). Telson of *C. nangroma* sp. nov. possesses fewer plumose setae on the distal margin than that of *C. propinqua* (3–4 pairs vs 5 pairs). When compared to the description of *C. propinqua* reported in Cai and Shokita (2006a) and Cai et al. (2007), *C. nangroma* sp. nov. has a shorter antennular peduncle (0.76–0.91 as long as cl vs subequal to cl), more dorsal teeth on rostrum (15–19 vs 9–16), more spiniform setae on P3 dactylus (5–8 vs 2–5), fewer spiniform setae on P5 dactylus (47–55 vs 57–76), a lack of posteromedian projection (vs with a small posteromedian projection), shorter sixth abdominal somite (0.51–0.74× as long as cl vs 0.8), and much larger embryos (0.68–0.75 × 0.37–0.49 mm vs 0.32–0.48 × 0.20–0.30 mm).

*Caridina nangroma* sp. nov. can be distinguished from *C. johnsoni* primarily by the morphology of its rostrum. While the rostrum of *C. johnsoni* is notably narrow, relatively straight, and maintains a subequal width throughout its length; the rostrum of *C. nangroma* sp. nov. is conspicuously wider at its middle, before tapering distinctly towards the tip. The P5 dactylus of the new species also possesses fewer spiniform setae (47–55 vs 50–62).

*Caridina nangroma* sp. nov. is also similar to *C. rangoona*. However, this new species differs from *C. cf. rangoona* by the shape of rostrum (strongly curved downward and slightly upcurved toward the tip vs straight or slightly curved downward anteriorly and not upturned at the tip), having fewer spiniform setae on uropodal diaeresis (11–13 vs 16–21), and larger embryos (0.67–0.77 × 0.37–0.49 mm vs 0.37–0.46 × 0.24–0.31 mm).

The new species can be distinguished from *C. kottelati* by its rostral shape (sloped down, wider at the middle and tapering toward the tip vs straight and subequal in width throughout the rostrum length), a longer antennular peduncle (0.76–0.91× as long as cl vs 0.67), a smaller number of spiniform setae on P5 dactylus (47–55 vs 62), an armed preanal carina (vs without a spine), and significantly smaller embryos (0.67–0.77 × 0.37–0.49 mm vs 0.95 × 0.60 mm) (Cai and Naiyanetr 2024).

Kemp (1918b) reported the presence of *C. propinqua* in southern Thailand among specimens collected by Dr. Annandale from Songkhla Lake and the Pattani River. However, according to the given description, those specimens shared several similar characters with *C. nangroma* sp. nov., including the rostral formula: (2–5) 15–19/ 1–5 vs (2–4) 11–20/ 0–4, spiniform setae on P3 dactylus (5–8 vs 6–7), spiniform setae on P5 dactylus (47–55 vs 43–55), embryo size (0.68–0.75 × 0.37–0.49 mm vs 0.67–0.77 × 0.37–0.49 mm). Therefore, we suggest that the specimens in Kemp (1918b) are likely to be *C. nangroma* sp. nov. instead of *C. propinqua*. A thorough re-examination of this collection is necessary to confirm this conclusion.

##### Caridina srivijaya sp. nov.

Taxon classificationAnimaliaDecapodaAtyidae

8C398F1A-0A36-5176-A4E3-884C30ED65BB

https://zoobank.org/C368C99E-0965-4B9E-82BB-7111C3059B80

**Figure 27.**
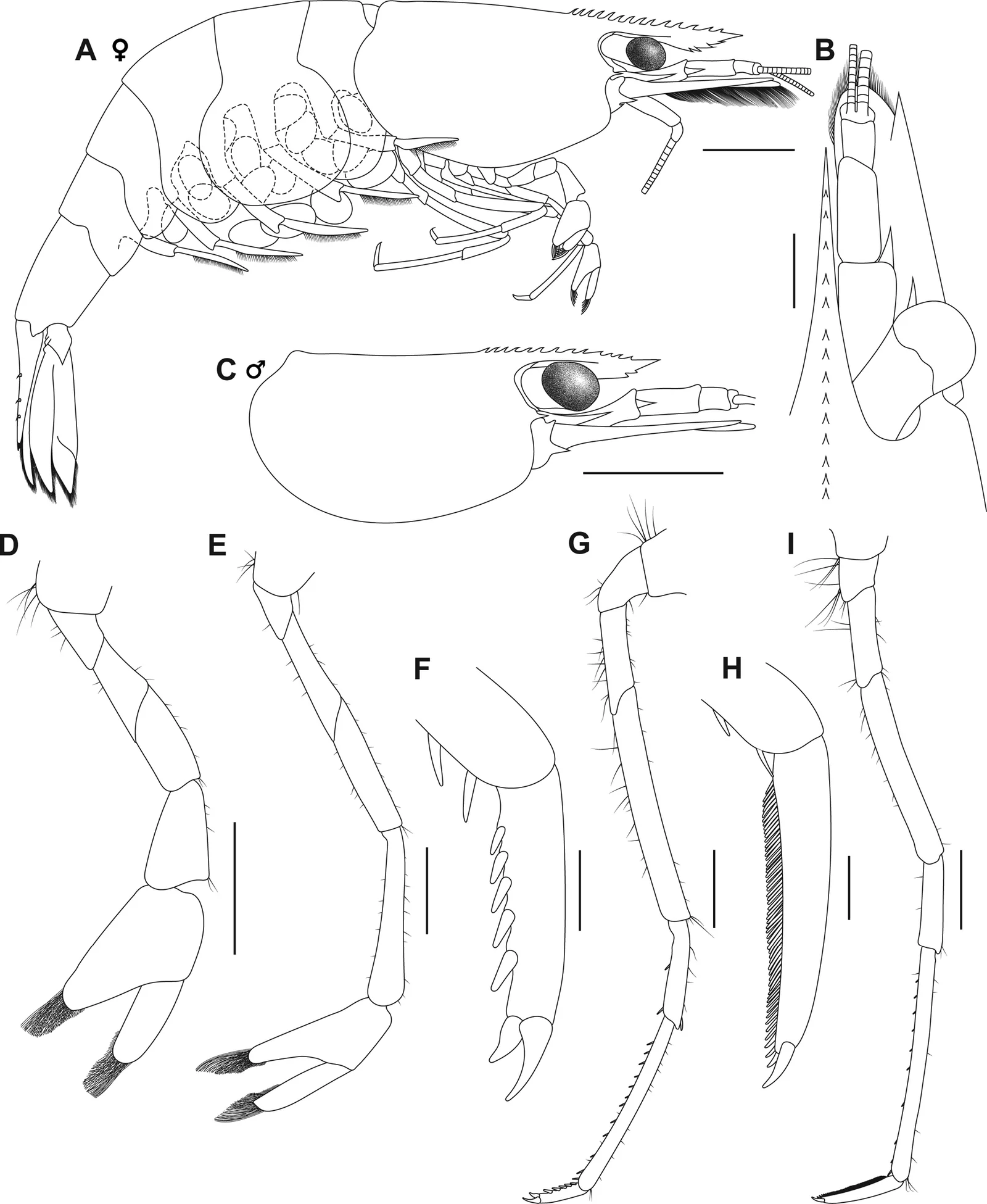
*Caridina srivijaya* sp. nov. **A**. Ovigerous female; **B**. Top view of carapace; **C**. Cephalothorax and cephalic appendages of male; **D**. First pereopod; **E**. Second pereopod; **F**. Dactylus of third pereopod; **G**. Third pereopod; **H**. Dactylus of fifth pereopod; **I**. Fifth pereopod. Drawings were made of paratype MUMNH-CAR502-F2 (**A, B, D–I**); paratype MUMNH-CAR502-M1 (**K–L**). Scale bars: 1 mm (**A, B**); 0.5 mm (**C–E, G, I**); 0.1 mm (**F, H**).

**Figure 28.**
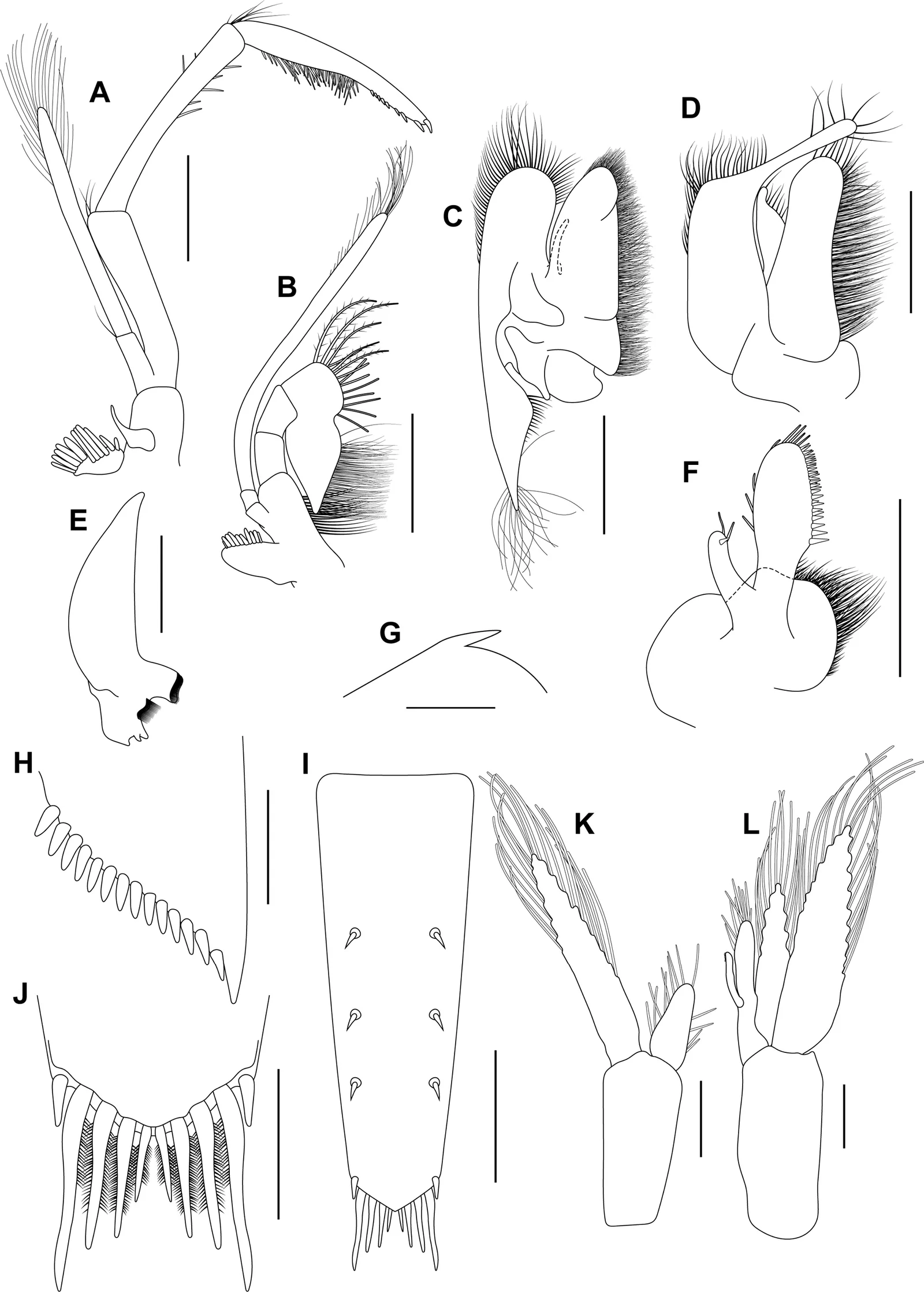
*Caridina srivijaya* sp. nov. **A**. Third maxilliped; **B**. Second maxilliped; **C**. Maxilla; **D**. First maxilliped; **E**. Mandible; **F**. Maxillula; **G**. Preanal carina; **H**. Uropodal diaeresis; **I**. Telson; **J**. Distal end of telson; **K**. First pleopod; **L**. Second pleopod. Drawings were made of paratype MUMNH-CAR502-F8 (**A–I**); paratype MUMNH-CAR502-M1 (**K–L**). Scale bars: 0.5 mm (**A–F, I**); 0.25 mm (**J, K, L**); 0.1 mm (**G, H**).

Figs 5E, 23E, 23F, 27, 28

###### Material examined.

***Holotype*. Thailand** • ovigerous ♀, cl 2.59 mm; Surat Thani Province, Phrasaeng District, Bang Sawan Spring; 8.6730°N, 98.9798°E; MUMNH-CAR502-H, coll. K. Macharoenboon, E. Jeratthitikul, W. Siriwut, P. Yongsiri & P. Sapparojpattana, 18 December 2022. ***Paratypes*. Thailand** • 8 ovigerous ♀, cl 2.38–2.73 mm; same data as for holotype; MUMNH-CAR502-F1 to F6, F8, and CAR502-P1 • 9 ♂, cl 1.66–2.15 mm; MUMNH-CAR502-M1 to M9, coll. K. Macharoenboon, E. Jeratthitikul, W. Siriwut, P. Yongsiri & P. Sapparojpattana, 18 December 2022.

###### Other material.

One lot (see Suppl. material 1: Data S1).

###### Description.

***Cephalothorax and cephalic appendage*** (*n* = 7). cl 2.38–2.83 (median 2.67; Fig. 27A) mm, width 1.72–2.14 (median 1.90) mm. Rostrum minute (Fig. 27A), slightly pointed down, reaching at least the end of second segment of antennular peduncle, but not longer than the end of third segment of antennular peduncle (Fig. 27B), 0.58–0.72 (median 0.64) as long as cl. Rostral formula: (4–5) 14–19/ 2–6. Antennal spine placed below inferior orbital angle, Pterygostomian margin round. Eye well developed, anterior end reaching to 0.61–0.78 (median 0.71) of first segment of antennular peduncle. Antennular peduncle 0.65–0.81 (median 0.71; Fig. 27A, B) as long as cl, first segment 1.60–1.98 (median 1.73) × as long as second segment, second segment 1.28–2.07 (median 1.59) × as long as third segment. Tooth on distolateral margin of first segment of antennular peduncle acute and well developed. Stylocerite reaching to 0.74–0.86 (median 0.79) of first segment of antennular peduncle. Scaphocerite 2.93–3.34 (median 3.17) × as long as wide, distal margin with short plumose setae.

***Branchial formula*** (*n* = 7). Podobranch on second maxilliped well developed. Third maxilliped possessed two small arthrobranchs. Pleurobranchs present on all pereopods.

***Mouthparts*** (*n* = 3). Mandible without palp (Fig. 28E); incisor process possessed three or four irregular teeth, and two rows of setae placed along inner margin; molar process truncated. Lower lacinia of maxillula sub–rectangular (Fig. 28F), with many rows of plumose setae; upper lacinia elongate, inner margin straight, with numerous conical setae and distal setae; palp elongate, with one conical seta and few distal setae. Scaphognathite of maxilla wide anteriorly and tapering posteriorly (Fig. 28C), ending in a tuft of setae and short marginal setae placed along proximal triangular process; anterior margin with long plumose setae; palp slender; upper and middle endites subdivided with many rows of marginal setae; lower endite partly fused with middle endite, with relatively longer marginal setae.

Palp of first maxilliped subtriangular (Fig. 28D), ending in finger-like projection and with few plumose setae; caridean lobe broad, with marginal plumose setae; exopod well developed with distally marginal plumose setae; ultimate and penultimate segments of endopod indistinctly divided, with marginal setae. Ultimate and penultimate segments of endopod of second maxilliped incompletely divided (Fig. 28B); inner margin of ultimate and penultimate segments with simple and pappose setae; exopod long and slender, with a tuft of long setae at the tip. Third maxilliped with epipod (Fig. 28A), reaching to end of second segment of antennular peduncle; ultimate segment of endopod, with a row of strong setae at proximal 2/3 of posterior margin, ending in one large claw and 6–9 spiniform setae on distal 1/3 of posterior margin, 0.91–1.09 (median 0.98, *n* = 7) × as long as penultimate segment; exopod long and slender, with a tuft of long setae at the tip.

***Pereopods*** (*n* = 7). Epipod well developed first two pereopods, reduced in third pereopod, and absent in last two pereopods. Chelae of P1 and P2 well developed.

P1 short (Fig. 27D); chela 1.68–2.01 (median 1.93) × as long as wide, 1.16–1.46 (median 1.42) × as long as carpus, tips of fingers rounded, with tuft of setae near tip; dactylus 1.05–1.49 (median 1.23) × as long as palm; carpus stout, excavated distally, 1.71–2.24 (median 1.91) × as long as wide, 1.04–1.54 (median 1.32) × as long as merus; merus 1.76–2.37 (median 2.08) × as long as wide, 0.96–1.09 (median 1.01) × as long as ischium.

P2 slenderer, longer than P1 (Fig. 27E); chela 2.41–3.19 (median 2.96) × as long as wide, 0.79–0.99 (median 0.83) as long as carpus, tips of fingers rounded, with tuft of setae near tip; dactylus 1.31–1.78 (median 1.46) × as long as palm; carpus slender, 4.78–5.61 (median 5.16) × as long as wide, 1.20–1.59 (median 1.47) × as long as merus; merus 3.82–4.92 (median 4.25) × as long as wide, 0.95–1.12 (median 1.04) × as long as ischium.

P3 not sexually dimorphic (Fig. 27G); dactylus with six or seven (mode 6) spiniform setae on flexor margin (Fig. 27F), 3.74–4.88 (median 4.33) × as long as wide (including terminal claw), terminating with one large claw; propodus bearing numerous spiniform setae on posterior margin, 11.31–15.51 (median 11.77) × as long as wide, 3.09–3.95 (median 3.48) × as long as dactylus; carpus possessed three setae on posterior margin of outer surface, the distal seta largest, the other setae minute, 3.74–6.27 (median 5.30) × as long as wide, 0.51–0.54 (median 0.53) as long as propodus; merus possessed four large setae on posterior margin of outer surface, 7.32–9.15 (median 8.42) × as long as wide, 1.77–2.40 (median 2.20) × as long as carpus; ischium with one spiniform seta.

P5 slender (Fig. 27I); dactylus with 41–55 spiniform setae on flexor margin (Fig. 27H), 4.10–6.67 (median 5.07) × as long as wide (including terminal claw), terminating with one large claw; propodus bearing numerous spiniform setae on posterior margin, 12.39–17.12 (median 15.40) × as long as wide, 2.40–3.08 (median 2.78) × as long as dactylus; carpus possessed two or three setae on posterior margin of outer surface, the distal seta largest, the other setae minute, 4.33–5.47 (median 4.54) × as long as wide, 0.40–0.54 (median 0.45) as long as propodus; merus possessed three large setae on posterior margin of outer surface, 5.32–8.00 (median 7.11) × as long as wide, 1.21–1.98 (median 1.79) × as long as carpus; ischium without spiniform seta.

***Male pleopods*** (*n* = 9). Pl1 endopod subtriangular (Fig. 28K), wider proximally, 2.20–2.81 (median 2.53) × as long as wide, 0.33–0.39 (median 0.35) of exopod length, without appendix interna. Pl2 appendix masculina rod-shaped (Fig. 28L), with many setae, 0.70–0.85 (median 0.80) as long as endopod; appendix interna very slender, reaching 0.72–0.87 (median 0.80) of appendix masculina length.

***Abdomen*** (*n* = 7). Sixth abdominal somite ~ 0.48–0.57 (median 0.51) of cl, 1.65–2.00 (median 1.71) × as long as fifth somite, 0.71–0.95 (median 0.81) as long as telson (Fig. 27A). Telson 2.85–3.46 (median 3.25; Fig. 28I) × as long as wide, with three or four pairs of dorsal spiniform setae and one pair of dorsolateral spiniform setae; distal margin subtriangular, without posteromedian projection, with 6–8 moveable plumose setae, the lateral pair longest, the intermediate pair shortest (Fig. 28J). Preanal carina low with a spine (Fig. 28G). Uropodal diaeresis with 13–16 moveable spiniform setae (Fig. 28H).

***Embryos***. Ovigerous females with 13–29 embryos (mean 21, *n* = 5). Size of embryos with eyes 0.80–0.88 × 0.47–0.58 mm (*n* = 50).

###### Coloration.

Body translucent, with numerous deep-sea blue spots. Rostrum translucent, with a few dark spots. Cephalic region darker than the other parts, decorated with black stripes. Embryos muddy green to dark brown (Fig. 23E, F).

###### Distribution and habitat.

Thailand. The distribution of *C. srivijaya* sp. nov. is restricted to the type locality (Fig. 5E). The shrimps were collected from an isolated freshwater spring located far from the coastal area. They were found to dwell densely within the root system of riparian plants and the stems and leaves of submerged plants.

###### Phylogenetic placement.

The molecular analyses indicated that *C. srivijaya* sp. nov. is monophyletic (BS = 100%, BPP = 1.00; Fig. 4) and sister to *C. nangroma* sp. nov. (BS = 100%, BPP = 1.00).

###### Etymology.

The specific name refers to *Srivijaya*—the ancient empire existing from the 7^th^ to the 11^th^ century influencing the Malay Peninsula at some stage.

###### Remarks.

This species can be separated from its relatives (i.e., other southern Thai species in the *C. laevis* species group) by characters of the rostrum and P1 chelae. The rostrum of *C. srivijaya* sp. nov. is minute, straight, and slightly pointed down, and its P1 chelae are much stouter compared to its relatives.

*Caridina srivijaya* sp. nov. is similar to *C. nangroma* sp. nov. in terms of morphology and genetics. They were recovered as a monophyletic with high support in our phylogenetic analyses (BS = 100%, BPP = 1; Fig. 4). Both species share characteristics of their rostral formulae and number of spiniform setae on each appendage. However, *C. srivijaya* sp. nov. can be separated from *C. nangroma* sp. nov. by possessing a shorter and slenderer rostrum that is not wider at the middle of rostrum, stouter P1 chelae (1.68–2.01× as long as wide vs 2.12–3.24), stouter P1 carpus (1.71–2.24× as long as wide vs 2.33–2.88), stouter P2 chela (2.41–3.19× as long as wide vs 3.03–4.39), stouter P2 carpus (4.78–5.61× as long as wide vs 5.44–7.01), and bearing slightly larger embryos (0.80–0.88 × 0.47–0.58 mm vs 0.68–0.75 × 0.37–0.49 mm).

This new species also resembles *C. propinqua*, but differs by having shorter antennular peduncle (0.65–0.81× as long as cl vs subequal to carapace length; Cai et al. 2007), higher number of spiniform setae on P3 dactylus (6–7 vs 2–5; De Man 1908a; Cai et al. 2007), shorter sixth abdominal somite (0.48–0.57× as long as cl vs 0.8; Cai et al. 2007), and much larger embryos (0.80–0.88 × 0.47–0.58 vs 0.32–0.36 × 0.20–0.22; Cai et al. 2007).

##### Caridina takkola sp. nov.

Taxon classificationAnimaliaDecapodaAtyidae

BBBF9DD4-88ED-59CD-9D8F-17220D3E62E0

https://zoobank.org/D8FB6E97-DB0D-441F-82C2-19AA52A759E2

**Figure 29.**
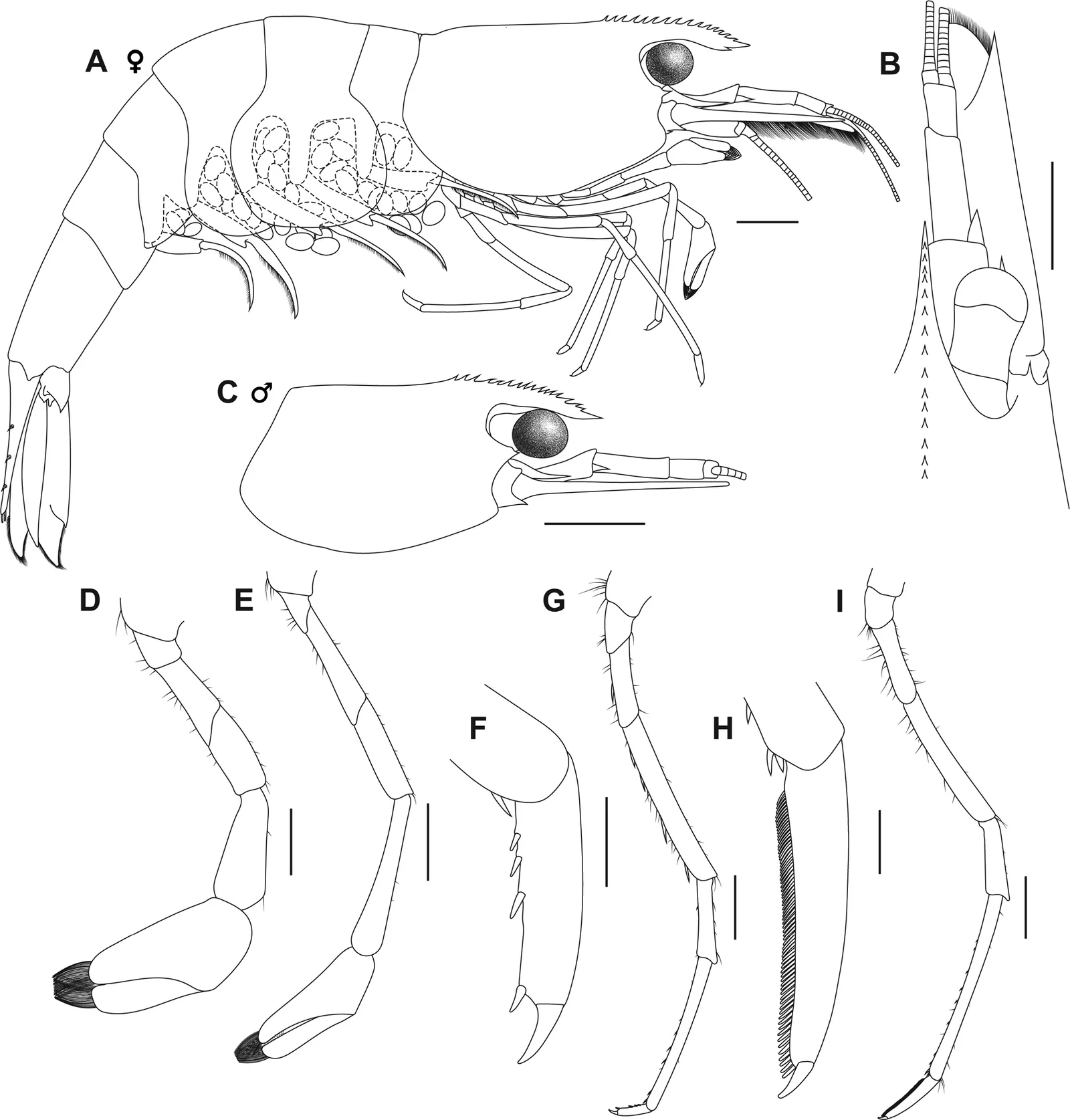
*Caridina takkola* sp. nov. **A**. Ovigerous female; **B**. Top view of carapace; **C**. Cephalothorax and cephalic appendages of female; **D**. First pereopod; **E**. Second pereopod; **F**. Dactylus of third pereopod; **G**. Third pereopod; **H**. Dactylus of fifth pereopod; **I**. Fifth pereopod. Drawings were made of paratype MUMNH-CAR469-F8 (**A, B, D–I**); paratype MUMNH-CAR469-M1 (**C**). Scale bars: 1 mm (**A–C**); 0.5 mm (**D, E, G, I**); 0.1 mm (**F, H**).

**Figure 30.**
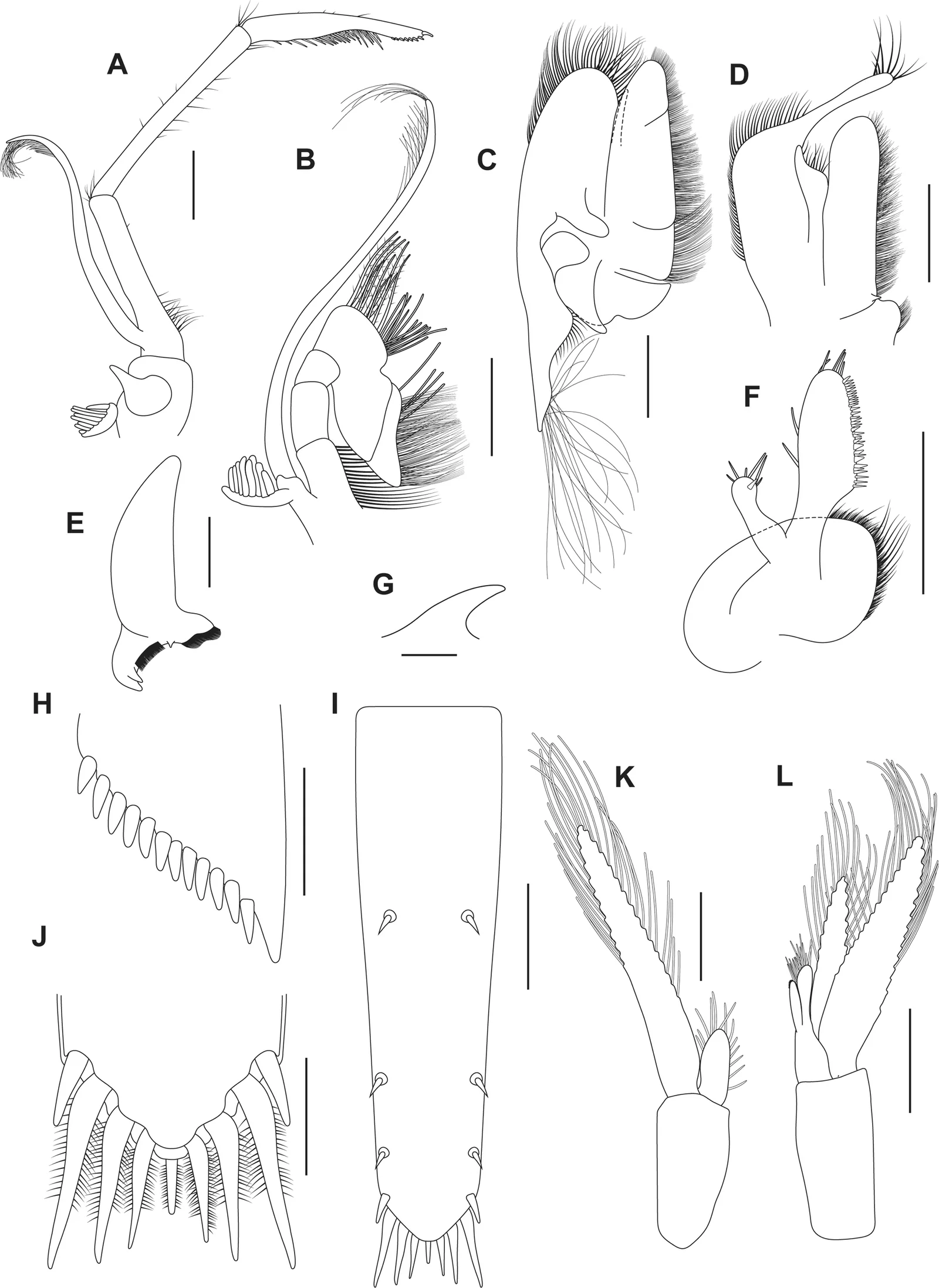
*Caridina takkola* sp. nov. **A**. Third maxilliped; **B**. Second maxilliped; **C**. Maxilla; **D**. First maxilliped; **E**. Mandible; **F**. Maxillula; **G**. Preanal carina; **H**. Uropodal diaeresis; **I**. Telson; **J**. Distal end of telson; **K**. First pleopod; **L**. Second pleopod. Drawings were made of paratype MUMNH-CAR469-F1 (**A–I**); MUMNH-CAR469-M1 (**K–L**). Scale bars: 0.5 mm (**A–F, I, K, L**); 0.25 mm (**H, J**); 0.1 mm (**G**).

Figs 5F, 23G, 29, 30

###### Material examined.

***Holotype*. Thailand** • ovigerous ♀, cl 3.16 mm; Surat Thani Province, Mueang District, Phunphin Stream; 9.1936°N, 99.2816°E; MUMNH-CAR469-H, coll. K. Macharoenboon, E. Jeratthitikul, W. Siriwut, P. Yongsiri & P. Sapparojpattana, 17 December 2022. ***Paratypes*. Thailand** • 8 ovigerous ♀, cl 3.07–3.80 mm; same data as for holotype; MUMNH-CAR469-F1 to F7 and CAR469-P1 • 8 ♂, cl 1.89–2.49 mm; same data as for holotype; MUMNH-CAR469-M1 to M10, coll. K. Macharoenboon, E. Jeratthitikul, W. Siriwut, P. Yongsiri & P. Sapparojpattana, 17 December 2022.

###### Other material.

Three lots (see Suppl. material 1: Data S1).

###### Description.

***Cephalothorax and cephalic appendage*** (*n* = 7). cl 3.07–3.80 (median 3.60; Fig. 29A) mm, width 2.41–2.98 (median 2.79) mm. Rostrum very short, convex (Fig. 29A), reaching near or slightly beyond the end of first segment of antennular peduncle (Fig. 29B), 0.44–0.52 (median 0.50) as long as cl. Rostral formula: (3–4) 13–18/ 1–2. Antennal spine placed below inferior orbital angle, fused with, or slightly separated from orbital margin. Pterygostomian margin round. Eye well developed, anterior end reaching to 0.45–0.68 (median 0.57) of first segment of antennular peduncle. Antennular peduncle 0.80–0.92 (median 0.84; Fig. 29A, B) as long as cl, first segment 1.47–1.97 (median 1.59) × as long as second segment, second segment 1.40–2.18 (median 1.66) × as long as third segment. Tooth on distolateral margin of first segment of antennular peduncle acute and well developed. Stylocerite reaching to 0.77–0.89 (median 0.83) of first segment of antennular peduncle. Scaphocerite 3.28–4.03 (median 3.54) × as long as wide, distal margin with short plumose setae.

***Branchial formula*** (*n* = 7). Podobranch on second maxilliped well developed. Third maxilliped possessed two small arthrobranchs. Pleurobranchs present on all pereopods.

***Mouthparts*** (*n* = 3). Mandible without palp (Fig. 30E); incisor process possessed 2–4 irregular teeth, and two rows of setae placed along inner margin; molar process truncated. Lower lacinia of maxillula sub–rectangular (Fig. 30F), with many rows of plumose setae; upper lacinia elongate, inner margin straight, with numerous conical setae and distal setae; palp elongate, with one conical seta and few distal setae. Scaphognathite of maxilla wide anteriorly and tapering posteriorly (Fig. 30C), ending in a tuft of setae and short marginal setae placed along proximal triangular process; anterior margin with long plumose setae; palp slender; upper and middle endites subdivided with many rows of marginal setae; lower endite partly fused with middle endite, with relatively longer marginal setae.

Palp of first maxilliped subtriangular (Fig. 30D), ending in triangular projection and with few plumose setae; caridean lobe broad, with marginal plumose setae; exopod well developed with distally marginal plumose setae; ultimate and penultimate segments of endopod indistinctly divided, with many marginal setae. Ultimate and penultimate segments of endopod of second maxilliped incompletely divided (Fig. 30B); inner margin of ultimate and penultimate segments with simple and pappose setae; exopod long and slender, with a tuft of long setae at the tip. Third maxilliped with epipod (Fig. 30A), reaching to end of second segment of antennular peduncle; ultimate segment of endopod, with a row of strong setae at proximal 2/3 of posterior margin, ending in one large claw and nine or ten spiniform setae on distal 1/3 of posterior margin, 0.74–0.80 (median 0.79, *n* = 7) as long as penultimate segment; exopod long and slender, with a tuft of long setae at the tip.

***Pereopods*** (*n* = 7). Epipod well developed on first two pereopods, reduced in third pereopod, and absent in last two pereopods. Chelae of P1 and P2 well developed.

P1 short (Fig. 29D); chela 1.91–2.28 (median 2.18) × as long as wide, 1.26–1.38 (median 1.32) × as long as carpus, tips of fingers rounded, with tuft of setae near tip; dactylus 1.20–179 (median 1.56) × as long as palm; carpus excavated distally, 2.27–2.70 (median 2.52) × as long as wide, 1.16–1.42 (median 1.39) × as long as merus; merus 2.29–2.70 (median 2.45) × as long as wide, 0.95–1.07 (median 0.99) × as long as ischium.

P2 slenderer, longer than P1 (Fig. 29E); chela 2.48–2.93 (median 2.88) × as long as wide, 0.75–0.88 (median 0.82) as long as carpus, tips of fingers rounded, with tuft of setae near tip; dactylus 1.38–1.79 (median 1.60) × as long as palm; carpus slender, 5.20–6.10 (median 5.43) × as long as wide, 1.39–1.67 (median 1.60) × as long as merus; merus 3.72–4.73 (median 4.26) × as long as wide, 0.84–1.07 (median 0.93) × as long as ischium.

P3 not sexually dimorphic (Fig. 29G); dactylus with 4–6 (mode 4) spiniform setae on flexor margin (Fig. 29F), 3.38–4.25 (median 3.87) × as long as wide (including terminal claw), terminating with one large claw; propodus bearing numerous spiniform setae on posterior margin, 11.76–14.20 (median 13.54) × as long as wide, 3.39–4.90 (median 4.21) × as long as dactylus; carpus possessed 2–4 setae on posterior margin of outer surface, the distal seta largest, the other setae minute, 4.73–6.02 (median 5.71) × as long as wide, 0.50–0.57 (median 0.52) as long as propodus; merus possessed 3–5 (mode 4) large setae on posterior margin of outer surface, 8.16–9.38 (median 8.65) × as long as wide, 1.87–2.22 (median 2.10) × as long as carpus; ischium with one spiniform seta.

P5 slender (Fig. 29I); dactylus with 59–67 spiniform setae on flexor margin (Fig. 29H), 4.35–6.34 (median 5.34) × as long as wide (including terminal claw), terminating with one large claw; propodus bearing numerous spiniform setae on posterior margin, 14.36–15.86 (median 15.11) × as long as wide, 2.58–3.13 (median 2.75) × as long as dactylus; carpus possessed 1–3 setae on posterior margin of outer surface, the distal seta largest, the other setae minute, 4.30–6.46 (median 5.49) × as long as wide, 0.41–0.51 (median 0.47) as long as propodus; merus possessed two or three (mode 2) large setae on posterior margin of outer surface, 7.36–9.62 (median 8.56) × as long as wide, 1.71–1.84 (median 1.76) × as long as carpus; ischium without spiniform seta.

***Male pleopods*** (*n* = 8). Pl1 endopod subtriangular (Fig. 30K), wider proximally, 2.34–3.39 (median 2.85) × as long as wide, 0.25–0.31 (median 0.29) of exopod length, without appendix interna. Pl2 appendix masculina rod-shaped (Fig. 30L) with many setae, 0.47–0.65 (median 0.54) as long as endopod; appendix interna very slender, reaching 0.80–0.99 (median 0.87) of appendix masculina length.

***Abdomen*** (*n* = 7). Sixth abdominal somite, 0.66–0.69 (median 0.67) of cl, 1.46–1.77 (median 1.67) × as long as fifth somite, 0.82–0.97 (median 0.91) as long as telson (Fig. 29A). Telson 3.52–3.83 (median 3.73; Fig. 30I) × as long as wide, with three pairs of dorsal spiniform setae and one pair of dorsolateral spiniform setae; distal margin subtriangular without posteromedian projection, six or seven moveable plumose setae, the lateral pair longest (Fig. 30J). Preanal carina high, fin-shaped, without a spine (Fig. 30G). Uropodal diaeresis with 12–15 moveable spiniform setae (Fig. 30H).

***Embryos***. Ovigerous females with 85–149 embryos (mean 118, *n* = 7). Size of embryos with eyes 0.57–0.66 × 0.35–0.42 mm (*n* = 50).

###### Coloration.

Body translucent, with brown stripes and a few dark spots. Rostrum translucent. Cephalic region darker than the other parts. Embryos brown (Fig. 23G).

###### Distribution and habitat.

Thailand. This species was collected from freshwater and brackish water near the coastal area of the Gulf of Thailand (Fig. 5F). It inhabits lake and lowland rivers. Individuals are found living on aquatic vegetation along the banks.

###### Phylogenetic placement.

The molecular analyses indicated that *C. takkola* sp. nov. is monophyletic (BS = 100%, BPP = 1.00; Fig. 4), and sister to *C. johnsoni* (BS = 100%, BPP = 1.00).

###### Etymology.

The proposed specific name “Takkola” or “Takōla” refers to the name of a mysterious ancient port city believed to exist in southern Thailand. This major maritime port appears in at least two historical documents, which are “Geography of Claudius Ptolemy” and “Milinda Pañha” [= The Questions of Milinda]. It has been suggested that this city was founded approximately 2,000 years ago, located on the western coast of the Malay Peninsula.

###### Remarks.

*Caridina takkola* sp. nov. can be distinguished from other species from southern Thailand that belong to the *C. laevis* species group by the shape of rostrum. The rostrum of *C. takkola* sp. nov. is remarkable in being convex and very short, reaching near the end of first segment of antennular peduncle, 0.44–0.52× as long as cl.

Based on the current phylogenetic tree, *C. takkola* sp. nov. is sister to *C. johnsoni* with maximum support (BS = 100%, BPP = 1; Fig. 4). In addition, they share several key characteristics such as the proportion of segment on P1–P5, the same number of spiniform setae on the P3 dactylus (4–6) and a comparable number on the P5 dactylus (59–67 vs 50–62), slender Pl1 exopod (endopod 0.25–0.31× as long as exopod vs 0.23–0.27), the number of spiniform setae on uropodal diaeresis (12–15 vs 11–15), the feature of plumose setae on distal end of telson (lateral setae longer than median setae). However, *C. takkola* sp. nov. differed distinctly from *C. johnsoni* in having much shorter (0.44–0.52× as long as cl vs 0.62–0.75) and more convex rostrum (vs slightly curved downward rostrum), a fin-shaped preanal carina (vs low preanal carina), and its slightly smaller embryo size (0.57–0.66 × 0.35–0.42 mm vs 0.62–0.73 × 0.38–0.45 mm).

##### Caridina tambralinga sp. nov.

Taxon classificationAnimaliaDecapodaAtyidae

97526334-4E87-57ED-96A3-C66A1EFE2AC1

https://zoobank.org/F2547BC4-4E85-40FD-8704-C259F36867DA

**Figure 31.**
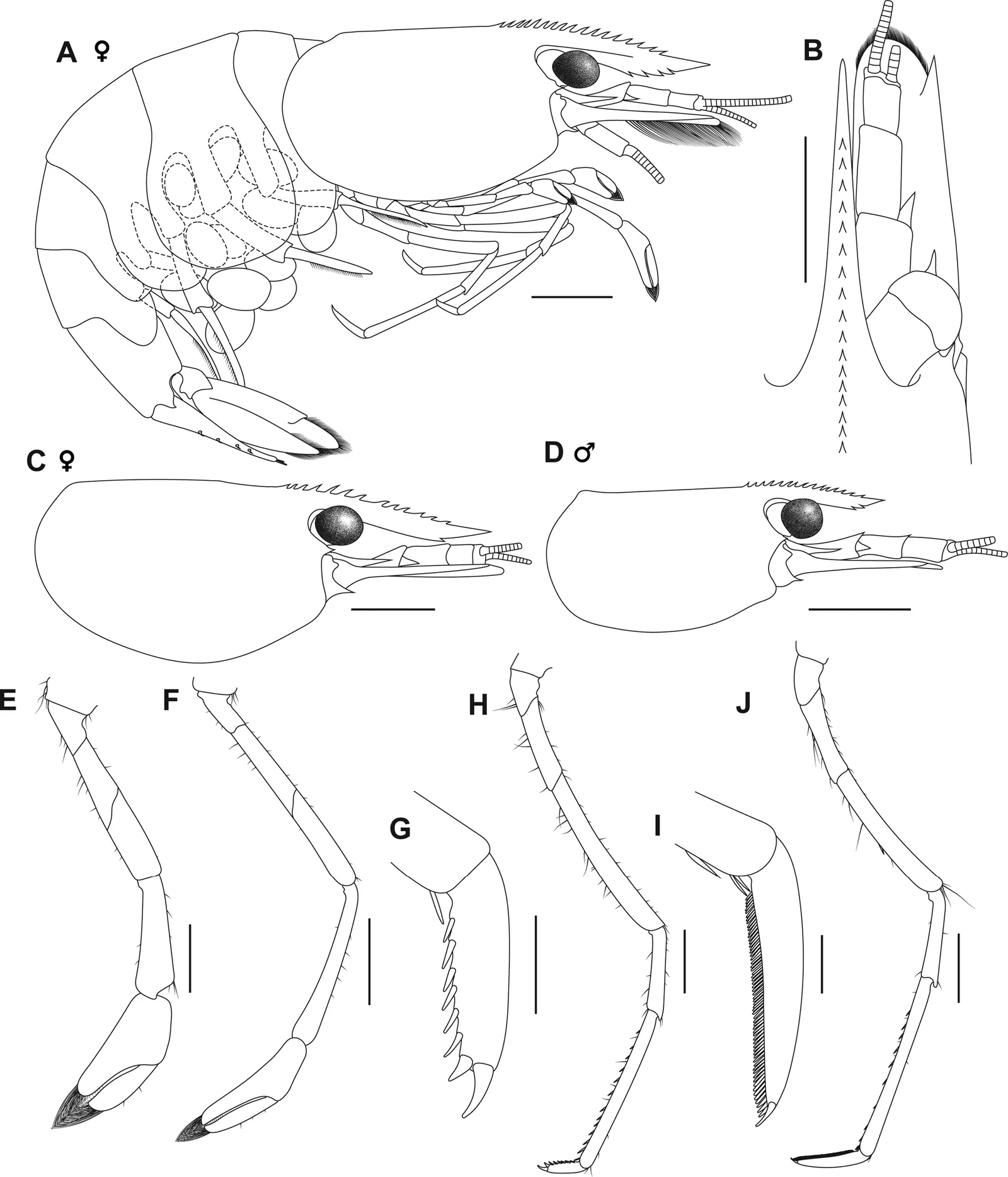
*Caridina tambralinga* sp. nov. **A**. Ovigerous female; **B**. Top view of carapace; **C**. Cephalothorax and cephalic appendages of female; **D**. Cephalothorax and cephalic appendages of male; **E**. First pereopod; **F**. Second pereopod; **G**. Dactylus of third pereopod; **H**. Third pereopod; **I**. Dactylus of fifth pereopod; **J**. Fifth pereopod. Drawings were made of paratype MUMNH-CAR461-F2 (**A, B, E–J**); MUMNH-CAR461-H (**C**); paratype MUMNH-CAR461-M1 (**D**). Scale bars: 1 mm (**A–D**); 0.5 mm (**E, F, H, J**); 0.1 mm (**G, I**).

**Figure 32.**
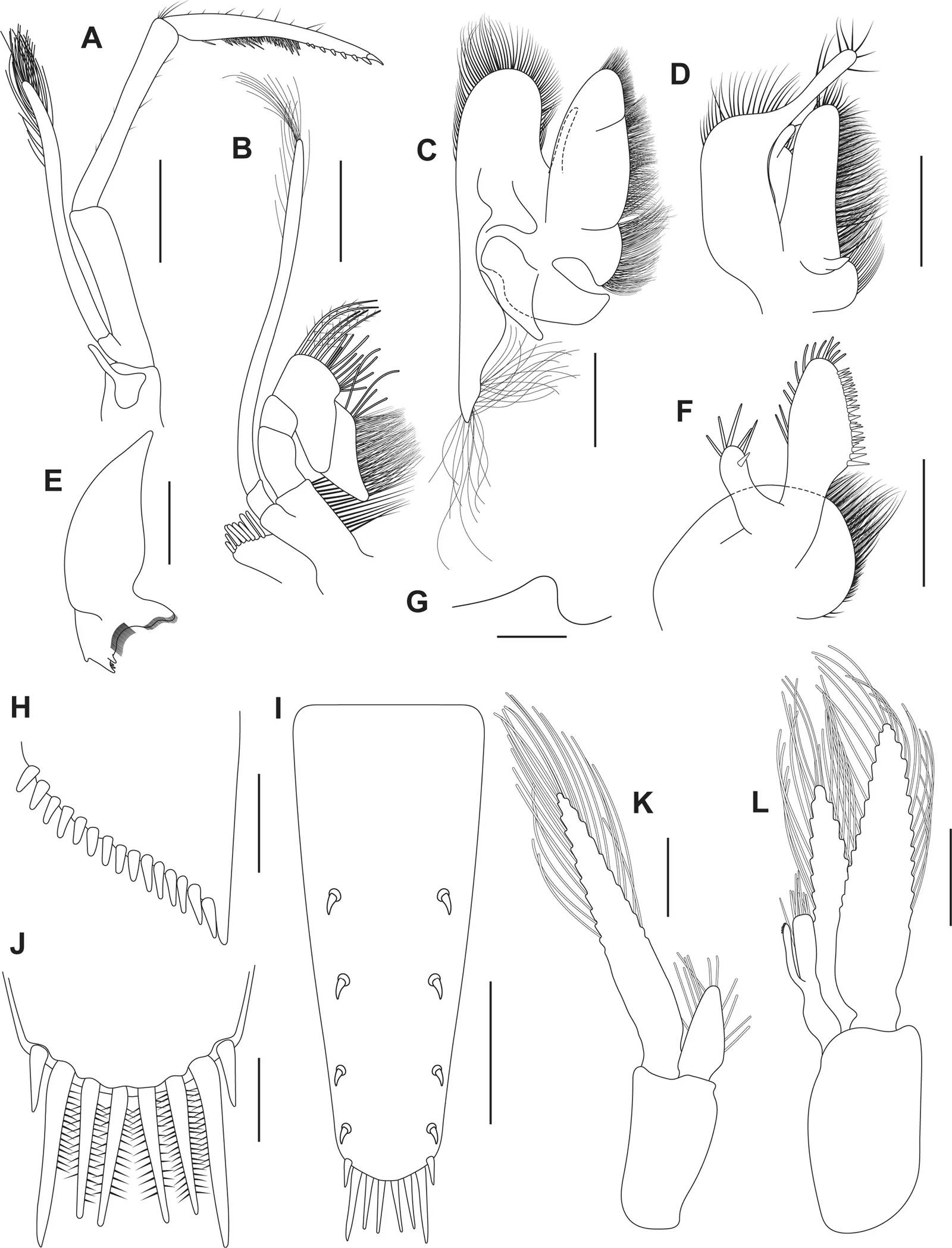
*Caridina tambralinga* sp. nov. **A**. Third maxilliped; **B**. Second maxilliped; **C**. Maxilla; **D**. First maxilliped; **E**. Mandible; **F**. Maxillula; **G**. Preanal carina; **H**. Uropodal diaeresis; **I**. Telson; **J**. Distal end of telson; **K**. First pleopod; **L**. Second pleopod. Drawings were made of paratype MUMNH-CAR461-F7 (**A–I**); paratype MUMNH-CAR461-M1 (**K–L**). Scale bars: 0.5 mm (**A–F, I**); 0.25 mm (**K, L**); 0.1 mm (**G, H, J**).

Figs 5F, 23H, 31, 32

###### Material examined.

***Holotype*. Thailand** • ovigerous ♀, cl 2.90 mm; Nakhon Si Thammarat Province, Sichon District, Thathon Stream; 8.8876°N, 99.8997°E; MUMNH-CAR461-H, coll. K. Macharoenboon, E. Jeratthitikul & N. Wutthituntisil, 17 June 2022. ***Paratypes*. Thailand** • 6 ♀, cl 2.47–2.91 mm; same data as for holotype; MUMNH-CAR461-F2 to F7 • 6 ♂, cl 1.61–1.95 mm; same data as for holotype; MUMNH-CAR461-M1 to M6, coll. K. Macharoenboon, E. Jeratthitikul & N. Wutthituntisil, 17 June 2022.

###### Other material.

15 lots (see Suppl. material 1: Data S1).

###### Description.

***Cephalothorax and cephalic appendage*** (*n* = 6). cl 2.47–2.91 (median 2.73; Fig. 31A, C) mm, width 2.13–2.43 (median 2.17) mm. Rostrum curved down (Fig. 31A, C), slightly recurved near the tip, reaching at least the end of second segment of antennular peduncle (Fig. 31B), usually longer than the end of third segment of antennular peduncle, 0.70–0.88 (median 0.78) as long as cl. Rostral formula: (3–5) 15–20/ 2–4. Antennal spine placed below inferior orbital angle, fused with, or slightly separated from orbital margin. Pterygostomian margin broadly round. Eye well developed, anterior end reaching to 0.55–0.65 (median 0.60) of first segment of antennular peduncle. Antennular peduncle 0.66–0.80 (median 0.77; Fig. 31A–C) as long as cl, first segment 1.68–2.05 (median 2.00) × as long as second segment, second segment 1.28–1.56 (median 1.42) × as long as third segment. Tooth on distolateral margin of first segment of antennular peduncle acute and well developed. Stylocerite reaching to 0.71–0.85 (median 0.82) of first segment of antennular peduncle. Scaphocerite 2.99–3.25 (median 3.19) × as long as wide, distal margin with short plumose setae.

***Branchial formula*** (*n* = 6). Podobranch on second maxilliped well developed. Third maxilliped possessed two small arthrobranchs. Pleurobranchs present on all pereopods.

***Mouthparts*** (*n* = 3). Mandible without palp (Fig. 32E); incisor process possessed four or five irregular teeth, and two rows of setae placed along inner margin; molar process truncated. Lower lacinia of maxillula round (Fig. 32F), with many rows of plumose setae; upper lacinia elongate, inner margin straight, with numerous conical setae and distal plumose setae; palp elongate, emerged from the base of upper lacinia, with one conical seta and few distal setae. Scaphognathite of maxilla wide anteriorly and tapering posteriorly (Fig. 32C), ending in a tuft of setae and short marginal setae placed along proximal triangular process; anterior margin with long plumose setae; palp slender; upper and middle endites subdivided with many rows of marginal setae; lower endite partly fused with middle endite, with relatively longer marginal setae.

Palp of first maxilliped subtriangular (Fig. 32D), ending in conspicuous finger-like projection, and with few plumose setae; caridean lobe broad, with marginal plumose setae; exopod well developed with distally marginal plumose setae; ultimate and penultimate segments of endopod indistinctly divided, with many marginal setae. Ultimate and penultimate segments of endopod of second maxilliped incompletely divided (Fig. 32B); inner margin of ultimate and penultimate segments with simple and pappose setae; exopod long and slender, with a tuft of long setae at the tip. Third maxilliped with epipod (Fig. 32A), reaching to end of second segment of antennular peduncle; ultimate segment of endopod, with a row of strong setae at proximal 2/3 of posterior margin, ending in one large claw and 6–10 spiniform setae on distal 1/3 of posterior margin, 1.02–1.07 (median 1.05, *n* = 5) × as long as penultimate segment; exopod long and slender, with a tuft of long setae at the tip.

***Pereopods*** (*n* = 6). Epipod well developed on first two pereopods, reduced in third pereopod, and absent in last two pereopods. Chelae of P1 and P2 well developed.

P1 short (Fig. 31E); chela 2.12–2.36 (median 2.21) × as long as wide, 0.99–1.15 (median 1.09) × as long as carpus, tips of fingers rounded, with tuft of setae near tip; dactylus 1.02–1.46 (median 1.14) × as long as palm; carpus hardly excavated distally, 3.10–3.78 (median 3.35) × as long as wide, 1.08–1.28 (median 1.17) × as long as merus; merus 2.70–3.29 (median 3.14) × as long as wide, 0.94–1.09 (median 1.00) × as long as ischium.

P2 slenderer, longer than P1 (Fig. 31F); chela 3.15–3.70 (median 3.30) × as long as wide, 0.65–0.79 (median 0.75) as long as carpus, tips of fingers rounded, with tuft of setae near tip; dactylus 1.18–1.41 (median 1.24) × as long as palm; carpus slender, 6.54–8.70 (median 8.01) × as long as wide, 1.49–1.67 (median 1.56) × as long as merus; merus 4.20–5.28 (median 4.59) × as long as wide, 0.94–1.10 (median 1.02) × as long as ischium.

P3 not sexually dimorphic (Fig. 31H); dactylus with six or seven (mode 7) spiniform setae on flexor margin (Fig. 31G), 3.82–4.57 (median 4.21) × as long as wide (including terminal claw), terminating with one large claw; propodus bearing numerous spiniform setae on posterior margin, 10.35–13.71 (median 11.90) × as long as wide, 2.98–3.37 (median 3.26) × as long as dactylus; carpus possessed three or four (mode 3) setae on posterior margin of outer surface, the distal seta largest, the other setae minute, 5.56–6.71 (median 5.81) × as long as wide, 0.52–0.66 (median 0.59) as long as propodus; merus possessed three or four (mode 3) large setae on posterior margin of outer surface, 7.40–9.40 (median 8.11) × as long as wide, 1.65–2.07 (median 1.97) × as long as carpus; ischium with one spiniform seta.

P5 slender (Fig. 31J); dactylus with 50–64 spiniform setae on flexor margin (Fig. 31I), 5.48–6.82 (median 5.77) × as long as wide (including terminal claw), terminating with one large claw; propodus bearing numerous spiniform setae on posterior margin, 10.63–15.15 (median 11.10) × as long as wide, 2.09–2.45 (median 2.18) × as long as dactylus; carpus possessed three or four (mode 3) setae on posterior margin of outer surface, the distal seta largest, the other setae minute, 4.73–6.85 (median 5.39) × as long as wide, 0.49–0.58 (median 0.53) as long as propodus; merus possessed two or three large setae on posterior margin of outer surface, 6.24–8.29 (median 6.38) × as long as wide, 1.43–1.67 (median 1.54) × as long as carpus; ischium without spiniform seta.

***Male pleopods*** (*n* = 6). Pl1 endopod subtriangular (Fig. 32K), wider proximally, 2.23–2.68 (median 2.38) × as long as wide, 0.30–0.36 (median 0.34) of exopod length, without appendix interna. Pl2 appendix masculina rod-shaped (Fig. 32L) with many setae, 0.52–0.76 (median 0.69) as long as endopod; appendix interna very slender, reaching 0.82–0.91 (median 0.85) of appendix masculina length.

***Abdomen*** (*n* = 6). Sixth abdominal somite ~ 0.48–0.65 (median 0.55) of cl, 1.45–1.74 (median 1.48) × as long as fifth somite, 0.89–0.98 (median 0.94) as long as telson (Fig. 31A). Telson 2.80–3.43 (median 3.04; Fig. 32I) × as long as wide, with three or four pairs of dorsal spiniform setae and one pair of dorsolateral spiniform setae; distal margin round without posteromedian projection, with 5–7 moveable plumose setae, the lateral pair longest (Fig. 32J). Preanal carina subtriangular, without spine (Fig. 32G). Uropodal diaeresis with 13–15 moveable spiniform setae (Fig. 32H).

***Embryos***. Ovigerous females with 16–34 embryos (mean 24, *n* = 6). Size of embryos with eyes 0.76–0.90 × 0.46–0.53 mm (*n* = 50).

###### Coloration.

Body grayish brown, with numerous dull brown spots. Rostrum is translucent, pale gray. Dorsally decorated with transverse sandy brown bands on each abdominal somite. Embryos are dark grayish brown (Fig. 23H).

###### Distribution and habitat.

Thailand. *Caridina tambralinga* sp. nov. is distributed in the southern region of the peninsula (Fig. 5F). Individuals inhabit both inland lotic and lentic freshwaters such as streams, lakes, lowland rivers, and ponds. They were found living on aquatic vegetation along the banks.

###### Phylogenetic placement.

The molecular analyses indicated that *C. tambralinga* sp. nov. is monophyletic (BS = 100%, BPP = 1.00; Fig. 4) and sister to *C. temasek* (BS = 100%, BPP = 1.00).

###### Etymology.

The specific name refers to *Tambralinga*—the ancient kingdom located on the Malay Peninsula, which existed from the 10^th^ to the 13^th^ centuries.

###### Remarks.

*Caridina tambralinga* sp. nov. is very similar to *C. temasek*. The close relationship between them is supported by both shared morphology and a robust phylogenetic relationship (BS = 100%, BPP = 1; Fig. 4). However, they can be distinguished by several key morphological characters, including features of the male first pleopod, rostrum length, and embryo size. The endopod of the male first pleopod of *C. tambralinga* sp. nov. lacks an appendix interna but this character is notably present in *C. temasek* (Choy and Ng 1991; Cai et al. 2007; this study). In addition, the rostrum of *C. tambralinga* sp. nov. is longer than that of *C. temasek* (0.70–0.88 as long as cl vs 0.57–0.64). and the embryo size is also larger (0.76–0.90 × 0.46–0.53 mm vs 0.59–0.67 × 0.34–0.41 mm) (this study).

*Caridina tambralinga* sp. nov. is also similar to *C. nangroma* sp. nov. but differs by having slenderer P1 carpus (3.10–3.78× as long as wide vs 2.33–2.88), slenderer P2 carpus (6.54–8.70× as long as wide vs 5.44–7.01), unarmed preanal carina (vs spine present), and the larger embryo (0.75–0.91 × 0.46–0.52 mm vs 0.68–0.75 × 0.37–0.49 mm).

##### Caridina kottelati

Taxon classificationAnimaliaDecapodaAtyidae

Cai & Naiyanetr, 2024

0DB0C50F-3041-5C87-892C-07A453B865ED

Fig. 5E

Caridina kottelati Cai & Naiyanetr, 2024: 790, fig. 8. Type locality southern Thailand: Narathiwat Province, stream ~ 2 km south of Ban Bu Ke Ta on road to Ban Sac, 5°49'24.7"N, 101°52'18.9"E.

###### Material examined.

None

###### Distribution and habitat.

Thailand. This species inhabits streams draining into the Golok River, the southeasternmost river forming the border between Thailand and Malaysia (Cai and Naiyanetr 2024).

###### Remarks.

In this study, we failed to obtain specimens of *C. kottelati*. Based on the original description provided by Cai and Naiyanetr (2024), the rostrum of *C. kottelati* is straight and extends to approximately the end of the antennular peduncle, with a rostral formula of (3–4) 13–14/ 3–5. Together with other key characters, such as the epipods (only present on P1 and P2) and the number of spiniform setae on uropodal diaeresis, these features suggest that this species likely belongs to the *C. laevis* species group. Further molecular analyses, however, are essential to confirm this preliminary placement.

*Caridina kottelati* is also notable for its very large embryos (up to 0.95 × 0.60 mm). Its distribution is also restricted to a few streams draining into the Golok River (Cai and Naiyanetr 2024). These two characteristics indicate *C. kottelati* as a landlocked species.

### Key to species of the freshwater shrimp genus *Caridina* in southern Thailand

**Table:** 

1	Rostrum without dorsal teeth (Figs 7A, 9A)	**2** (*C. typus* species group)
–	Rostrum with dorsal teeth	**3**
2	Rostrum relatively long, usually extending near or beyond the end of second segment of antennular peduncle (Fig. 7A, C). P1 dactylus 0.86–1.42× as long as palm (Fig. 7D). Appendix interna on endopod of male Pl1 shorter than endopod width (Fig. 7N).	***C. typus***
–	Rostrum relatively short, usually not extending beyond the end of second segment of antennular peduncle (Fig. 9A, C). P1 dactylus stout, 0.56–0.80× as long as palm (Fig. 9D). Appendix interna on endopod of male Pl1 slightly longer than endopod width (Fig. 9N)	***C. zhujiangensis***
3	Rostrum slender, curved upward, and obviously extending beyond the end of third segment of antennular peduncle (Figs 15A, 16A, 17A, 18A)	**4**
–	Rostrum short, variously shaped, not extending or slightly beyond the end of third segment of antennular peduncle (Figs 10A, 11A, 13A, 20A, 21A, 24A, 25A, 31A)	**7**
4	Rostrum very long, greatly extending beyond the end of third segment of antennular peduncle, ~ or > 1.5× as long as cl, strongly curved upwards (Fig. 15A). Segments of walking appendages slender. Telson very slender, with few plumose setae at the distal end (Fig. 14L–N). Uropodal diaeresis usually with < 10 moveable spiniform setae	***C. gracillima* or *C. gracilirostris^1^***
–	Rostrum long, extending beyond the end of third segment of antennular peduncle, ~ 1.0–1.5× as long as cl. (Figs 16A, 17A, 18A). Uropodal diaeresis usually with > 10 moveable spiniform setae	**5** (*C. nilotica* species group)
5	Endopod of male Pl1 with or without an appendix interna (Fig. 16N). Females carry numerous small-sized embryos (> 300 embryos per individual, 0.35–0.44 × 0.20–0.29 mm) (Fig. 17A, 18A). Preanal carina high with or without a spine (Figs 16K, 18L).	**6**
–	Endopod of male Pl1 without an appendix interna (Fig. 17N). Females carry a small number of large-sized embryos (< 100 embryos per individual, 0.79–0.98 × 0.49–0.60 mm) (Fig. 17A). Preanal carina low without a spine (Fig. 17K).	***C. macrophora***
6	Rostrum curved upward, dorsal teeth not placed beyond proximal 2/3 of dorsal margin (Fig. 16A). Preanal carina high, with a spine (Fig. 16K). P5 dactylus 4.33–6.41× as long as wide (Fig. 16H), with 47–64 spiniform setae on flexor margin.	***C. gracilipes***
–	Rostrum relatively straight or slightly curved upward, dorsal teeth usually placed throughout dorsal margin (Fig. 18C) or placed beyond proximal 2/3 of dorsal margin (Fig. 18A). Preanal carina subtriangular, without a spine (Fig. 18L). P5 dactylus 2.87–4.53× as long as wide (Fig. 18I), with 30–42 spiniform setae on flexor margin	***C. peninsularis***
7	Rostrum straight, or slightly pointed down, with a prominent basal ridge (Figs 10A, 11A, 13A). Epipods on the first four pereopods well developed. Endopod of male Pl1 always with appendix interna (Figs 10N, 13O)	**8**
–	Rostrum varies in shape, without a prominent basal ridge. Epipods on P2–P3 reduced or absent, epipod on P4 always absent. Endopod of male Pl1 with or without appendix interna.	**11** (*C. laevis* species group)
8	Rostrum slightly pointed down, provided similar width throughout the rostrum (Fig. 10A, C). Lateral plumose setae of distal end of telson longer than median plumose setae (Fig. 10L). Endopod of male Pl1 subtriangular with an appendix interna emerging near the tip (Fig. 10N)	**9** (*C. sumatrensis* species group)
–	Rostrum quite straight, wider at the middle and abruptly tapering toward the tip (Fig. 13A, C–D). Lateral plumose setae of distal end of telson shorter than median plumose setae (Fig. 13M). Endopod of male Pl1 long subtriangular with an appendix interna, emerging from distal 1/3 (Fig. 13P)	**10** (*C. lanceifrons* species group)
9	Rostrum usually reaching near the end of third segment of antennular peduncle (Fig. 10A). Telson with posteromedian projection (Fig. 10L). Females carry numerous small-sized embryos (> 300 embryos per individual, 0.38–0.52 × 0.28–0.34 mm) (Fig. 10A)	***C. sumatrensis***
–	Rostrum reaching near the end of second segment of antennular peduncle (Fig. 11A). Telson with greatly reduced or without posteromedian projection (Fig. 12J). Females carry a small number of very large-sized embryos (< 50 embryos per individual, 0.92–1.07 × 0.52–0.67 mm) (Fig. 11A)	***C. kuehnei* sp. nov**.
10	P5 dactylus with < 40 spiniform setae on flexor margin	***C. lanceifrons***
–	P5 dactylus with > 40 spiniform setae on flexor margin	***C. thai***
11	Telson relatively long, > 3.0× as long as wide. Endopod of male first pleopod without appendix interna (Fig. 26K).	**12**
–	Telson relatively stout, ~ 2.5–3.0× as long as wide. Endopod of male first pleopod with appendix interna (Fig. 21O).	***C. temasek***
12	Epipod only present on first two pereopods.	**13**
–	Epipod present on first two pereopods, reduced on third pereopod.	**15**
13	Rostrum sigmoidal. P2 carpus > 6.00× as long as wide.	**14**
–	Rostrum quite straight. P2 carpus < 6.00× as long as wide	***C. kottelati***
14	P5 dactylus with > 80 spiniform setae on flexor margin (Fig. 19I)	***C. laevis***
–	P5 dactylus with 50–70 spiniform setae on flexor margin (Fig. 19G)	***C. excavatoides***
15	Rostrum various in shape. Embryos large (> 0.50 × 0.35 mm)	**16**
–	Rostrum large, quite straight, reaching near or slightly beyond the end of third segment of antennular peduncle, wider at the middle and abruptly tapering toward the tip (Fig. 23A, C, D). Embryos small (0.37–0.46 × 0.24–0.31 mm).	***C. rangoona***
16	Rostrum narrow, not reaching or reaching slightly beyond the end of second segment of antennular peduncle (Figs 24A, 29A). Females carry a moderate number of medium-sized embryos (> 70 embryos per individual, ~ 0.60–0.70 × 0.35–0.45 mm)	**17**
–	Rostrum variously shaped, usually wider at the middle and tapering at the tip, reaching near the end of third segment of antennular peduncle (Figs 25A, 27A, 31A). Females carry a small number of large-sized embryos (< 50 embryos per individual, ~ 0.70–0.90 × 0.40–0.50 mm)	**18**
17	Rostrum long, usually reaching near the end of second segment of antennular peduncle, 0.62–0.75× as long as carapace length (Fig. 24A). Preanal carina low, with or without a spine (Fig. 24L)	***C. johnsoni***
–	Rostrum slightly convex, very short, usually reaching near the end of first segment of antennular peduncle, 0.44–0.52× as long as carapace length (Fig. 29A). Preanal carina high, fin-shaped, without a spine (Fig. 30G)	***C. takkola* sp. nov**.
18	Preanal carina high, with a spine (Figs 26G, 28G). Distal margin of telson subtriangular (Figs 26I, 28I).	**19**
–	Preanal carina low, without a spine (Fig. 32G). Distal margin of telson round (Fig. 32I)	***C. tambralinga* sp. nov**.
19	Rostrum large, straight or curved down, wider at the middle and abruptly tapering toward the tip (Fig. 25A, C–E). P1 chela 2.12–3.24× as long as wide (Fig. 25G). P2 chela 3.03–4.39× as long as wide. P2 carpus 5.44–7.01× as long as wide (Fig. 25H). Embryo size relatively small (0.68–0.75 × 0.37–0.49 mm) (Fig. 25A)	***C. nangroma* sp. nov**.
–	Rostrum minute, slightly pointed down, provided similar width throughout the rostrum (Fig. 27A). P1 chela 1.68–2.01× as long as wide (Fig. 27D). P2 chela 2.41–3.19× as long as wide. P2 carpus 4.78–5.61× as long as wide (Fig. 27E). Embryo size relatively large (0.80–0.88 × 0.47–0.58 mm) (Fig. 27A)	***C. srivijaya* sp. nov**.

## Discussion

Lanchester (1902) reported *C. multidentata* Stimpson, 1860 based on an examination of a single female collected during the “Skeat Expedition” with an uncertain locality. This specimen possesses a large number of rostral teeth and very large embryos of ~ 1 mm diameter. The exact taxonomic status of this specimen has been challenged since then. Cai et al. (2006) examined topotypes of *C. multidentata* from the Ogasawara Islands (Bonin). They redescribed the species and designated a neotype for the species. Subsequently, during a revision of Thai *Caridina*, Cai and Naiyanetr (2024) found that the description of *C. multidentata* provided by Lanchester (1902) did not correspond to any of their examined samples from Thailand. Moreover, it could not be matched to any of the documented *Caridina* species reported from the Malay Peninsula and surrounding areas. Therefore, Cai and Naiyanetr (2024) classified *C. multidentata sensu*Lanchester (1902) as an unknown species. In fact, the geographical extent of scientific collecting during the Skeat Expedition was substantial across the Malay Peninsula. As documented, the area probably spanned a large area from Thale Noi, Phatthalung Province, Thailand, southward to at least Selama River, Perak on the west coast, and Trengganu on the east coast of modern Malaysia (Annandale 1900; Lanchester 1902; Gibson-Hill et al. 1953). Consequently, this broad geographical scope led to a hypothesis that the specimen might have been collected outside of Thailand. Due to its limited morphological characters and molecular data, including the lack of a precise sampling locality, we suggest excluding this species from the current checklist of *Caridina* in southern Thailand until its true origin and identity can be confirmed.

In total, 21 species of *Caridina*, including five new species, have now been recorded from southern Thailand. Our findings revealed a higher-than-expected number of *Caridina* species in southern Thailand, suggesting that the number of species of *Caridina* in Thailand might have been substantially underestimated. Several basins in Thailand, particularly the Salween and headwater regions of the Chao Phraya (Ping, Wang, Yom, Nan, and Pasak rivers), remain largely unexplored for *Caridina* species. Furthermore, several *Caridina* species exhibit a stygophilic or stygobitic lifestyle, often showing high endemism and being restricted to single localities (Cai et al. 2009; Cai and Ng 2009; Li and Li 2010; Xu et al. 2020; Do et al. 2021a). Future surveys should thus prioritize these inland freshwaters and subterranean systems, which are likely to yield additional undescribed, landlocked, or cave-dwelling *Caridina* species.

### Morphological diagnostic characters in *Caridina*

Species delimitation of *Caridina* has been traditionally based on external morphological characters such as the shape of the rostrum, telson characters, the setal count of various appendages, the proportional length of appendage segments, the telson, and the embryo size (De Man 1892; Bouvier 1904, 1925; Kemp 1918b; Holthuis 1965, 1978; Richard and Clark 2005, 2010, 2014; Cai et al. 2007). However, defining robust species boundaries using this approach may be hindered by extensive interspecific similarity and intraspecific variation (De Man 1908b; Riek 1953; Holthuis 1965; Bouvier 1925; Jalihal et al. 1984; Chace 1997; Richard and Clark 2010, 2014; de Mazancourt et al. 2018a). More recently, molecular-based analyses have proven to be a valuable tool for overcoming this challenge, as demonstrated in the present study and some previous studies (Klotz and von Rintelen, 2014; de Mazancourt et al. 2018a, 2018b, 2019a, 2019b; Klotz et al. 2021, 2024). This integrative molecular approach allows systematists to critically re-evaluate the reliability of putative diagnostic morphological characters for accurate species delimitation within the genus *Caridina* (de Mazancourt et al. 2017b, 2018a). Based on this molecular framework, the following discussion evaluates specific diagnostic morphological characters of *Caridina* species, identifying those supported by genetic evidence as reliable identifiers and those that might be misleading or unreliable. This approach aims to refine the criteria for future species identification in this genus.

Although the plasticity of the rostrum has been mentioned in previous works (de Mazancourt et al. 2017a, 2018a), our current observations did not reveal any distinct forms of the rostrum related to different habitats or environments within the species examined in this study. However, notable intraspecific variation in the rostral shape has been detected in several southern Thai *Caridina* species, such as *C. excavatoides*, *C. johnsoni*, *C. lanceifrons*, *C. nangroma* sp. nov., *C. tambralinga* sp. nov., and *C. temasek*. These distinct rostral morphotypes often occurred sympatrically within species and were confirmed by molecular analyses to represent conspecific individuals.

Nevertheless, we found out that the rostral shape and its teeth arrangement are still reliable characters for species identification in most cases. For example, *C. sumatrensis* can be distinguished from *C. maeklongensis* and *C. kuehnei* sp. nov. in having a longer rostrum (reaching near the distal end of antennular peduncle vs reaching near the end of second segment of antennular peduncle). The rostrum of *C. peninsularis* has more dorsal teeth placed throughout the rostrum length, while that of *C. gracilipes* possesses a smaller number of dorsal teeth confined to proximal 2/3 of dorsal margin.

Furthermore, the rostrum is very useful for the preliminary classification of *Caridina* species into their respective species groups. Its shape remains relatively stable among closely related species within a given species group, and rostrum characteristics clearly differentiate members across various *Caridina* species groups. For example, the *C. typus* species group is defined by a dorsally unarmed rostrum. In contrast, species in the *C. gracilirostris* group possess a distinctively long, upcurved rostrum with subapical teeth, while those belonging to the *C. sumatrensis* group are characterized by a short, pointed-down rostrum with a prominent basal ridge.

The number of setae on each appendage and the proportions between appendage segments plays a key role in species identification (Cai et al. 2007; von Rintelen and Cai 2009; Do et al. 2020, 2021a, 2021b; Klotz et al. 2021, 2023, 2024), especially those in closely related species (Richard and Clark 2014; de Mazancourt et al. 2018a, 2020; Vijayamma et al. 2021). For example, *C. gracillima* is differentiated from *C. wilsoni* in having a greater number of spiniform setae on P5 dactylus (35–48 vs 21–35; Klotz et al. 2024). Furthermore, the segment proportions of P1 and P2 serve as a dominant distinguishing feature between close relatives. For example, the P1 dactylus of *C. typus* is relatively longer than that of *C. zhujiangensis* (0.86–1.42× as long as palm vs 0.56–0.80). The P1 chela of *C. srivijaya* sp. nov. is stouter than that of *C. nangroma* sp. nov. (1.68–2.01× as long as wide vs 2.12–3.24).

The presence or absence of an appendix interna on the endopod of the male first pleopod is a crucial and reliable diagnostic character for distinguishing between closely related *Caridina* species. For example, *C. tambralinga* sp. nov. lacks the appendix interna, distinguishing it from *C. temasek* (appendix interna present). Similarly, *C. macrophora* lacks the structure, separating it from both *C. peninsularis* and *C. gracilipes* (appendix interna present). Finally, within the *C. gracilirostris* species group, *C. neglecta* possesses an appendix interna, a feature that differentiates it from its close relatives that lack this structure, i.e., *C. gracillima*, *C. gracilirostris*, and *C. wilsoni* (Cai and Ng 2007; de Mazancourt et al. 2020; Klotz et al. 2024).

However, our observations revealed significant intraspecific variation of the appendix interna, which is occurring in two morphological states. In our collection, nearly all male specimens (> 90%) of *C. gracilipes* possessed an appendix interna, with < 10% lacking it. Interestingly, this variation occurred sympatrically across all *C. gracilipes* populations examined during this study. This variability has also been reported in other species, including *C. africana* Kingsley, 1883, *C. longirostris* H. Milne Edwards, 1837, and *C. nilotica* Roux, 1833 (Chace 1997; Richard and Clark 2005, 2010; Cai et al. 2007). This phenomenon in some species has been suggested to exhibit seasonal variation, associated with the breeding period (de Mazancourt et al. 2018a). Further investigation should focus on breeding experiments and multi-seasonal sampling to examine the proportion of males with and without appendix interna. Such studies would help to determine whether this variation is linked to the seasonal changes or reproductive cycles.

The presence or absence of epipods on walking legs is conserved in several species group. For example, well developed epipods on the first four pereopods are consistently found in *C. gracilirostris*, *C. lanceifrons*, *C. serrata*, *C. sumatrensis*, *C. typus*, and *C. weberi* species groups (de Mazancourt et al. 2020; Do et al. 2021b). In contrast, this character is highly variable in other species groups, such as in the *C. laevis* species group. While epipods on the last two pereopods are always absent in this species group, the epipods on P3 vary among species by consisting either reduced or absent states. This pattern is also evident in lacustrine *Caridina* in the ancient lakes of Sulawesi (von Rintelen and Cai 2009; Klotz et al. 2021). While epipods are consistently absent from the last two pereopods in these groups, the characteristic of epipods on P1–P3 is significantly variable among species. For example, *C. ensifera* Schenkel, 1902, *C. caerulea* von Rintelen & Cai, 2009, and *C. schenkeli* von Rintelen & Cai, 2009 possess well developed epipods on the first two pereopods, whereas the epipod of *C. spinata* Woltereck, 1937 and *C. striata* von Rintelen & Cai, 2009 is only present on P1. The epipods of *C. sarasinorum* Schenkel, 1902 are present on P1 but strongly reduced on P2. The epipods have completely disappeared in several species such as *C. longidigita* Cai & Wowor, 2007, *C. lilianae* Klotz, von Rintelen, Wowor, Lukhaup & von Rintelen, 2021, and *C. poso* Klotz, von Rintelen, Wowor, Lukhaup & von Rintelen, 2021.

In general, the shape of preanal carina is a reliable character for species identification (Cai et al. 2007; von Rintelen and Cai 2009; de Mazancourt et al. 2018a). However, the current study revealed that some species, such as *C. gracillima*, *C. johnsoni*, and *C. temasek* possess both armed and unarmed preanal carinae. This variability was confirmed as intraspecific variation by molecular analyses in the present study. Nevertheless, this character remains useful in several cases such as for the distinguishing between *C. nangroma* sp. nov. (preanal carina with a spine) and *C. tambralinga* sp. nov. (preanal carina without a spine), and between *C. macrophora* (preanal carina without a spine) and *C. gracilipes* (preanal carina with a spine).

The current study also confirmed the distinctness of the morphologically cryptic species between *C. gracillima* and *C. gracilirostris*, and between *C. maeklongensis* and *C. kuehnei* sp. nov. Although their morphological characters are largely overlapping, our molecular analyses confirmed all of these lineages as distinct species-level clades with high nodal supports (BS = 91–100%, BPP = 1.00).

In summary, the observed variability in key diagnostic characters, such as the rostral shape, appendix interna on male Pl1, and preanal carina, underscores the challenges of relying solely on morphological traits for distinguishing *Caridina* species. Because these features can be inconsistent in some cases, their use alone may result in ambiguous species delimitation. Consequently, this morphological instability emphasizes the importance of an integrated taxonomic approach that incorporates molecular analyses (e.g., DNA sequencing of the 16S rRNA gene, as demonstrated in this study). Genetic data provides robust, independent evidence that can assist taxonomists in establishing clear and defensible species boundaries within the genus.

### Embryos and life-histories in *Caridina*

The embryo size of freshwater shrimp can reflect their life-history strategies, which can be broadly categorized into amphidromous and landlocked species (Han et al. 2011; Hamasaki et al. 2021; de Mazancourt et al. 2023a). In amphidromous species, the female produces a large number of small embryos. Their prolonged planktonic larvae require saline water to complete development and migrate to inshore marine areas after hatching. This life cycle allows them to maintain broad geographic distributions and colonize distant areas (Bauer 2013; Yatsuya et al. 2013; de Mazancourt et al. 2021, 2023a; de Mazancourt and Ravaux 2024). In contrast, females of landlocked species spawn a small number of large embryos and spend their entire life in freshwater. Females of these species invest more heavily in yolk provisioning than in fecundity, producing relatively few but yolk-rich embryos that support a direct or rapid development of their lecithotrophic larvae (Page et al. 2008; Lai and Shy 2009; Han et al. 2011). As a result, their offspring are more likely to remain and establish populations in the same areas as their parents (Hamasaki et al. 2021). This reproductive strategy is thought to have evolved in response to limited food availability in freshwater environments and to reduce downstream larval loss in lotic habitats (Vogt 2013; Anger 2016; de Mazancourt et al. 2018a; Hamasaki et al. 2021; Bauer 2023).

The findings of the present study corroborate established knowledge regarding their reproductive traits and distribution patterns. Specifically, our field surveys suggest that Group I species (Fig. 6), including *C. gracilipes*, *C. gracillima*, *C. peninsularis*, *C. cf. rangoona*, *C. sumatrensis*, *C. typus*, and *C. zhujiangensis*, can be characterized as amphidromous based on their reproductive traits, which feature numerous small-sized embryos. In addition, they are consistently found in lowland rivers and streams connected to marine environment. These taxa typically exhibit wide geographic distributions spanning multiple watersheds or extensive coastal regions (Richard and Clark 2014; Bernardes et al. 2017; de Mazancourt et al. 2018a; Cai and Naiyanetr 2024).

A notable exception was found in *C. gracillima*, which is restricted to the Malay Peninsula and Sabah, and in *C. cf. rangoona*, which is confined to western coast of mainland Southeast Asia. A narrow-range distribution in species with small-sized embryos has been reported in previous works e.g. for *C. longicarpus* Roux, 1926 and *C. meridionalis* (endemic to New Caledonia; de Mazancourt et al. 2018a), *C. nana* de Mazancourt, Boseto, Marquet & Keith, 2020, *C. piokerai* de Mazancourt, Boseto, Marquet & Keith, 2020, and *C. turipi* (endemic to Solomon Islands; de Mazancourt et al. 2020), and *C. ponapensis* de Mazancourt, Marquet & Keith, 2024 (endemic to Pohnpei Island, Micronesia; de Mazancourt et al. 2024). It has been suggested that oceanic currents may play a significant role in limiting their larval dispersal, contributing to their localized ranges (de Mazancourt et al. 2021).

Furthermore, *C. gracillima* and *C. cf. rangoona* exhibit a relatively low number of embryos compared to other amphidromous species from southern Thailand (Group I; Fig. 6). Their restricted distribution and the number of embryos can be further explained by their alternative dispersal strategy. As recently discussed by de Mazancourt et al. (2023a), the term ‘amphidromy’ in freshwater faunas should be considered as a continuum rather than a discrete life-history trait, varying in larval duration, genetic structure, habitat requirement, and dispersal capacity. Based on our observed distribution, *C. gracillima* and *C. cf. rangoona* may exhibit a form of ‘reduced amphidromy,’ which could limit their dispersal compared to other small-embryo species that undergo ‘common amphidromy’. Ecologically, this strategy, characterized by a shorter planktonic larval phase (de Mazancourt et al. 2023a), may reduce the selective pressure to produce large numbers of embryos. Because their larvae are likely exposed to lower mortality than those species undergoing prolonged oceanic dispersal of ‘common amphidromous’ species, selection may favor reduced fecundity coupled with greater investment per offspring (McDowall 2007; Anger 2016). Morphologically, this reduced fecundity is likely a consequence of a smaller female body size in these two species, which limits the available abdominal space and constrains their capacity to carry a high number of small-sized embryos (Rao et al. 1981; Corey and Reid 1991; Hoffmann and Negreiros-Fransozo 2010).

In contrast, the members of Group III (Fig. 6), consisting of *C. macrophora*, *C. kuehnei* sp. nov., *C. nangroma* sp. nov., *C. srivijaya* sp. nov., and *C. tambralinga* sp. nov., can be characterized as landlocked species due to the production of few large-sized embryos. These species were typically collected from inland freshwater habitats. Interestingly, their distribution ranges are very variable. Several taxa, including *C. kuehnei* sp. nov., *C. srivijaya* sp. nov., and the previously studied *C. kottelati*, *C. maeklongensis*, and *C. panhai*, exhibit highly restricted ranges, being confined to a single location or watershed (Macharoenboon et al. 2023, 2024; Cai and Naiyanetr 2024; this study). The extreme endemism is commonly found in several species, such as cave-dwellers and lacustrine species that inhabit isolated freshwater habitats (von Rintelen and Cai 2009; Xu et al. 2020; Do et al. 2021a; Feng et al. 2021; Klotz et al. 2021).

Conversely, other landlocked species, such as *C. macrophora*, *C. nangroma* sp. nov., and *C. tambralinga* sp. nov., are widely distributed throughout the Malay Peninsula. This pattern of relatively broad distribution in species with low fecundity is not unique; similar distributions are reported for other taxa with few large-sized embryos, such as *C. babaulti* (distributed across central, western, and southern India; Pandya and Richard 2019) and *C. serrata* Stimpson, 1860 (distributed in southern China, Hong Kong, and Vietnam; Chen et al. 2020; Do et al. 2021b). These distribution patterns suggest that, although a low number of embryos is often associated with limited dispersal, some species characterized by a low number of embryos are nevertheless capable to dispersing among adjacent freshwater drainages or occupying a broader range of habitat conditions.

The current study also documented several *Caridina* species that fall into Group II (Fig. 6), characterized by the production of a moderate number of medium-sized embryos, consisting of *C. excavatoides*, *C. johnsoni*, *C. lanceifrons*, *C. takkola* sp. nov., and *C. temasek*. These species are likely to occur in lowland streams and rivers throughout the Malay Peninsula, except *C. lanceifrons* which is typically found in streams at middle and high courses. Although the number of embryos differences between the landlocked species (Group III) and these intermediate-trait species (Group II) were not statistically significant, a clear trend still emerged. Species in Group II possess a larger number of embryos compared to landlocked species (54–208 embryos per individual; mean 101 embryos vs 13–70 embryos per individual; mean 33 embryos), but a significantly smaller number than amphidromous species (Group I; 195–1483 embryos per individual; mean 758 embryos). Moreover, embryo size in Group II was significantly different from both the small-sized embryos of amphidromous species and the large-sized embryos of landlocked species. This intermediate reproductive pattern may align well with the “abbreviated type” of larval development (Lai and Shy 2009). This development mode is typically characterized by a shortened planktonic phase (fewer larval stages, usually 4–7 stages; Hayashi and Hamano 1984; Jalihal et al. 1984; Lai and Shy 2009). In addition, this abbreviated development is generally regarded as a transitional strategy between the “common type,” characterized by small-sized embryos, high fecundity, and numerous planktonic stages (usually 9–12 stages), and the “completely suppressed type”, featuring large-sized embryos, low fecundity, and an absence of planktonic stages (Hayashi and Hamano 1984; Lai and Shy 2009; Bauer 2023). However, as the present study did not include laboratory rearing, this hypothesis remains tentative. Future experimental studies documenting larval development under controlled conditions, particularly the exact number of larval stages, are required to confirm whether Group II species indeed exhibit abbreviated development.

However, embryonic development is a dynamic process that must be considered when comparing reproductive traits among species. As shown in previous studies, embryo size typically increases during embryogenesis (Anger et al. 2002; Wehrtmann and López 2003; Bradecina 2018). At the same time, the number of embryos usually decreases as development progresses due to brood mortality (Kuris 1991; Nazari et al. 2003; Herrera-Correal et al. 2013), implying that embryo counts reported herein likely reflect the minimum of the actual fecundity. These concurrent changes in size and number may obscure differences among reproductive groups when data from different developmental stages are compared. Thus, embryonic developmental stage should be standardized in interspecific comparisons. To address these limitations and ensure reliable interspecific comparisons, measurements in the present study were restricted to assessing late-stage (i.e., eyed embryos). Focusing on this specific embryonic stage minimizes the variation due to embryo developmental stage. This control ensures that the obtained counts represent the embryo numbers just prior to hatching, thereby facilitating meaningful comparisons of reproductive traits among Groups I, II, and III. Consequently, we recommend that future taxonomic, ecological, and life-history studies explicitly report embryonic stage of embryos analyzed to facilitate accurate and comparable assessments of reproductive traits across species.

Our findings further highlight a significant gap in our understanding of the evolution of life-history strategies in atyid shrimps. In fact, little is known about the relationships between reproductive traits (embryo size and the number of embryos), larval duration, and dispersal ability, especially in species with intermediate reproductive traits and in landlocked species. Therefore, future research integrating these aspects is therefore essential to improve our understanding of the evolution of reproductive strategies in this family.

Intraspecific variation in embryo size has been widely documented in some atyid species. For example, studies on *C. gracilipes* from Taiwan revealed distinct reproductive traits between lake (landlocked) and stream (amphidromous) populations. Lake populations produce larger embryos, while stream populations exhibit smaller embryo sizes (Han et al. 2011). A similar pattern has been observed in two populations of *C. meridionalis*, in which females from higher elevations produced significantly larger embryos than those from lower elevations (de Mazancourt et al. 2018a). In contrast, our observations of southern Thai *Caridina* populations revealed no comparable pattern of intraspecific reproductive plasticity in embryo size.

Despite the observed intraspecific variation, embryo size, including the number of embryos, remains conserved within species and is still a promising character for distinguishing between closely related species. For example, *C. kuehnei* sp. nov. is morphologically similar to *C. sumatrensis*. However, *C. kuehnei* sp. nov. bears a lower number of much larger embryos (0.92–1.07 × 0.52–0.67 mm, 18–26 embryos), compared to the smaller embryos and higher count of *C. sumatrensis* (0.38–0.52 × 0.23–0.34 mm, > 400 embryos). Similarly, *C. temasek* is similar to *C. tambralinga* sp. nov., but it possesses smaller embryos in a higher quantity (0.59–0.67 × 0.34–0.41 mm, 70–110 embryos) compared to the larger embryos and lower count of *C. tambralinga* sp. nov. (0.76–0.90 × 0.46–0.53 mm, 16–34 embryos). These clear distinctions highlight the utility of embryo-size traits in the classification of *Caridina*, specifically for distinguishing pairs of closely related species.

### Conservation implications

Due to the increasing loss of habitat and overharvesting for the ornamental pet trade, many *Caridina* species are currently facing serious threats (De Grave et al. 2015; de Mazancourt et al. 2021; de Mazancourt and Ravaux 2024; Sayer et al. 2025). Up to 56 *Caridina* species have been evaluated for their conservation status, ranging from Vulnerable (VU) to Critically Endangered (CR), according to the IUCN (2024). Notably, more than half of these assessed species are endemic. According to their habitat preferences, several *Caridina* species inhabit aquatic vegetation along riparian zones (Pillai 1964; Cai and Ng 2000; Yatsuya et al. 2013; Macharoenboon et al. 2024, 2025; this study). However, many river and stream banks in Thailand are now being fortified with concrete structures and ripraps (field observation), which has led to a significant decrease in this crucial riparian vegetation. The narrow distribution range of endemic and landlocked species makes them especially susceptible to a high risk of extinction (Régnier et al. 2009; Strayer and Dudgeon 2010; Giam et al. 2011). Those species, including *C. kottelati*, *C. kuehnei* sp. nov., and *C. srivijaya* sp. nov., require particular attention to preserve their highly localized habitats. On the other hand, amphidromous species of *Caridina* require unobstructed access to a variety of habitats from estuaries to headwater streams (de Mazancourt et al. 2021, 2023a; de Mazancourt and Ravaux 2024). Consequently, maintaining both riparian vegetation and the ecological connectivity between the high- and low-elevation watercourses is essential for their long-term survival (Yatsuya et al. 2013; de Mazancourt et al. 2021). Given the high rate of endemicity and the escalating pressures from human development, targeted conservation efforts are urgently needed to secure the diversity of the genus *Caridina* and the overall ecological health of Southeast Asian aquatic ecosystems.

## Supplementary Material

XML Treatment for Caridina

XML Treatment for Caridina typus

XML Treatment for Caridina zhujiangensis

XML Treatment for Caridina sumatrensis

XML Treatment for Caridina kuehnei

XML Treatment for Caridina lanceifrons

XML Treatment for Caridina thai

XML Treatment for Caridina gracilirostris

XML Treatment for Caridina gracillima

XML Treatment for Caridina gracilipes

XML Treatment for Caridina macrophora

XML Treatment for Caridina peninsularis

XML Treatment for Caridina laevis

XML Treatment for Caridina excavatoides

XML Treatment for Caridina temasek

XML Treatment for Caridina cf. rangoona

XML Treatment for Caridina johnsoni

XML Treatment for Caridina nangroma

XML Treatment for Caridina srivijaya

XML Treatment for Caridina takkola

XML Treatment for Caridina tambralinga

XML Treatment for Caridina kottelati

## Footnotes

Lacks a reliable character to distinguish between these two species (see remarks in C. gracillima).

## Additional information

### Artificial Intelligence (AI) use

The authors accept full responsibility for the content of the manuscript, including the disclosure of any use of AI.

Regarding the use of AI in the preparation of this manuscript, the authors declare the following: Gemini, GPT

Used for: Language, style and writing

### Author ORCIDs

K. Macharoenboon https://orcid.org/0009-0009-0888-5409

T. von Rintelen https://orcid.org/0000-0002-6253-3078

W. Klotz https://orcid.org/0000-0002-1104-5613

C. Sutcharit https://orcid.org/0000-0001-7670-9540

W. Siriwut https://orcid.org/0000-0001-6760-9724

E. Jeratthitikul https://orcid.org/0000-0002-3477-9548

### Data availability

All of the data that support the findings of this study are available in the main text or Supplementary Information.

## Supplementary materials

Supplementary material 1 List of samples, statistical analysis results, and material examined Data type docx Explanation note **table SS1**. List of samples used in phylogenetic analyses, with specimen vouchers and GenBank accession numbers. **table S2**. Results of One-Way ANOVA comparing mean embryo volume across species. **table S3**. Results of One-Way ANOVA comparing the mean number of embryos across species. **Data 1**. Material examined in the present study. https://binary.pensoft.net/file/1772122This dataset is made available under the Open Database License (http://opendatacommons.org/licenses/odbl/1.0/). The Open Database License (ODbL) is a license agreement intended to allow users to freely share, modify, and use this Dataset while maintaining this same freedom for others, provided that the original source and author(s) are credited. Kongkit Macharoenboon, Thomas von Rintelen, Werner Klotz, Chirasak Sutcharit, Warut Siriwut, Ekgachai Jeratthitikul
