# Supplementary material for: A taxonomic review of the freshwater shrimp genus Caridina H. Milne Edwards, 1837 (Decapoda, Atyidae) from southern Thailand, with descriptions of five new species
Source: Zookeys. 2026 Sep 15;1292:1–126. doi: 10.3897/zookeys.1292.193168 (PMC13598490; doi:10.3897/zookeys.1292.193168)
Supplement: Supplementary file 1 [file zookeys-1292-001_article-193168__-s001.docx]

**Supplementary Table 1** List of samples used in phylogenetic analyses, with specimen vouchers and GenBank accession numbers. n/a = data not available. Reference: 1 = Mazancourt *et al*. (2019); 2 = Klotz *et al*. (2019); 3 = Mazancourt *et al*. (2018a); 4 = Klotz and von Rintelen, (2014); 5 = de Mazancourt *et al*. (2020); 6 = Bernardes *et al*. (2017); 7 = Do *et al*. (2020); 8 = Macharoenboon *et al*. (2024); 9 = Macharoenboon *et al*. (2023); 10 = Xu *et al*. (2020); 11 = Macharoenboon *et al*. (2025); 12 = von Rintelen *et al*. (2012); 13 = Page *et al*. (2007a); 14 = Page *et al*. (2008); 15 = de Mazancourt *et al*. (2024); 16 = Page *et al.* (2007b).

| **Species** | **Voucher** | **Type Status** | **Locality** | **16S** | **COI** | **Reference** |
| --- | --- | --- | --- | --- | --- | --- |
| *Caridina appendiculata* | CA1670 |  | Australia | MH497534 | MK190059 | 1 |
| *Caridina appendiculata* | CA1708 |  | Australia | MK189903 | MK190063 | 1 |
| *Caridina* cf. *babaulti* | ZMB_30757_1 |  | West Bengal, India | MN399172 | n/a | 2 |
| *Caridina* cf. *babaulti* | ZMB_30757_2 |  | West Bengal, India | MN399173 | n/a | 2 |
| *Caridina brachydactyla* | CA1129 | Topotype | Sulawesi | MH497500 | n/a | 3 |
| *Caridina brachydactyla* | CA1130 | Topotype | Sulawesi | MH497501 | n/a | 3 |
| *Caridina breviata* | ZMB_DNA_1153 | Topotype | China | KP168718 | n/a | 4 |
| *Caridina breviata* | ZMB_DNA_1154 | Topotype | China | KP168719 | n/a | 4 |
| *Caridina cantonensis* | ZMB_DNA_1162 |  | Qingyuan Town, China | KP168720 | KP168802 | 4 |
| *Caridina cantonensis* | ZMB_DNA_1163 |  | Qingyuan Town, China | KP168721 | KP168803 | 4 |
| *Caridina elongapoda* | CA1899 |  | Malaysia | MK189924 | MK190085 | 1 |
| *Caridina excavatoides* | MUMNH-CAR490-F1 |  | Southern Thailand | PZ627272 | PZ627364 | This study |
| *Caridina excavatoides* | MUMNH-CAR616-F1 |  | Southern Thailand | PZ627273 | n/a | This study |
| *Caridina excavatoides* | ZMB_29686_1 |  | Southern Thailand | PZ675886 | PZ676184 | This study |
| *Caridina excavatoides* | ZMB_30195_3 |  | Southern Thailand | PZ675887 | PZ676185 | This study |
| *Caridina excavatoides* | ZMB_30196_3 |  | Southern Thailand | PZ675888 | PZ676186 | This study |
| *Caridina excavatoides* | ZMB_30198_1 |  | Southern Thailand | PZ675889 | PZ676187 | This study |
| *Caridina gracilipes* | CA1673 |  | Borneo | MH497535 | n/a | 3 |
| *Caridina gracilipes* | CA1694 |  | Australia | MH497540 | n/a | 3 |
| *Caridina gracilipes* | MUMNH-CAR252-F1 |  | Southern Thailand | PZ627275 | n/a | This study |
| *Caridina gracilipes* | MUMNH-CAR252-F2 |  | Southern Thailand | PZ627274 | n/a | This study |
| *Caridina gracilipes* | MUMNH-CAR462 |  | Southern Thailand | PZ627276 | n/a | This study |
| *Caridina gracilirostris* | MNHN-IU-2018-2804 |  | Kolombangara, Solomon Is. | MT303886 | n/a | 5 |
| *Caridina gracilirostris* | MNHN-IU-2018-2805 |  | Australia | MT303887 | n/a | 5 |
| *Caridina gracilirostris* | MNHN-IU-2018-2807 |  | Australia | MT303888 | n/a | 5 |
| *Caridina gracillima* | MUMNH-CAR431-F1 |  | Southern Thailand | PZ627277 | n/a | This study |
| *Caridina gracillima* | MUMNH-CAR449-F1 |  | Southern Thailand | PZ627278 | n/a | This study |
| *Caridina gracillima* | MUMNH-CAR497-F1 | Topotype | Southern Thailand | PZ627279 | n/a | This study |
| *Caridina gracillima* | MUMNH-CAR617-F1 |  | Southern Thailand | PZ627280 | n/a | This study |
| *Caridina gracillima* | MUMNH-CAR631-F1 |  | Southern Thailand | PZ627281 | n/a | This study |
| *Caridina gracillima* | MUMNH-CAR832-F1 |  | Southern Thailand | PZ627282 | n/a | This study |
| *Caridina gracillima* | ZMB_30691_1 |  | Southern Thailand | PZ675890 | n/a | This study |
| *Caridina gracillima* | ZMB_30691_2 |  | Southern Thailand | PZ675891 | n/a | This study |
| *Caridina gracillima* | ZMB_33501_1 |  | Southern Thailand | PZ675892 | n/a | This study |
| *Caridina grandirostris* | CA1891 |  | Japan | MH497551 | n/a | 3 |
| *Caridina grandirostris* | CA1892 |  | Japan | MH497552 | n/a | 3 |
| *Caridina jeani* | ZMB Crust. 29344-1 |  | Bali, Indonesia | KY069397 | KY069516 | 6 |
| *Caridina jeani* | ZMB Crust. 29416-1 |  | Halmahera, Indonesia | KY069410 | KY069529 | 6 |
| *Caridina jeani* | ZMB 29011_1 |  | Sulawesi | KY069383 | KY069502 | 6 |
| *Caridina* cf. *jeani* | CA1370 |  | Vanuatu | MK189867 | MK190028 | 1 |
| *Caridina* cf. *jeani* | CA1415 |  | Samoa | MK189877 | n/a | 1 |
| *Caridina johnsoni* | ZMB 30260 |  | Kien Giang, Vietnam | MT526817 | MT526836 | 7 |
| *Caridina johnsoni* | MUMNH-CAR380-F1 |  | Southern Thailand | PZ627283 | n/a | This study |
| *Caridina johnsoni* | MUMNH-CAR383-F1 |  | Southern Thailand | PZ627284 | n/a | This study |
| *Caridina johnsoni* | MUMNH-CAR470-F1 |  | Southern Thailand | PZ627285 | n/a | This study |
| *Caridina johnsoni* | MUMNH-CAR471-F1 |  | Southern Thailand | PZ627286 | PZ627365 | This study |
| *Caridina johnsoni* | MUMNH-CAR491-F1 |  | Southern Thailand | PZ627287 | PZ627366 | This study |
| *Caridina johnsoni* | MUMNH-CAR834-F1 |  | Southern Thailand | PZ627288 | n/a | This study |
| *Caridina johnsoni* | ZMB_30675_1 |  | Southern Thailand | PZ675893 | PZ676188 | This study |
| *Caridina johnsoni* | ZMB_30687_2 |  | Southern Thailand | PZ675894 | PZ676189 | This study |
| *Caridina johnsoni* | ZMB_30689_1 |  | Southern Thailand | PZ675895 | PZ676190 | This study |
| *Caridina johnsoni* | ZMB_30692_1 |  | Southern Thailand | PZ675896 | PZ676191 | This study |
| *Caridina johnsoni* | ZMB_30692_2 |  | Southern Thailand | PZ675897 | PZ676192 | This study |
| *Caridina johnsoni* | ZMB_30692_3 |  | Southern Thailand | PZ675898 | PZ676193 | This study |
| *Caridina johnsoni* | ZMB_30692_4 |  | Southern Thailand | PZ675899 | PZ676194 | This study |
| *Caridina kuehnei* sp. nov. | MUMNH-CAR438_H | Holotype | Southern Thailand | PZ627289 | n/a | This study |
| *Caridina kuehnei* sp. nov. | MUMNH-CAR438_F1 | Paratype | Southern Thailand | PZ627290 | PZ627367 | This study |
| *Caridina kuehnei* sp. nov. | MUMNH-CAR438_M1 | Paratype | Southern Thailand | PZ627291 | n/a | This study |
| *Caridina kuehnei* sp. nov. | ZMB_29671_1 | Paratype | Southern Thailand | PZ675900 | n/a | This study |
| *Caridina kuehnei* sp. nov. | ZMB_29671_2 | Paratype | Southern Thailand | PZ675901 | n/a | This study |
| *Caridina kuehnei* sp. nov. | ZMB_29672_1 | Paratype | Southern Thailand | PZ675902 | n/a | This study |
| *Caridina kuehnei* sp. nov. | ZMB_29672_2 | Paratype | Southern Thailand | PZ675903 | n/a | This study |
| *Caridina kuehnei* sp. nov. | ZMB_29672_3 | Paratype | Southern Thailand | PZ675904 | n/a | This study |
| *Caridina kuehnei* sp. nov. | ZMB_32388_1 | Paratype | Southern Thailand | PZ675905 | n/a | This study |
| *Caridina laevis* | ZMB_30683_1 |  | Southern Thailand | PZ675906 | PZ676195 | This study |
| *Caridina laevis* | ZMB_30702_1 |  | Southern Thailand | PZ675907 | PZ676196 | This study |
| *Caridina laevis* | ZMB_30702_2 |  | Southern Thailand | PZ675908 | PZ676197 | This study |
| *Caridina laevis* | ZMB_33504_1 |  | Southern Thailand | PZ675909 | n/a | This study |
| *Caridina laevis* | ZMB_33504_2 |  | Southern Thailand | PZ675910 | n/a | This study |
| *Caridina lanceifrons* | MUMNH-CAR139-F1 |  | Southern Thailand | PZ627292 | n/a | This study |
| *Caridina lanceifrons* | MUMNH-CAR411-F1 |  | Southern Thailand | PZ627293 | n/a | This study |
| *Caridina lanceifrons* | MUMNH-CAR426-F1 |  | Southern Thailand | PZ627294 | PZ627368 | This study |
| *Caridina lanceifrons* | MUMNH-CAR500-F1 |  | Southern Thailand | PZ627295 | PZ627369 | This study |
| *Caridina lanceifrons* | MUMNH-CAR500-M1 |  | Southern Thailand | PZ627296 | PZ627370 | This study |
| *Caridina lanceifrons* | MUMNH-CAR503-F1 |  | Southern Thailand | PZ627297 | PZ627372 | This study |
| *Caridina lanceifrons* | ZMB_30684_1 |  | Southern Thailand | PZ675911 | PZ676198 | This study |
| *Caridina lanceifrons* | ZMB_30684_2 |  | Southern Thailand | PZ675912 | PZ676199 | This study |
| *Caridina lanceifrons* | ZMB_32403_1 |  | Southern Thailand | PZ675913 | n/a | This study |
| *Caridina lanceifrons* | ZMB_32405_1 |  | Southern Thailand | PZ675914 | n/a | This study |
| *Caridina leucosticta* | CA1912 |  | Japan | MH497556 | n/a | 3 |
| *Caridina leucosticta* | CA1913 |  | Japan | MH497557 | n/a | 3 |
| *Caridina longicarpus* | CA1557 |  | New Caledonia | MK189892 | MK190051 | 1 |
| *Caridina longicarpus* | CA1599 |  | New Caledonia | MK189895 | MK190054 | 1 |
| *Caridina macrodentata* | CA1374 |  | Vanuatu | MK189869 | MK190030 | 1 |
| *Caridina macrodentata* | CA1375 |  | Vanuatu | MK189870 | MK190031 | 1 |
| *Caridina macrophora* | MUMNH-CAR099-F1 |  | Southern Thailand | PZ627298 | n/a | This study |
| *Caridina macrophora* | MUMNH-CAR453-F1 |  | Southern Thailand | PZ627299 | n/a | This study |
| *Caridina macrophora* | MUMNH-CAR458-F1 |  | Southern Thailand | PZ627300 | n/a | This study |
| *Caridina macrophora* | MUMNH-CAR833-F1 |  | Southern Thailand | PZ627301 | n/a | This study |
| *Caridina macrophora* | MUMNH-CAR853-F1 |  | Southern Thailand | PZ627302 | n/a | This study |
| *Caridina macrophora* | ZMB_29684_1 |  | Southern Thailand | PZ675915 | n/a | This study |
| *Caridina macrophora* | ZMB_30677_1 |  | Southern Thailand | PZ675916 | PZ676200 | This study |
| *Caridina macrophora* | ZMB_30693_1 |  | Southern Thailand | PZ675917 | PZ676201 | This study |
| *Caridina maeklongensis* | MUMNH-CAR064-4 | Paratype | Kanchanaburi, Thailand | PQ041487 | PQ042419 | 8 |
| *Caridina maeklongensis* | MUMNH-CAR064-7 | Paratype | Kanchanaburi, Thailand | PQ041486 | PQ042420 | 8 |
| *Caridina maeklongensis* | MUMNH-CAR064-9 | Holotype | Kanchanaburi, Thailand | PQ041485 | PQ042418 | 8 |
| *Caridina meridionalis* | CA1585 |  | New Caledonia | MK189894 | MK190053 | 1 |
| *Caridina meridionalis* | CA1620 |  | New Caledonia | MK189898 | MK190057 | 1 |
| *Caridina meridionalis* | CA048 |  | New Caledonia | MH497491 | n/a | 3 |
| *Caridina nangroma* sp. nov. | MUMNH-CAR404-H | Holotype | Southern Thailand | PZ627303 | n/a | This study |
| *Caridina nangroma* sp. nov. | MUMNH-CAR404-P1 | Paratype | Southern Thailand | PZ627304 | n/a | This study |
| *Caridina nangroma* sp. nov. | MUMNH-CAR404-P3 | Paratype | Southern Thailand | PZ627305 | n/a | This study |
| *Caridina nangroma* sp. nov. | MUMNH-CAR404-F1 | Paratype | Southern Thailand | PZ627306 | n/a | This study |
| *Caridina nangroma* sp. nov. | MUMNH-CAR484-F1 |  | Southern Thailand | PZ627307 | n/a | This study |
| *Caridina nangroma* sp. nov. | MUMNH-CAR492-F1 |  | Southern Thailand | PZ627308 | n/a | This study |
| *Caridina nangroma* sp. nov. | MUMNH-CAR598-F1 |  | Southern Thailand | PZ627309 | n/a | This study |
| *Caridina nangroma* sp. nov. | ZMB_29683_1 |  | Southern Thailand | PZ675918 | n/a | This study |
| *Caridina nangroma* sp. nov. | ZMB_30674_1 |  | Southern Thailand | PZ675919 | PZ676202 | This study |
| *Caridina nangroma* sp. nov. | ZMB_30682_1 |  | Southern Thailand | PZ675920 | PZ676203 | This study |
| *Caridina nangroma* sp. nov. | ZMB_30695_1 |  | Southern Thailand | PZ675921 | PZ676204 | This study |
| *Caridina nangroma* sp. nov. | ZMB_30696_1 |  | Southern Thailand | PZ675922 | PZ676205 | This study |
| *Caridina neglecta* | CA1703 |  | Vella Lavella, Solomon Is. | MT303889 | n/a | 5 |
| *Caridina neglecta* | CA1938 |  | Kolombangara, Solomon Is. | MT303890 | n/a | 5 |
| *Caridina neglecta* | CA1310 |  | Choiseul, Solomon Is. | MT303891 | n/a | 5 |
| *Caridina panhai* | MUMNH-CAR507-F1 | Paratype | Sakon Nakhon, Thailand | OQ092406 | OQ107450 | 9 |
| *Caridina panhai* | MUMNH-CAR507-F2 | Paratype | Sakon Nakhon, Thailand | OQ092407 | OQ107451 | 9 |
| *Caridina panhai* | MUMNH-CAR507-M1 | Paratype | Sakon Nakhon, Thailand | OQ092405 | OQ107452 | 9 |
| *Caridina paratypus* | MNHN-IU-2018-2925 | Holotype | Malaita, Solomon Is. | MT303934 | n/a | 5 |
| *Caridina paratypus* | MNHN-IU-2018-2926 | Paratype | Vanuatu | MT303935 | n/a | 5 |
| *Caridina peninsularis* | MUMNH-CAR228-F1 |  | Southern Thailand | PZ627310 | n/a | This study |
| *Caridina peninsularis* | MUMNH-CAR451-F1 |  | Southern Thailand | PZ627311 | n/a | This study |
| *Caridina peninsularis* | MUMNH-CAR459-F1 |  | Southern Thailand | PZ627312 | n/a | This study |
| *Caridina peninsularis* | MUMNH-CAR633-F1 |  | Southern Thailand | PZ627313 | n/a | This study |
| *Caridina peninsularis* | MUMNH-CAR783-F1 |  | Southern Thailand | PZ627314 | n/a | This study |
| *Caridina peninsularis* | ZMB_30685_1 |  | Southern Thailand | PZ675923 | PZ676206 | This study |
| *Caridina peninsularis* | ZMB_30685_2 |  | Southern Thailand | PZ675924 | PZ676207 | This study |
| *Caridina peninsularis* | ZMB_32097_1 |  | Southern Thailand | PZ675925 | n/a | This study |
| *Caridina peninsularis* | ZMB_33505_1 |  | Southern Thailand | PZ675926 | n/a | This study |
| *Caridina peninsularis* | ZMB_33506_1 |  | Southern Thailand | PZ675927 | n/a | This study |
| *Caridina rangoona* | MUMNH-CAR154-F1 |  | Southern Thailand | PZ627315 | n/a | This study |
| *Caridina rangoona* | MUMNH-CAR434-F1 |  | Southern Thailand | PZ627316 | n/a | This study |
| *Caridina rangoona* | MUMNH-CAR436-F1 |  | Southern Thailand | PZ627317 | n/a | This study |
| *Caridina rangoona* | MUMNH-CAR454-F1 |  | Southern Thailand | PZ627318 | PZ627371 | This study |
| *Caridina rangoona* | MUMNH-A187-6 |  | Southern Thailand | PZ627319 | n/a | This study |
| *Caridina rangoona* | MUMNH-A191-1 |  | Southern Thailand | PZ627320 | n/a | This study |
| *Caridina serrata* | isolate 137 |  | Zhuhai, China | MT446454 | MN701595 | 10 |
| *Caridina serrata* | isolate 138 |  | Zhuhai, China | MT446455 | MN701596 | 10 |
| *Caridina simoni* | CA1831 |  | Sri Lanka | MK189920 | MK190081 | 1 |
| *Caridina simoni* | CA1833 |  | Sri Lanka | MK189921 | MK190082 | 1 |
| *Caridina sinanensis* | isolate 286 |  | Sinan Guizhou, China | MT434874 | MT433963 | 10 |
| *Caridina sinanensis* | isolate 287 |  | Sinan Guizhou, China | MT434875 | MT433964 | 10 |
| *Caridina sirindhornae* | MUMNH-CAR683-F1 |  | Mukdahan, Thailand | PV364275 | PV364386 | 11 |
| *Caridina sirindhornae* | MUMNH-CAR683-F2 |  | Mukdahan, Thailand | PV364276 | PV364387 | 11 |
| *Caridina sirindhornae* | MUMNH-CAR683-F3 |  | Mukdahan, Thailand | PV364277 | PV364388 | 11 |
| *Caridina srivijaya* sp. nov. | MUMNH-CAR502-F1 | Paratype | Southern Thailand | PZ627321 | PZ627373 | This study |
| *Caridina srivijaya* sp. nov. | MUMNH-CAR502-F2 | Paratype | Southern Thailand | PZ627322 | PZ627374 | This study |
| *Caridina srivijaya* sp. nov. | MUMNH-CAR502-F3 | Paratype | Southern Thailand | PZ627323 | PZ627375 | This study |
| *Caridina srivijaya* sp. nov. | MUMNH-CAR502-M1 | Paratype | Southern Thailand | PZ627324 | PZ627376 | This study |
| *Caridina srivijaya* sp. nov. | MUMNH-CAR502-M2 | Paratype | Southern Thailand | PZ627325 | PZ627377 | This study |
| *Caridina sumatrensis* | MUMNH-CAR054-F1 |  | Ratchaburi, Thailand | PQ041492 | PQ042425 | 8 |
| *Caridina sumatrensis* | ZMB_29491 |  | Southern Thailand | FN995360 | n/a | 12 |
| *Caridina sumatrensis* | MUMNH-CAR440-F1 |  | Southern Thailand | PZ627326 | PZ627378 | This study |
| *Caridina sumatrensis* | MUMNH-CAR463-F1 |  | Southern Thailand | PZ627327 | PZ627379 | This study |
| *Caridina sumatrensis* | MUMNH-CAR780-F1 |  | Southern Thailand | PZ627328 | PZ627380 | This study |
| *Caridina sumatrensis* | ZMB_30681_1 |  | Southern Thailand | PZ675928 | PZ676208 | This study |
| *Caridina sumatrensis* | ZMB_30697_1 |  | Southern Thailand | PZ675929 | PZ676209 | This study |
| *Caridina sumatrensis* | ZMB_31994_1 |  | Southern Thailand | PZ675930 | n/a | This study |
| *Caridina takkola* sp. nov. | MUMNH-CAR469F | Paratype | Southern Thailand | PZ627329 | PZ627382 | This study |
| *Caridina takkola* sp. nov. | MUMNH-CAR469M | Paratype | Southern Thailand | PZ627330 | PZ627383 | This study |
| *Caridina takkola* sp. nov. | MUMNH-CAR495 |  | Southern Thailand | PZ627331 | PZ627383 | This study |
| *Caridina takkola* sp. nov. | ZMB_30690-1 |  | Southern Thailand | PZ675931 | PZ676210 | This study |
| *Caridina takkola* sp. nov. | ZMB_30690-2 |  | Southern Thailand | PZ675932 | PZ676211 | This study |
| *Caridina tambralinga* sp. nov. | MUMNH-CAR456-F1 |  | Southern Thailand | PZ627332 | n/a | This study |
| *Caridina tambralinga* sp. nov. | MUMNH-CAR461-H | Holotype | Southern Thailand | PZ627333 | PZ627384 | This study |
| *Caridina tambralinga* sp. nov. | MUMNH-CAR461-M1 | Paratype | Southern Thailand | PZ627334 | PZ627385 | This study |
| *Caridina tambralinga* sp. nov. | MUMNH-CAR476-F1 |  | Southern Thailand | PZ627335 | n/a | This study |
| *Caridina tambralinga* sp. nov. | MUMNH-CAR477-F1 |  | Southern Thailand | PZ627336 | PZ627386 | This study |
| *Caridina tambralinga* sp. nov. | MUMNH-CAR489-F1 |  | Southern Thailand | PZ627337 | PZ627387 | This study |
| *Caridina tambralinga* sp. nov. | MUMNH-CAR494-F1 |  | Southern Thailand | PZ627338 | PZ627388 | This study |
| *Caridina tambralinga* sp. nov. | ZMB_30698_2 |  | Southern Thailand | PZ675933 | PZ676212 | This study |
| *Caridina tambralinga* sp. nov. | ZMB_30704_1 |  | Southern Thailand | PZ675934 | PZ676213 | This study |
| *Caridina tambralinga* sp. nov. | ZMB_31662_1 |  | Southern Thailand | PZ675935 | n/a | This study |
| *Caridina temasek* | MUMNH-CAR812-F2 |  | Southern Thailand | PZ627339 | n/a | This study |
| *Caridina temasek* | MUMNH-CAR835-F1 |  | Southern Thailand | PZ627340 | n/a | This study |
| *Caridina temasek* | MUMNH-CAR835-M1 |  | Southern Thailand | PZ627341 | n/a | This study |
| *Caridina temasek* | ZMB_29687_1 |  | Southern Thailand | PZ675936 | n/a | This study |
| *Caridina temasek* | ZMB_29687_2 |  | Southern Thailand | PZ675937 | n/a | This study |
| *Caridina temasek* | ZMB_32130_2 |  | Southern Thailand | PZ675938 | n/a | This study |
| *Caridina thai* | MUMNH-CAR861-F1 | Topotype | Southern Thailand | PZ627342 | PZ627389 | This study |
| *Caridina thai* | MUMNH-CAR861-F2 | Topotype | Southern Thailand | PZ627343 | PZ627390 | This study |
| *Caridina tricincta* | ZMB_DNA_667 |  | Ha Giang, Vietnam | MT526823 | MT526840 | 7 |
| *Caridina tricincta* | ZMB_DNA_668 |  | Ha Giang, Vietnam | MT526824 | MT526841 | 7 |
| *Caridina trifasciata* | MNHN-IU-2014-20876 |  | Zuhai, China | KP168765 | KP168795 | 4 |
| *Caridina trifasciata* | MNHN-IU-2014-20883 |  | Zuhai, China | KP168766 | KP168796 | 4 |
| *Caridina turipi* | MNHN-IU-2014-20876 | Holotype | Choiseul, Solomon Is. | MT303911 | n/a | 5 |
| *Caridina turipi* | MNHN-IU-2014-20883 | Paratype | Choiseul, Solomon Is. | MT303910 | n/a | 5 |
| *Caridina typus* | MEFGL_CT_Sey01 |  | Seychelles | KY069374 | KY069493 | 6 |
| *Caridina typus* | OUMNH.ZC-2010-22-0001 |  | Mascarene Islands, Mauritius | KY069380 | KY069499 | 6 |
| *Caridina typus* | ZMB Crust. 29344-1 |  | Bali, Indonesia | KY069397 | KY069516 | 6 |
| *Caridina typus* | ZMB Crust. 29416-1 |  | Halmahera, Indonesia | KY069410 | KY069529 | 6 |
| *Caridina typus* | ZMB 29011_1 |  | Sulawesi | KY069383 | KY069502 | 6 |
| *Caridina typus* | ZMB_Crust_29489-2 |  | Langkawi, Malaysia | KY069416 | KY069535 | 6 |
| *Caridina typus* | MUMNH-CAR321-F1 |  | Southern Thailand | PZ627344 | n/a | This study |
| *Caridina typus* | MUMNH-CAR532-F1 |  | Southern Thailand | PZ627345 | PZ627391 | This study |
| *Caridina typus* | MUMNH-CAD051 |  | Southern Thailand | PZ627346 | n/a | This study |
| *Caridina viladolidi* | ZMBunid1227 |  | Mindoro, Philippines | KY436222 | n/a | 6 |
| *Caridina viladolidi* | ZMBunid968 |  | Mindoro, Philippines | KY436220 | n/a | 6 |
| *Caridina weberi* | MNHN-IU-2018-2867 |  | Kolombangara, Solomon Is. | MT303920 | n/a | 5 |
| *Caridina weberi* | MNHN-IU-2018-2868 |  | Papua New Guinea | MT303921 | n/a | 5 |
| *Caridina weberi* | MNHN-IU-2018-2869 |  | Papua New Guinea | MT303922 | n/a | 5 |
| *Caridina wilsoni* | GUC567 |  | Australia | DQ478530 | n/a | 13 |
| *Caridina wilsoni* | GUC584 |  | Australia | DQ478531 | n/a | 13 |
| *Caridina wilsoni* | GUC996 |  | Australia | DQ478532 | n/a | 13 |
| *Caridina zhujiangensis* | isolate 22 | Topotype | Zhuhai, China | MT446448 | MN701603 | 10 |
| *Caridina zhujiangensis* | isolate 23 | Topotype | Zhuhai, China | MT446449 | MN701604 | 10 |
| *Caridina zhujiangensis* | CT_Sar02 |  | Sarawak, Malaysia | KY069345 | KY069464 | 6 |
| *Caridina zhujiangensis* | DW0850a |  | Taliabu, Indonesia | KY069351 | KY069470 | 6 |
| *Caridina zhujiangensis* | ZMBunid1258 |  | Mindoro, Philippines | KY069435 | n/a | 6 |
| *Caridina zhujiangensis* | MUMNH-CAR464-F1 |  | Southern Thailand | PZ627347 | PZ627392 | This study |
| *Caridina zhujiangensis* | ZMB_28069_1 |  | Southern Thailand | PZ675939 | n/a | This study |
| *Caridina zhujiangensis* | ZMB_28069_2 |  | Southern Thailand | PZ675940 | n/a | This study |
| **outgroup** |  |  |  |  |  |  |
| *Atya innocous* | GUEL1 |  | Rio Espiritu Santo, Puerto Rico | EF489987 | EF489968 | 14 |
| *Atya scabra* | ZMB_DNA510 |  | Rio Guarumo, Panama | EF489985 | EF489964 | 14 |
| *Atyoida bisulcata* | GU755 |  | Waiau Stream, Hawaii | DQ681278 | DQ681257 | 15 |
| *Atyoida chacei* | CA1858 |  | Babeldaob, Palau | PP455330 | n/a | 16 |
| *Atyoida pilipes* | GU991 |  | Moorea, French Polynesia | DQ681279 | DQ681255 | 15 |
| *Atyopsis moluccensis* | KZ222 |  | Petshop in Germany | DQ681281 | DQ681246 | 15 |
| *Atyopsis spinipes* | GU877 |  | Guadalcanal, Solomon Is. | DQ681282 | DQ681260 | 15 |

**Supplementary Table 2** Results of One-Way ANOVA Comparing mean embryo volume across species

|  | Df | Sum Sq | Mean Sq | F-value | P-value |
| --- | --- | --- | --- | --- | --- |
| Species | 16 | 2.1515 | 0.13447 | 1865 | **<2e-16** |
| Residuals | 833 | 0.0601 | 0.00007 |  |  |
| Total | 849 | 2.2116 |  |  |  |

**Supplementary Table 3** Results of One-Way ANOVA Comparing the mean number of embryos across species

|  | Df | Sum Sq | Mean Sq | F-value | P-value |
| --- | --- | --- | --- | --- | --- |
| Species | 16 | 16777335 | 1048583 | 50.97 | **<2e-16** |
| Residuals | 101 | 2077720 | 20571 |  |  |
| Total | 117 | 18855055 |  |  |  |

**Literature cited (Table S1)**

Bernardes, S.C., Pepato, A.R., von Rintelen, T., von Rintelen, K., Page, T.J., Freitag, H. and de Bruyn, M. 2017. The complex evolutionary history and phylogeography of *Caridina typus* (Crustacea: Decapoda): long-distance dispersal and cryptic allopatric species. Scientific Reports, 7(1): 9044. https://doi.org/10.1038/s41598-017-08494-w

de Mazancourt, V., Boseto, D., Marquet, G. and Keith, P. 2020. Solomon’s Gold Mine: Description or redescription of 24 species of *Caridina* (Crustacea: Decapoda: Atyidae) freshwater shrimps from the Solomon Islands, including 11 new species. European Journal of Taxonomy, 696: 1–86. https://doi.org/10.5852/ejt.2020.696

de Mazancourt, V., Klotz, W., Marquet, G. and Keith, P. 2018. Integrative taxonomy helps separate four species of freshwater shrimps commonly overlooked as *Caridina longirostris* (Crustacea : Decapoda : Atyidae) on Indo-West Pacific islands. Invertebrate Systematics, 32(6): 1422–1447. https://doi.org/10.1071/IS18034

de Mazancourt, V., Klotz, W., Marquet, G., Mos, B., Rogers, D.C. and Keith, P. 2019. The complex study of complexes: The first well-supported phylogeny of two species complexes within genus *Caridina* (Decapoda: Caridea: Atyidae) sheds light on evolution, biogeography, and habitat. Molecular Phylogenetics and Evolution, 131: 164–180. https://doi.org/https://doi.org/10.1016/j.ympev.2018.11.002

de Mazancourt, V., Marquet, G. and Keith, P. 2024. An integrative taxonomic revision of the freshwater atyid shrimps (Crustacea: Decapoda: Caridea) of Micronesia. Diversity, 16(4): 200. https://doi.org/https://doi.org/10.3390/d16040200

Do, V.T., von Rintelen, T. and Dang, V.D. 2020. Descriptions of two new freshwater shrimps of the genus *Caridina* H. Milne Edwards, 1837 (Crustacea: Decapoda: Atyidae) from northern Vietnam. Raffles Bulletin of Zoology, 68: 404–420. https://doi.org/10.26107/RBZ-2020-0057

Klotz, W. and von Rintelen, T. 2014. To "bee" or not to be - On some ornamental shrimp from Guangdong Province, Southern China and Hong Kong SAR, with descriptions of three new species. Zootaxa, 3889: 151–184. https://doi.org/10.11646/zootaxa.3889.2.1

Klotz, W., von Rintelen, T. and Christodoulou, M. 2019. Middle East *Caridina* (Decapoda: Atyidae): Redescription of *C. fossarum* Heller, 1862, description of three new species, and remarks on the status of *C. syriaca* Bouvier, 1904 and C. babaulti basrensis Al-Adhub & Hamzah, 1987. Zoologischer Anzeiger, 283: 161–185. https://doi.org/https://doi.org/10.1016/j.jcz.2019.08.008

Macharoenboon, K., Manonai, V. and Jeratthitikul, E. 2024. *Caridina maeklongensis*, a new landlocked freshwater shrimp species (Crustacea: Decapoda: Atyidae) from the Mae Klong Basin, Thailand. THE RAFFLES BULLETIN OF ZOOLOGY, 72: 450–468.

Macharoenboon, K., Sutcharit, C., Siriwut, W. and Jeratthitikul, E. 2023. A new species of land-locked freshwater shrimp genus *Caridina* (Decapoda: Atyidae) from Middle Mekong Basin, Thailand. Tropical Natural History, 23(7): 229–241. https://li01.tci-thaijo.org/index.php/tnh/article/view/257589

Macharoenboon, K., Sutcharit, C., Siriwut, W. and Jerratthitikul, E. 2025. *Caridina sirindhornae* sp. nov., a new landlocked atyid species (Decapoda: Atyidae) from the Middle Mekong Basin, Thailand. . Tropical Natural History.

Page, T.J., Cook, B.D., von Rintelen, T., von Rintelen, K. and Hughes, J.M. 2008. Evolutionary relationships of atyid shrimps imply both ancient Caribbean radiations and common marine dispersals. Journal of the North American Benthological Society, 27(1): 68–83. https://doi.org/10.1899/07-044r.1

Page, T.J., von Rintelen, K. and Hughes, J.M. 2007a. An island in the stream: Australia’s place in the cosmopolitan world of Indo-West Pacific freshwater shrimp (Decapoda: Atyidae: Caridina). Molecular Phylogenetics and Evolution, 43(2): 645–659. https://doi.org/https://doi.org/10.1016/j.ympev.2006.08.007

Page, T.J., von Rintelen, K. and Hughes, J.M. 2007b. Phylogenetic and biogeographic relationships of subterranean and surface genera of Australian Atyidae (Crustacea:Decapoda:Caridea) inferred with mitochondrial DNA. Invertebrate Systematics, 21(2): 137–145. https://doi.org/https://doi.org/10.1071/IS06023

von Rintelen, K., Page, T.J., Cai, Y., Roe, K., Stelbrink, B., Kuhajda, B.R., Iliffe, T.M., Hughes, J. and von Rintelen, T. 2012. Drawn to the dark side: A molecular phylogeny of freshwater shrimps (Crustacea: Decapoda: Caridea: Atyidae) reveals frequent cave invasions and challenges current taxonomic hypotheses. Molecular Phylogenetics and Evolution, 63(1): 82–96. https://doi.org/https://doi.org/10.1016/j.ympev.2011.12.015

Xu, D.J., Li, D.X., Zheng, X.Z. and Guo, Z.L. 2020. *Caridina sinanensis*, a new species of stygobiotic atyid shrimp (Decapoda, Caridea, Atyidae) from a karst cave in the Guizhou Province, southwestern China. ZooKeys, 1008: 17–35. https://doi.org/10.3897/zookeys.1008.54190

**Material examined in the present study**

***Caridina typus* H. Milne Edwards, 1837**

**Materials.** THAILAND • 5 ♀, cl. 5.31–6.79 mm, 5 ♂, cl. 3.97–4.65 mm, Phuket Province, Thalang District, Bang Prae Stream; 8.0383°N, 98.4034°E; MUMNH-CAR093, CAR532 (used for measurement) • 2 ♀, cl. 4.80–5.12 mm, 1 ♂, cl. 3.66 mm, Ranong Province, Mueang District, Bang Rin Stream; 9.9186°N, 98.6298°E; MUMNH-CAR321 • 3 ♀, cl. 5.60–6.40 mm, 3 ♂, cl. 4.19–4.35mm, Ranong Province, Mueang District, Ngao Noi Stream; 9.8309°N, 98.6247°E; MUMNH-CAR591, CAD051.

***Caridina zhujiangensis* Chen *et al.* 2018**

**Materials.** THAILAND • 10 ♀, cl. 5.39–6.37 mm, 10 ♂, cl. 3.30–4.13 mm, Nakhon Si Thammarat Province, Khanom District, Khao Phlai Dam; 9.0905°N, 99.9052°E; MUMNH-CAR464 (used for measurement) • Nakhon Si Thammarat Province, Khanom District, Khao Phlai Dam; 9.0852°N, 99.9070°E; ZMB 28069.

***Caridina sumatrensis* De Man, 1892**

**Materials.** THAILAND • 1 ♀, cl. 4.00 mm, Ranong Province, La-un District, Rawee Stream; 10.0666°N, 98.7248°E; MUMNH-CAR106 • 18 ♀, cl. 4.11–5.23 mm, 6 ♂, cl. 2.93–3.87 mm, Ranong Province, Mueang District, Ngao Noi Stream; 9.8309°N, 98.6247°E; MUMNH-CAR178, CAR217, CAR219 • 3 ♀, cl. 3.32–3.86 mm, 2 ♂, cl. 3.01–3.24, Krabi Province, Khlong Thom District, Khlong Thom Stream; 7.9181°N, 99.1865°E; MUMNH-CAR197 • 8 ♀, cl. 4.76–5.82 mm, 4 ♂, 3.12–3.79 mm Phuket Province, Thalang District, Bang Prae Stream; 8.0383°N, 98.4034°E; MUMNH-CAR198, CAR430 (used for measurement) • 2 ♀, cl. 4.24–4.29 mm, Prachuap Khiri Khan Province, Kui Buri District, Kui Buri River; 12.0920°N, 99.7130°E; MUMNH-CAR238 • 4 ♀, cl. 3.89–4.43 mm, Krabi Province, Mueang District, Krabi Stream; 8.1229°N, 98.9253°E; MUMNH-CAR291 • 1 ♀, cl. 4.48 mm, Phetchaburi Province, Tha Yang District, Yang Chum Stream; MUMNH-CAR294 • 5 ♀, cl. 3.88–4.25 mm, Ranong Province, Mueang District, Bang Rin Stream; 9.9186°N, 98.6298°E; MUMNH-CAR317 • 5 ♀, cl. 3.72–4.30 mm, Chumphon Province, Sawi District, Sawi Stream; 10.2237°N, 99.0120°E; MUMNH-CAR322 • 4 ♀, cl. 4.05–4.71 mm, Ranong Province, Kra Buri District, Lan Liang Stream; MUMNH-CAR340 • 4 ♀, cl. 3.83–4.15 mm, Prachuap Khiri Khan Province, Hua Hin District, Pran Buri River; 12.4784°N, 99.7982°E; MUMNH-CAR379 • 2 ♀, cl. 3.90–4.18 mm, Prachuap Khiri Khan Province, Hua Hin District, Pa Lao Stream; 12.5066°N, 99.5173°E; MUMNH-CAR414 • 1 ♀, cl. 4.03 mm, Prachuap Khiri Khan Province, Kui Buri District, Kui Stream; 12.0561°N, 99.8601°E; MUMNH-CAR417 • 1 ♀, cl. 3.64 mm Surat Thani Province, Don Sak District; 9.078371°N, 99.7147°E MUMNH-CAR420 • 1 ♀, cl. 3.89 mm, Chumphon Province, Tha Sae District, Kaeng Pega Stream; 10.7027°N, 99.2049°E; MUMNH-CAR421 • 3 ♀, cl. 4.75–5.82 mm, 1 ♂, cl. 2.81 mm, Phang Nga Province, Takua Pa District, Bang Niang Stream; 8.6658°N, 98.2774°E; MUMNH-CAR427 (used for measurement) • 3 ♀, cl. 3.71–4.00 mm, Phang Nga Province, Takuatung District, Khum Mut Stream; 8.3907°N, 98.2995°E; MUMNH-CAR432 • 2 ♀, cl. 4.08–4.63 mm, Phang Nga Province, Mueang District, Menora Stream; 8.5096°N, 98.5357°E; MUMNH-CAR435 • 5 ♀, cl. 4.15–4.88 mm, 1 ♂, cl. 3.53 mm, Krabi Province, Khlong Thom District, Thom Stream (connected to Emerald Pool); 7.9230°N, 99.2598°E; MUMNH-CAR439 • 5 ♀, cl. 4.04–4.80 mm, 3 ♂, cl. 3.10–3.30 mm, Trang Province, Kantang District, Huai Dam Stream; 7.4032°N, 99.4352°E; MUMNH-CAR440 (used for measurement) • 4 ♀, cl. 3.70–4.20 mm, 3 ♂, cl. 3.02–3.39 mm, Trang Province, Palian District, Lak Khan Stream; 7.2190°N, 99.7183°E; MUMNH-CAR443 (used for measurement) • 5 ♀, cl. 4.08–5.73 mm, Satun Province, La-ngu District, La-ngu Stream; 6.9069°N, 99.8115°E; MUMNH-CAR452 • 4 ♀, cl. 4.47–5.52 mm, Nakhon Si Thammarat Province, Khanom District, Khao Phlai Dam; MUMNH-CAR463 (used for measurement) • 5 ♀, cl. 4.55–5.30 mm, Phang Nga Province, Takuatung District, Khum Mut Stream; 8.3953°N, 98.4324°E; MUMNH-CAR634 • 5 ♀, cl. 3.98–4.72 mm, Phang Nga Province, Takua Pa District, Takua Pa River; 8.8541°N, 98.3541°E; MUMNH-CAR636 • 1 ♀, cl. 4.30 mm Satun Province, Mueang District, Chalung Stream; 6.7379°N, 100.0609°E; MUMNH-CAR638 • 5 ♀, cl. 4.40–4.80 mm, 4 ♂, cl. 3.46–3.98 mm, Satun Province, Manang District, La-ngu Stream; MUMNH-CAR780 (used for measurement) • 2 ♀, cl. 4.08–4.22 mm Surat Thani Province, Khiri Rat Nikhom District, Hin Kong Stream; 8.8427°N, 98.9824°E; MUMNH-CAR782, CAR793 • 1 ♀, cl. 3.92 mm, Surat Thani Province, Phrasaeng District, I-Pun Stream 8.6212°N, 98.9666°E; MUMNH-CAR790 • 3 ♀, cl. 4.81 5.24 mm, Trang Province, Sikao District, Ang-Thong Stream; 7.5511°N, 99.4184°E; MUMNH-CAR805 • 1 ♀, cl. 3.70 mm, Prachuap Khiri Khan Province, Kui Buri District, Kui Buri River; 12.0915°N, 99.8049°E; MUMNH-CAR814 • 1 ♀, cl. 3.77 mm, Trang Province, Mueang District, Trang River; 7.612°N, 99.5185°E; MUMNH-CAR815 • Trang Province, Palian District, Lak Khan Stream; 7.2331°N, 99.7256°E; ZMB 30681 • Trang Province, Na Yong District, Ton Nam Piew Stream; 7.5486°N, 99.7881°E; ZMB 30697 • Surat Thani Province, Phanom District, Klong Phanom-Khlong Sok; 8.8867°N, 98.6683°E; ZMB 32420 • Krabi Province, Mueang District; 8.1583°N, 98.7833°E; ZMB 31994.

***Caridina kuehnei* sp. nov.**

**Materials.** THAILAND • 4 ♀ cl. 2.74–3.50, mm; 3 ♂, cl. 2.23–2.78 mm Krabi Province, Khlong Thom District, Thom Stream (connected to Emerald Pool); 7.9230°N, 99.2598°E; MUMNH-CAR438.

***Caridina lanceifrons* Yu, 1936**

**Materials.** THAILAND • 1 ♀, cl. 4.29 mm, Trang Province, Wang Wiset District; 7.889367°N, 99.33343°E; MUMNH-CAR139 • 1 ♀, cl. 4.10 mm, Phetchaburi Province, Tha Yang District, Yang Chum Stream; 12.7821°N, 99.6788°E; MUMNH-CAR332 • 1 ♀, cl. 4.08 mm, Prachuap Khiri Khan Province, Kui Buri District, Kui Stream; 12.0561°N, 99.8601°E; MUMNH-CAR337 • 5 ♀, cl. 4.41–4.75 mm, 5 ♂, cl. 2.41–2.69 mm, Prachuap Khiri Khan Province, Bang Saphan District, Sai Khu Waterfall; MUMNH-CAR411 (used for measurement) • 3 ♀, cl. 3.90–4.45 mm, 2 ♂, cl. 2.29–2.32 mm, Chumphon Province, Mueang District, Hin Wang Stream, 10.4827°N, 98.9209°E; MUMNH-CAR426 (used for measurement) • 5 ♀, cl. 3.91–4.47 mm, 3 ♂, cl. 2.50–2.84 mm, Chumphon Province, Pathio District, Kra Por Stream; 10.7474°N, 99.2275°E; MUMNH-CAR472 (used for measurement) • Chumpon Province, Phato District, Nake Stream; 9.9050°N, 98.8686°E; MUMNH-CAR473 • Nakhon Si Thammarat Province, Nopphitam District, Waad Stream; 8.8045°N, 99.5884°E; MUMNH-CAR479 • 5 ♀, cl. 4.14–4.75 mm, Phatthalung Province, Si Banphot District, Tha-nae Stream; 7.7071°N, 99.8412°E; MUMNH-CAR485 • 1 ♀, cl. 3.60 mm, Songkhla Province, Khlong Hoi Khong District, Chum Sai Stream; 6.8109°N, 100.3184°E; MUMNH-CAR499 • 3 ♀, cl. 3.63–4.18 mm, 3 ♂, cl. 2.06–2.22 mm, Songkhla Province, Rattaphum District, Ton Stream; 7.1432°N, 100.1231°E; MUMNH-CAR500 (used for measurement) • 3 ♀, cl. 4.11–4.68 mm, 3 ♂, cl. 2.10-2.35 mm, Chumphon Province, Mueang District, Wisai Stream10.3754°N, 99.0606°E; MUMNH-CAR503 (used for measurement) • 5 ♀, cl. 3.92–4.50 mm, 1 ♂, cl. 2.53 mm, Phang Nga Province; Mueang District, Lar Stream; 8.5301°N, 98.5205°E; MUMNH-CAR537 • 1 ♀, cl. 4.13 mm, Nakhon Si Thammarat, Thung Song District, Tha-Luang Stream; 8.0549°N, 99.6424°E; MUMNH-CAR639 • Nakhon Si Thammarat Province, Bang Khan District, Lam Dang Stream; 7.9499°N, 99.5296°E; MUMNH-CAR802 • Nakhon Si Thammarat Province, Chang Klang District, River after limestone cave (-200m); 8.3342°N, 99.6514°E; ZMB 29674, ZMB 32403 • Nakhon Si Thammarat Province, Sichon District, Tha Ruea Ri Stream; 9.0078°N, 99.8217°E; ZMB 30684 • Satun Province, La-ngu District; 6.9000°N, 99.8000°E; ZMB 32405 • Songkhla Province, Rattaphum District, Ton Stream; 7.1432°N, 100.1231°E; ZMB 33503.

***Caridina thai* Cai & Naiyanetr, 2024**

**Materials.** THAILAND • **Topotype** • 6 ♀, cl. 3.15–4.23 mm, Nakhon Ratchasima, Pak Chong District, Muak Lek Waterfall; 14.6424°N, 101.2051°E; MUMNH-CAR861.

***Caridina gracillima* Lanchester, 1902**

**Materials.** THAILAND • **Topotype** • 5 ♀, cl. 4.09–4.89 mm, 3 ♂, cl. 3.20–3.89 mm, Songkhla Province, Hat Yai District, Thale Sap Songkhla (Songkhla Lake); 7.1552°N, 100.4686°E; MUMNH-CAR497 (used for measurement). **Other Materials •** 2 ♀, cl. 3.41–3.58 mm, Trang Province, Mueang District, Thung Khai Stream, 7.4496°N, 99.6343°E; MUMNH-CAR080 • 1 ♀, cl. 3.41–3.58 mm, Songkhla Province, Sathing Phra District; 7.4239°N, 100.4183°E; MUMNH-CAR140 • 1 ♀, cl. 3.99 mm, Krabi Province, Khlong Thom District, Pela Stream; 8.0094°N, 99.1291°E; MUMNH-CAR270 • 1 ♀, cl. 3.40 mm, Chumphon Province, Sawi District, Sawi Stream; 10.2237°N, 99.0120°E; MUMNH-CAR272 • 3 ♀, cl. 3.31–3.57 mm, Phetchaburi Province, Tha Yang District, Yang Chum Stream; 12.7821°N, 99.6788°E; MUMNH-CAR295 • 3 ♀, cl. 3.28–3.83 mm, Phetchaburi Province, Tha Yang District, Phet Dam; 12.9138°N, 99.8559°E; MUMNH-CAR297 • 1 ♀, cl. 3.05 mm, Chumphon Province, Tha Sae District, Tha Sae Stream; 10.6891°N, 99.1771°E; MUMNH-CAR303 •1 ♀, cl. 3.64 mm, Ranong Province, La-un District, La-un Stream; 10.1097°N, 98.7595°E; MUMNH-CAR305 • 2 ♀, cl. 3.22–3.71 mm, Prachuap Khiri Khan Province, Bang Saphan District, Wang Hin Stream; 11.3243°N, 99.5538°E; MUMNH-CAR314 • 2 ♀, cl. 3.24–3.60 mm, Prachuap Khiri Khan Province, Kui Buri District, Kui Buri River; 12.0920°N, 99.7130°E; MUMNH-CAR323 • 1 ♀, cl. 3.08 mm, Phetchaburi Province, Nong Ya Plong District, Mae Prachan Stream; 13.1092°N, 99.7230°E, MUMNH-CAR368 • 1 ♀, cl. 2.96 mm, Prachuap Khiri Khan Province, Hua Hin District, Pran Buri River; 12.4784°N, 99.7982°E; MUMNH-CAR382 • 1 ♀, cl. 3.11 mm, Prachuap Khiri Khan Province, Hua Hin District, Pran Buri River; 12.6049°N, 99.8591°E; MUMNH-CAR392 • 1 ♀, cl. 3.23 mm, Prachuap Khiri Khan Province, Kui Buri District, Kui Stream; 12.0561°N, 99.8601°E; MUMNH-CAR396 • 10 ♀, cl. 3.37–4.30 mm, 5 ♂, cl. 2.46–3.01 mm, Phang Nga Province, Takuatung District, Khum Mut Stream; 8.3907°N,98.2995°E; MUMNH-CAR431 (used for measurement) • 5 ♀, cl. 3.45–4.00 mm, 2 ♂, cl. 2.25–2.80 mm, Trang Province, Kantang District, Huai Dam Stream; 7.40324°N, 99.4351°E; MUMNH-CAR441 • 5 ♀, cl. 3.80–4.46 mm, 2 ♂, cl. 2.49–3.90 mm, Satun Province, La-ngu District, La-ngu Stream; 6.9069°N, 99.8115°E; MUMNH-CAR449 (used for measurement) • 5 ♀, cl. 3.82–4.35 mm, Surat Thani Province, Mueang District, Phun Phin Stream; 9.1936°N, 99.2816°E; MUMNH-CAR468 (used for measurement) • 2 ♀, cl. 3.13–3.38 mm, 2 ♂, cl. 2.06–2.29 mm, Nakhon Sri Thammarat Province, Cha-uat District, Wangkiean Stream; 7.9750°N, 99.9170°E; MUMNH-CAR482 • 1 ♀, cl. 3.46 mm, Songkhla Province, Ranot District, Thale Noi Non-Hunting Area; 7.7838°N, 100.2031°E; MUMNH-CAR488 • 1 ♀, cl. 3.40 mm, Phatthalung Province, Mueang District, Lampam Stream; 7.6259°N, 100.1577°E; MUMNH-CAR493 • 5 ♀, cl. 3.45–4.00 mm, 2 ♂, cl. 2.25–2.80 mm, Nakhon Sri Thammarat Province, Thung Song District, Tha-Luang Stream; 08.0550°N, 99.6424°E; MUMNH-CAR614 • 5 ♀, cl. 3.39–4.27 mm, 3 ♂, cl. 2.51–2.62 mm, Nakhon Sri Thammarat, Bangkhan District, Trang River; 7.9786°N, 99.6041°E; MUMNH-CAR617 • 2 ♀, cl. 3.22–3.40 mm, Nakhon Sri Thammarat, Bangkhan District, Trang River; 7.9451°N, 99.5781°E; MUMNH-CAR619 • 3 ♀, cl. 3.15–3.93 mm, 2 ♂, cl. 2.37–2.99 mm, Trang Province, Mueang District, Trang River; 7.6117°N, 99.5188°E; MUMNH-CAR622 • 10 ♀, cl. 3.80–4.44 mm, 5 ♂, cl. 2.76–3.25 mm, Trang Province, Kantang District, Trang River; 7.4341°N, 99.5153°E; MUMNH-CAR625 • 5 ♀, cl. 3.72–4.05 mm, 2 ♂, cl. 2.75–3.02 mm, Trang Province, Mueang District, Trang River; 7.5957°N, 99.5807°E; MUMNH-CAR626 • 3 ♀, cl. 3.60–3.97 mm, Trang District, Huai Yot District, Trang River 7.8153°N, 99.5317°E; MUMNH-CAR628 • 10 ♀, cl. 3.68–4.69 mm, 5 ♂, cl. 2.55–3.43 mm, Krabi Province, Mueang District, Tha Hindarn Stream; 8.1585°N, 98.7843°E; MUMNH-CAR631 (used for measurement) • 5 ♀, cl. 3.49–4.04 mm, Phang Nga Province, Takuatung District, Khum Mut Stream; 8.3953°N, 98.4324°E; MUMNH-CAR632 • 3 ♀, cl. 3.57–3.88 mm, Phang Nga Province, Takua Pa District, Takua Pa River; 8.8541°N, 98.3541°E; MUMNH-CAR637 • 2 ♀, cl. 3.32–3.56 mm, Prachuap Khiri Khan Province, Hua Hin District, Pran Buri River; 12.4779°N, 99.7982°E; MUMNH-CAR640 • 4 ♀, cl. 3.57–4.76 mm, 2 ♂, cl. 3.09–3.30 mm, Sawi Stream, Chumphon Province, Sawi District; 10.2236°N, 99.0120°E; MUMNH-CAR703 • 3 ♀, cl. 3.69–3.85 mm, Surat Thani Province, Chai Buri District, Sok Stream; 8.5068°N, 99.0992°E; MUMNH-CAR796 • 10 ♀, cl. 3.41–4.09 mm, 5 ♂, cl. 2.83–3.59 mm, Krabi Province, Nuea Khlong District, Pakasai Stream; 8.0264°N, 99.0449°E; MUMNH-CAR798 • 1 ♀, cl. 3.67 mm, Nakhon Si Thammarat Province, Bang Khan District, Lam Dang Stream; 7.9499°N, 99.5296°E; MUMNH-CAR801 • 3 ♀, cl. 3.52–4.00 mm, Prachuap Khiri Khan Province, Kui Buri District, Kui Buri River; 12.0915°N, 99.8049°E; MUMNH-CAR813 • 1 ♀, cl. 3.64, Ranong Province, Kra Buri District, Pak Chan Stream; 10.5272°N, 98.8338°E; MUMNH-CAR816 • 1 ♀, cl. 3.75, Trang Province, Yan Ta Khao District, Thung Khai Stream; 7.4495°N, 99.6343°E; MUMNH-CAR818 • 5 ♀, cl. 3.32–3.99 mm, Narathiwat, Tak Bai District, Irrigation Canal; 6.1628°N, 102.0827°E; MUMNH-CAR820 • 10 ♀, cl. 3.28–4.55 mm, 5 ♂, cl. 3.26–3.60 mm, Pattani Province, Mueang District, Pattani River; 6.8214°N, 101.2447°E; MUMNH-CAR832 • Songkhla Province, Thale Noi District, Marshland E-SE of Thale Noi; 7.7839°N, 100.2015°E; ZMB 30676 • Nakhon Sri Thammarat Province, Hua Sai District; 8.0172°N, 100.3006°E; ZMB 30686 • Phatthalung Province, Mueang District, Lampam Stream; 7.6261°N, 100.1558°E; ZMB 30688 • Surat Thani Province, Mueang District, Phunphin Stream; 9.1906°N, 99.2814°E; ZMB 30691 • Pattani Province, Nong Chik District; 6.8069°N, 101.1514°E; ZMB 30694 • Surat Thani Province, Mueang District, Phunphin Stream; 9.1906°N, 99.2814°E; ZMB 33501.

***Caridina gracilipes* De Man, 1892**

**Materials.** THAILAND • 1 ♀, cl. 3.88 mm, Ranong Province, Mueang District, Bang Rin Stream; 9.9186°N, 98.6298°E; MUMNH-CAR196 • 8 ♀, cl. 2.26–3.28 mm Prachuap Khiri Khan Province, Bang Saphan District, Wang Hin Stream; 11.3243°N, 99.5538°E; MUMNH-CAR210 • 10 ♀, cl. 4.00–4.51 mm, 5 ♂, cl. 2.00–2.39 mm, Songkhla Province, Chana District; 7.0118°N, 100.7279°E; MUMNH-CAR218, CAR252 (used for measurement) • 4 ♀, cl. 3.50–4.37 mm, 2 ♂, cl. 2.16–2.25 mm, Ranong Province, Kapoe District; 9.61°N, 98.4791°E; MUMNH-CAR247 (used for measurement) • 1 ♀, cl. 3.15 mm, Prachuap Khiri Khan Province, Sam Roi Yot District; 12.3017°N, 99.9373°E; MUMNH-CAR326 • 1 ♀, cl. 3.58 mm, Prachuap Khiri Khan Province, Hua Hin District, Pran Buri River; 12.4780°N, 99.7982°E; MUMNH-CAR375 • 5 ♀, cl. 3.98–4.66 mm, Nakhon Si Thammarat Province, Sichon District, Thathon Stream; 8.8876°N, 99.8997°E; MUMNH-CAR460 • 10 ♀, cl. 4.42–5.37 mm, 5 ♂, cl. 3.06–3.52 mm, Nakhon Si Thammarat Province, Khanom District, Khao Phlai Dam; 9.0905°N, 99.9052°E; MUMNH-CAR462, CAR465 (used for measurement) • Trang Province, Palian District, Lak Khan Stream; 7.2190°N, 99.7183°E; MUMNH-CAR567.

***Caridina macrophora* Kemp, 1918**

**Materials.** THAILAND • 2 ♀, cl. 3.20–3.40 mm, Phang Nga Province, Mueang District, Bok Pui Stream; 8.5739°N, 98.4142°E; MUMNH-CAR086 • 5 ♀, cl. 3.00–3.51 mm, 3 ♂, cl. 2.35–2.72 mm, Phetchaburi Province, Nong Ya Plong District, Mae Prachan Stream; 13.1092°N, 99.7230°E; MUMNH-CAR099 • Phetchaburi Province, Tha Yang District, Phetchaburi River; 12.9487°N, 99.8544°E; MUMNH-CAR124 • 2 ♀, cl. 3.04–3.28 mm, Trang Province, Mueang District, Bang Rak; 7.5392°N, 99.5900°E; MUMNH-CAR138 • 1 ♀, cl. 3.00 mm, Chumphon Province, Tha Sae District, Tha Sae Stream; 10.6892°N, 99.1771°E; MUMNH-CAR151 • 5 ♀, cl. 2.96–3.58 mm, 2 ♂, cl. 2.22–2.76 mm, Surat Thani Province, Chaiya District, Talat Chaiya; 9.3917°N, 99.18722°E; MUMNH-CAR156 • 1 ♀, cl. 3.30 mm, Songkhla Province, Sathing Phra District, Chathing Phra Stream; 7.4738°N, 100.4259°E; MUMNH-CAR301 • 1 ♀, cl. 3.19 mm, Trang Province, Wang Wiset district, Chee Stream; 7.7844°N, 99.3990°E; MUMNH-CAR304 • 1 ♀, cl. 3.00 mm, Satun Province, Mueang District, Nong Plak Phra Ya; 6.7436°N, 100.0386°E; MUMNH-CAR 324 • 2 ♀, cl. 3.35–3.48 mm, Ranong Province, La-un District, Rawee Stream; 10.0666°N, 98.7248°E; MUMNH-CAR327 • 4 ♀, cl. 3.30–3.46 mm, Satun Province, La-ngu District, La-ngu Stream; 6.9069°N, 99.8115°E; MUMNH-CAR453 • 10 ♀, cl. 2.80–3.75 mm, 5 ♂, cl. 2.15–2.51 mm, Nakhon Si Thammarat Province, Sichon District, Thathon Stream; 8.8876°N, 99.8997°E; MUMNH-CAR458 (used for measurement) • 1 ♀, cl. 3.50 mm, Nakhon Si Thammarat Province, Sichon District, Tha-kwai Stream; 8.9467°N, 99.7567°E; MUMNH-CAR475 • 1 ♀, cl. 3.39 mm, Nakhon Si Thammarat Province, Lan Saka District, Yuad Stream; 8.3861°N, 99.8167°E; MUMNH-CAR481 • 2 ♀, cl. 3.25–3.47 mm, Nakhon Si Thammarat Province, Cha-uat District, Wangkiean Stream; 7.9750°N, 99.9170°E; MUMNH-CAR483 • 6 ♀, cl. 3.11–3.64 mm, 3 ♂, cl. 2.28–2.45 mm, Phatthalung Province, Si Banphot District, Tha-nae Stream; 7.7071°N, 99.8412°E; MUMNH-CAR486 (used for measurement) • 5 ♀, cl. 3.32–3.88 mm, 3 ♂, cl. 2.15–2.61 mm, Songkhla Province, Khlong Hoi Khong District, Chum Sai Stream; 6.8109°N, 100.3184°E; MUMNH-CAR498 (used for measurement) • 8 ♀, cl. 3.10–3.58 mm, 5 ♂, cl. 2.18–2.64 mm, Nakhon Si Thammarat Province, Bang Khan District, Lamdung Stream (dam); 8.0710°N, 99.5288°E, MUMNH-CAR504 (used for measurement) • 2 ♀, cl. 3.12–3.50 mm, Nakhon Sri Thammarat Province, Thung Song District, Tha-Luang Stream; 08.0550°N, 99.6424°E; MUMNH-CAR615 • 3 ♀, cl. 3.49–3.59 mm, Trang Province, Mueang District, Trang River; 7.6117°N, 99.5188°E; MUMNH-CAR623 • 5 ♀, cl. 3.39–3.72 mm, 3 ♂, cl. 2.35–2.90 mm, Trang District, Huai Yot District, Trang River 7.8153°N, 99.5317°E; MUMNH-CAR629 • 2 ♀, cl. 3.19–3.32 mm, Nakhon Si Thammarat Province, Phrom Khiri District, unnamed pond; 8.5185°N, 99.9238°E; MUMNH-CAR642 • 1 ♀, cl. 3.41 mm, Surat Thani Province, Phrasaeng District, I-Pun Stream 8.6212°N, 98.9666°E; MUMNH-CAR791 • 1 ♀, cl. 3.01 mm, Trang Province, Huai Yot District, Thale Song Hong Lake; 7.8418°N, 99.4784°E; MUMNH-CAR799 • 2 ♀, cl. 3.38–3.65 mm, Nakhon Si Thammarat Province, Bang Khan District, Lam Dang Stream; 7.9499°N, 99.5296°E; MUMNH-CAR800 • 1 ♀, cl. 3.51 mm, Trang Province, Sikao District, Ang-Thong Stream; 7.5511°N, 99.4184°E; MUMNH-CAR806 • 7 ♀, cl. 3.13–3.76 mm, 4 ♂, cl. 2.30–2.86 mm, Songkhla Province; Hat Yai District, Wat Stream; 6.9812°N, 100.2996°E; MUMNH-CAR810 (used for measurement) • 2 ♀, cl. 3.44–3.69 mm, Phang Nga Province, Kapong District, Thap Yao Stream; 8.7138°N, 98.4930°E; MUMNH-CAR819 • 3 ♀, cl. 3.30–3.60 mm, 2 ♂, cl. 2.59–2.70 mm, Yala Province, Bannang Sata District, Tham Krachae Stream; 6.2140°N, 101.2040°E; MUMNH-CAR831 • 3 ♀, cl. 3.05–3.62 mm, Pattani Province, Mueang District, Pattani River; 6.8214°N, 101.2447°E; MUMNH-CAR833 • 3 ♀, cl. 3.26–3.54 mm, Krabi Province, Mueang District, unnamed pond; 8.1084°N, 98.9038°E; MUMNH-CAR851 • 4 ♀, cl. 3.28–3.95 mm, Phatthalung Province, Mueang District, unnamed pond; 7.5727°N, 100.1228°E; MUMNH-CAR852 • 5 ♀, cl. 3.25–3.77 mm, 3 ♂, cl. 2.24–2.46 mm, Nakhon Si Thammarat, Phrom Khiri District, unnamed pond; 8.5220°N, 99.9251°E; MUMNH-CAR853 • Songkhla Province, Thale Noi District, Marshland E-SE of Thale Noi; 7.7839°N, 100.2015°E; ZMB 30677 • Nakhon Si Thammarat Province, Sichon District, Thathon Stream; 8.8875°N, 99.9000°E; ZMB 33507 • Nakhon Si Thammarat Province, Ron Phibun District, Mae Setthi Waterfall; 8.1617°N, 99.8236°E; ZMB 29684 • Pattani Province, Nong Chik District; 6.8069°N, 101.1514°E; ZMB 30693.

***Caridina peninsularis* Kemp, 1918**

**Materials.** THAILAND • 3 ♀, cl. 4.45–4.72 mm, Krabi Province, Mueang District, Krabi Stream; 8.1229°N, 98.9253°E; MUMNH-CAR092 • 3 ♀, cl. 4.38–4.65 mm, Krabi Province, Mueang District; 8.0813°N, 98.9189°E; MUMNH-CAR116 • 4 ♀, cl. 4.18–4.60 mm, Chumphon Province, Sawi District, Sawi Stream; 10.2237°N, 99.0120°E; MUMNH-CAR134 • 3 ♀, cl. 4.28–4.52 mm, Ranong Province, Suk Samran District; 9.343706°N, 98.4363°E; MUMNH-CAR149 • 10 ♀, cl. 4.03–5.15 mm, 5 ♂, cl. 2.55–2.88 mm, Ranong Province, Mueang District, Bang Rin Stream; 9.9186°N, 98.6298°E; MUMNH-CAR203, CAR216, CAR222 • 2 ♀, cl. 4.48–4.65 mm, Phuket Province, Thalang District, Bang Prae Stream; 8.0383°N, 98.4034°E; MUMNH-CAR204 • 3 ♀, cl. 4.40–4.82 mm, Songkhla Province, Chana District; 7.0118°N, 100.7279°E; MUMNH-CAR205 • 10 ♀, cl. 4.69–5.62 mm, 5 ♂, cl. 2.68–3.35 mm, Chumphon Province, Sawi District, Sawi Stream; 10.2237°N, 99.0120°E; MUMNH-CAR228 (used for measurement) • 1 ♀, cl. 4.54 mm, Phang Nga Province, Mueang District, Bok Pui Stream; 8.5739°N, 98.4142°E; MUMNH-CAR286 • 1 ♀, cl. 4.63 mm, Krabi Province, Khlong Thom District, Khlong Thom Stream; 7.9181°N, 99.1865°E; MUMNH-CAR308 • 1 ♀, cl. 4.60 mm, Ranong Province, Mueang District, Ngao Noi Stream; 9.8309°N, 98.6247°E; MUMNH-CAR319 • 1 ♀, cl. 4.46 mm, Trang Province, Mueang District; 7.5392°N, 99.5900°E; MUMNH-CAR333 • 5 ♀, cl. 4.38–4.75 mm, 3 ♂, cl. 2.90–3.10 mm, Ranong Province, Kapoe District; 9.6100°N, 98.4791°E; MUMNH-CAR372 • 4 ♀, cl. 4.35–4.79 mm, Phang Nga Province, Takuatung District, Khum Mut Stream; 8.3907°N,98.2995°E; MUMNH-CAR433 • 1 ♀, cl. 4.72, Trang Province, Kantang District, Huai Dam Stream; 7.40324°N, 99.4351°E; MUMNH-CAR442 • 1 ♀, cl. 4.50 mm, Trang Province, Palian District, Lak Khan Stream; 7.2190°N, 99.7183°E; MUMNH-CAR446 • 3 ♀, cl. 4.24–4.83 mm, Satun Province, La-ngu District, La-ngu Stream; 6.9069°N, 99.8115°E; MUMNH-CAR451 (used for measurement) • 5 ♀, cl. 4.03–4.47 mm, 2 ♂, cl. 2.35–3.06 mm, Nakhon Si Thammarat Province, Sichon District, Thathon Stream; 8.8876°N, 99.8997°E; MUMNH-CAR459 (used for measurement) • 5 ♀, cl. 4.82–5.75 mm, 3 ♂, cl. 2.53–2.99 mm, Songkhla Province, Hat Yai District, Thale Sap Songkhla (Songkhla Lake); 7.1552°N, 100.4686°E; MUMNH-CAR496 (used for measurement) • 5 ♀, cl. 3.42–4.51 mm, 3 ♂, cl. 2.48–2.59 mm, Phang Nga Province, Takuatung District, Khum Mut Stream; 8.3953°N, 98.4324°E; MUMNH-CAR633 • 10 ♀, cl. 3.78–5.19 mm, 4 ♂, cl. 2.53–2.82 mm, Phang Nga Province, Takua Pa District, Takua Pa River; 8.8541°N, 98.3541°E; MUMNH-CAR635 (used for measurement) • 3 ♀, cl. 3.54–3.77 mm, Ranong Province, Kapoe District, reservoir near the road; 9.6168°N, 98.6318°E; MUMNH-CAR648 • 10 ♀, cl. 3.75–4.29 mm, 4 ♂, cl. 2.65–2.98 mm, Surat Thani Province, Khiri Rat Nikhom District, Hin Kong Stream; 8.8427°N, 98.9824°E; MUMNH-CAR783, CAR792 • Nakhon Si Thammarat Province, Sichon District, Tha Ruea Ri Stream; 9.0078°N, 99.8217°E; ZMB 30685 • Nakhon Si Thammarat Province, Khanom District, Khao Phlai Dam; 9.0877°N, 99.9108°E; ZMB 32097 • Nakhon Si Thammarat Province, Sichon District, Thathon Stream; 8.8875°N, 99.9000°E; ZMB 33505, ZMB33506.

***Caridina* *laevis* Heller, 1862**

**Materials.** THAILAND • 1 ♀, cl. 5.54 mm, Trang Province, Palian District, Lak Khan Stream; 7.2331°N, 99.7256°E; ZMB 30683 • 1 ♀, cl. 5.02 mm, Phatthalung Province, Khuan Khanun District, Phatthalung Botanical Garden; 7.7342°N, 100.1513°E; ZMB 30702.

***Caridina* *excavatoides* Johnson, 1961**

**Materials.** THAILAND • 5 ♀, cl. 2.79–3.59 mm, 2 ♂, cl. 2.17–2.27 mm, Songkhla Province, Ranot District, Thale Noi; 7.7837°N, 100.2030°E; MUMNH-CAR490 (used for measurement) • Nakhon Sri Thammarat Province, Hua Sai District; 8.0172°N, 100.3006°E; ZMB 29686 • Songkhla Province, Thale Noi District, Marshland E-SE of Thale Noi; 7.7839°N, 100.2015°E; ZMB 30195 • Phatthalung Province, Mueang District, Lampam Stream; 7.6261°N, 100.1558°E; ZMB 30196 • Pattani Province, Nong Chik District; 6.8069°N, 101.1514°E; ZMB 30198 • 10 ♀, cl. 2.45–2.91 mm, 5 ♂, cl. 2.02–2.48 mm, Nakhon Sri Thammarat Province, Thung Song District, Tha-Luang Stream; 08.0550°N, 99.6424°E; MUMNH-CAR616 (used for measurement) • 3 ♀, cl. 2.38–2.88 mm, 3 ♂, cl. 2.20–2.47 mm, Surat Thani Province, Khiri Rat Nikhom District, Hin Kong Stream; 8.8427°N, 98.9824°E; MUMNH-CAR794.

***Caridina temasek* Choy & Ng, 1991**

**Materials.** THAILAND • 5 ♀, cl. 2.76–3.35 mm, 3 ♂, cl. 2.15–2.41 mm, Songkhla Province; Hat Yai District, Wat Stream; 6.9812°N, 100.2996°E; MUMNH-CAR812 (used for measurement) • 10 ♀, cl. 3.03–3.81 mm, 5 ♂, cl. 2.25–2.59 mm, Pattani Province, Mueang District, Pattani River; 6.8214°N, 101.2447°E; MUMNH-CAR835 (used for measurement) • Phatthalung Province, Kong Ra District, Ban Phut Stream; 7.3717°N, 99.9758°E; ZMB 29687 • Nakhon Si Thammarat Province, Chang Klang District, River after limestone cave (-200m); 8.3342°N, 99.6514°E; ZMB 29674, ZMB 32130.

***Caridina* cf. *rangoona* Cai & Ng, 2000**

**Materials.** THAILAND • 6 ♀, cl. 2.56–4.00 mm, 3 ♂, cl. 2.00–2.47 mm, Ranong Province, La-un-District, La-un Stream; 10.1097°N, 98.7595°E; MUMNH-CAR154 (used for measurement) • 5 ♀, cl. 2.40–3.37 mm, 5 ♂, cl. 2.12–2.57 mm, Phang Nga Province, Takuatung District, Khum Mut Stream; 8.3907°N,98.2995°E; MUMNH-CAR434 (used for measurement) • Trang Province, Palian District, Lak Khan Stream; 7.2190°N, 99.7183°E; MUMNH-CAR444, MUMNH-A187 • 5 ♀, cl. 2.36–2.95 mm, 3 ♂, cl. 2.04–2.53 mm, Krabi Province, Mueang District, Sra-kaew Stream; 8.1692°N, 98.8070°E; MUMNH-CAR436, CAR437 • Satun Province, Mueang District, Chalung Stream; 6.7379°N, 100.0610°E; MUMNH-CAR448S • 5 ♀, cl. 2.13–2.88 mm, Satun Province, La-ngu District, La-ngu Stream; 6.9069°N, 99.8115°E; MUMNH-CAR454, MUMNH-CAR457, MUMNH-A191 • 1 ♀, cl. 2.30 mm, Krabi Province, Khlong Thom District, Thom Stream (connected to Emerald Pool); 7.9230°N, 99.2598°E; MUMNH-CAR506.

***Caridina johnsoni* Cai, Ng & Choy, 2007**

Materials. THAILAND • 3 ♀, cl. 2.45–2.70 mm, Phetchaburi Province, Nong Ya Plong District, Mae Prachan Stream; 13.1092°N, 99.7230°E, MUMNH-CAR126 • 2 ♀, cl. 2.75–2.98 mm, Phetchaburi Province, Tha Yang District, Phet Dam; 12.9138°N, 99.8559°E; MUMNH-CAR127 • 4 ♀, cl. 3.00–3.21 mm, 1 ♂, cl. 2.26 mm, Prachuap Khiri Khan Province, Kui Buri District, Kui Stream; 12.0561°N, 99.8601°E; MUMNH-CAR221 • 3 ♀, cl. 2.42–2.90 mm, Phetchaburi Province, Tha Yang District, Phetchaburi River; 12.9184°N, 99.8560°E; MUMNH-CAR299 • 1 ♀, cl. 2.58 mm, Ranong Province, Kra Buri District, Lam Liang Stream; 10.3100°N, 98.795200°E; MUMNH-CAR341 • 10 ♀, cl. 2.46–3.21 mm, 5 ♂, cl. 1.69–2.13 mm, Prachuap Khiri Khan Province, Hua Hin District, Sam Pran Nam Stream; 12.6049°N, 99.8591°E; MUMNH-CAR380, CAR383, CAR612 (used for measurement) • 1 ♀, cl. 2.71 mm, Prachuap Khiri Khan Province, Hua Hin District, Pran Buri River; 12.4780°N, 99.7982°E; MUMNH-CAR393 • 3 ♀, cl. 2.40–2.75 mm, Satun Province, La-ngu District, La-ngu Stream; 6.9069°N, 99.8115°E; MUMNH-CAR455 • 10 ♀, cl. 2.80–3.42 mm, 5 ♂, cl. 1.80–2.31 mm, Surat Thani, Mueang District, Phunphin Stream; 9.1936°N, 99.2816°E; MUMNH-CAR470, CAR471 (used for measurement) • 5 ♀, cl. 2.45–3.02 mm, 2 ♂, cl. 1.65–1.99 mm, Phatthalung Province, Si Banphot District, Tha-nae Stream; 7.7071°N, 99.8412°E; MUMNH-CAR487 • 10 ♀, cl. 2.46–3.15 mm, 5 ♂, cl. 1.68–2.22 mm, Songkhla Province, Ranot District, Thale Noi; 7.7837°N, 100.2030°E; MUMNH-CAR491 (used for measurement) • 3 ♀, cl. 2.33–2.61 mm, Nakhon Sri Thammarat, Bangkhan District, Trang River; 7.9451°N, 99.5781°E; MUMNH-CAR620 • 3 ♀, cl. 2.23–2.86 mm, 2 ♂, cl. 1.60–2.10 mm, Trang Province, Mueang District, Trang River; 7.6117°N, 99.5188°E; MUMNH-CAR624 • 1 ♀, cl. 2.72 mm, Trang Province, Mueang District, Trang River; 7.5957°N, 99.5807°E; MUMNH-CAR627 • 5 ♀, cl. 2.53–3.24 mm, 3 ♂, cl. 1.69–2.30 mm, Chumphon Province, Sawi District Sawi Stream;10.2237°N, 99.0121°E; MUMNH-CAR704 • 5 ♀, cl. 2.23–2.86 mm, Surat Thani Province, Khiri Rat Nikhom District, Hin Kong Stream; 8.8427°N, 98.9824°E; MUMNH-CAR784 • 5 ♀, cl. 2.56–3.19 mm, Surat Thani Province, Phrasaeng District, I-Pun Stream 8.6212°N, 98.9666°E; MUMNH-CAR789 • 5 ♀, cl. 2.65–3.38 mm, 4 ♂, cl. 1.86–2.34 mm, Pattani Province, Mueang District, Pattani River; 6.8214°N, 101.2447°E; MUMNH-CAR834 (used for measurement) • 3 ♀, cl. 2.29–2.83 mm, Phang Nga Province, Mueang District, unnamed pond; 8.4831°N, 98.5238°E; MUMNH-CAR850 • Surat Thani Province, Mueang District, Phunphin Stream; 9.1906°N, 99.2814°E; ZMB 30673 • Songkhla Province, Thale Noi District, Marshland E-SE of Thale Noi; 7.7839°N, 100.2015°E; ZMB 30675 • Nakhon Sri Thammarat Province, Hua Sai District; 8.0172°N, 100.3006°E; ZMB 30687 • Phatthalung Province, Mueang District, Lampam Stream; 7.6261°N, 100.1558°E; ZMB 30689 • Surat Thani Province, Mueang District; 9.1803°N, 99.2747°E; ZMB 30692 • Songkhla Province, Ranot District; Ranot Beach; 7.7850°N, 100.3619°E; ZMB 30700.

***Caridina nangroma* sp. nov.**

Materials. THAILAND • 3 ♀, cl. 2.36–2.95 mm, Nakhon Si Thammarat Province, Chawang District, Kra-Bead Stream; 8.594722°N, 99.46556°E; MUMNH-CAR135, CAR371 • Songkhla Province, Sathing Phra District, Cha Thing Phra Canal; 7.473828°N, 100.4259°E; MUMNH-CAR136 • 5 ♀, cl. 2.36–2.93 mm, 3 ♂, cl. 1.93–2.41 mm, Prachuap Khiri Khan Province, Sam Roi Yot District, pond near the road; 12.3017°N, 99.9373°E; MUMNH-CAR267 • 2 ♀, cl. 2.36–2.85 mm, Prachuap Khiri Khan Province, Bang Saphan District, Wang Hin Stream; 11.3243°N, 99.5538°E; MUMNH-CAR329 • 12 ♀, cl. 2.10–2.87 mm, 5 ♂, cl. 1.70–2.19 mm, Prachuap Khiri Khan Province, Hua Hin District, Pranburi River; 12.4780°N, 99.7982°E; MUMNH-CAR375, CAR378, CAR404, CAR405 ; • 3 ♀, cl. 2.63–2.69 mm, Prachuap Khiri Khan Province, Hua Hin District, Sam Pran Nam Stream; 12.6049°N, 99.8591°E; MUMNH-CAR381 • 2 ♀, cl. 2.39–2.78 mm, Prachuap Khiri Khan Province, Mueang District, Wa Thon Stream; 11.7509°N, 99.6968°E; MUMNH-CAR394 • 4 ♀, cl. 2.36–2.81 mm, Prachuap Khiri Khan, Thap Sakae District, Phrak Sai Stream; 11.619471°N, 99.63707°E; MUMNH-CAR402 • 5 ♀, cl. 2.38–2.92 mm, 3 ♂, cl. 1.96–2.34 mm, Prachuap Khiri Khan, Thap Sakae District, Thap Sakae Stream; 11.49837°N, 99.586261°E; MUMNH-CAR408, CAR409 • 5 ♀, cl. 2.40–2.90 mm, 3 ♂, cl. 2.00–2.48 mm, Prachuap Khiri Khan Province, Hua Hin District, Palao Stream; 12.5059°N, 99.5166°E; MUMNH-CAR413 • 4 ♀, cl. 2.40–2.76 mm, Prachuap Khiri Khan Province, Kui Buri District, Kui Stream; 12.0561°N, 99.8601°E; MUMNH-CAR415 • 1 ♀, cl. 2.99 mm, Prachuap Khiri Khan Province, Sam Roi Yot District, stream near Wat Tham Ko Phai Temple; 12.189319°N, 99.9207°E; MUMNH-CAR419 • 1 ♀, cl. 2.47 mm, Trang Province, Palian District, Lak Khan Stream; 7.2190°N, 99.7183°E; MUMNH-CAR445 • 8 ♀, cl. 2.73–3.13 mm, 3 ♂, cl. 2.18–2.50 mm, Satun Province, Mueang District, Chalung Stream; 6.7379°N, 100.0609°E; MUMNH-CAR448 • 5 ♀, cl. 2.60–3.10 mm, 3 ♂ 2.29–2.41 mm, Chumphon Province, Lamae District, Lamae Stream; 9.7369°N, 98.9900°E; MUMNH-CAR474 • 5 ♀, cl. 2.53–3.28 mm, 3 ♂, cl. 2.30–2.59 mm, Nakhon Si Thammarat Province, Lan Saka District, Yuad Stream; 8.3861°N, 99.8167°E; MUMNH-CAR480 • 5 ♀, cl. 2.38–3.30 mm, 3 ♂ cl. 2.21–2.46 mm, Nakhon Sri Thammarat Province, Cha-uat District, Wangkiean Stream; 7.9750°N, 99.9170°E; MUMNH-CAR484 • 10 ♀, cl. 2.25–2.96 mm, 5 ♂, cl. 2.12–2.66 mm, Phatthalung Province, Khuan Khanun District, Phatthalung Botanical Garden; 7.7353°N, 100.1509736°E; MUMNH-CAR492 • 3 ♀, cl. 2.41–2.74 mm, Songkhla Province, Rattaphum District, Ton Stream; 7.1432°N, 100.1231°E; MUMNH-CAR501 • 3 ♀, cl. 2.50–3.00 mm, Songkhla Province, Ranot District, Thale Noi Non-Hunting Area; 7.7838°N, 100.2031°E; MUMNH-CAR598 • 5 ♀, cl. 2.73–3.15 mm, 3 ♂, cl. 2.24–2.56 mm, Nakhon Sri Thammarat, Bangkhan District, Trang River; 7.9786°N, 99.6041°E; MUMNH-CAR618 • Nakhon Sri Thammarat, Bangkhan District, Trang River; 7.9451°N, 99.5781°E; MUMNH-CAR621 • 5 ♀, cl. 2.63–3.39 mm, 3 ♂, cl. 2.35–2.59 mm, Trang District, Huai Yot District, Trang River 7.8153°N, 99.5317°E; MUMNH-CAR630 • 1 ♀, cl. 2.78 mm, Chumphon Province, Tha Sae District, A-Tham Stream; 10.8791°N, 99.1306°E; MUMNH-CAR702 • 1 ♀, cl. 2.78 mm, 5 ♀, cl. 2.50–3.18 mm, 1 ♂, cl. 2.42 mm, Surat Thani Province, Chai Buri District, Sok Stream; 8.5068°N, 99.0992°E; MUMNH-CAR797 • 3 ♀, cl. 2.39–3.05 mm, 1 ♂, cl. 2.59 mm, Nakhon Si Thammarat Province, Bang Khan District, Lam Dang Stream; 7.9499°N, 99.5296°E; MUMNH-CAR804 • 1 ♀, cl. 2.89 mm, Nakhon Si Thammarat Province, Phrom Khiri District, unnamed pond; 8.5185°N, 99.9238°E; MUMNH-CAR855 • 3 ♀, cl. 2.83–3.10 mm, 2 ♂, cl. 2.45–2.59 mm, Surat Thani Province, Mueang District, Tha Kruat Stream; 9.0633°N, 99.3431°E; MUMNH-CAR856 • Nakhon Si Thammarat Province, Ron Phibun District, Mae Setthi Waterfall; 8.1617°N, 99.8236°E; ZMB 29683 • Phetchaburi Province, Mueang District, Hua Chang Stream; 13.0056°N, 100.0601°E; ZMB 30674 • Trang Province, Palian District, Lak Khan Stream; 7.2331°N, 99.7256°E; ZMB 30682 • Surat Thani Province, Ban Na San District, stream near the road; 8.7064°N, 99.3153°E; ZMB 30695 • Nakhon Si Thammarat Province, Hua Sai District, Hua Lam Phu Stream; 8.0511°N, 100.2469°E; ZMB 30696.

***Caridina srivijaya* sp. nov.**

Materials. THAILAND • 10 ♀, cl. 2.29–2.93 mm, 6 ♂, cl. 1.52–2.22 mm, Surat Thani Province, Phrasaeng District, Bang Sawan Spring; 8.6730°N, 98.9798°E; MUMNH-CAR502.

***Caridina takkola* sp. nov.**

Materials. THAILAND • 10 ♀, cl. 2.97–3.90 mm, 6 ♂, cl. 1.65–2.51 mm, Surat Thani Province, Mueang District, Phunphin Stream; 9.1936°N, 99.2816°E; MUMNH-CAR469 • 8 ♀, cl. 2.65–3.48 mm, 5 ♂, cl. 1.76–1.92 mm, Songkhla Province, Hat Yai District, Thale Sap Songkhla (Songkhla Lake); 7.1552°N, 100.4686°E; MUMNH-CAR495 • Surat Thani Province, Mueang District, Phunphin Stream; 9.1803°N, 99.2747°E; ZMB 30692.

***Caridina tambralinga* sp. nov.**

Materials. THAILAND • Satun Province, 2 ♀, cl. 2.07–2.17 mm, Mueang District, Nong Plak Phra Ya Pond; 6.7436°N, 100.0386°E; MUMNH-CAR240 • 3 ♀, cl. 2.15–2.50 mm, Satun Province, La-ngu District, La-ngu Stream; 6.9069°N, 99.8115°E; MUMNH-CAR456 • 5 ♀, cl. 2.25–2.67 mm, 2 ♂, cl. 1.59–1.78 mm, Nakhon Si Thammarat Province, Sichon District, Thathon Stream; 8.8876°N, 99.8997°E: MUMNH-CAR461 • 3 ♀, cl. 2.01–2.38 mm, Nakhon Si Thammarat Province, Sichon District, Tha-kwai Stream; 8.9467°N, 99.7567°E; MUMNH-CAR476, CAR477 • Nakhon Si Thammarat Province, Nopphitam District, Waad Stream; 8.8045°N, 99.5884°E; MUMNH-CAR478 • Songkhla Province, Ranot District, Thale Noi Non-Hunting Area; 7.7838°N, 100.2031°E; 5 ♀, cl. 2.25–2.63, 2 ♂, cl. 1.64–1.87 mm, MUMNH-CAR489 • 3 ♀, cl. 2.31–2.39, 1 ♂, cl. 1.75 mm, Phatthalung Province, Pa Bon District, Yao Stream; 7.2399°N, 100.2838°E; MUMNH-CAR494 • 3 ♀, cl. 2.20–2.51 mm, Nakhon Si Thammarat Province, Phrom Khiri District, unnamed pond; 8.5185°N, 99.9238°E; MUMNH-CAR641 • 5 ♀, cl. 2.15–2.78, 2 ♂, cl. 1.67–1.72 mm, Nakhon Si Thammarat Province, Phrom Khiri District, unnamed pond; 8.5220°N, 99.9251°E; MUMNH-CAR854 • Nakhon Si Thammarat Province, Mueang District, unnamed stream; 8.4844°N, 99.8411°E; ZMB 29763 • Pattani Province, Nong Chik District, Pattani River; 6.8069°N, 101.1514°E; ZMB 30698 • Surat Thani Province, Kanchanadit District, stream near the road; 9.1383°N, 99.4968°E; ZMB 30704 • Narathiwat Province, Su-ngai Kolok District, To Daeng peat swamp; 6.0716°N, 101.9650°E; ZMB 31662.
